# Supplementary figures and images for: Defining the interactome of the human mitochondrial ribosome identifies SMIM4 and TMEM223 as respiratory chain assembly factors (part 1 of 2)
Source: eLife. 2021 Dec 31;10:e68213. doi: 10.7554/eLife.68213 (PMC8719881; doi:10.7554/eLife.68213)

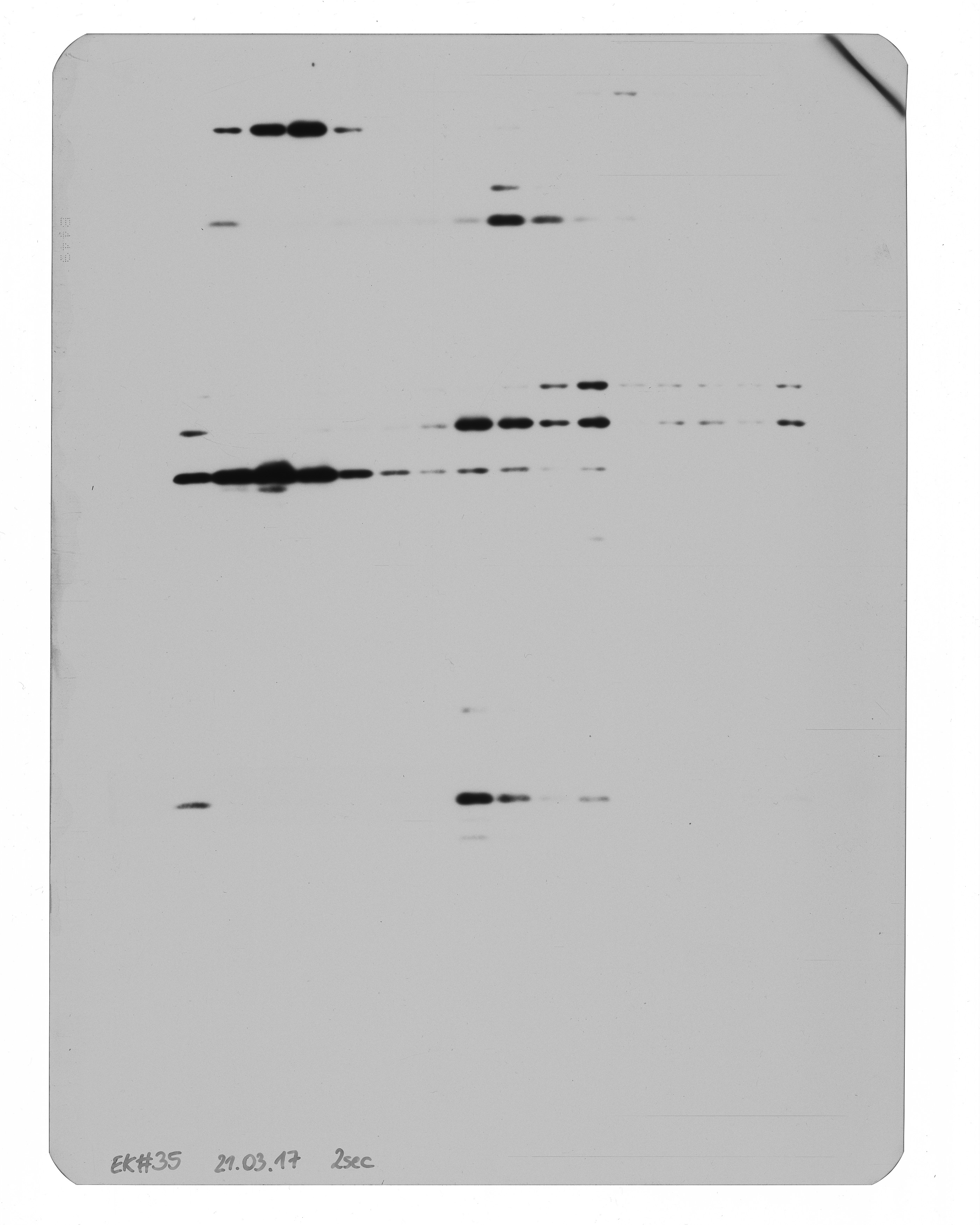

Supplement: Figure 1—source data 1. [file elife-68213-fig1-data1.zip › Figure_1_source_data/Figure_1_source_data_1_Figure_1A/Original_files/21.03.17 003.jpg]

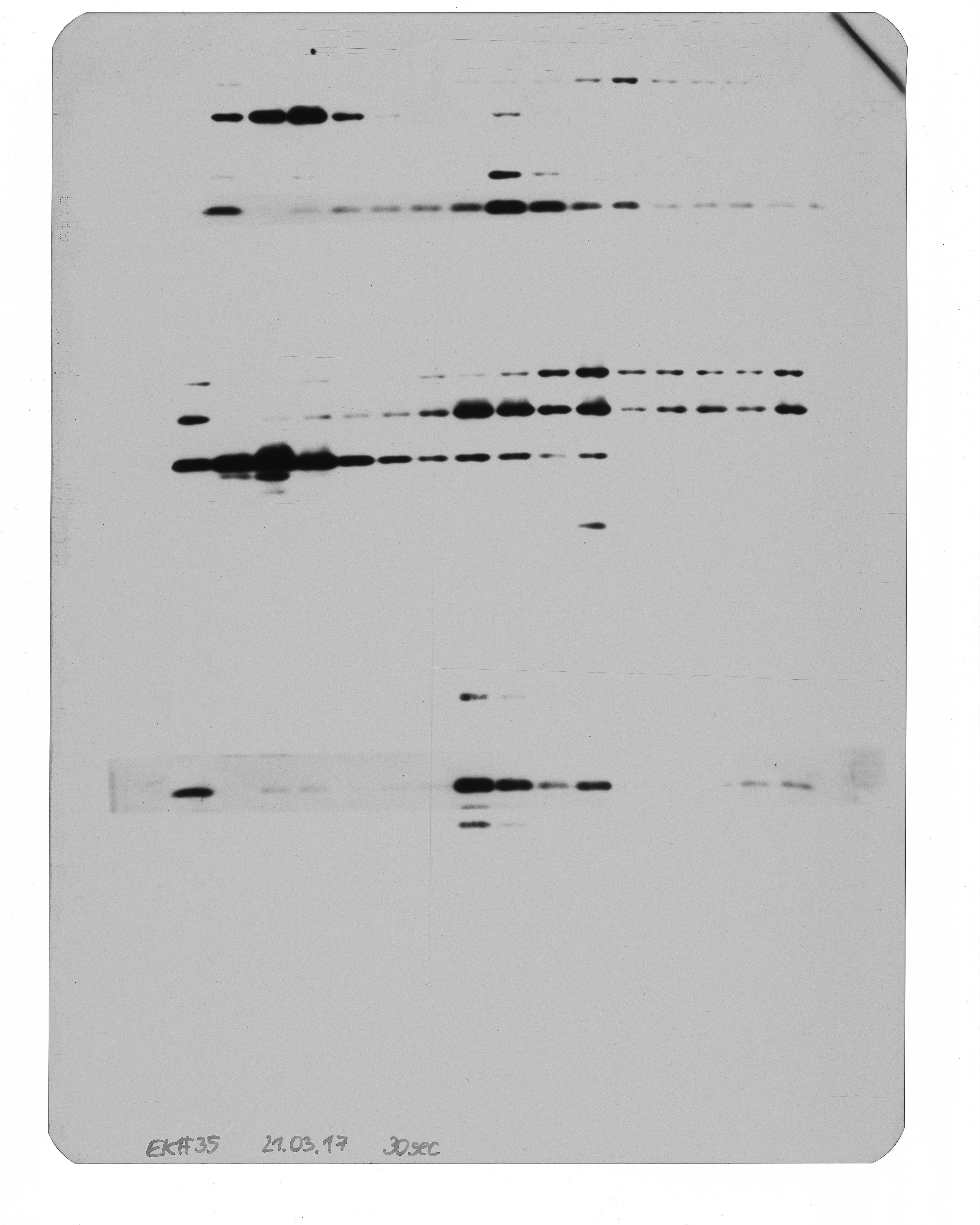

Supplement: Figure 1—source data 1. [file elife-68213-fig1-data1.zip › Figure_1_source_data/Figure_1_source_data_1_Figure_1A/Original_files/21.03.17 004.jpg]

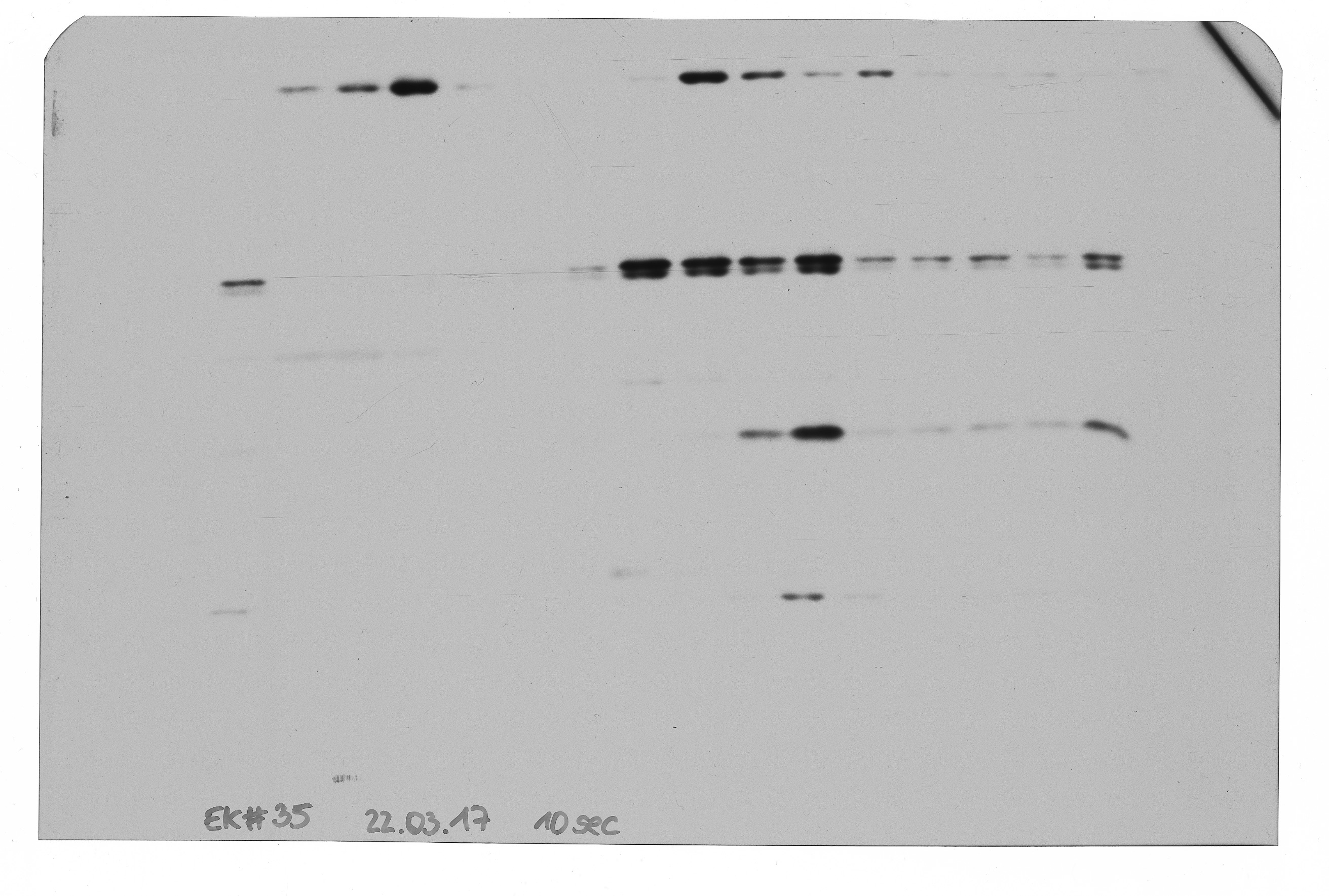

Supplement: Figure 1—source data 1. [file elife-68213-fig1-data1.zip › Figure_1_source_data/Figure_1_source_data_1_Figure_1A/Original_files/22.03.17 002.jpg]

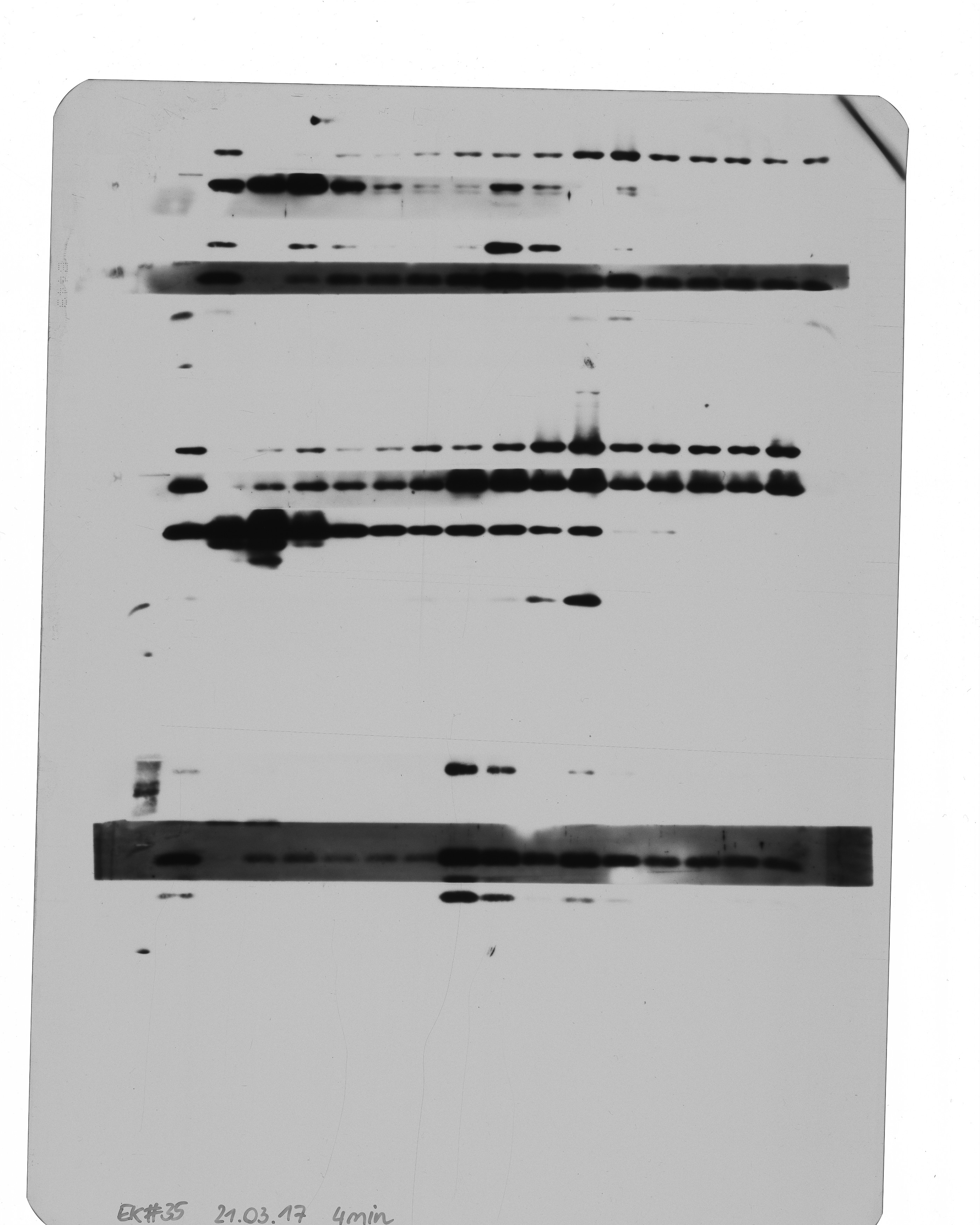

Supplement: Figure 1—source data 1. [file elife-68213-fig1-data1.zip › Figure_1_source_data/Figure_1_source_data_1_Figure_1A/Original_files/21.03.17 002.jpg]

Figure 1 source data 1 related to Figure 1A

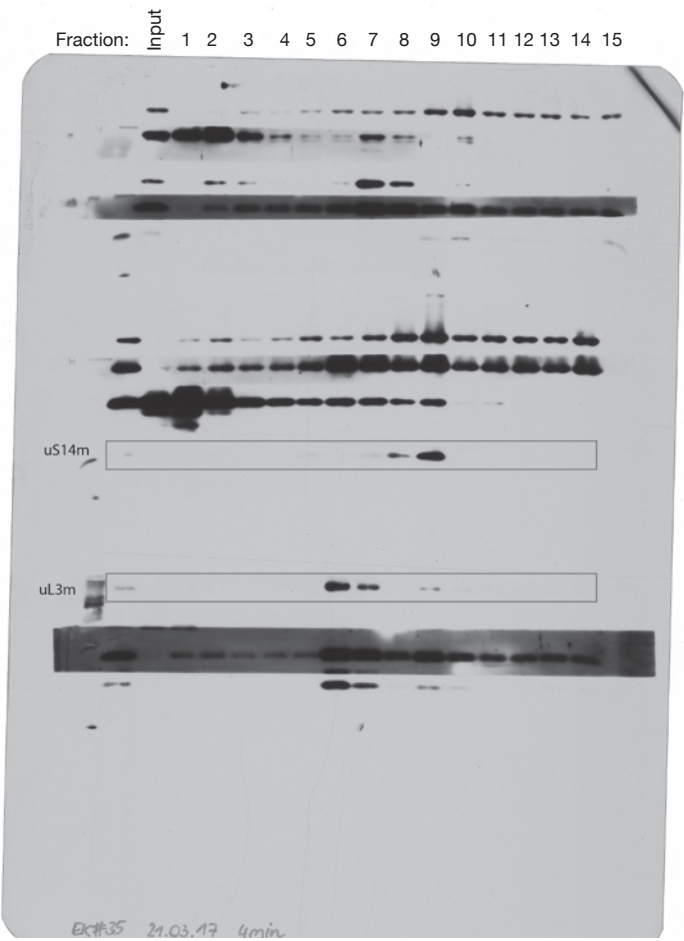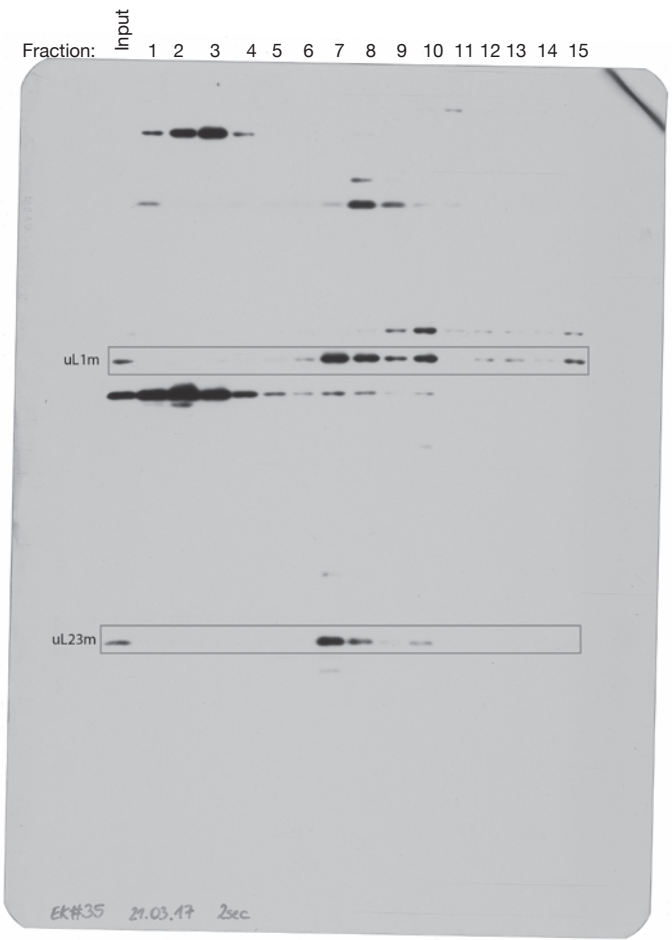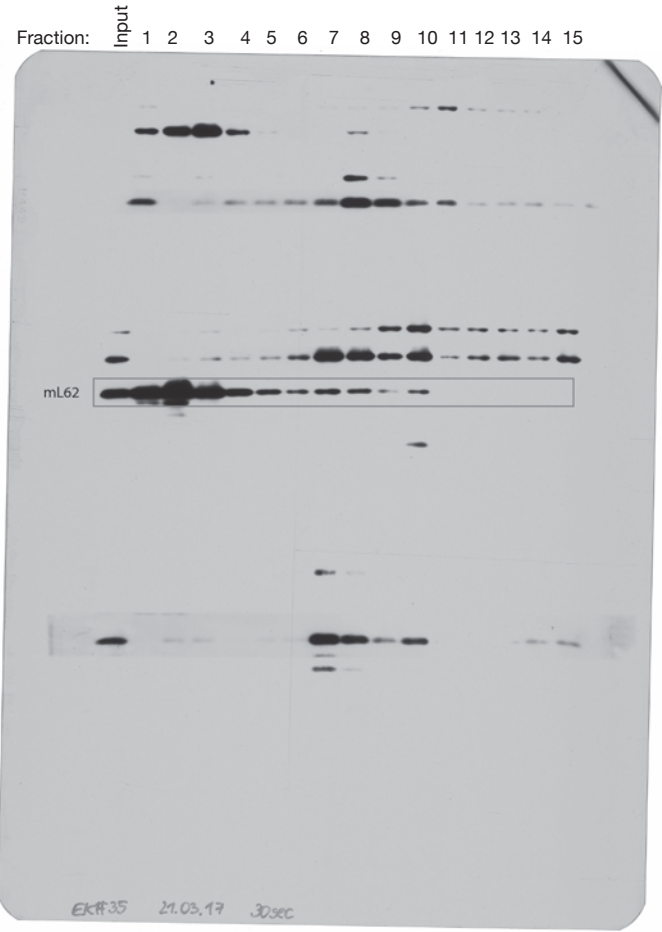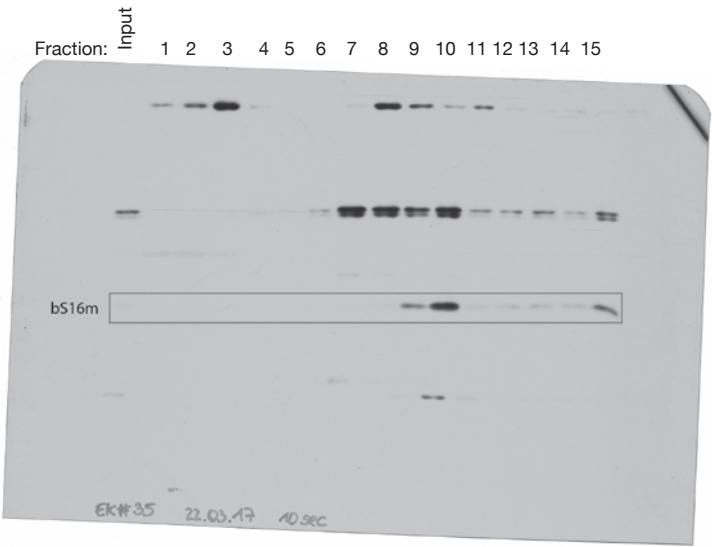

Supplement: Figure 1—source data 1. [file elife-68213-fig1-data1.zip › Figure_1_source_data/Figure_1_source_data_1_Figure_1A/Data_labelled/Figure_1_source_data_1_Figure_1A.pdf]

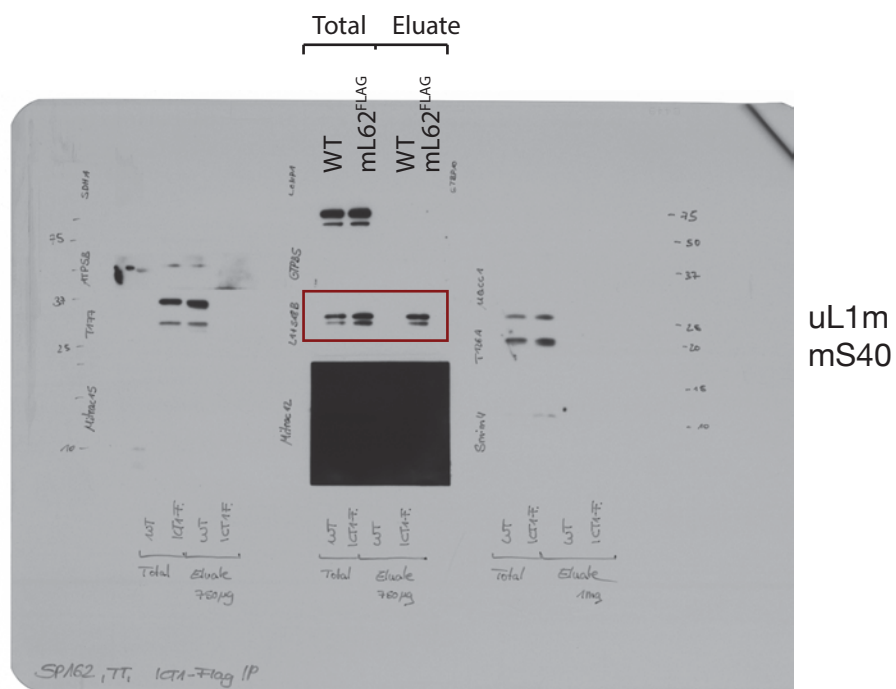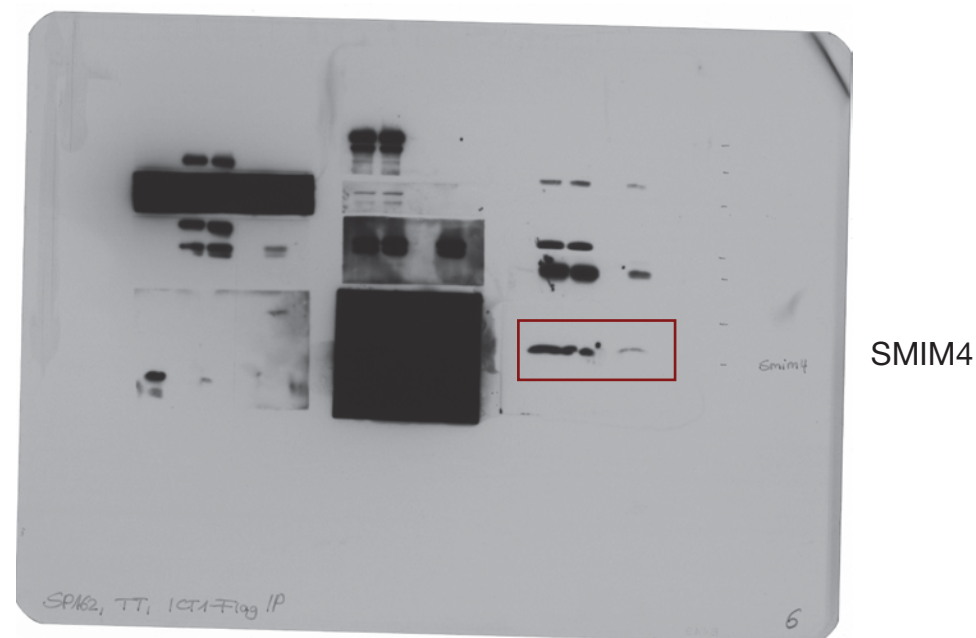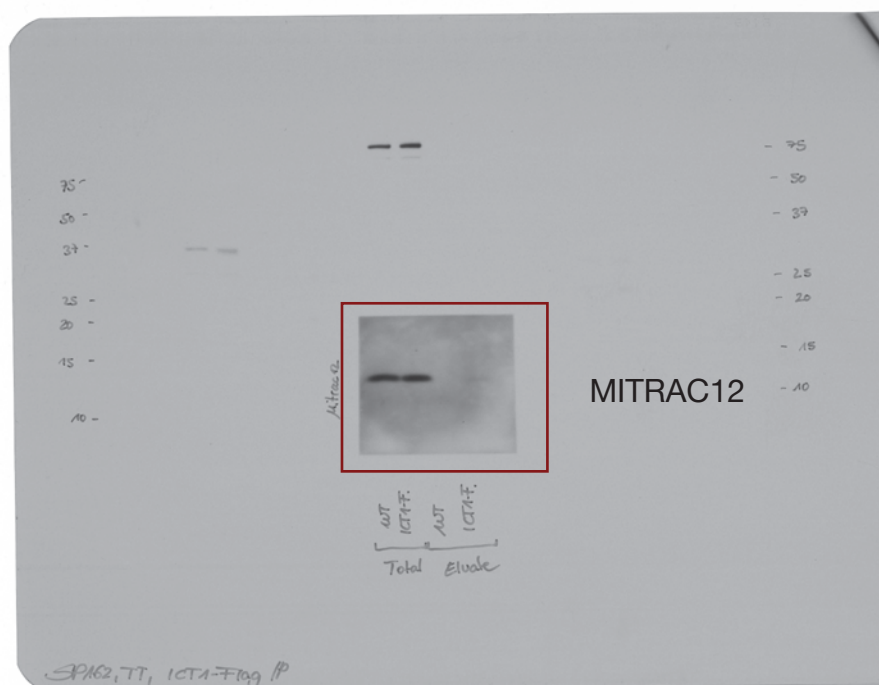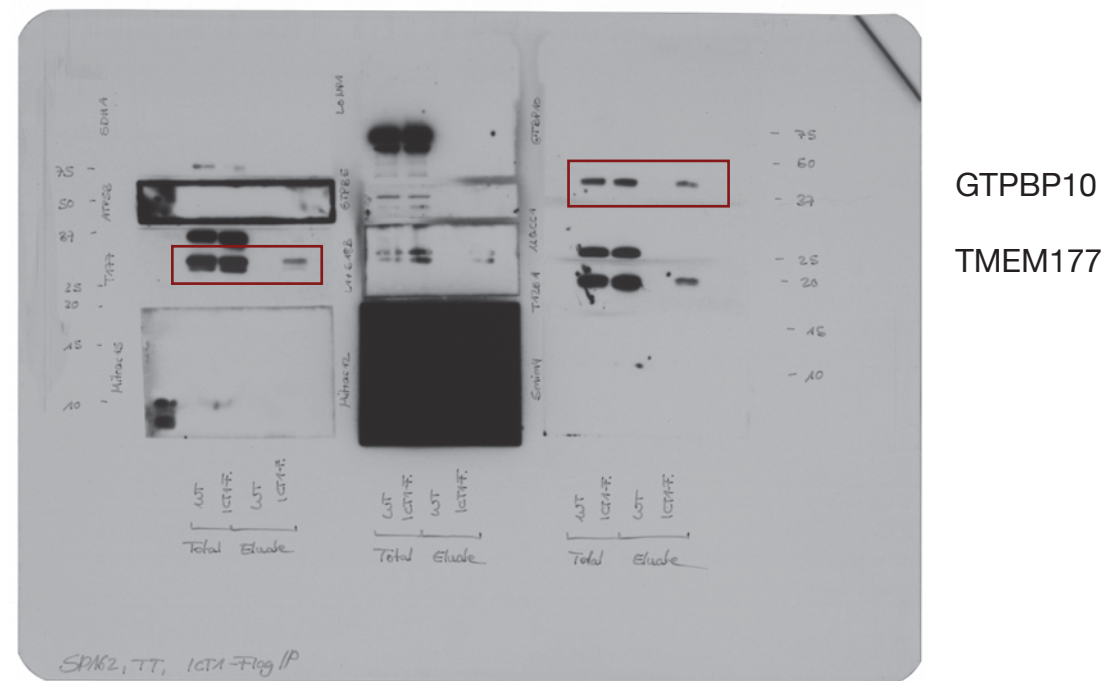

Figure\_1\_source\_data\_2\_Figure\_1D

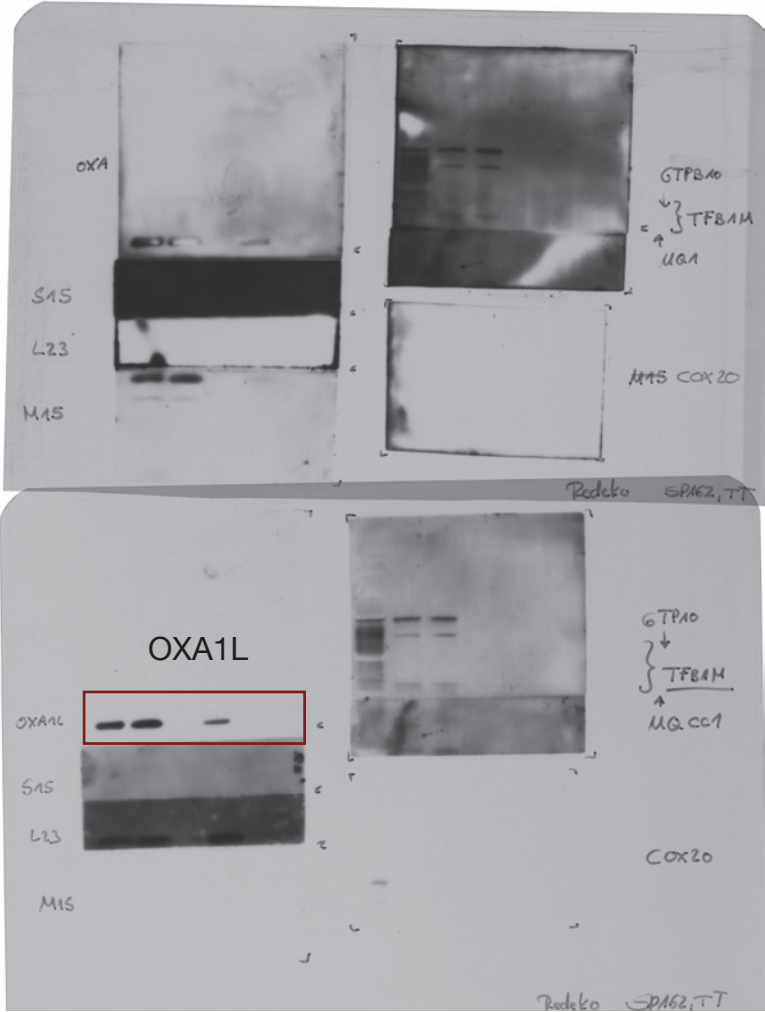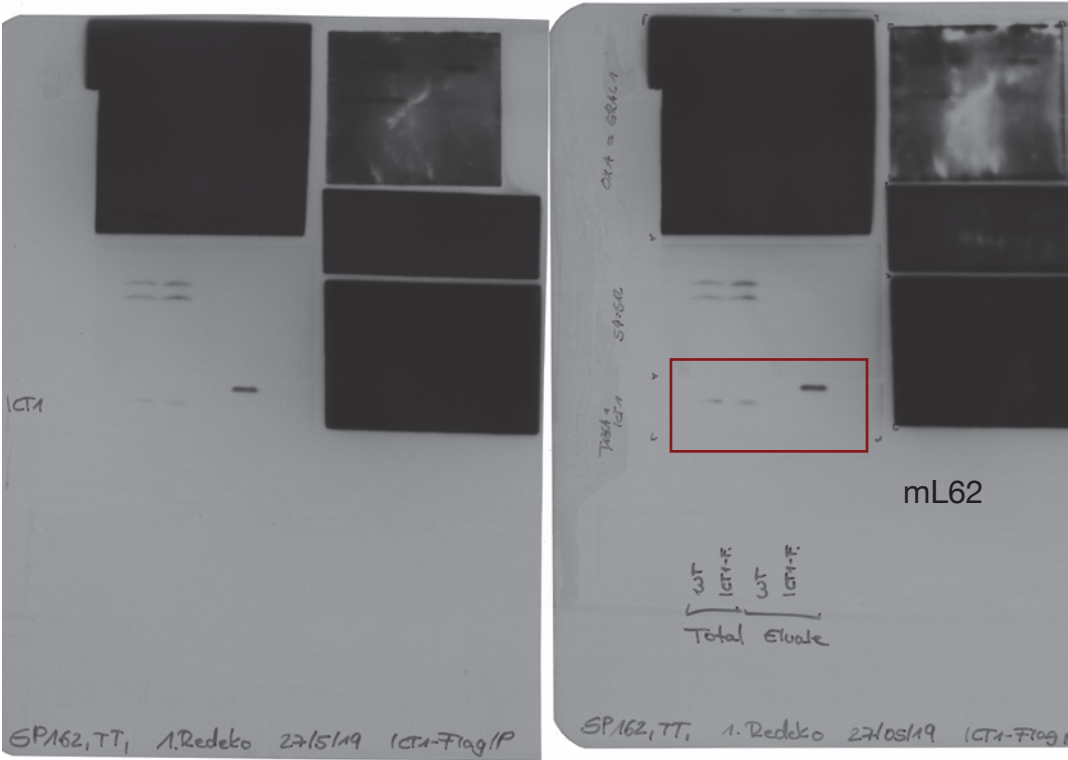

Supplement: Figure 1—source data 1. [file elife-68213-fig1-data1.zip › Figure_1_source_data/Figure_1_source_data_2_Figure_1D/Data_labelled/Figure_1_source_data_2_Figure_1D.pdf]

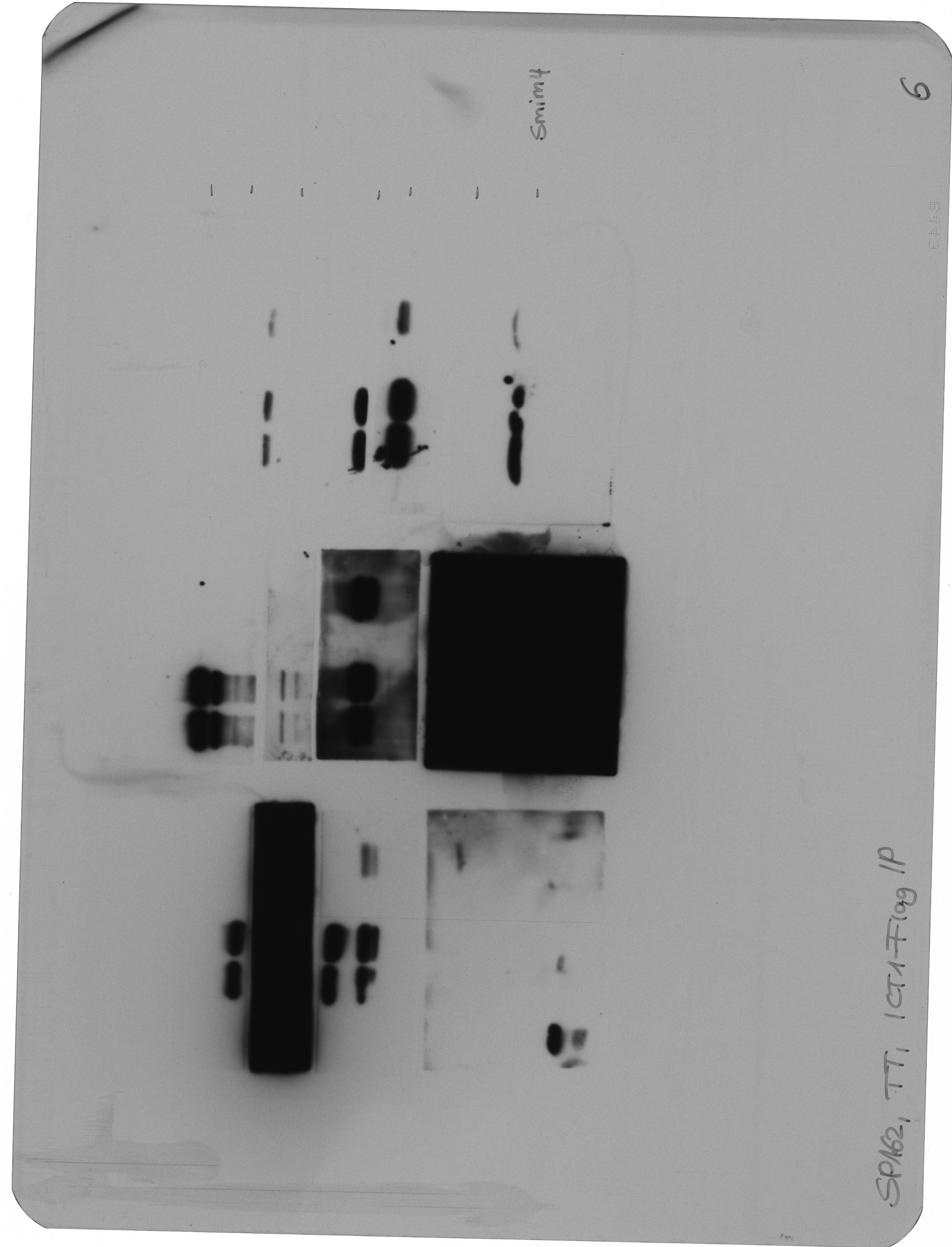

Supplement: Figure 1—source data 1. [file elife-68213-fig1-data1.zip › Figure_1_source_data/Figure_1_source_data_2_Figure_1D/Original_files/SP162 ICT1-IP006.jpg]

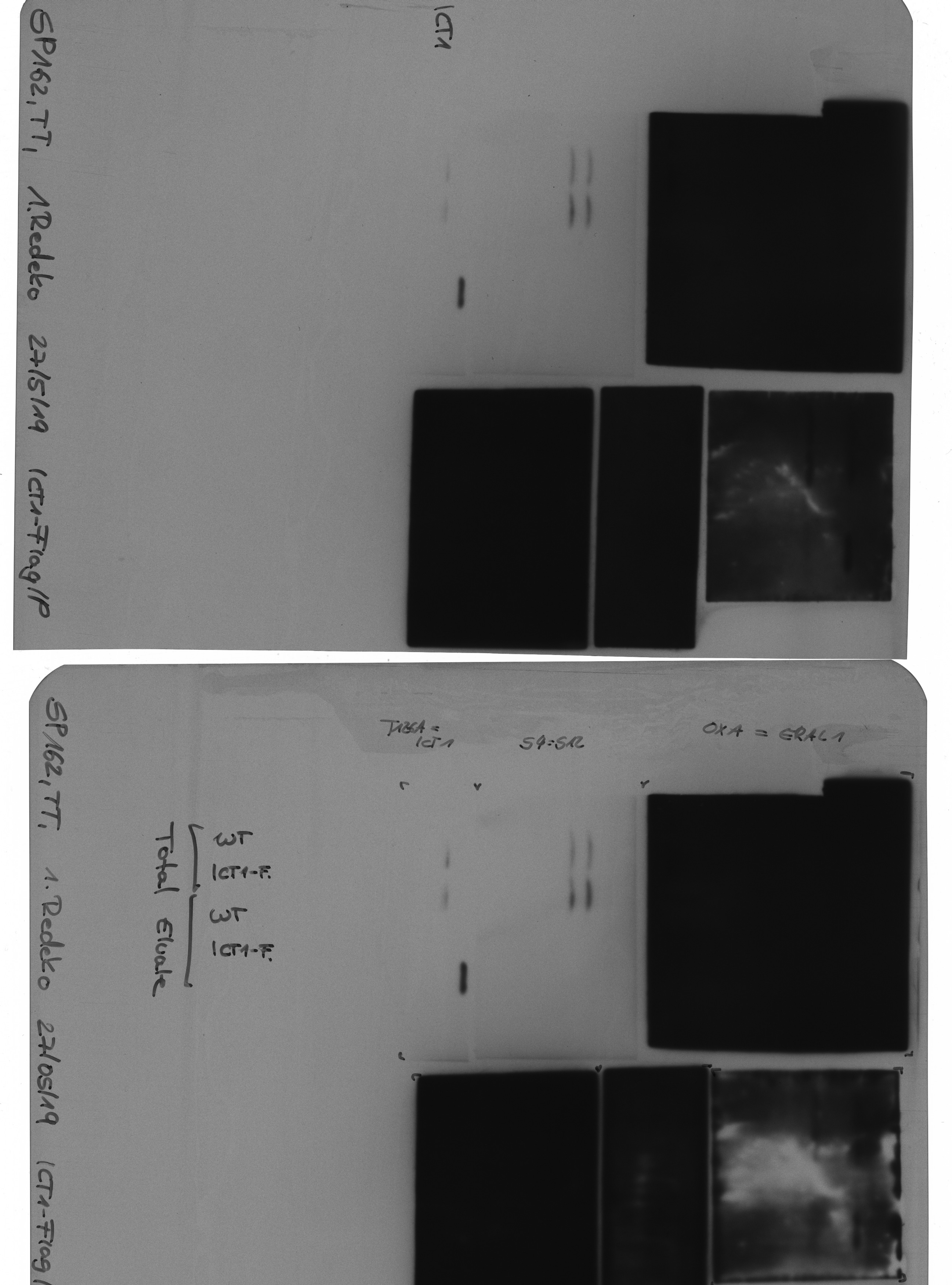

Supplement: Figure 1—source data 1. [file elife-68213-fig1-data1.zip › Figure_1_source_data/Figure_1_source_data_2_Figure_1D/Original_files/SP162 ICT1-IP07.jpg]

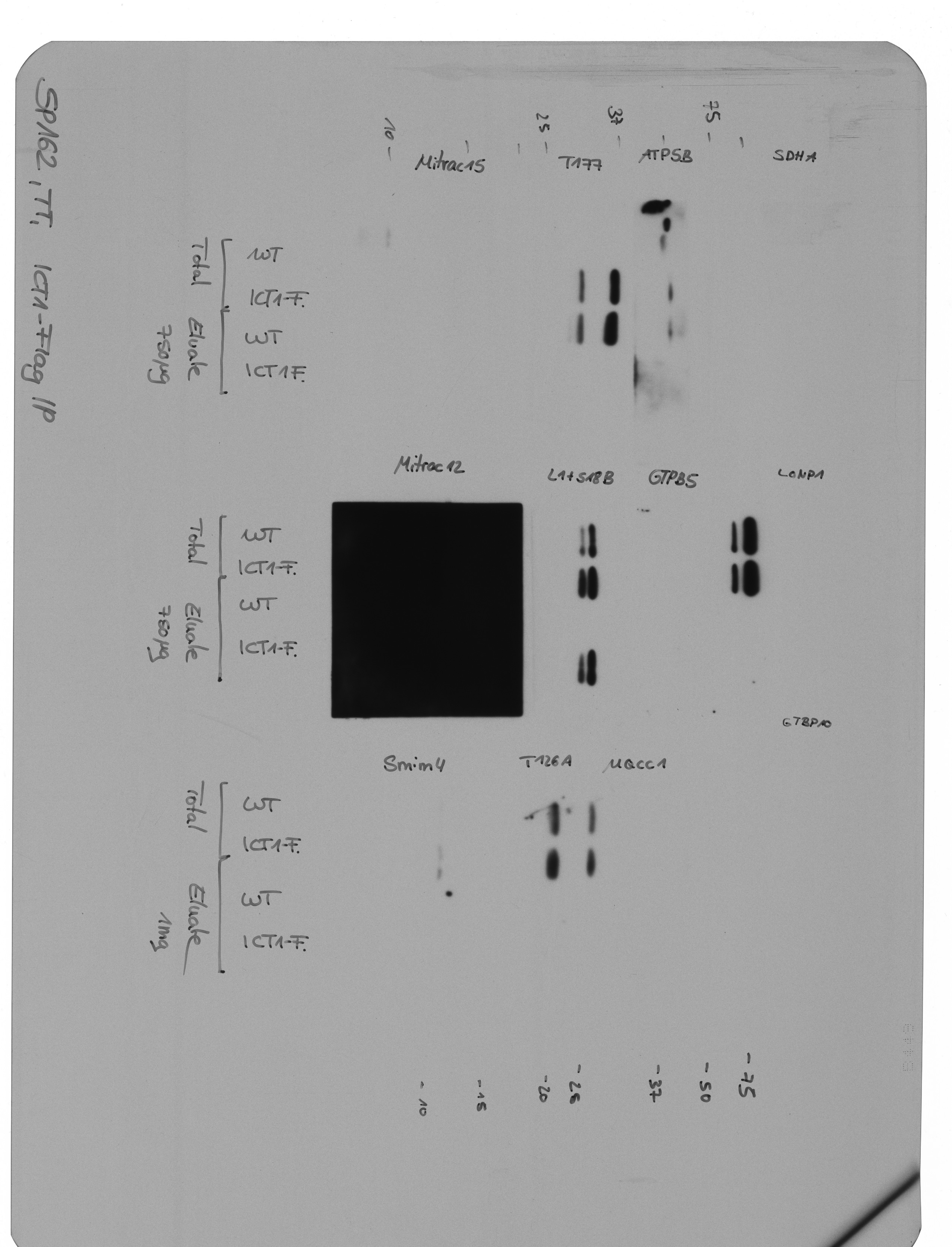

Supplement: Figure 1—source data 1. [file elife-68213-fig1-data1.zip › Figure_1_source_data/Figure_1_source_data_2_Figure_1D/Original_files/SP162 ICT1-IP005.jpg]

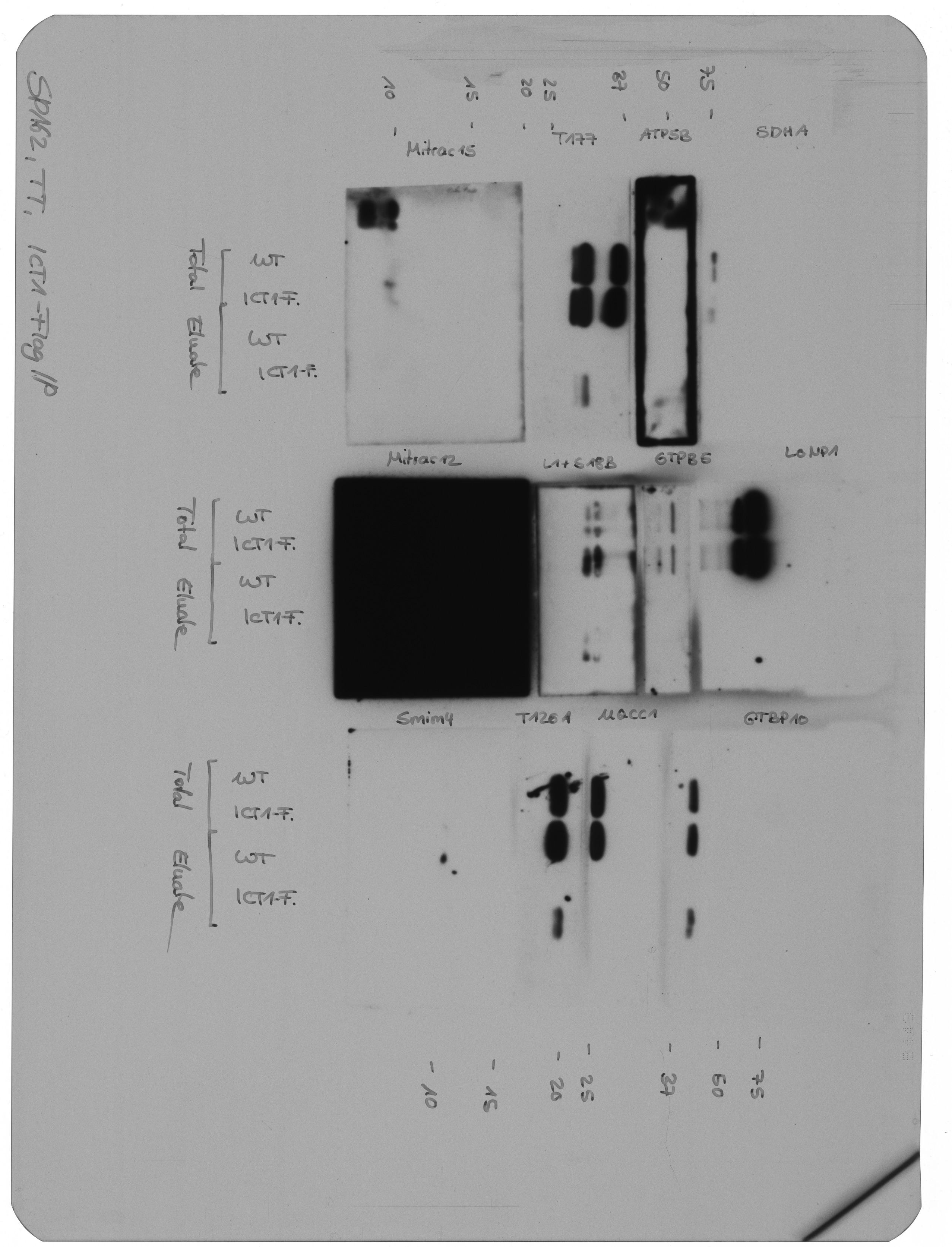

Supplement: Figure 1—source data 1. [file elife-68213-fig1-data1.zip › Figure_1_source_data/Figure_1_source_data_2_Figure_1D/Original_files/SP162 ICT1-IP002.jpg]

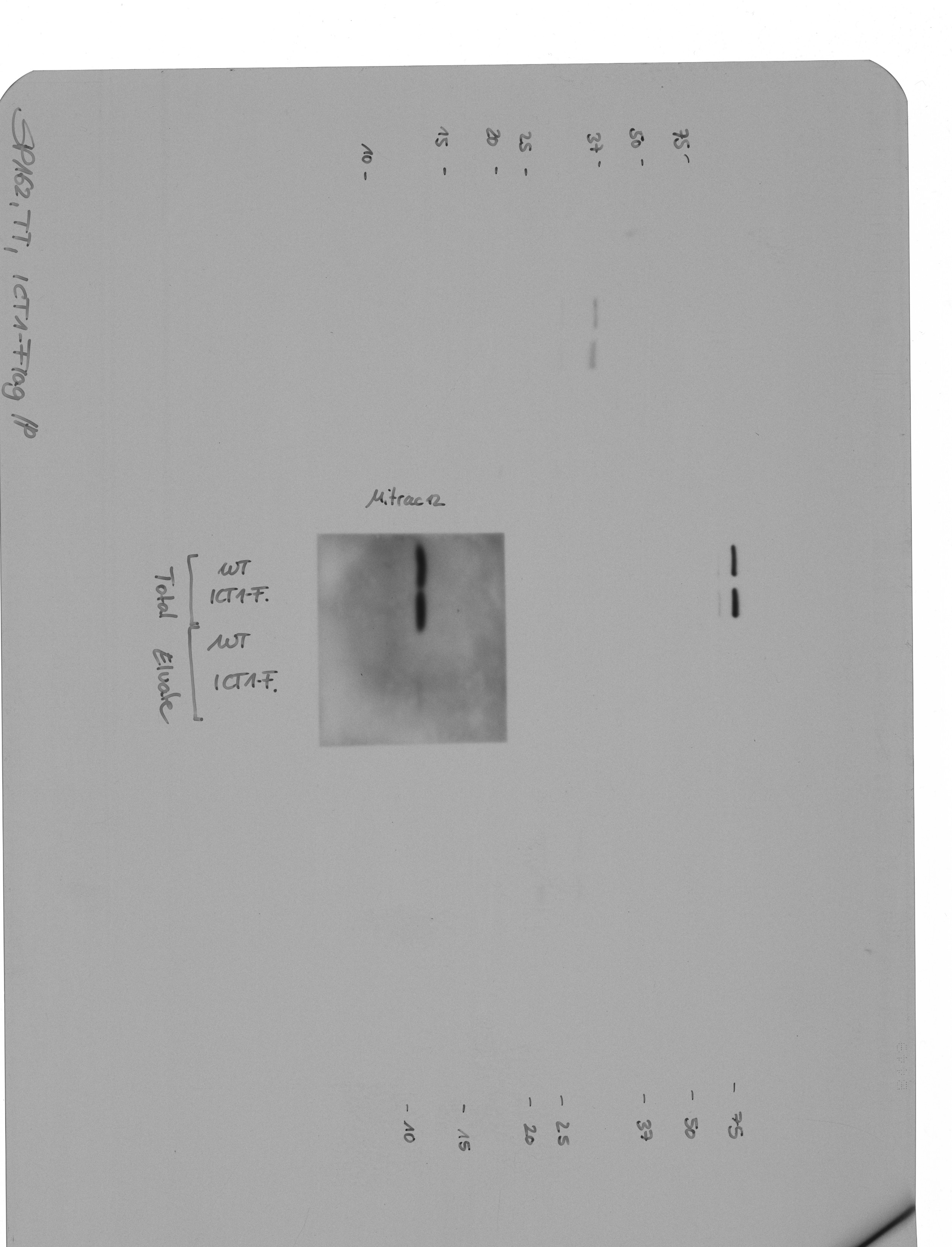

Supplement: Figure 1—source data 1. [file elife-68213-fig1-data1.zip › Figure_1_source_data/Figure_1_source_data_2_Figure_1D/Original_files/SP162 ICT1-IP004.jpg]

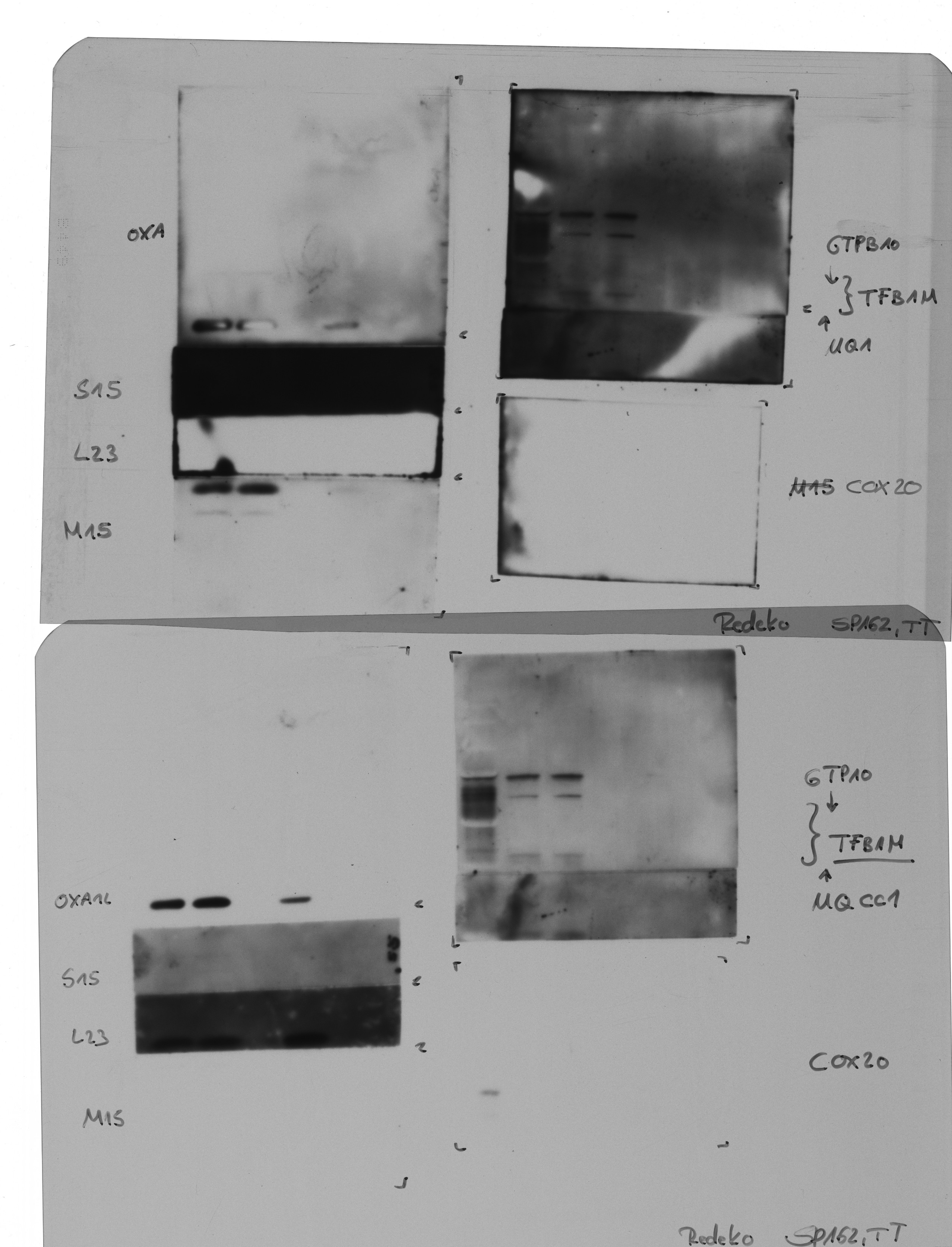

Supplement: Figure 1—source data 1. [file elife-68213-fig1-data1.zip › Figure_1_source_data/Figure_1_source_data_2_Figure_1D/Original_files/SP162 ICT1-IP001.jpg]

Figure 2 source data 2 related to Figure 2C

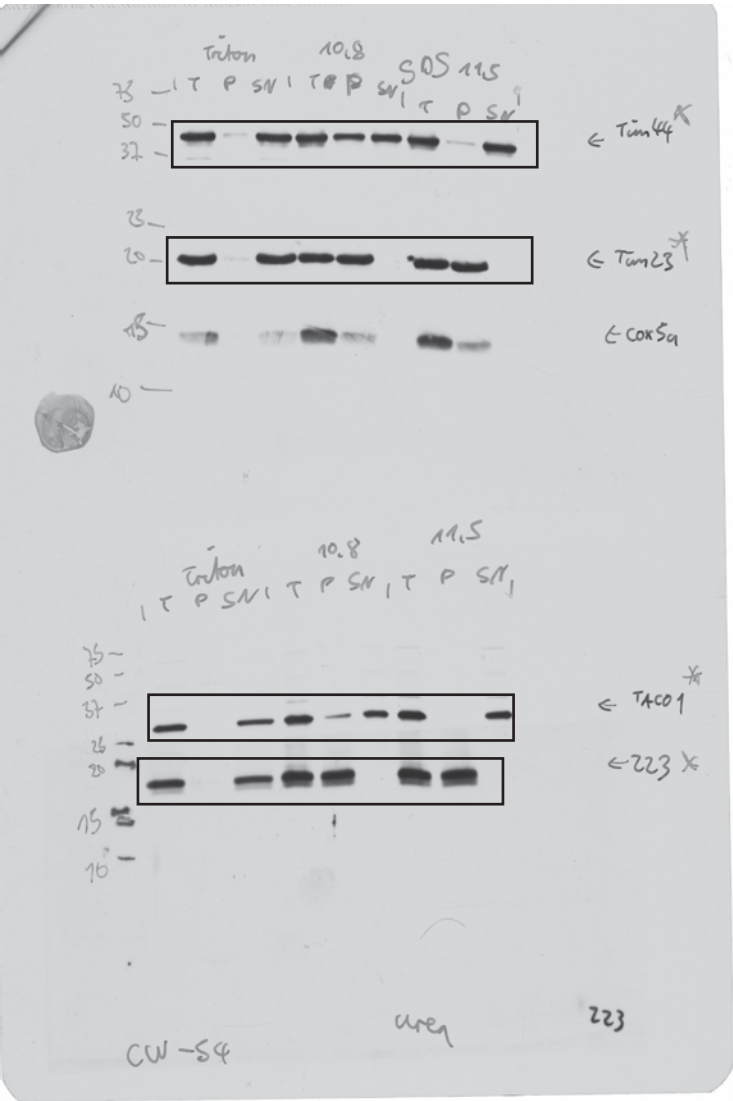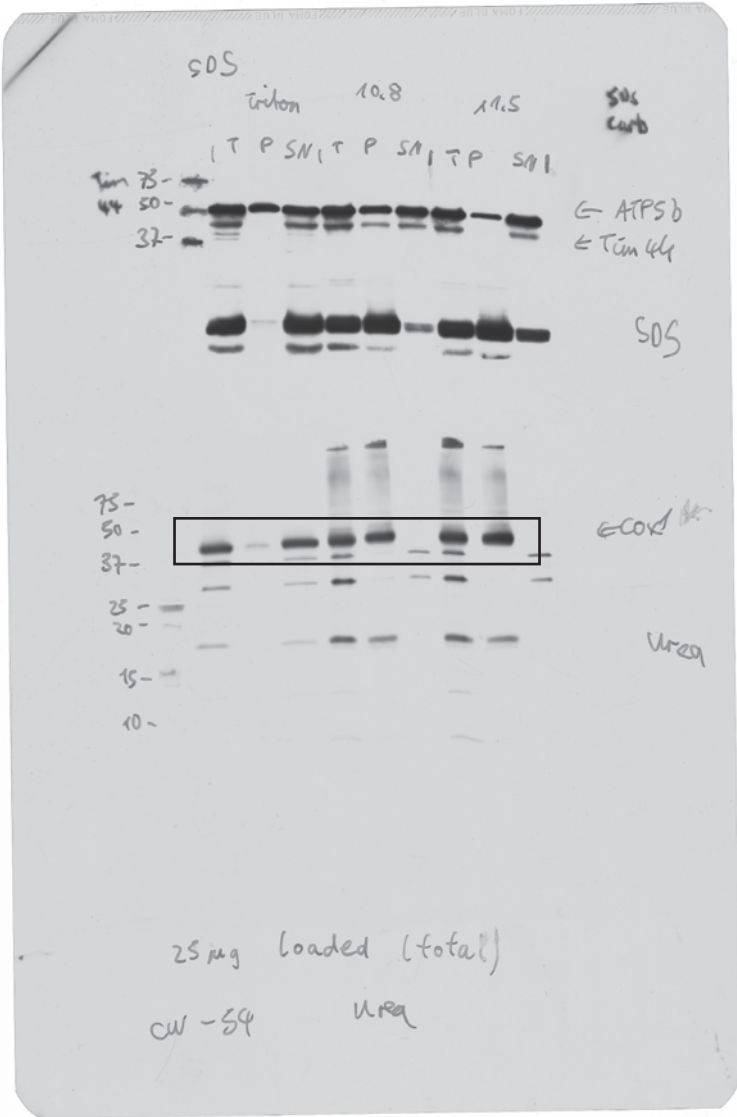

Supplement: Figure 2—source data 1. [file elife-68213-fig2-data1.zip › Figure_2_source_data/Figure_2_source_data_2_Figure_2C/Data_labelled/Figure_2_source_data_2_Figure_2C.pdf]

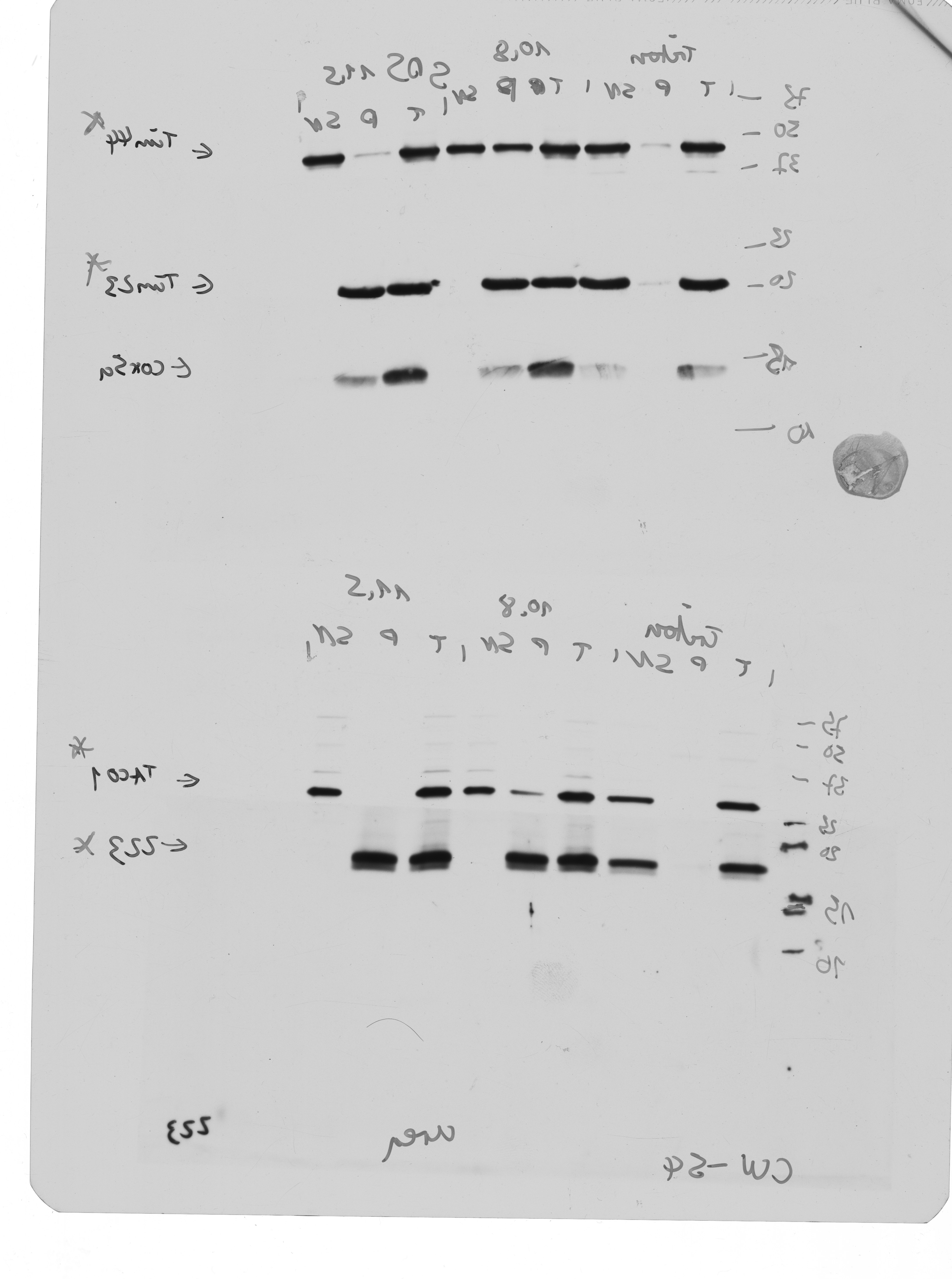

Supplement: Figure 2—source data 1. [file elife-68213-fig2-data1.zip › Figure_2_source_data/Figure_2_source_data_2_Figure_2C/Original_files/T44_T23_T223_TACO002.jpg]

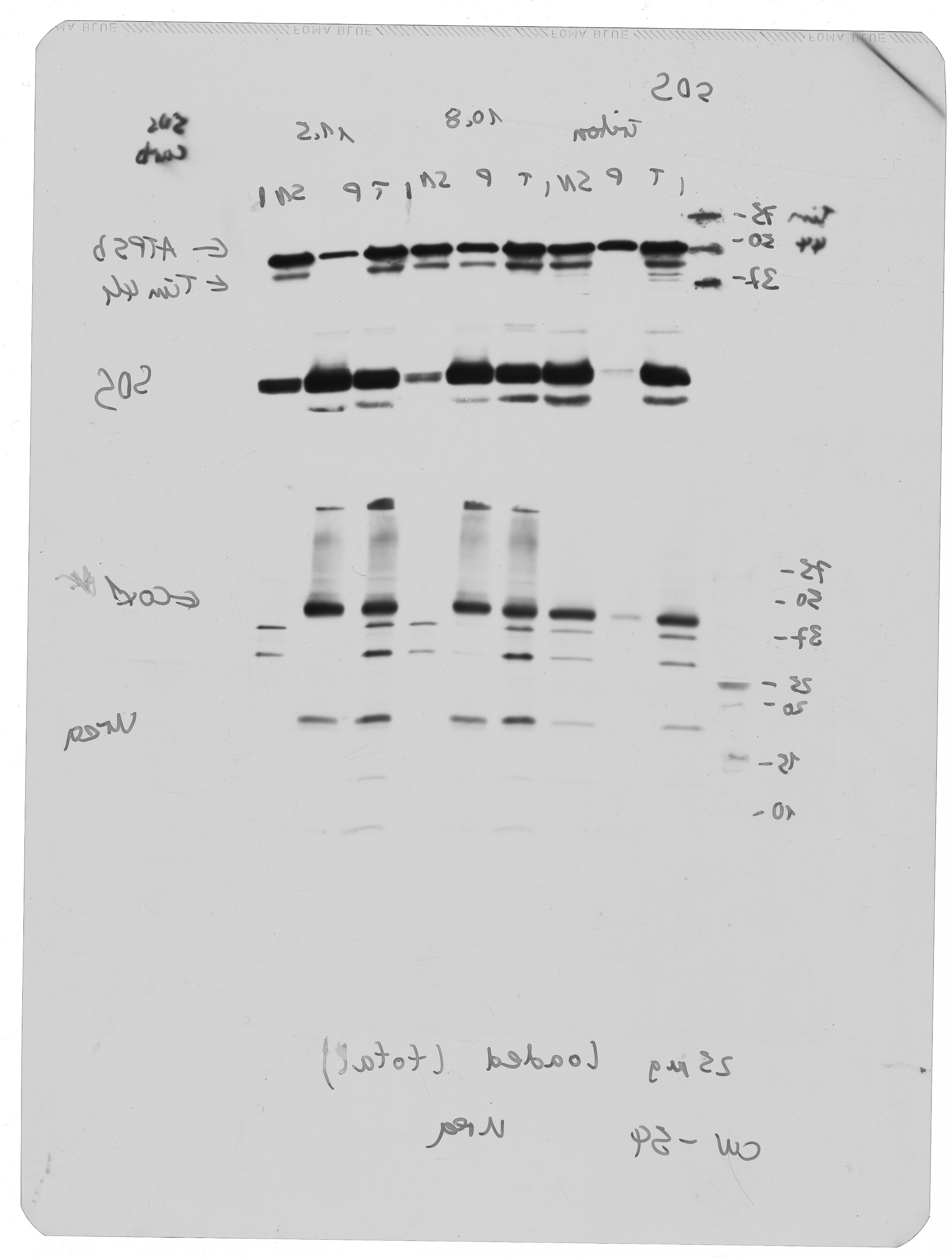

Supplement: Figure 2—source data 1. [file elife-68213-fig2-data1.zip › Figure_2_source_data/Figure_2_source_data_2_Figure_2C/Original_files/2019.jpg]

Figure 2 source data 1 related to Figure 2B

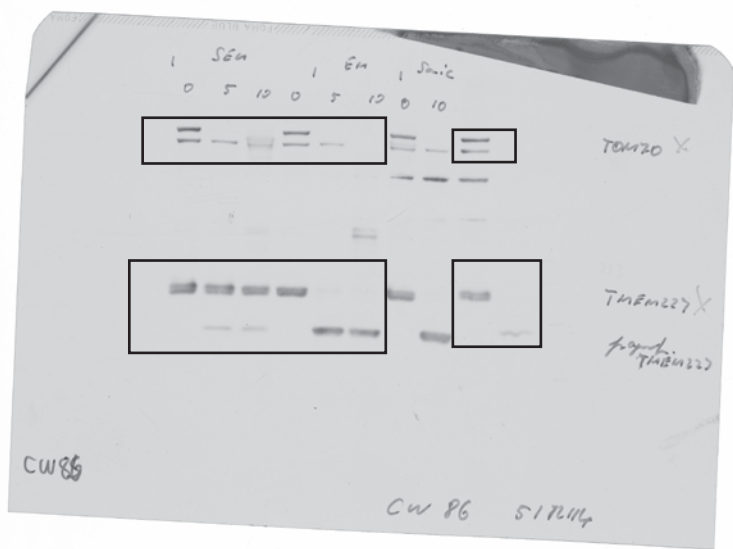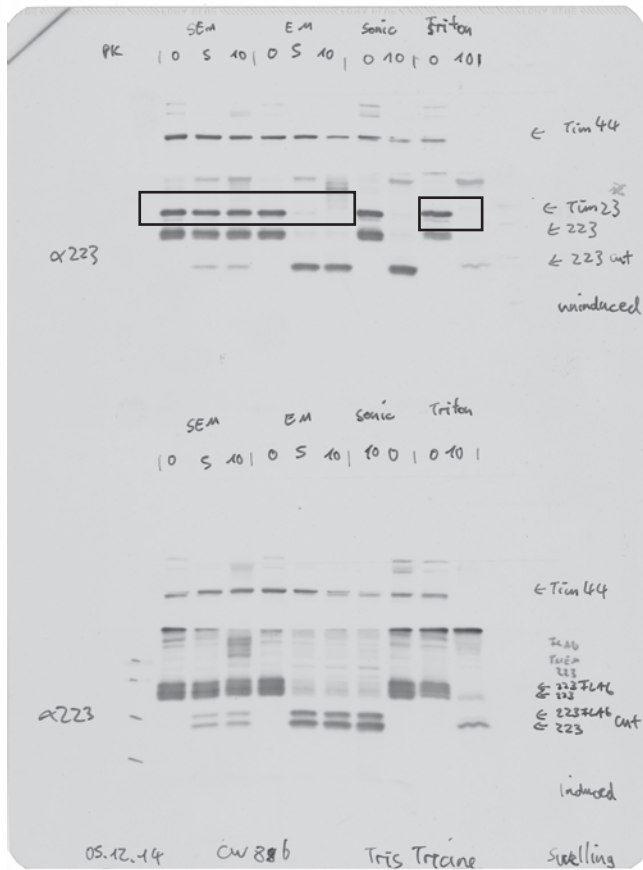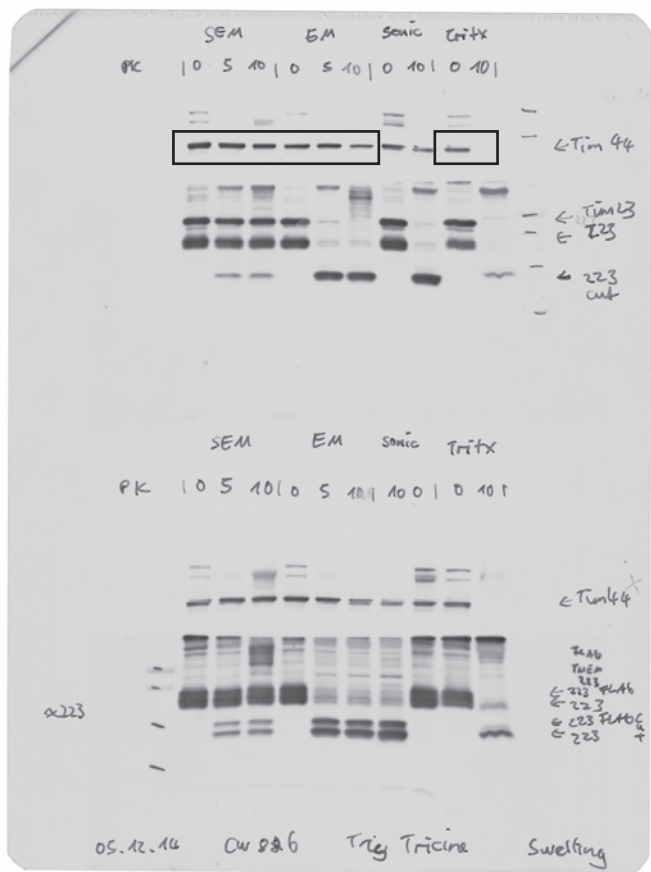

Supplement: Figure 2—source data 1. [file elife-68213-fig2-data1.zip › Figure_2_source_data/Figure_2_source_data_1_Figure_2B/Data_labelled/Figure_2_source_data_1_Figure_2B.pdf]

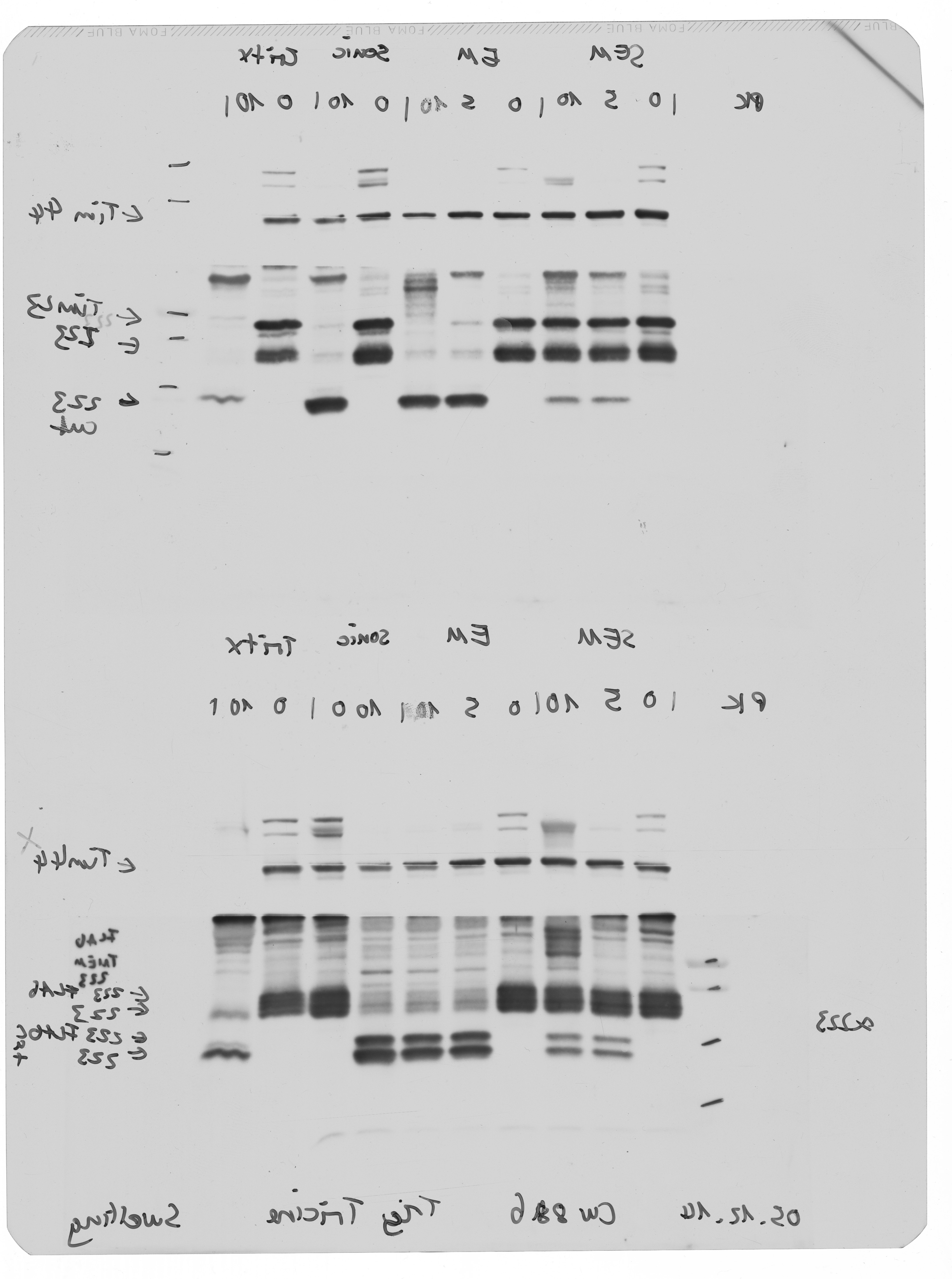

Supplement: Figure 2—source data 1. [file elife-68213-fig2-data1.zip › Figure_2_source_data/Figure_2_source_data_1_Figure_2B/Original_files/T44003.jpg]

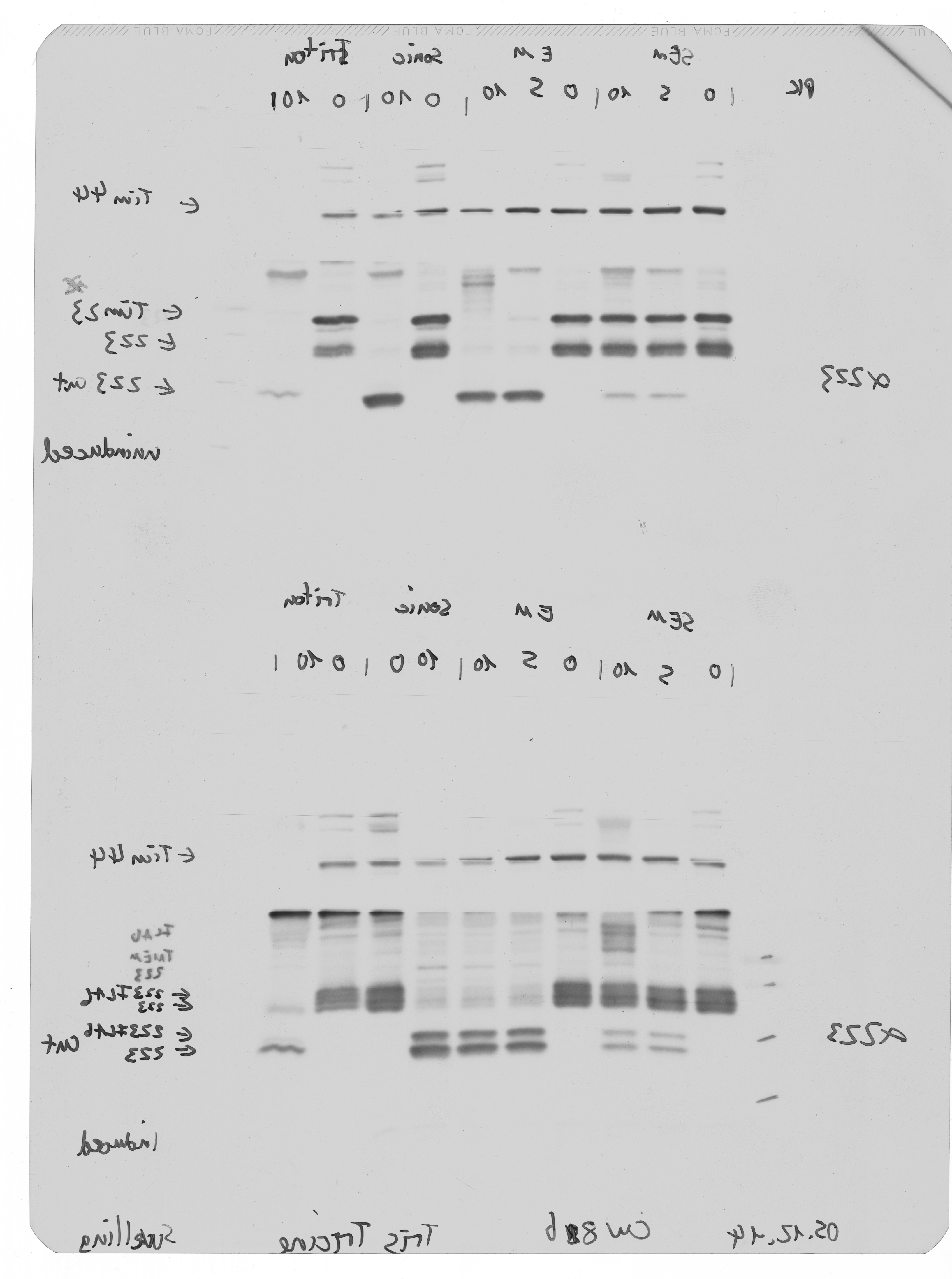

Supplement: Figure 2—source data 1. [file elife-68213-fig2-data1.zip › Figure_2_source_data/Figure_2_source_data_1_Figure_2B/Original_files/T23004.jpg]

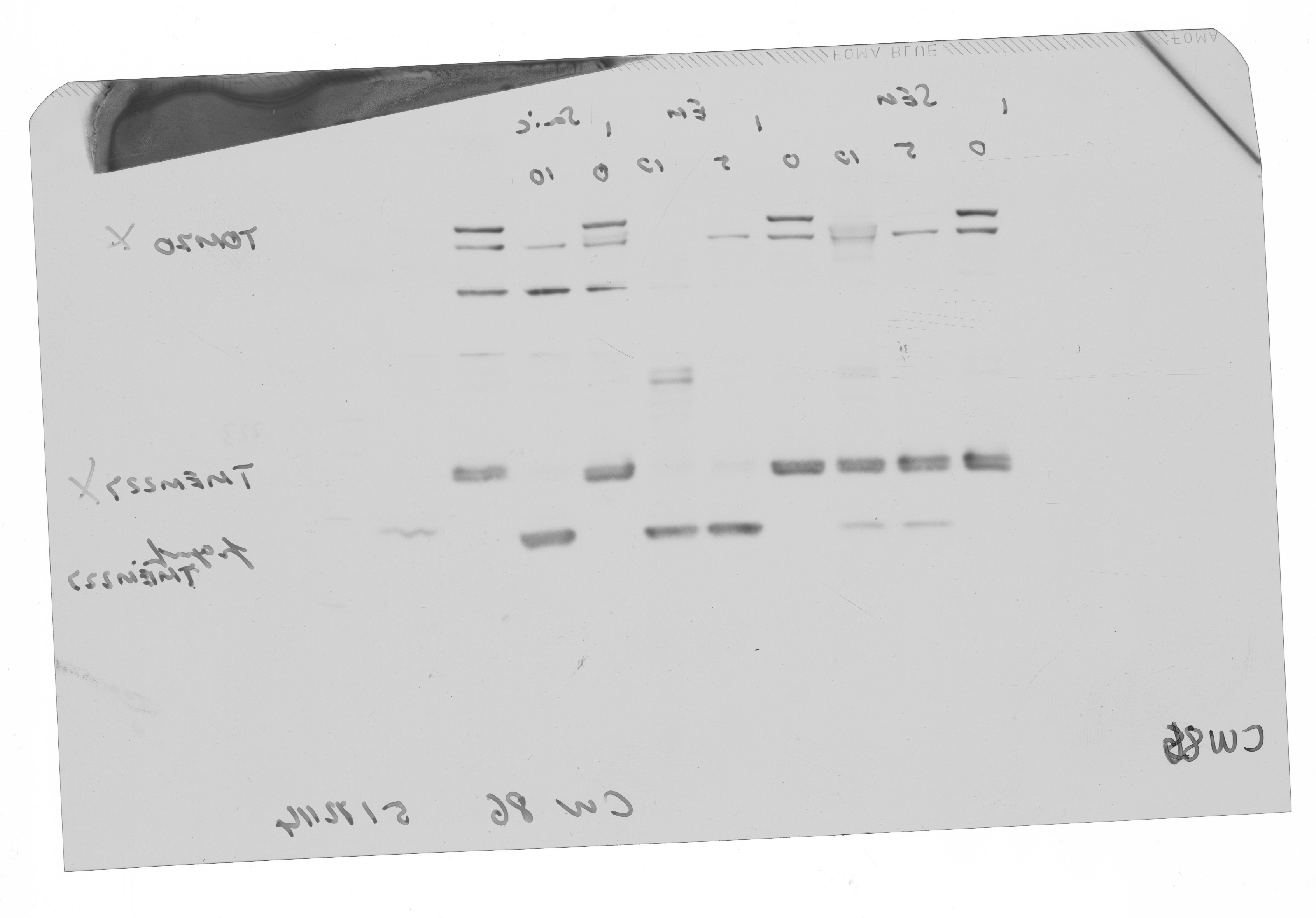

Supplement: Figure 2—source data 1. [file elife-68213-fig2-data1.zip › Figure_2_source_data/Figure_2_source_data_1_Figure_2B/Original_files/T70_T223005.jpg]

Figure 2 source data 3 related to Figure 2D

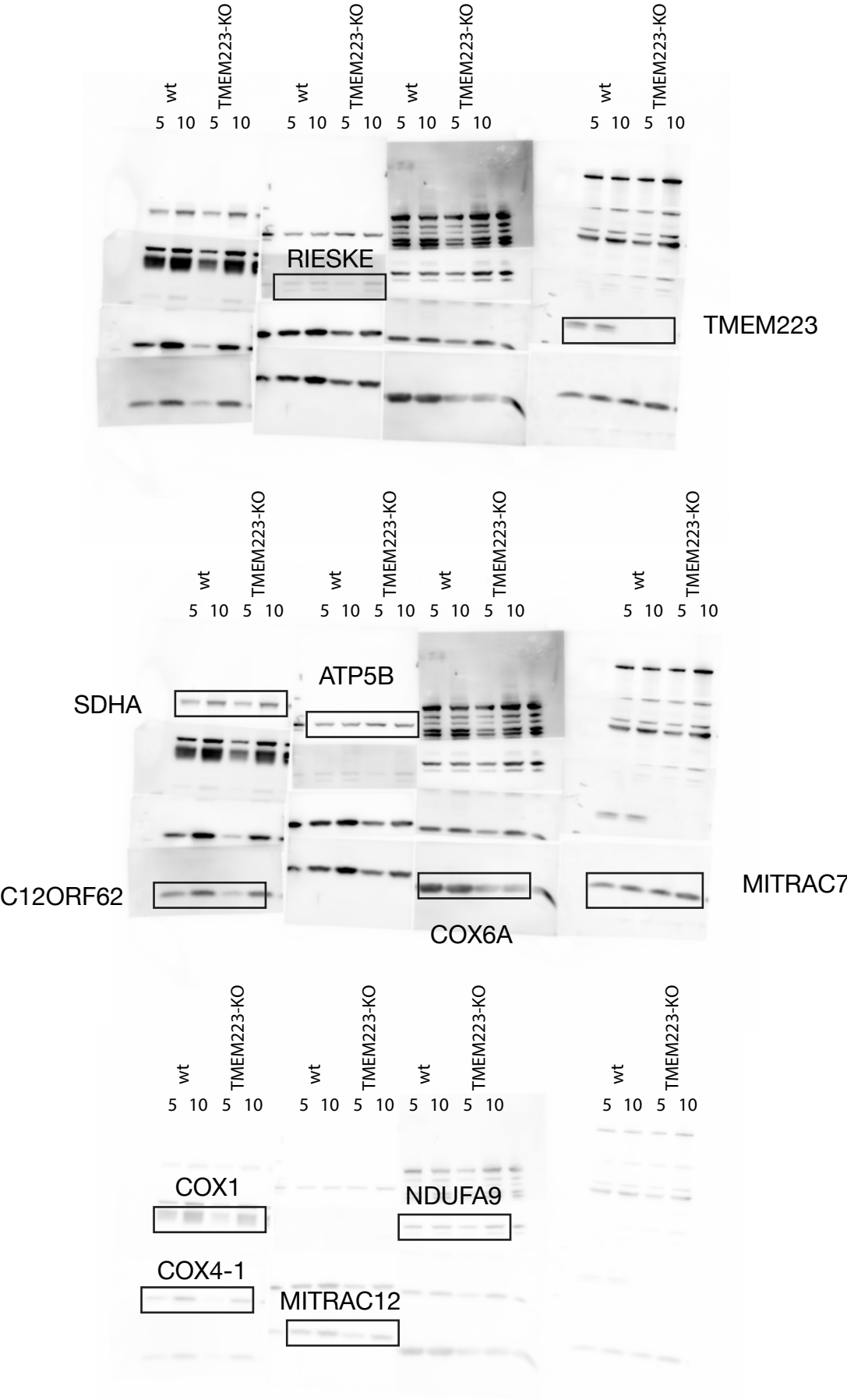

Supplement: Figure 2—source data 1. [file elife-68213-fig2-data1.zip › Figure_2_source_data/Figure_2_source_data_3_Figure_2D/Data_labelled/Figure_2_source_data_3_Figure_2D.pdf]

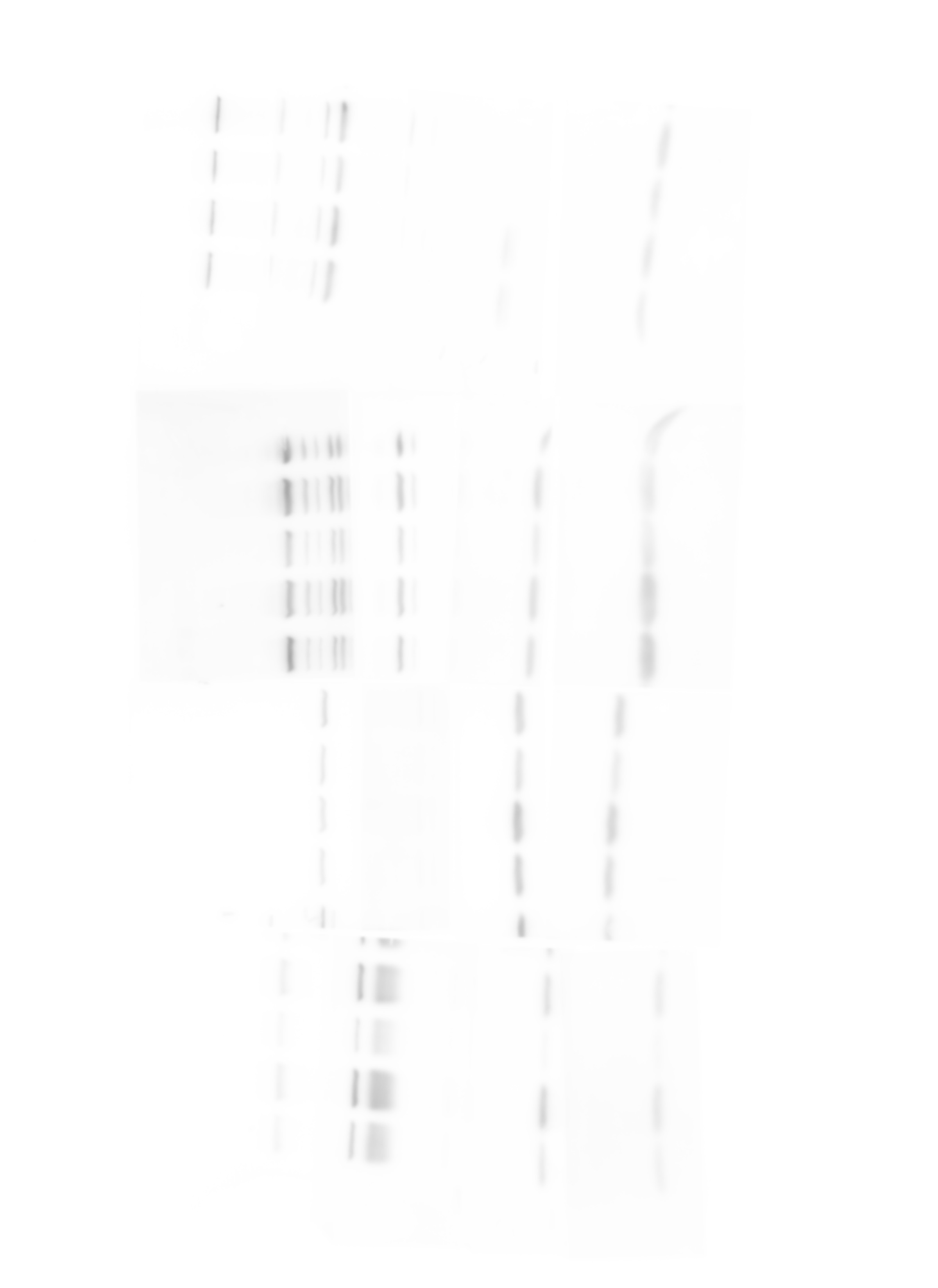

Supplement: Figure 2—source data 1. [file elife-68213-fig2-data1.zip › Figure_2_source_data/Figure_2_source_data_3_Figure_2D/Original_files/Steady_state_TMEM223_KO 20200526_112946-01_Ch_Chemi.jpg]

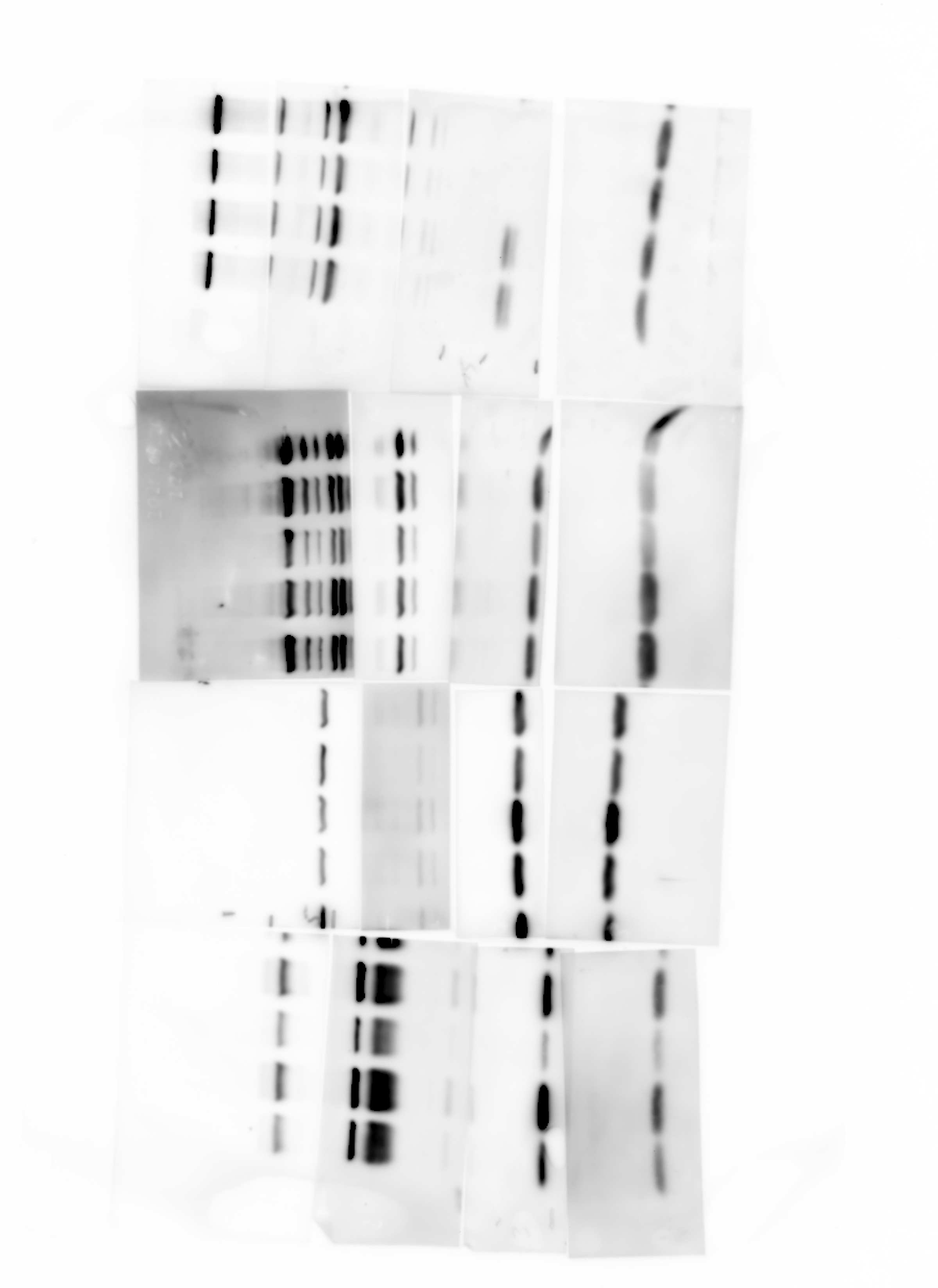

Supplement: Figure 2—source data 1. [file elife-68213-fig2-data1.zip › Figure_2_source_data/Figure_2_source_data_3_Figure_2D/Original_files/Steady_state_TMEM223_KO 20200526_112946-17_Ch_Chemi.jpg]

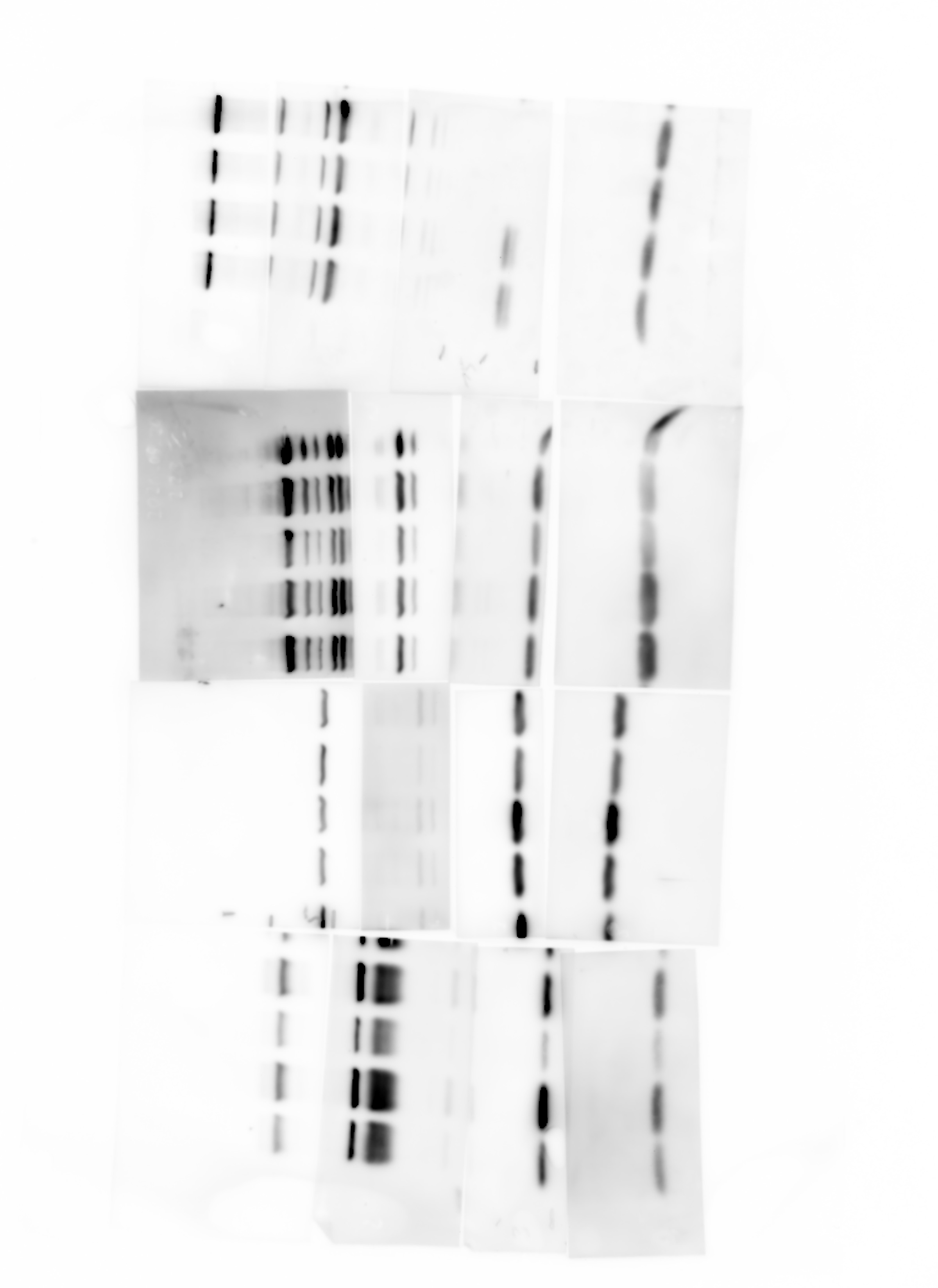

Supplement: Figure 2—source data 1. [file elife-68213-fig2-data1.zip › Figure_2_source_data/Figure_2_source_data_3_Figure_2D/Original_files/Steady_state_TMEM223_KO 20200526_112946-12_Ch_Chemi.jpg]

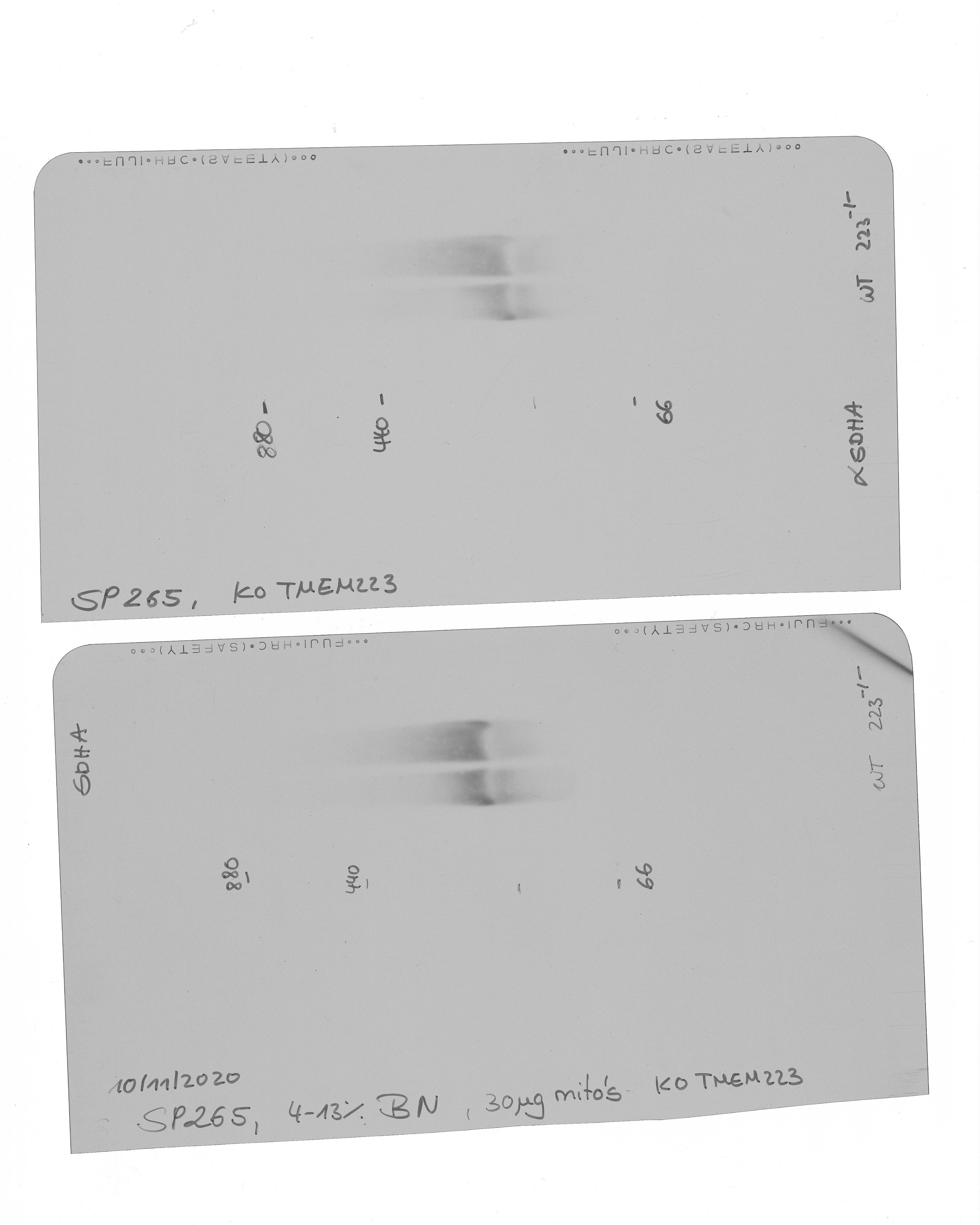

Supplement: Figure 2—source data 1. [file elife-68213-fig2-data1.zip › Figure_2_source_data/Figure_2_source_data_4_Figure_2E/Original_files/SP265 BN KO 223002.jpg]

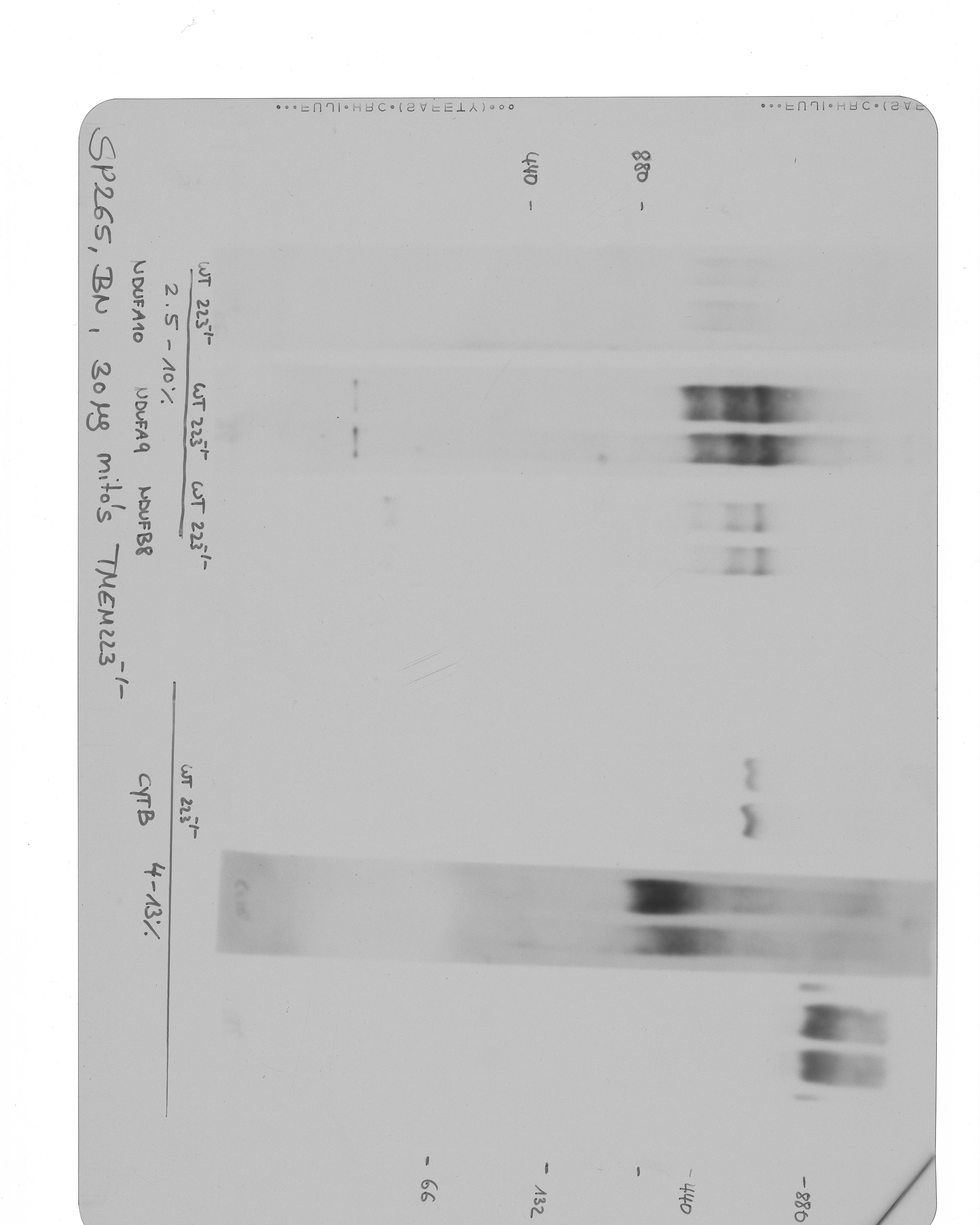

Supplement: Figure 2—source data 1. [file elife-68213-fig2-data1.zip › Figure_2_source_data/Figure_2_source_data_4_Figure_2E/Original_files/SP265 BN KO 223001.jpg]

Figure\_2\_source\_data\_4\_Figure\_2E

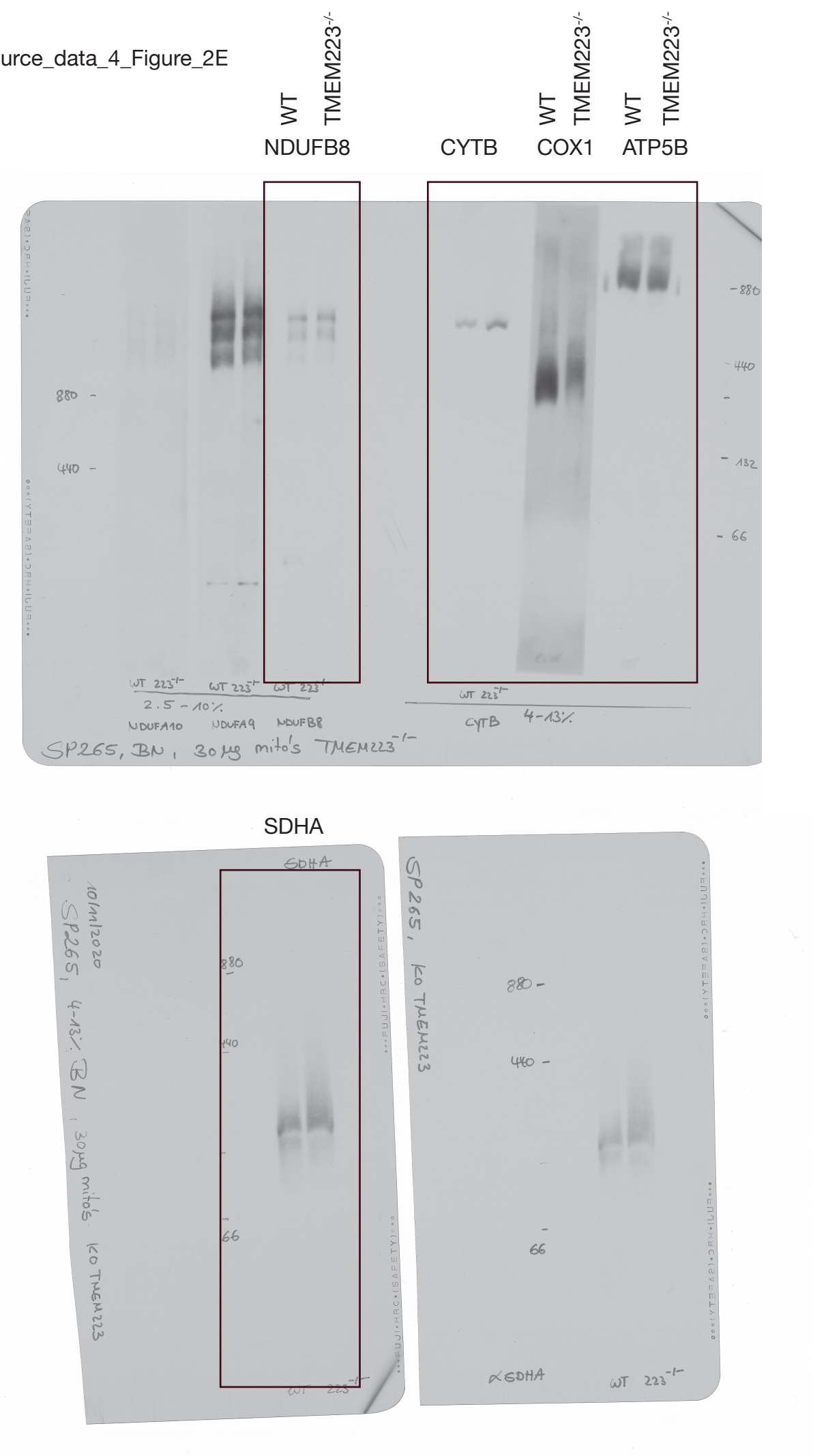

Supplement: Figure 2—source data 1. [file elife-68213-fig2-data1.zip › Figure_2_source_data/Figure_2_source_data_4_Figure_2E/Data_labelled/Figure_2_source_data_4_Figure_2E.pdf]

Figure 2 supplement 1 source data 2 related to Figure 1 supplement 1 B

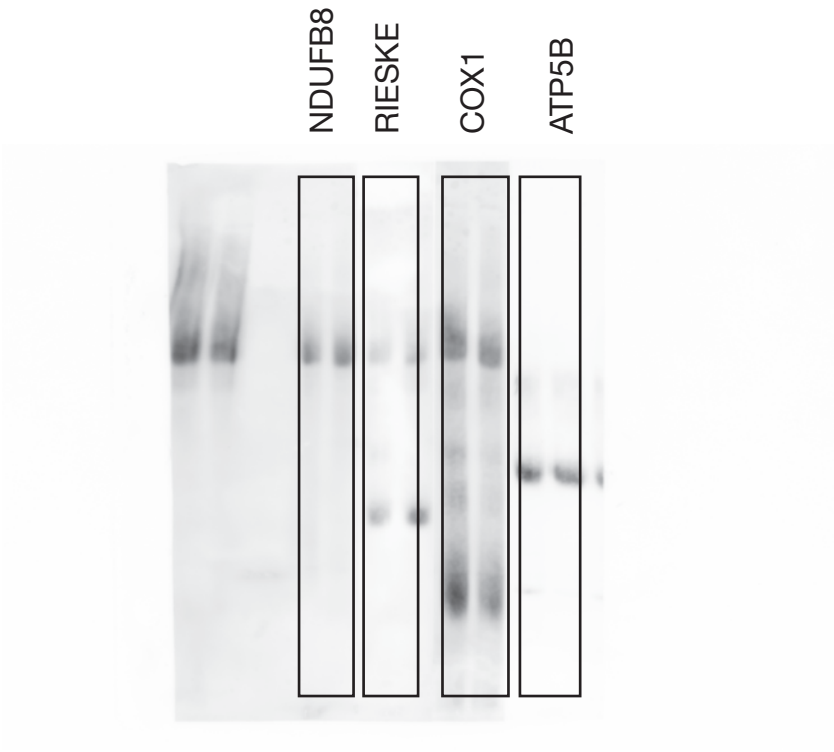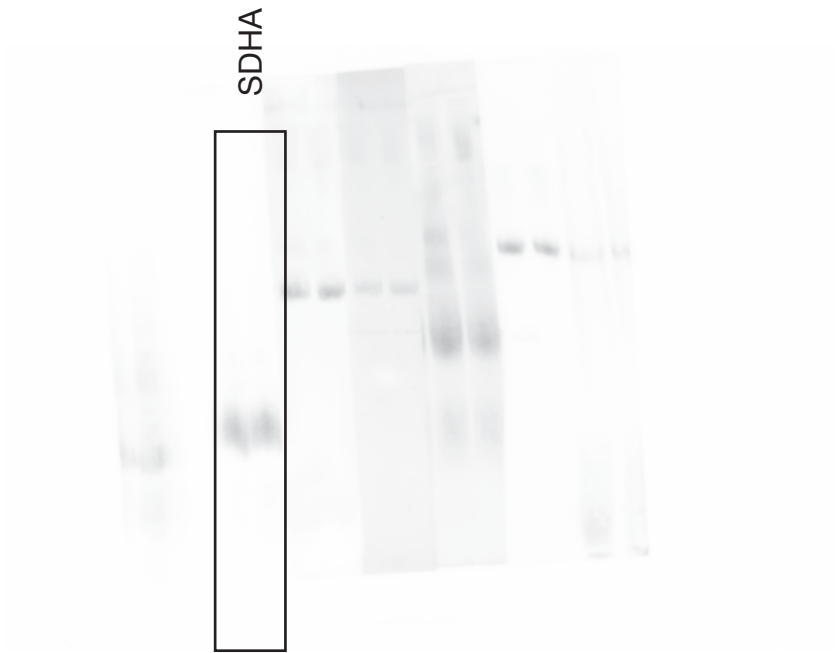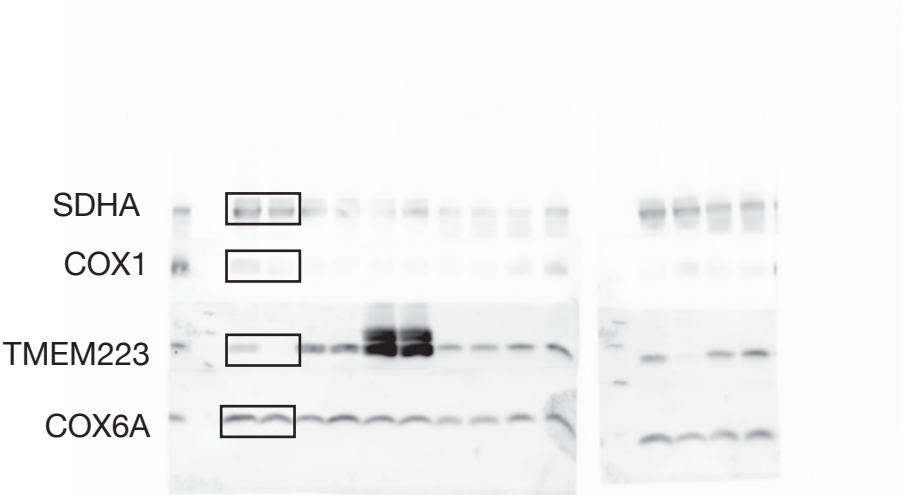

Supplement: Figure 2—figure supplement 1—source data 1. [file elife-68213-fig2-figsupp1-data1.zip › Figure_2_Supplement_1_source_data/Figure_2_supplement_1_source_data_2_Figure_2_supplement_1B/Data_labelled/Figure2_supplemente_1_source_data_2_related_Figure_2_supplement_1B.pdf]

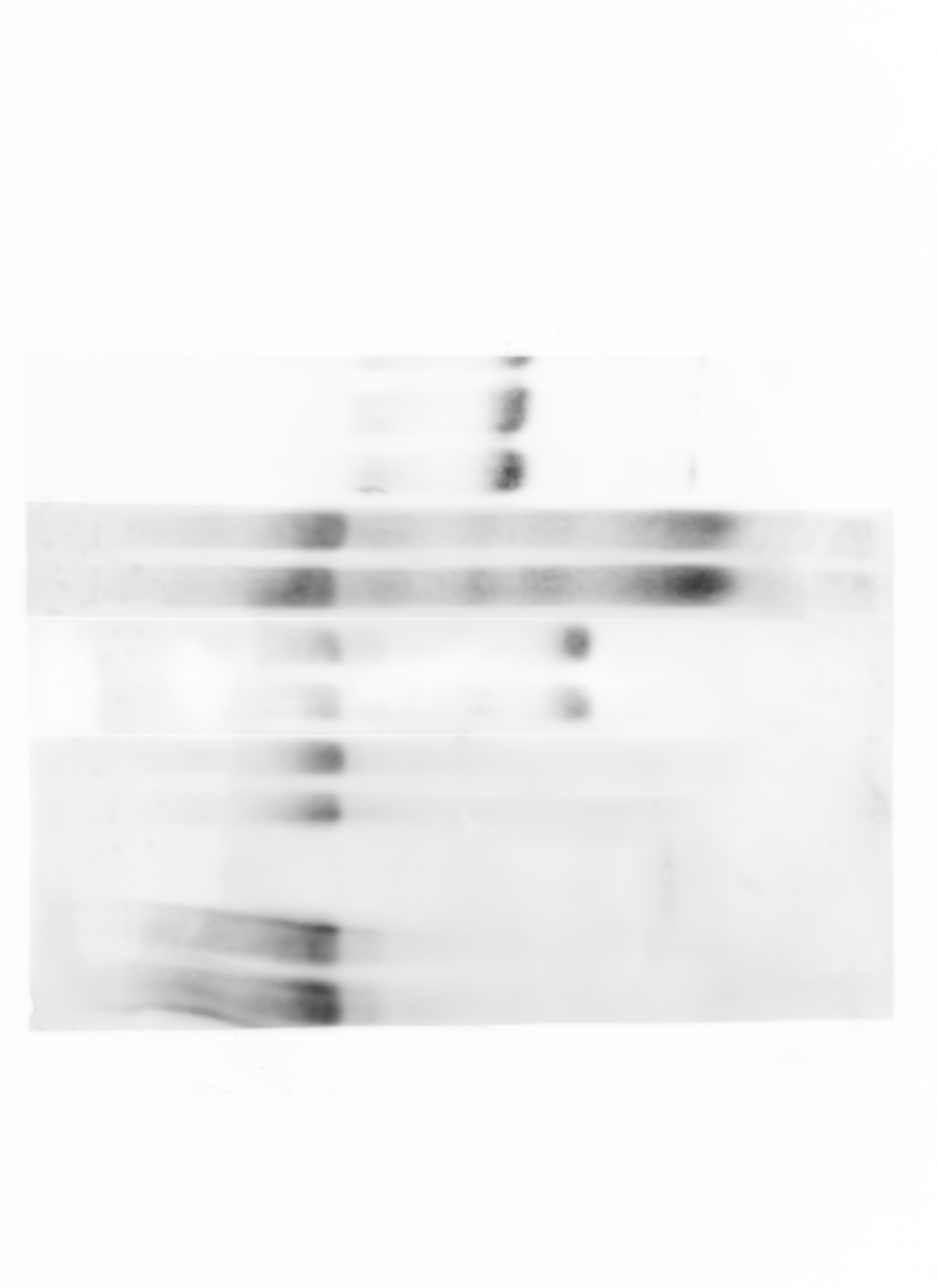

Supplement: Figure 2—figure supplement 1—source data 1. [file elife-68213-fig2-figsupp1-data1.zip › Figure_2_Supplement_1_source_data/Figure_2_supplement_1_source_data_2_Figure_2_supplement_1B/Original_data/1st LP 20210624_130858-13_Ch_Chemi.jpg]

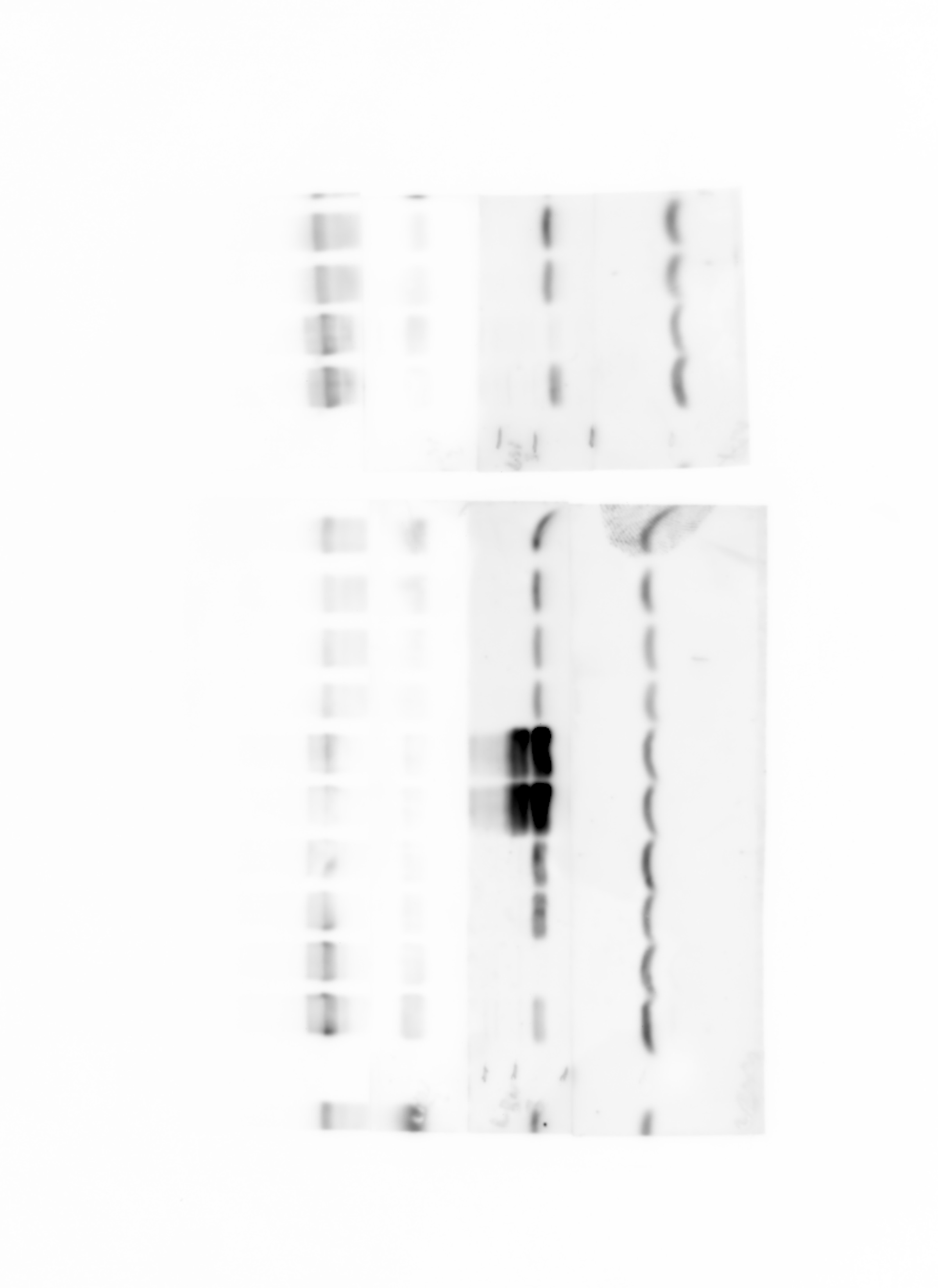

Supplement: Figure 2—figure supplement 1—source data 1. [file elife-68213-fig2-figsupp1-data1.zip › Figure_2_Supplement_1_source_data/Figure_2_supplement_1_source_data_2_Figure_2_supplement_1B/Original_data/1st load 20210713_143815-20_Ch_Chemi.jpg]

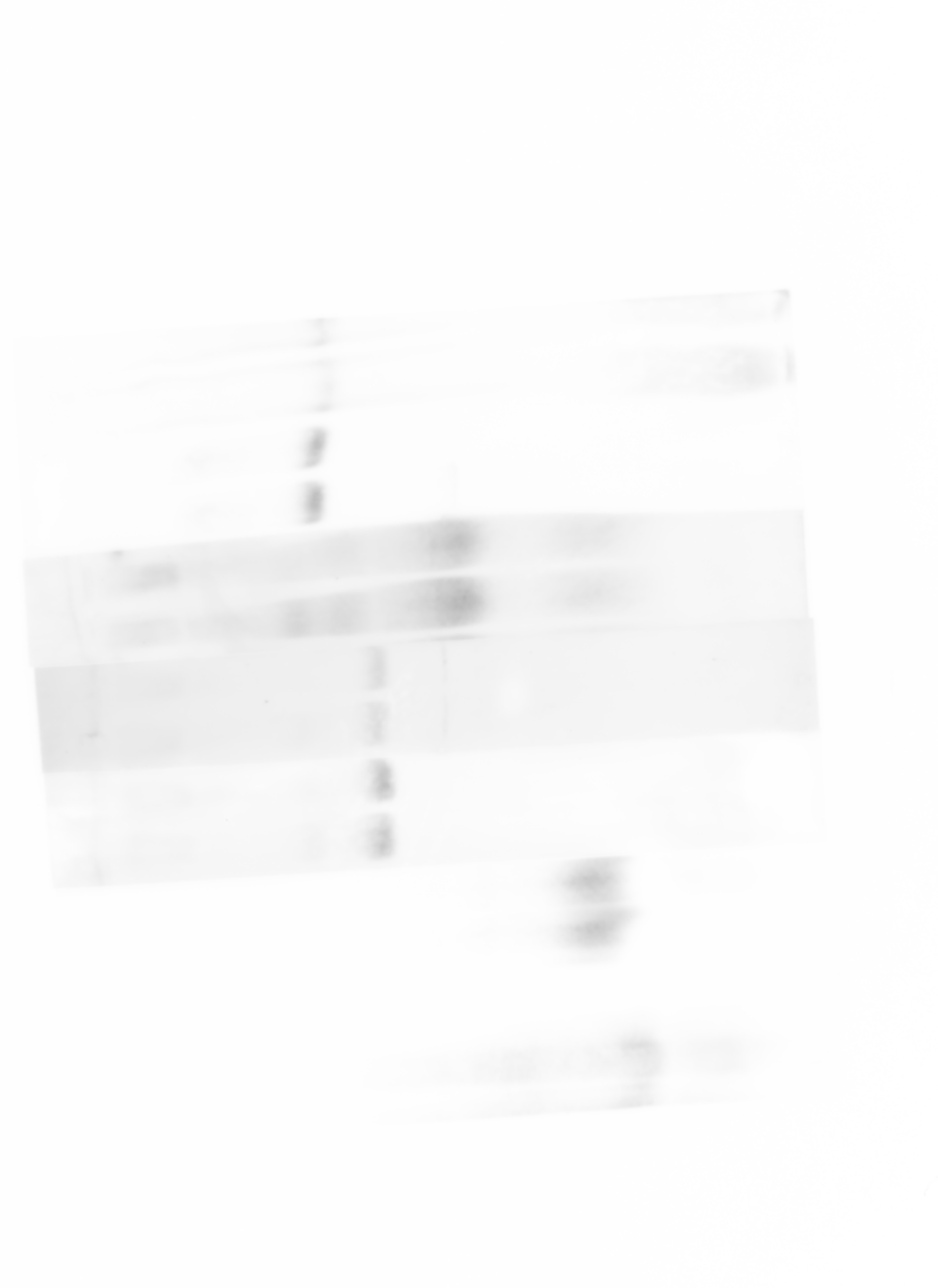

Supplement: Figure 2—figure supplement 1—source data 1. [file elife-68213-fig2-figsupp1-data1.zip › Figure_2_Supplement_1_source_data/Figure_2_supplement_1_source_data_2_Figure_2_supplement_1B/Original_data/1st 14% 20210624_131810-08_Ch_Chemi.jpg]

Figure 2 supplement 1 source data 3 related to Figure 1 supplement 1C

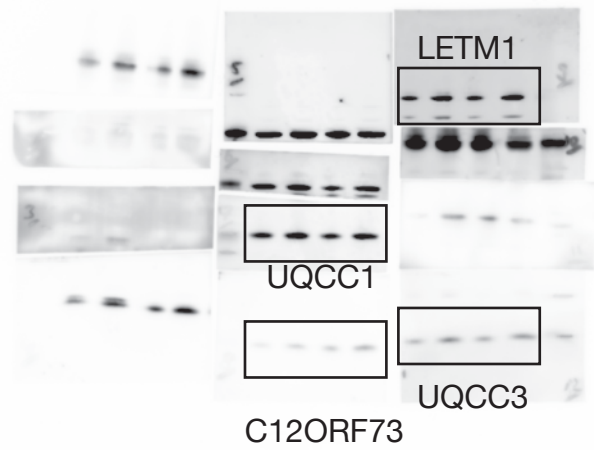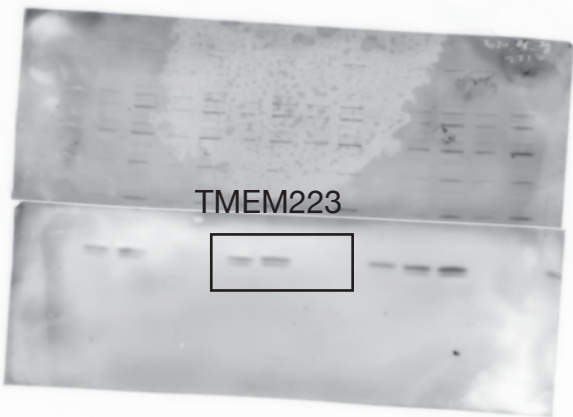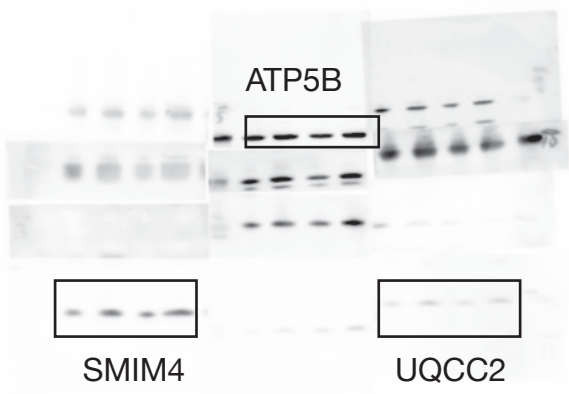

Supplement: Figure 2—figure supplement 1—source data 1. [file elife-68213-fig2-figsupp1-data1.zip › Figure_2_Supplement_1_source_data/Figure_2_supplement_1_source_data_3_Figure_2_supplement_1C/Data_labelled/Figure2_supplemente_1_source_data_3_related_Figure_2_supplement_1C.pdf]

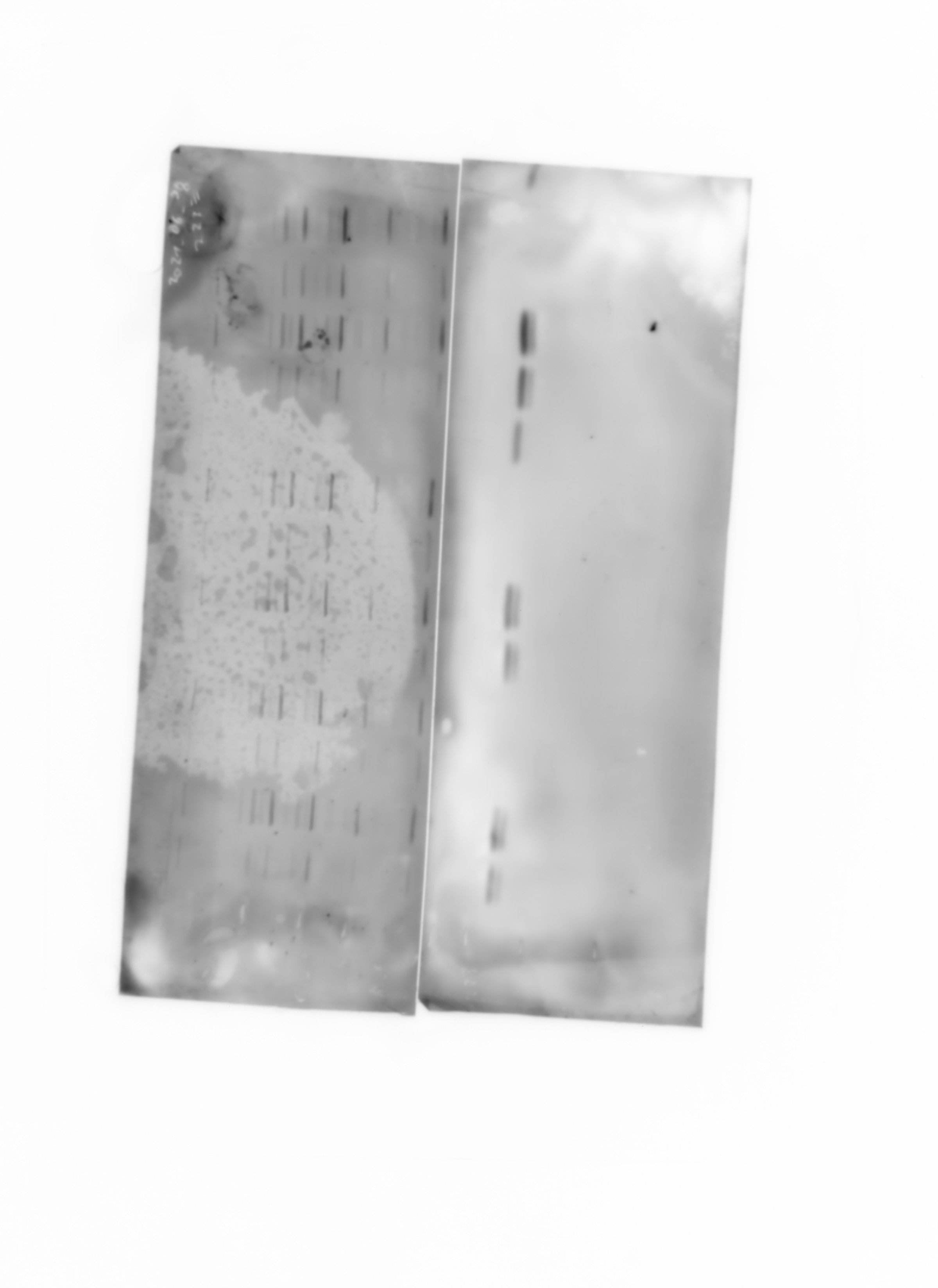

Supplement: Figure 2—figure supplement 1—source data 1. [file elife-68213-fig2-figsupp1-data1.zip › Figure_2_Supplement_1_source_data/Figure_2_supplement_1_source_data_3_Figure_2_supplement_1C/Original_data/redeco 20210609_162246-20_Ch_Chemi.jpg]

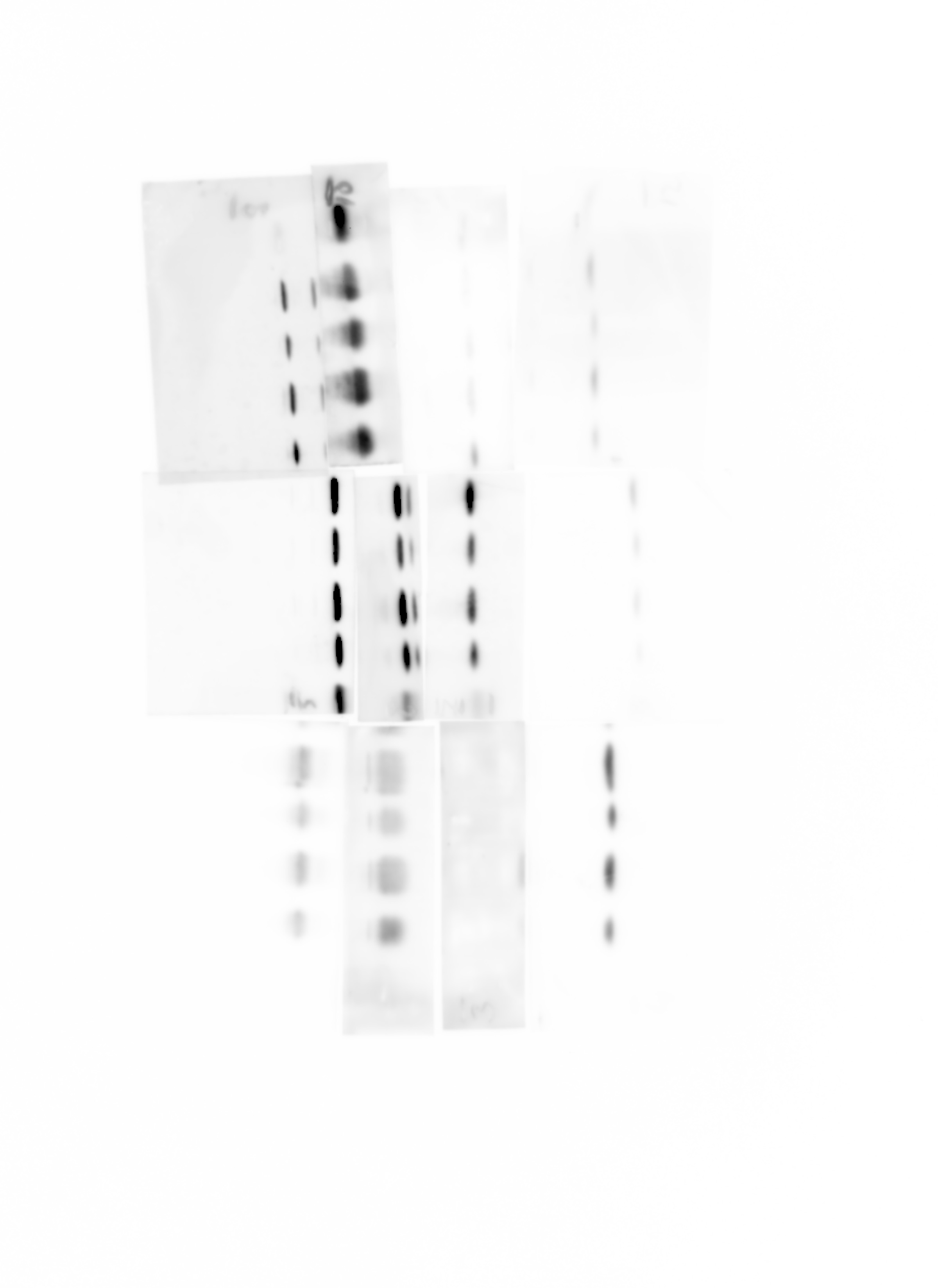

Supplement: Figure 2—figure supplement 1—source data 1. [file elife-68213-fig2-figsupp1-data1.zip › Figure_2_Supplement_1_source_data/Figure_2_supplement_1_source_data_3_Figure_2_supplement_1C/Original_data/1st expo 20210603_183545-05_Ch_Chemi.jpg]

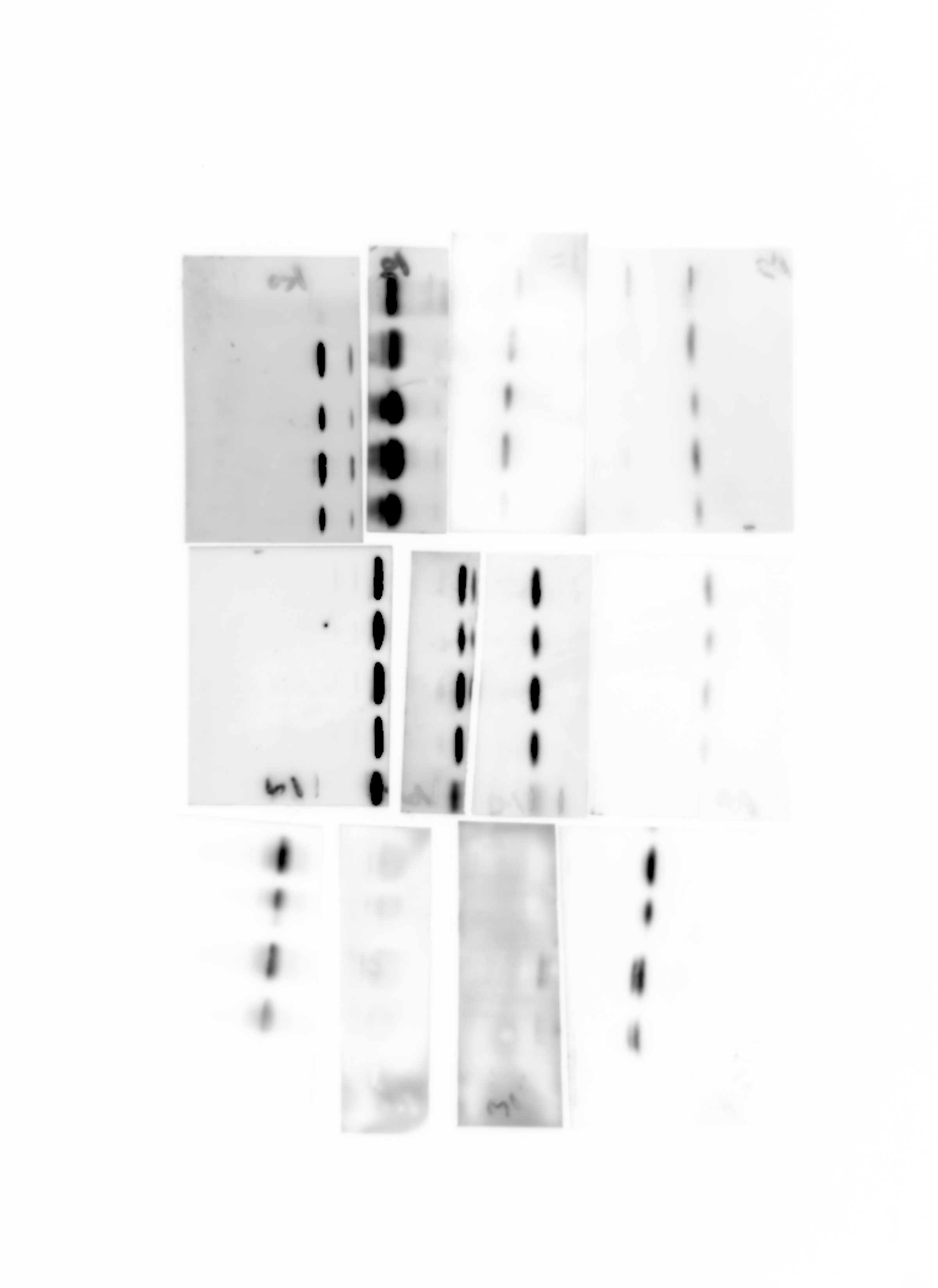

Supplement: Figure 2—figure supplement 1—source data 1. [file elife-68213-fig2-figsupp1-data1.zip › Figure_2_Supplement_1_source_data/Figure_2_supplement_1_source_data_3_Figure_2_supplement_1C/Original_data/1st expo 20210603_182700-10_Ch_Chemi.jpg]

Figure 2 supplement 1 source data 1 related to Figure 1 supplement 1 A

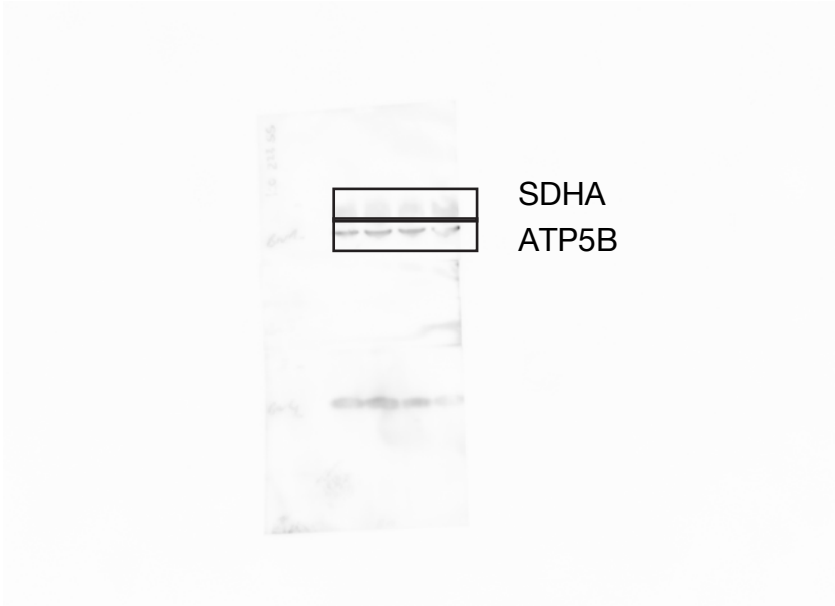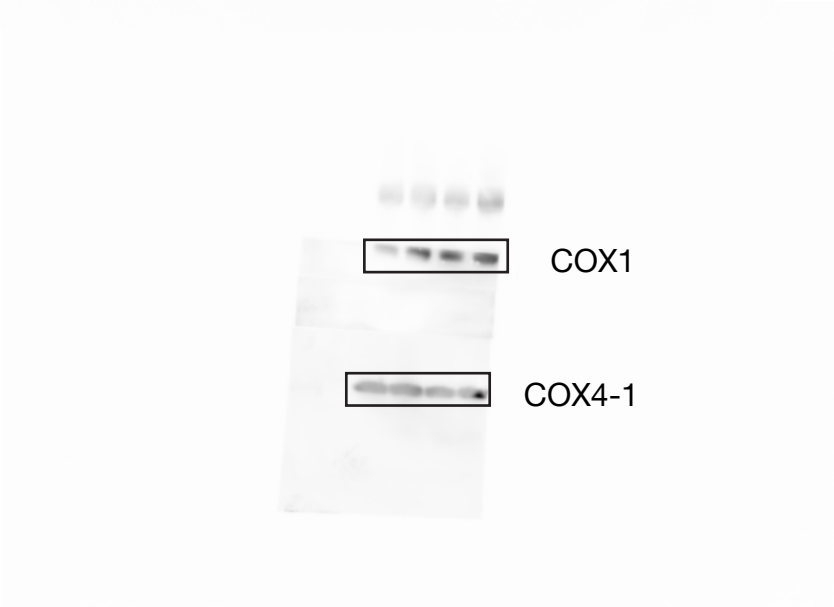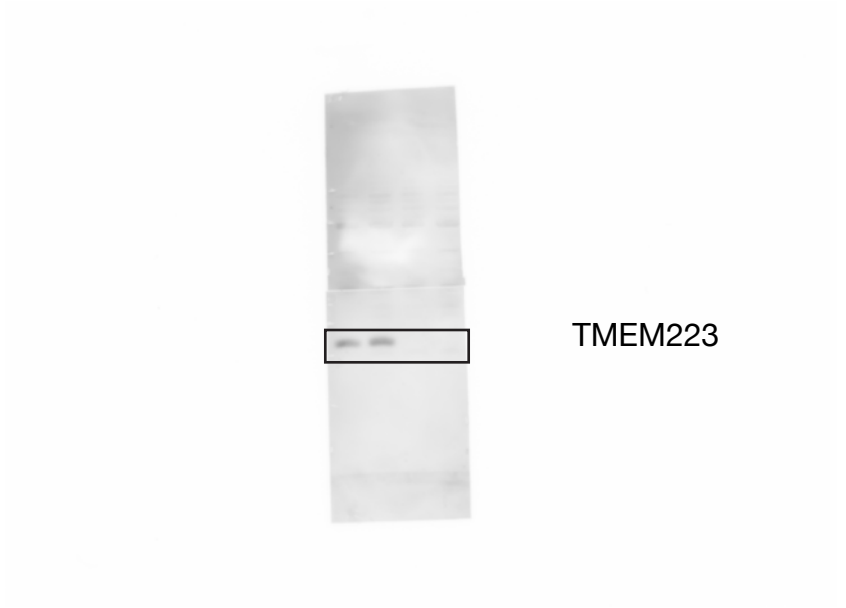

Supplement: Figure 2—figure supplement 1—source data 1. [file elife-68213-fig2-figsupp1-data1.zip › Figure_2_Supplement_1_source_data/Figure_2_supplement_1_Source_data_1_Figure_2_supplement_1A/Data_labelled/Figure_2_supplement_1_source_data_1_Figure_2_supplement_1A.pdf]

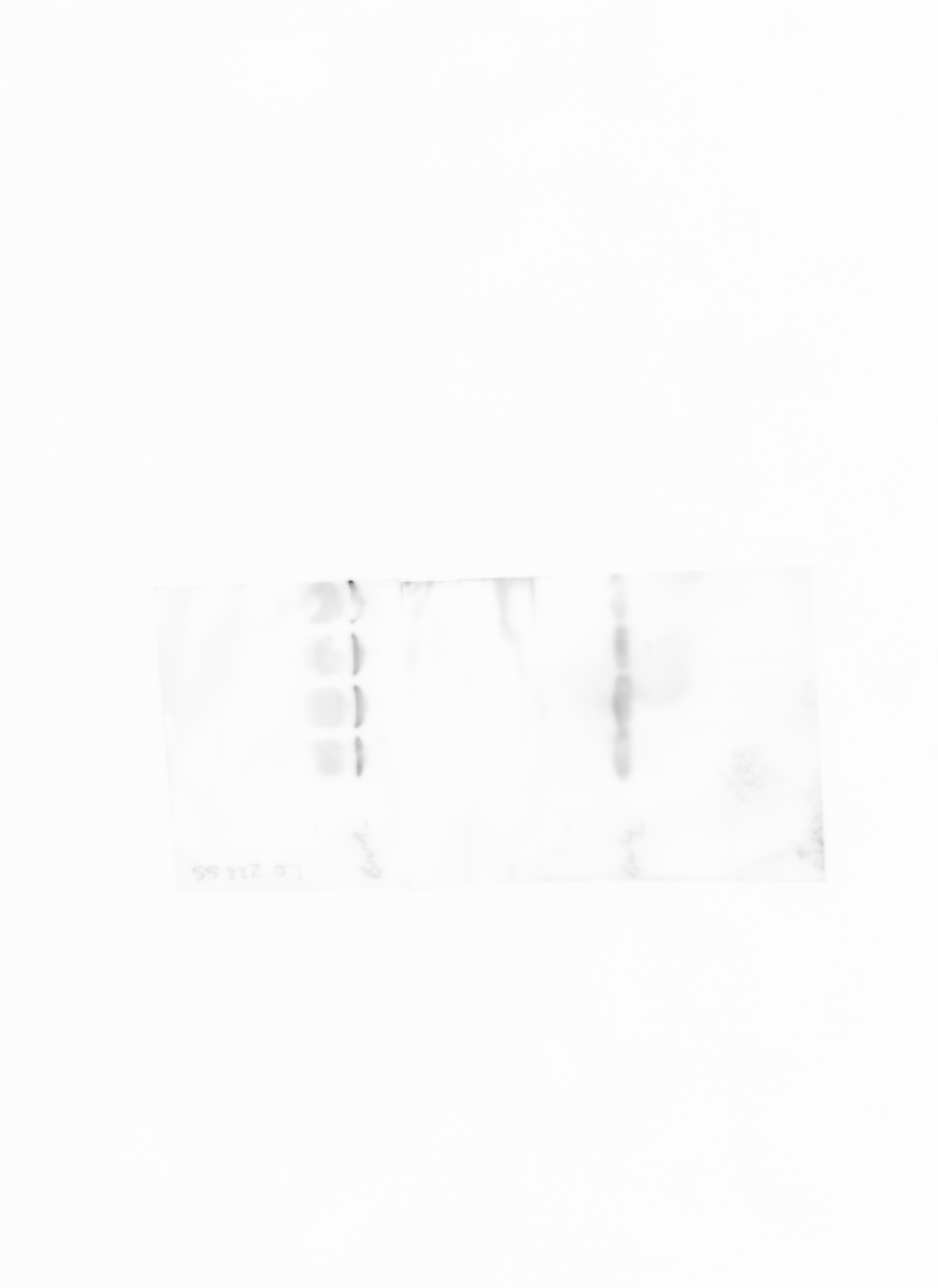

Supplement: Figure 2—figure supplement 1—source data 1. [file elife-68213-fig2-figsupp1-data1.zip › Figure_2_Supplement_1_source_data/Figure_2_supplement_1_Source_data_1_Figure_2_supplement_1A/Original_files/2nd 20210604_160237-17_Ch_Chemi.jpg]

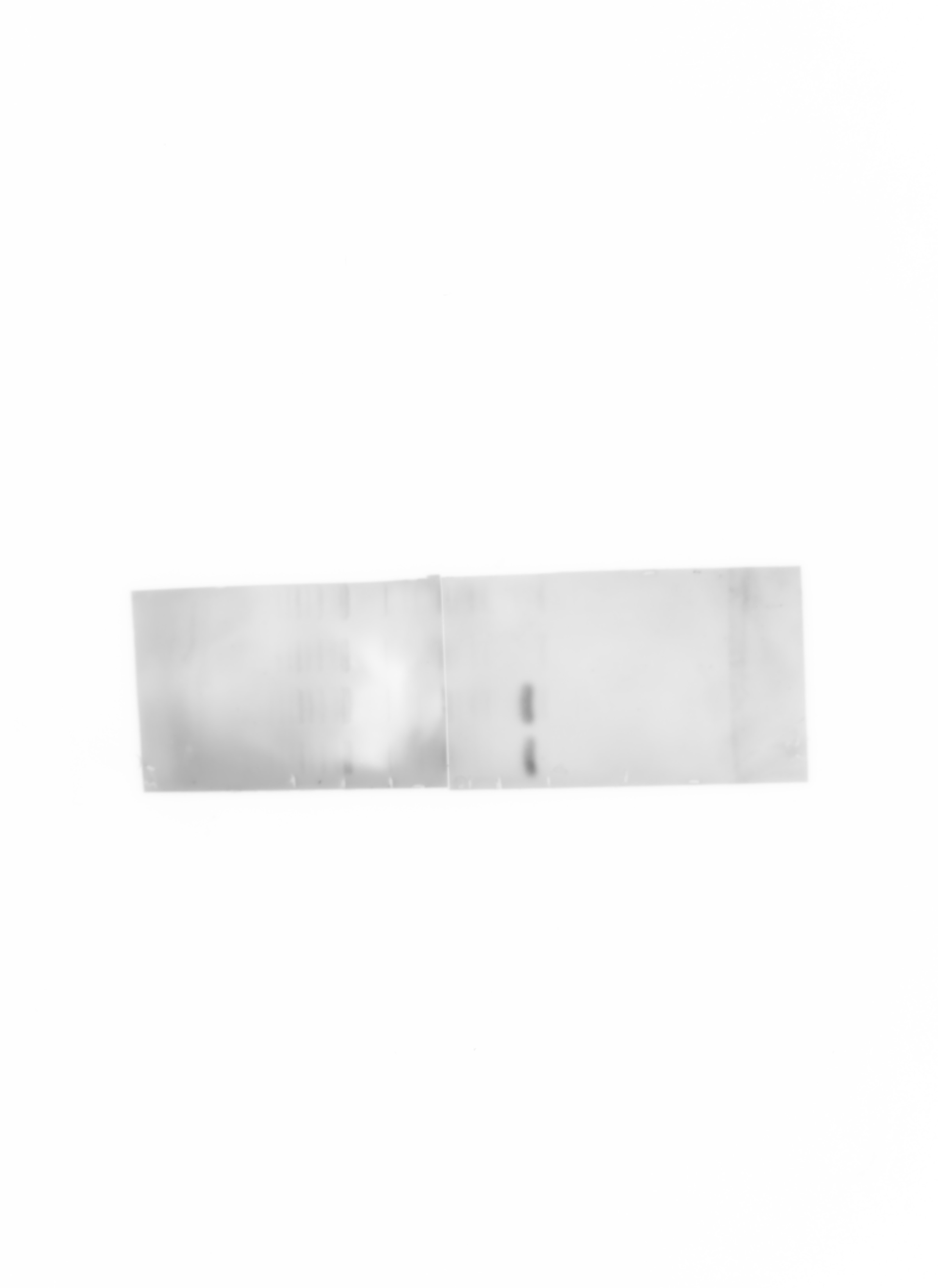

Supplement: Figure 2—figure supplement 1—source data 1. [file elife-68213-fig2-figsupp1-data1.zip › Figure_2_Supplement_1_source_data/Figure_2_supplement_1_Source_data_1_Figure_2_supplement_1A/Original_files/redeco 20210609_164247-09_Ch_Chemi.jpg]

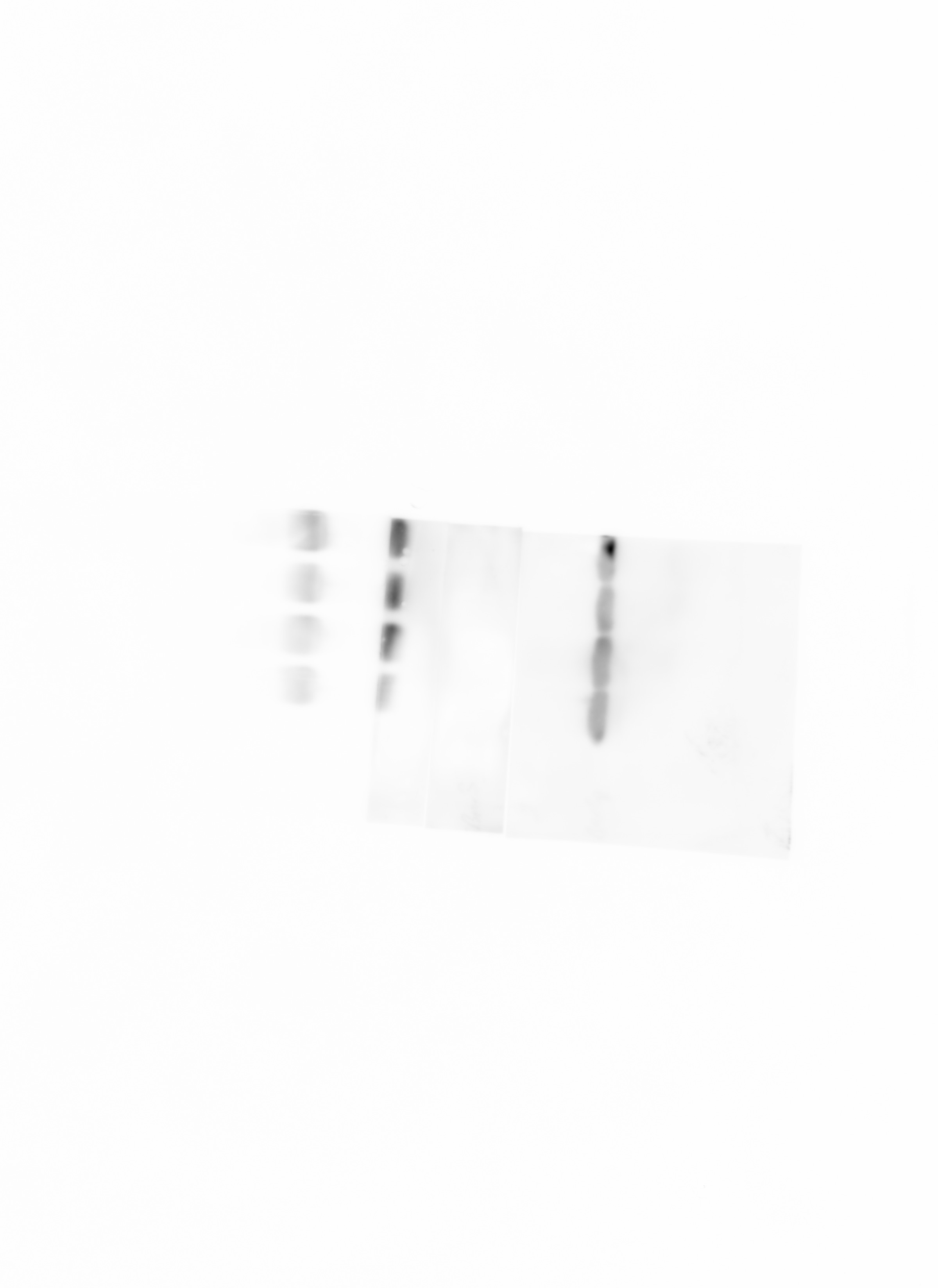

Supplement: Figure 2—figure supplement 1—source data 1. [file elife-68213-fig2-figsupp1-data1.zip › Figure_2_Supplement_1_source_data/Figure_2_supplement_1_Source_data_1_Figure_2_supplement_1A/Original_files/1st expo 20210603_175753-08_Ch_Chemi.jpg]

Figure 3 source data 1 related to Figure 3B

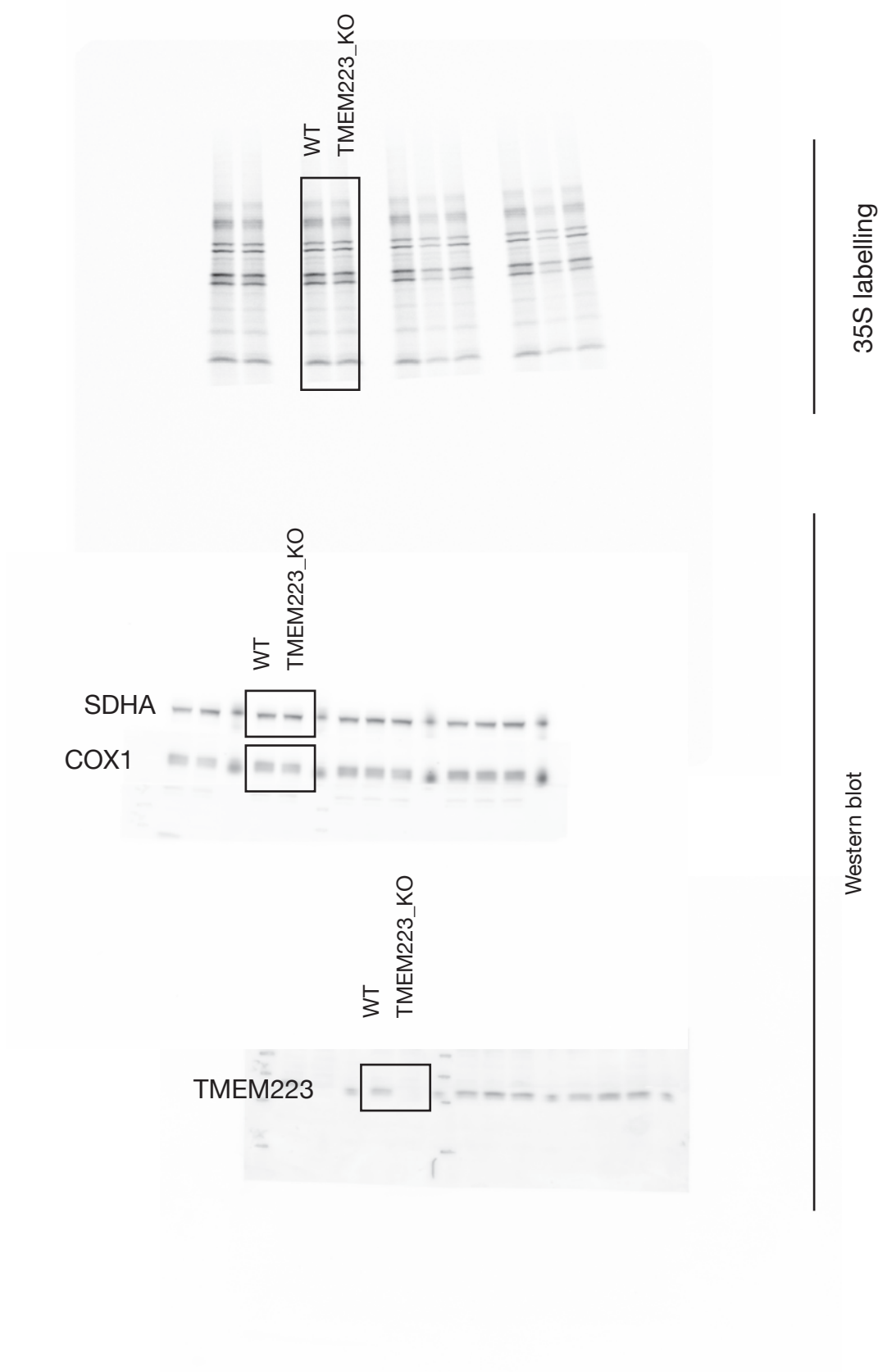

Supplement: Figure 3—source data 1. [file elife-68213-fig3-data1.zip › Figure_3_source_data/Figure_3_source_data_1_Figure_3B/Data_labelled/Figure_3_source_data_1_related_Figure_3B.pdf]

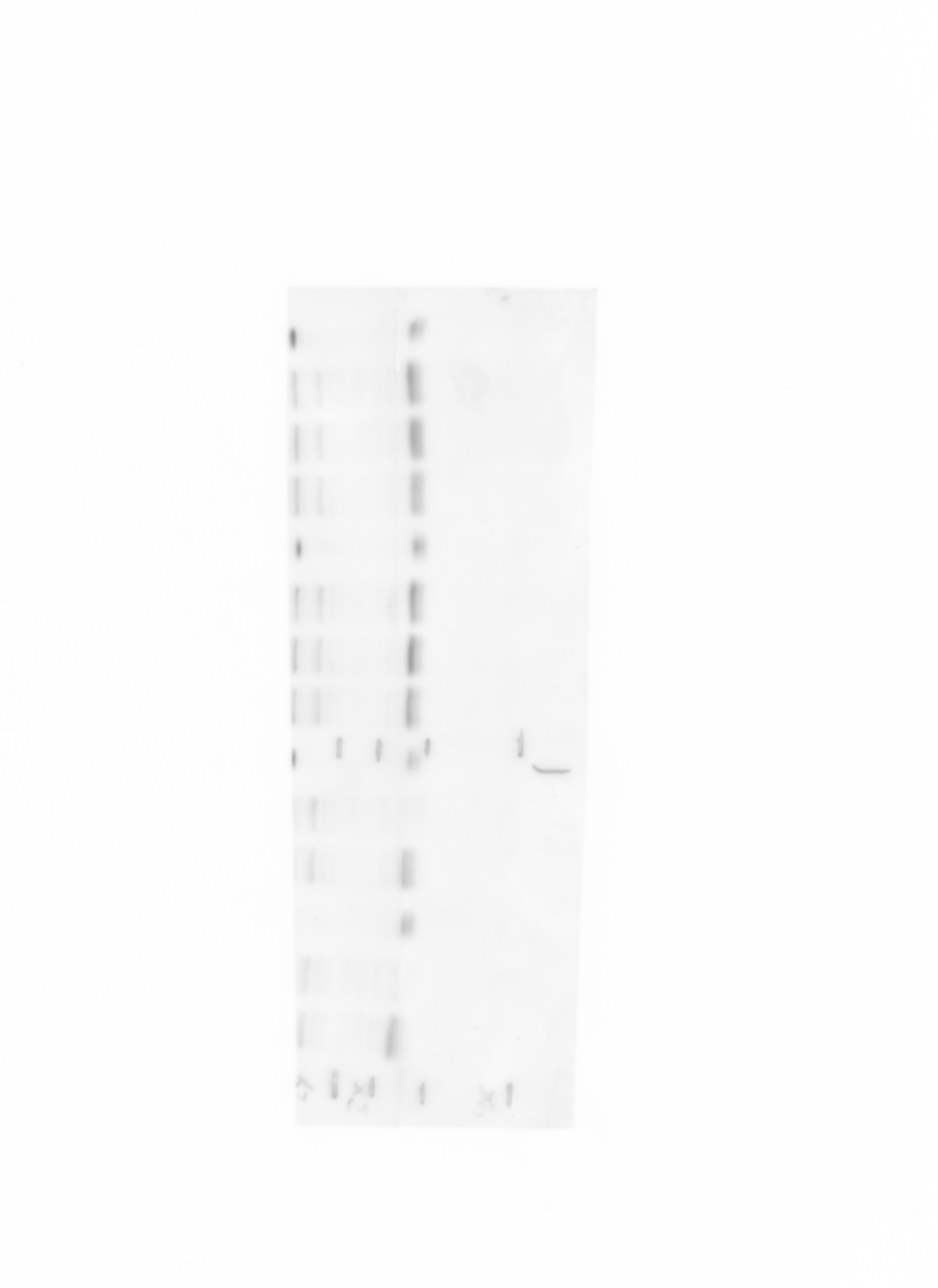

Supplement: Figure 3—source data 1. [file elife-68213-fig3-data1.zip › Figure_3_source_data/Figure_3_source_data_1_Figure_3B/Original_data/2nd expo 20200617_122355-20_Ch_Chemi.jpg]

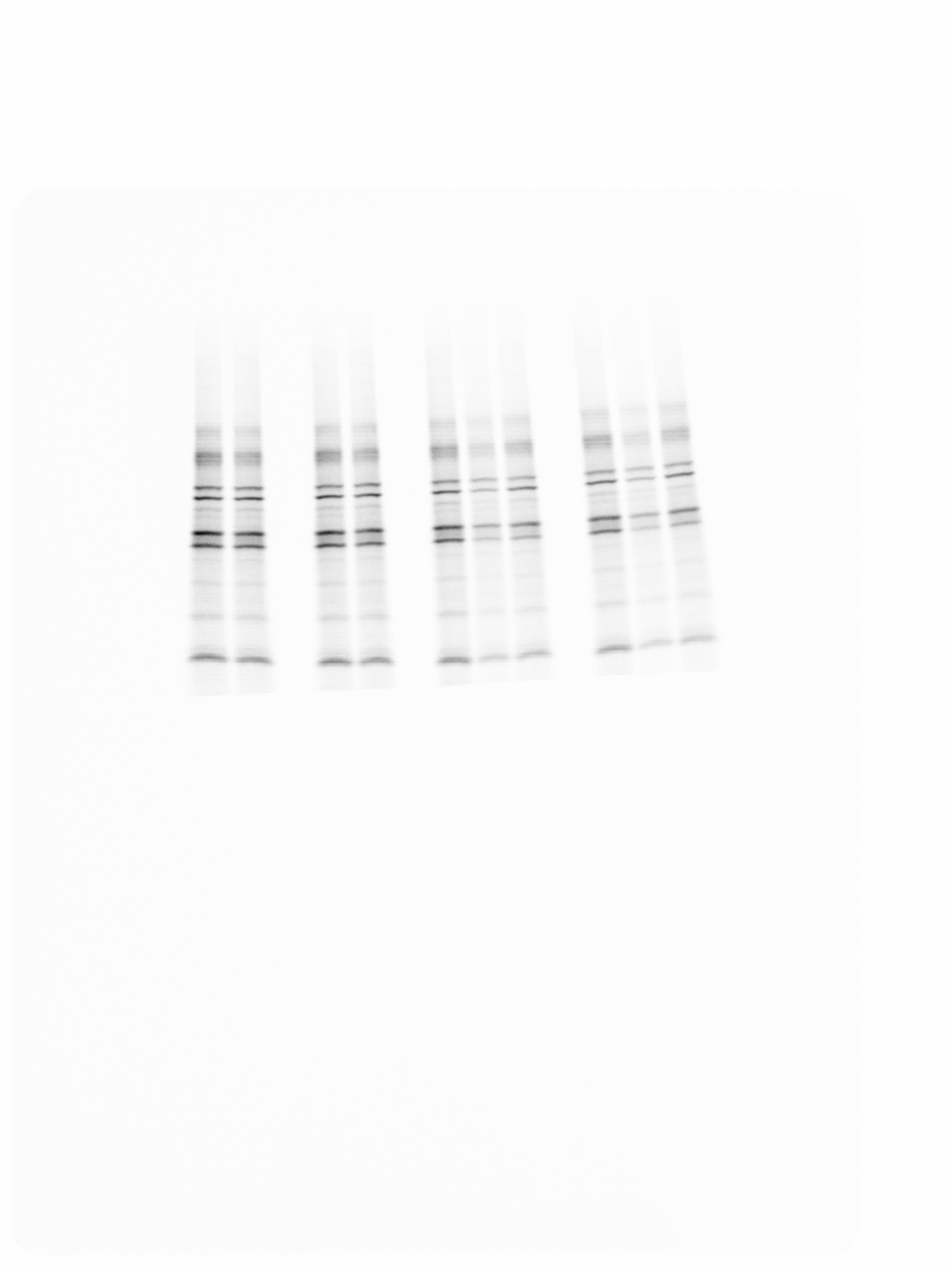

Supplement: Figure 3—source data 1. [file elife-68213-fig3-data1.zip › Figure_3_source_data/Figure_3_source_data_1_Figure_3B/Original_data/2days_expo-[Phosphor].jpg]

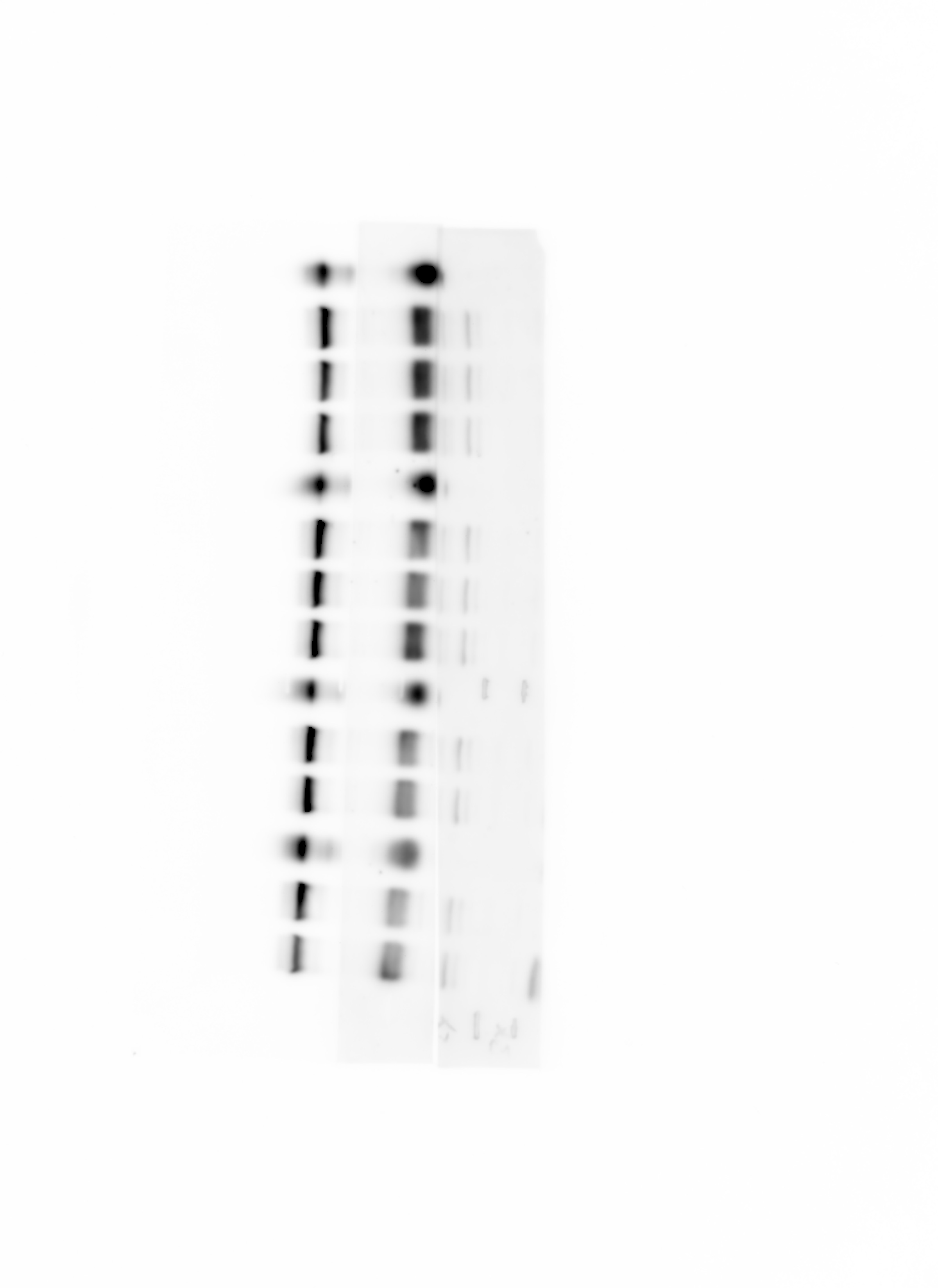

Supplement: Figure 3—source data 1. [file elife-68213-fig3-data1.zip › Figure_3_source_data/Figure_3_source_data_1_Figure_3B/Original_data/SD_342_343_34_345_1st_deco 20200616_123251-17_Ch_Chemi.jpg]

Figure 3 source data 2 related to Figure 3D

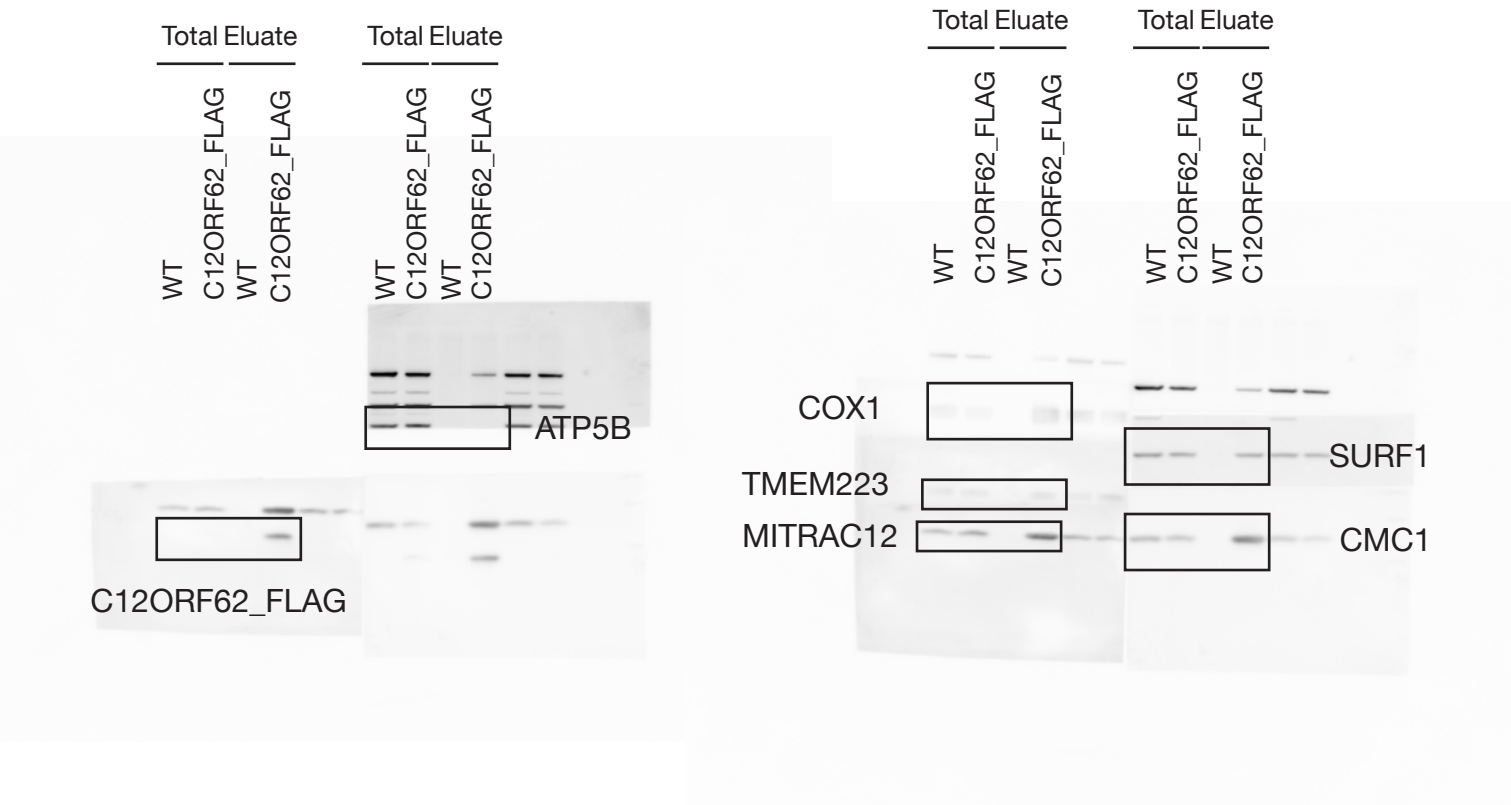

Supplement: Figure 3—source data 1. [file elife-68213-fig3-data1.zip › Figure_3_source_data/Figure_3_source_data_2_Figure_3D/Data_labelled/Figure_3_source_data_2_related_Figure_3D.pdf]

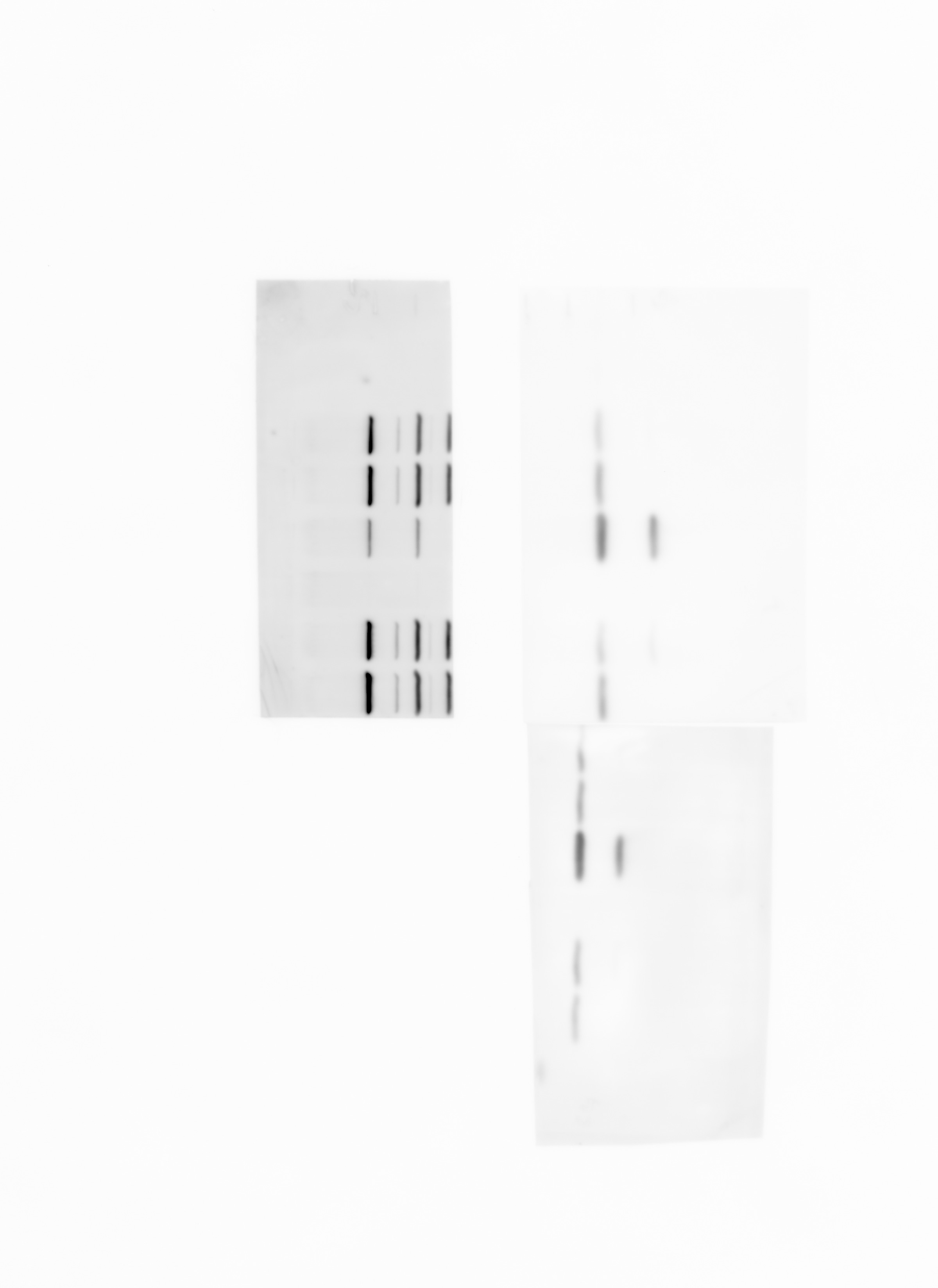

Supplement: Figure 3—source data 1. [file elife-68213-fig3-data1.zip › Figure_3_source_data/Figure_3_source_data_2_Figure_3D/Original_data/2nd 20210623_143210-20_Ch_Chemi.jpg]

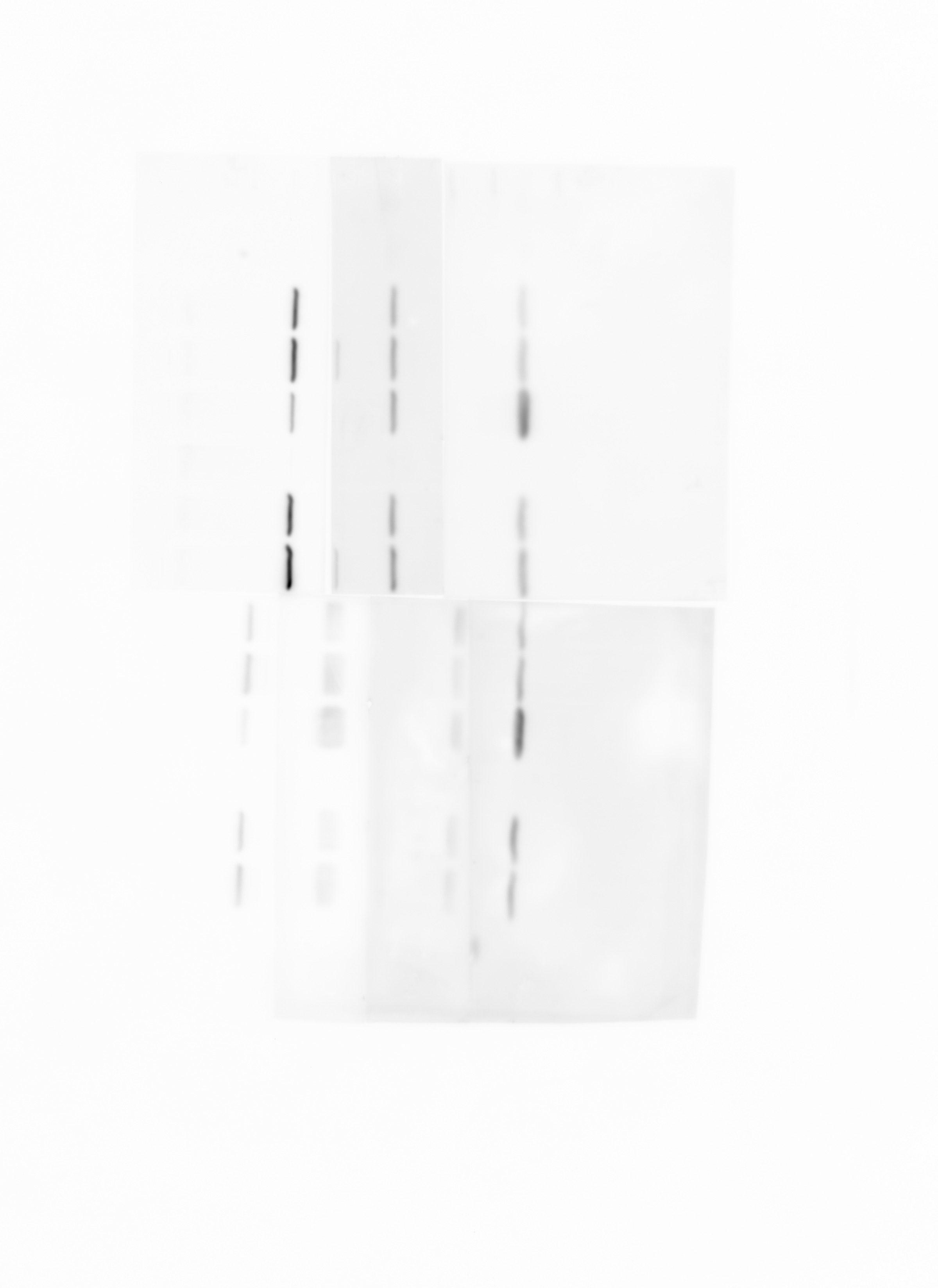

Supplement: Figure 3—source data 1. [file elife-68213-fig3-data1.zip › Figure_3_source_data/Figure_3_source_data_2_Figure_3D/Original_data/1st expo 20210622_150125-20_Ch_Chemi.jpg]

Figure 3 source data 3 related to Figure 3E

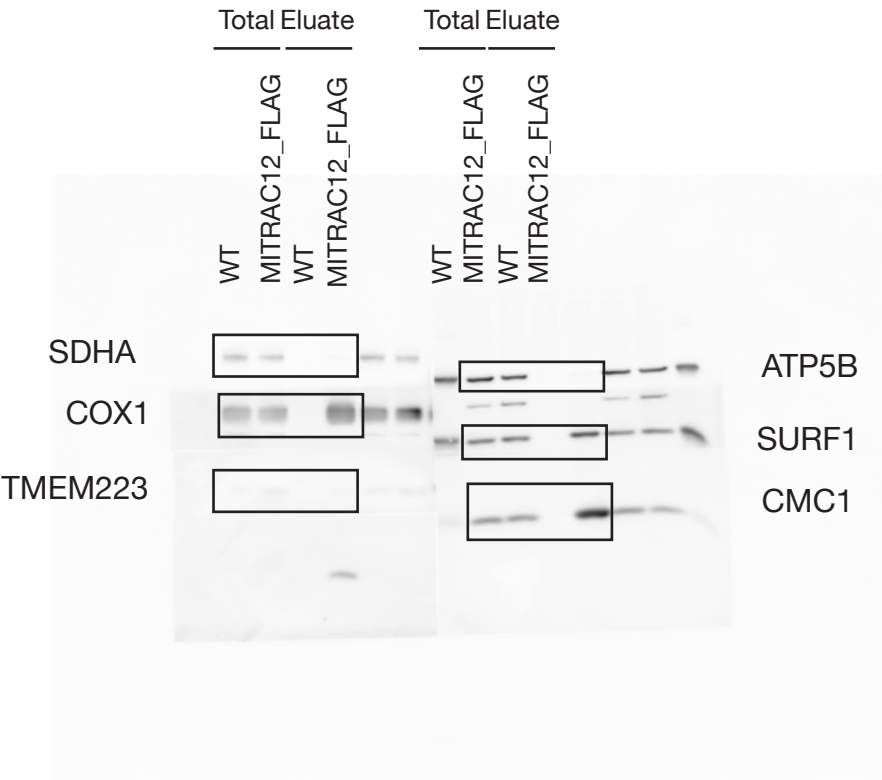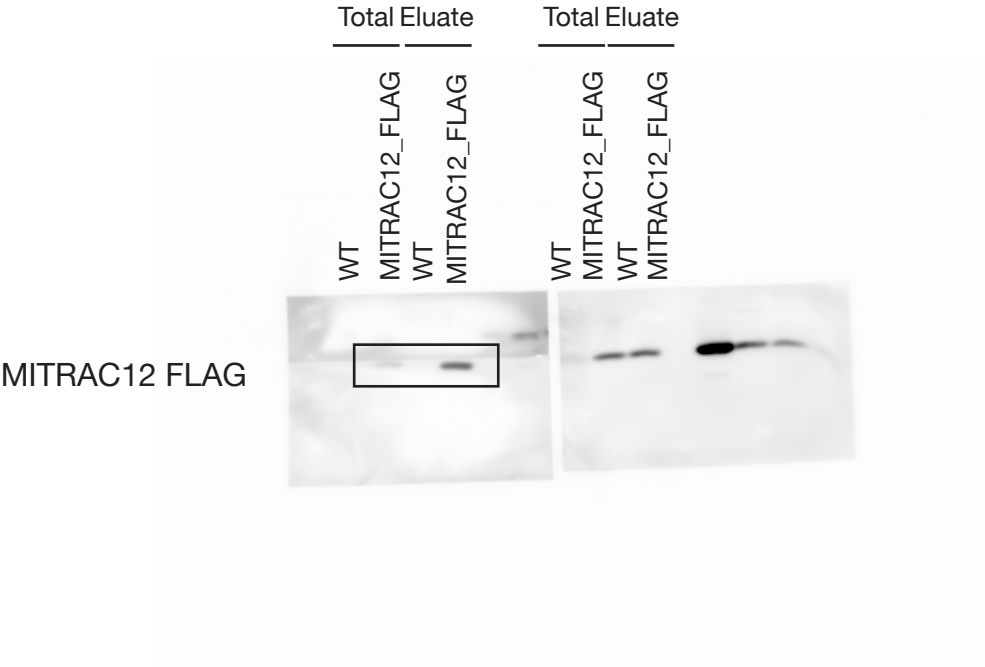

Supplement: Figure 3—source data 1. [file elife-68213-fig3-data1.zip › Figure_3_source_data/Figure_3_source_data_3_Figure_3E/Data_labelled/Figure_3_source_data_3_Figure_3E.pdf]

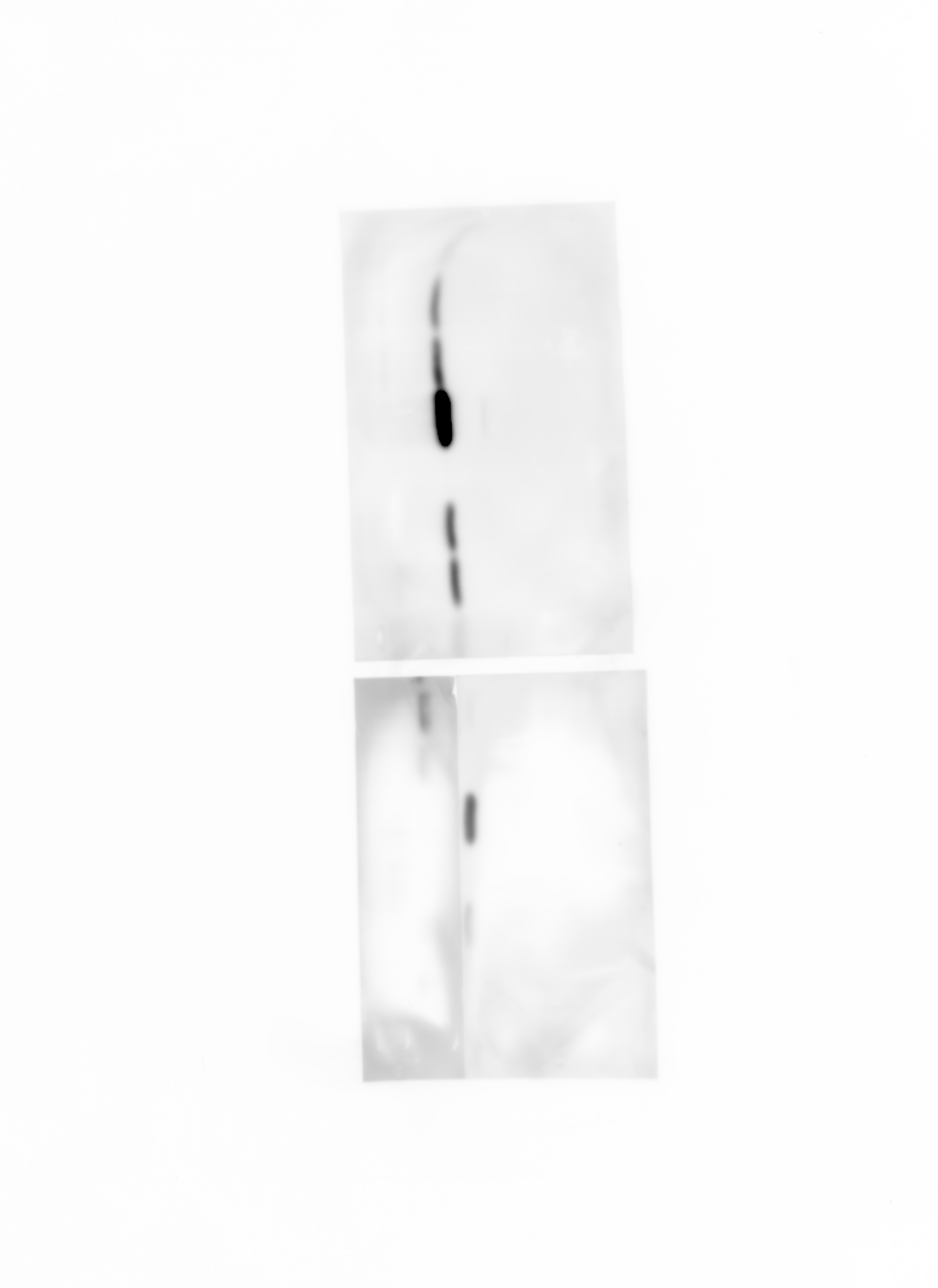

Supplement: Figure 3—source data 1. [file elife-68213-fig3-data1.zip › Figure_3_source_data/Figure_3_source_data_3_Figure_3E/Original_data/2nd flag 20210625_132828-20_Ch_Chemi.jpg]

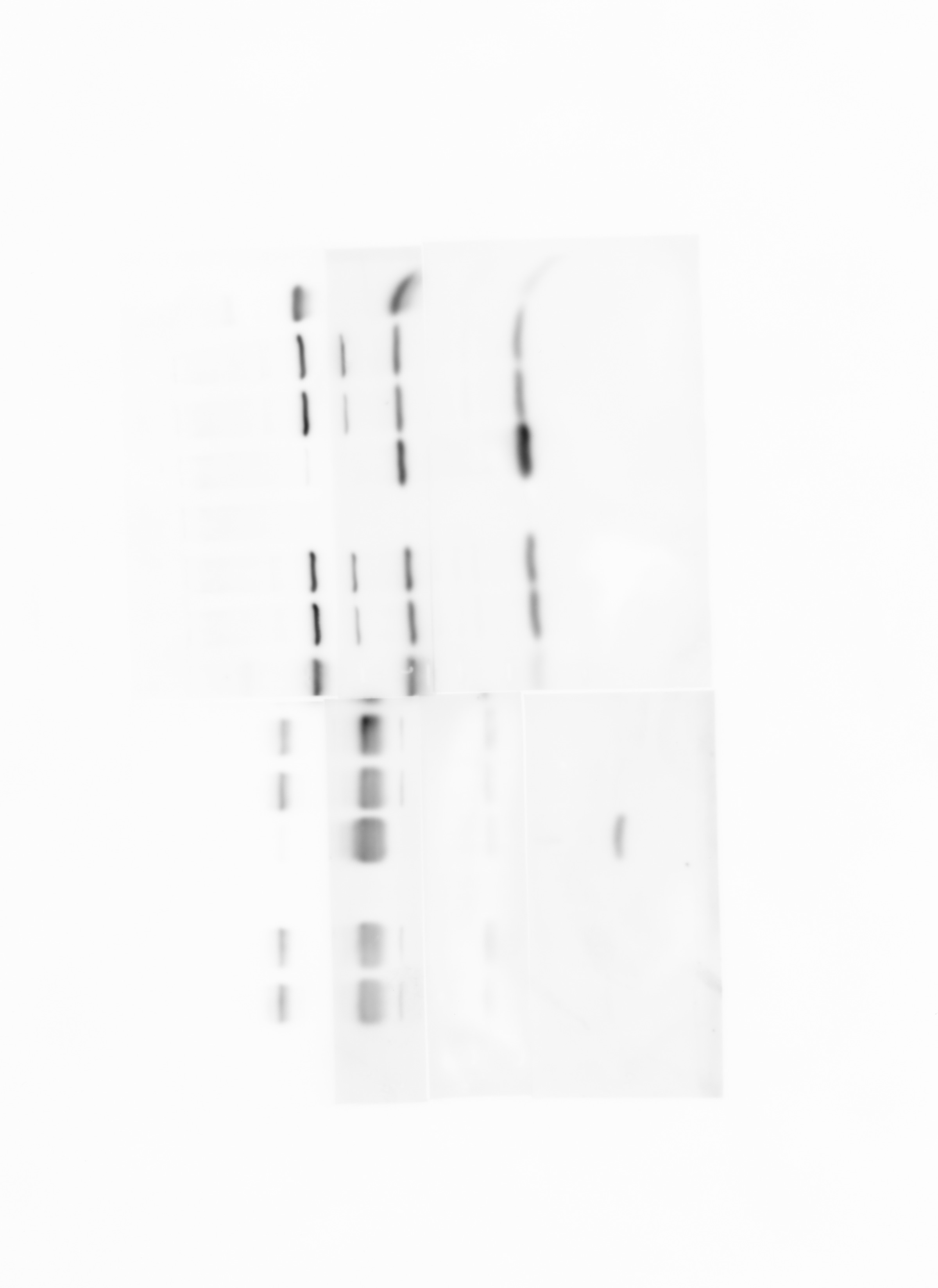

Supplement: Figure 3—source data 1. [file elife-68213-fig3-data1.zip › Figure_3_source_data/Figure_3_source_data_3_Figure_3E/Original_data/1st 20210624_122411-20_Ch_Chemi.jpg]

Figure 3 source data 4 related to Figure 3F

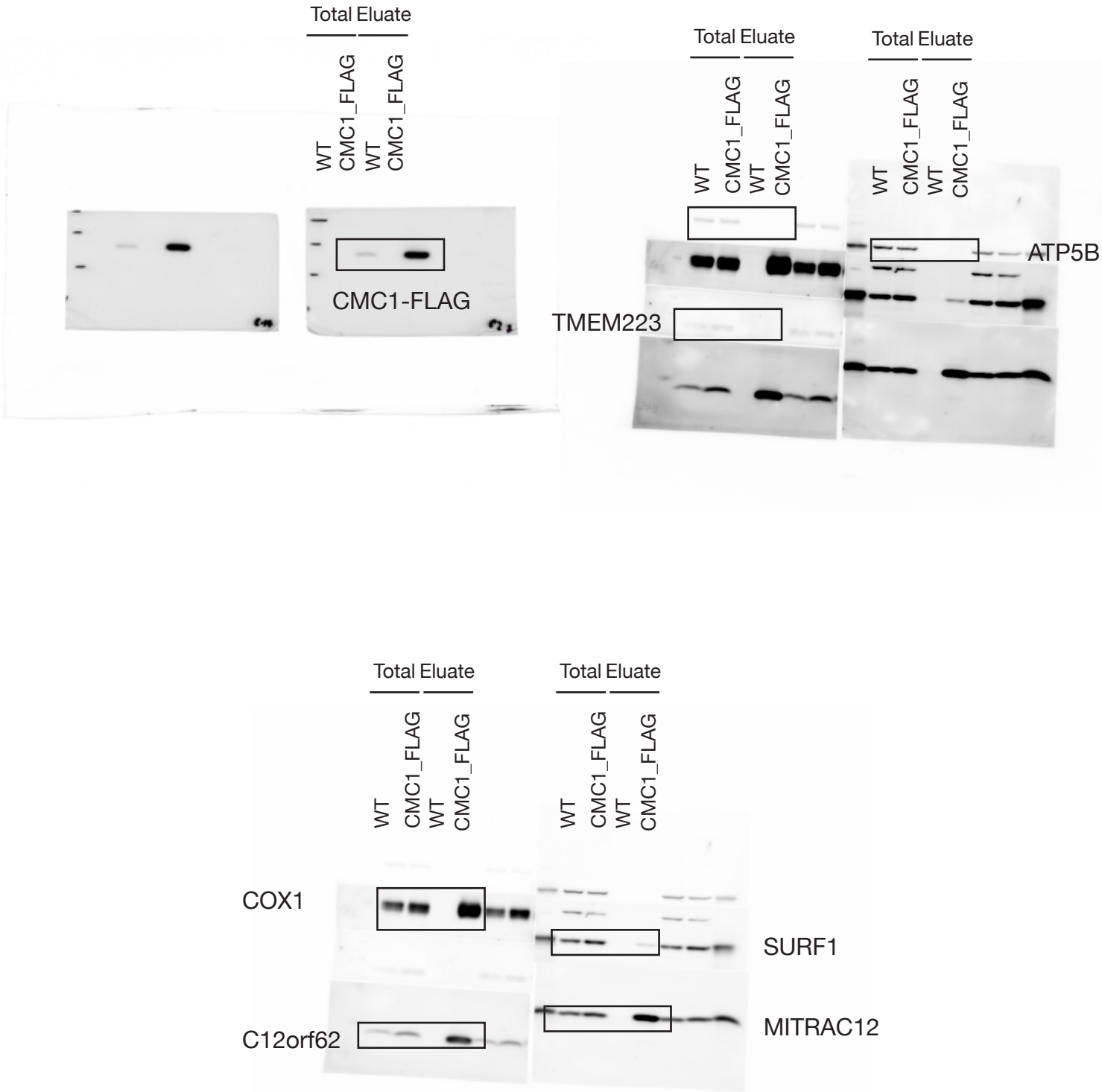

Supplement: Figure 3—source data 1. [file elife-68213-fig3-data1.zip › Figure_3_source_data/Figure_3_source_data_4_Figure_3F/Data_labelled/Figure_3_source_data_4_Figure_3F.pdf]

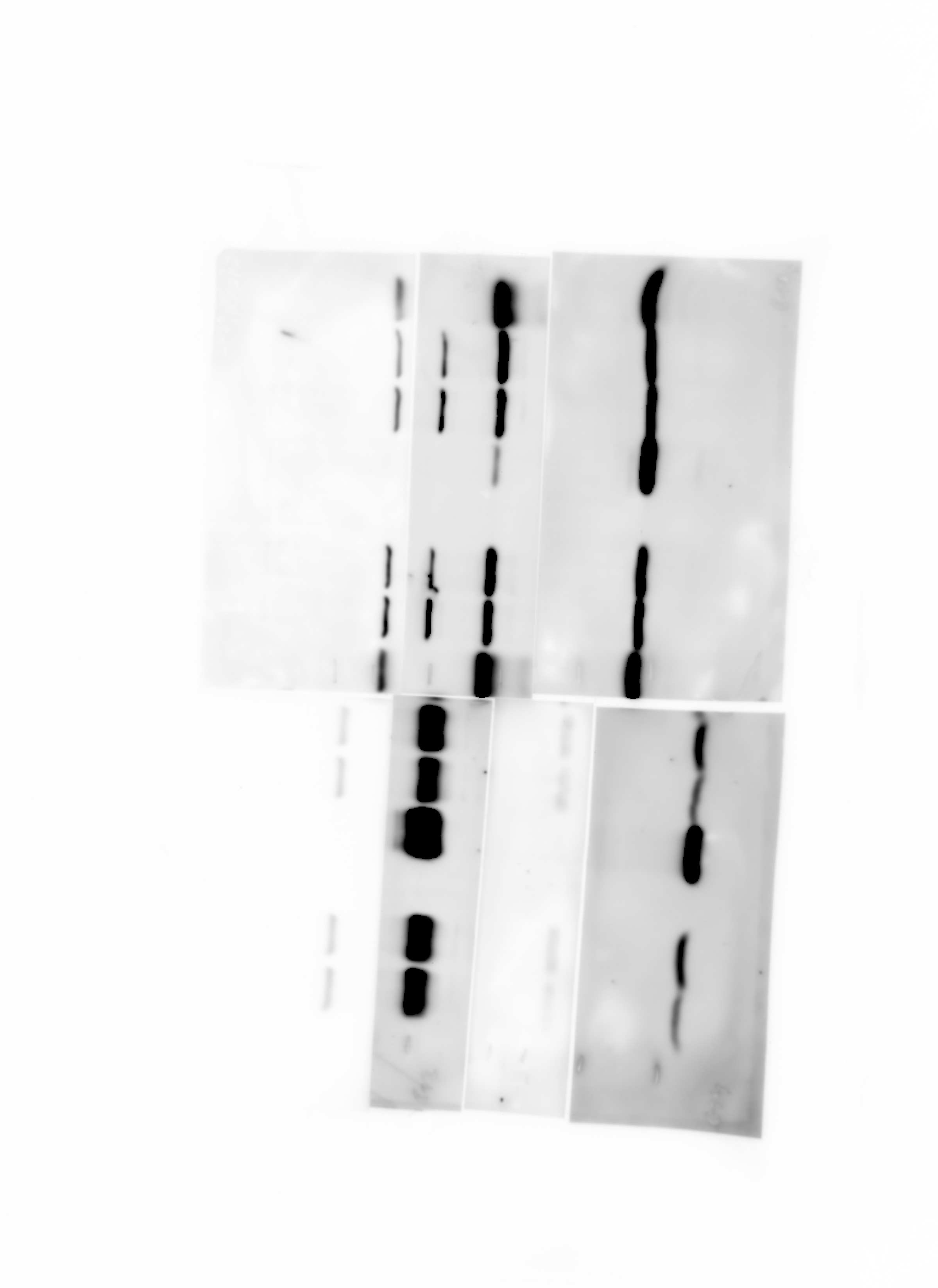

Supplement: Figure 3—source data 1. [file elife-68213-fig3-data1.zip › Figure_3_source_data/Figure_3_source_data_4_Figure_3F/Original_data/1st 20210630_133228-20_Ch_Chemi.jpg]

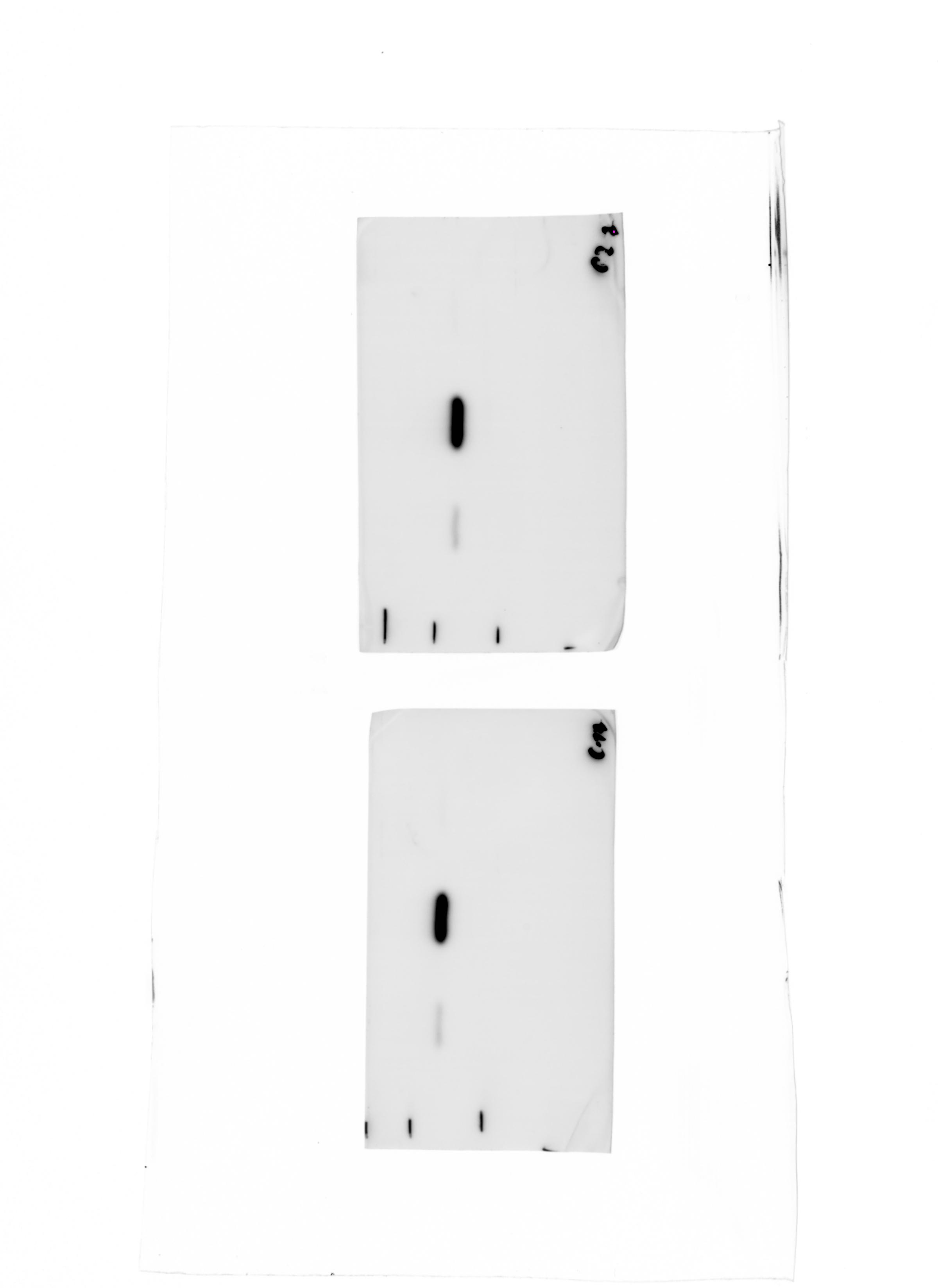

Supplement: Figure 3—source data 1. [file elife-68213-fig3-data1.zip › Figure_3_source_data/Figure_3_source_data_4_Figure_3F/Original_data/FLAG 20210701_145752_Fl_IRshort.jpg]

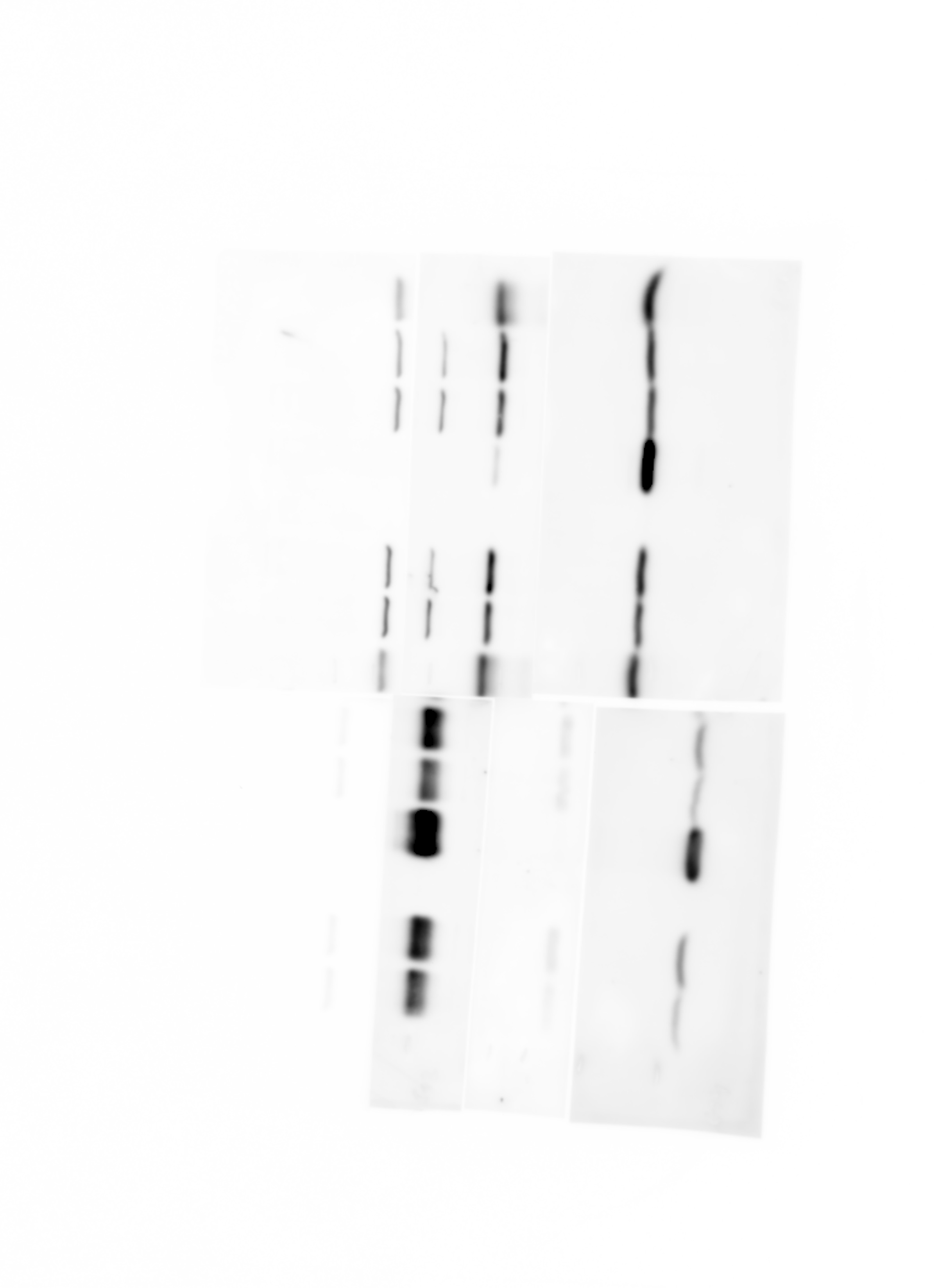

Supplement: Figure 3—source data 1. [file elife-68213-fig3-data1.zip › Figure_3_source_data/Figure_3_source_data_4_Figure_3F/Original_data/1st 20210630_133228-05_Ch_Chemi.jpg]

Figure 3 source data 5 related to Figure 3G

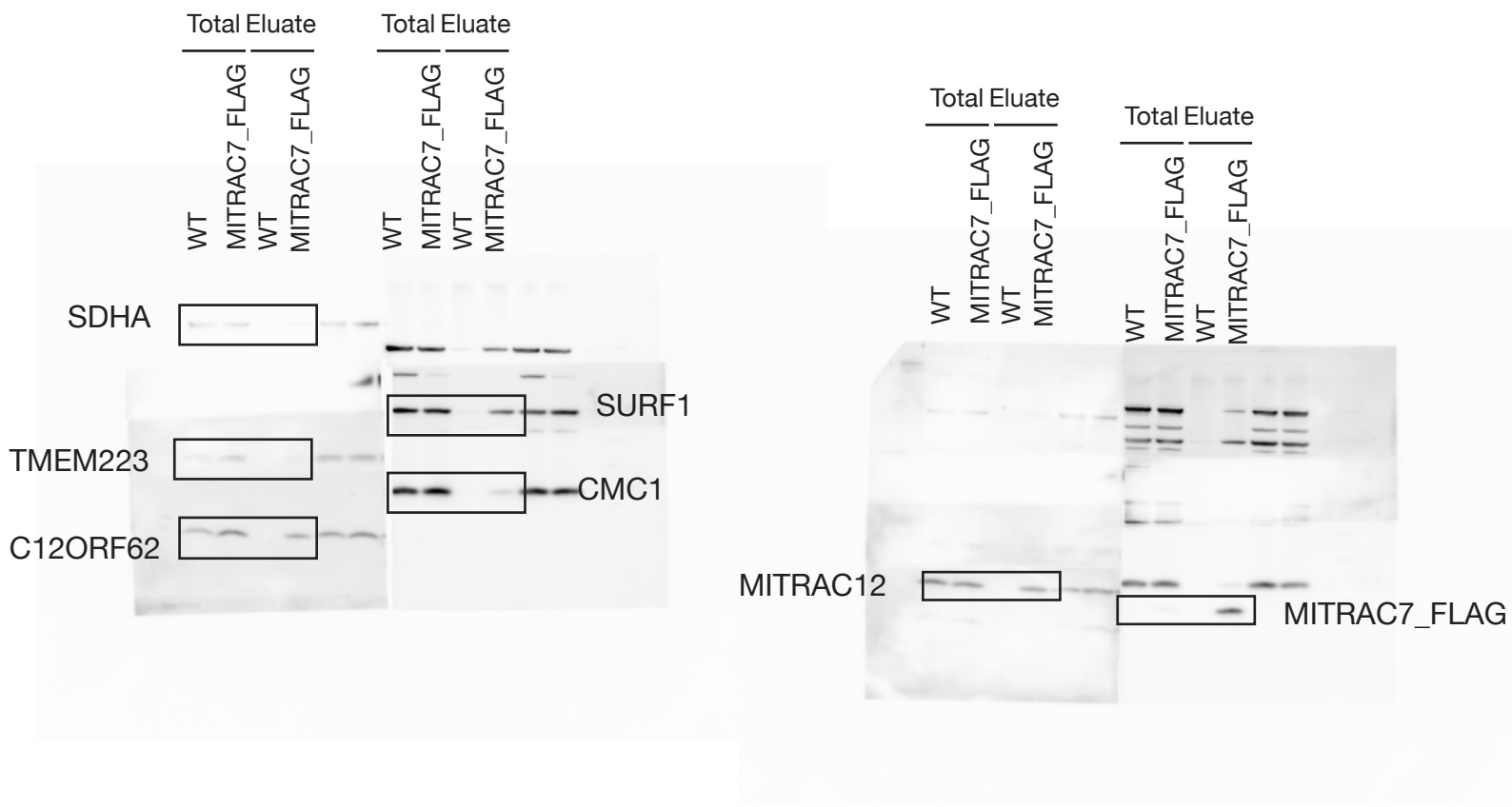

Supplement: Figure 3—source data 1. [file elife-68213-fig3-data1.zip › Figure_3_source_data/Figure_3_soure_data_5_Figure_3G/Data_labelled/Figure_3_source_data_5_Figure_3G.pdf]

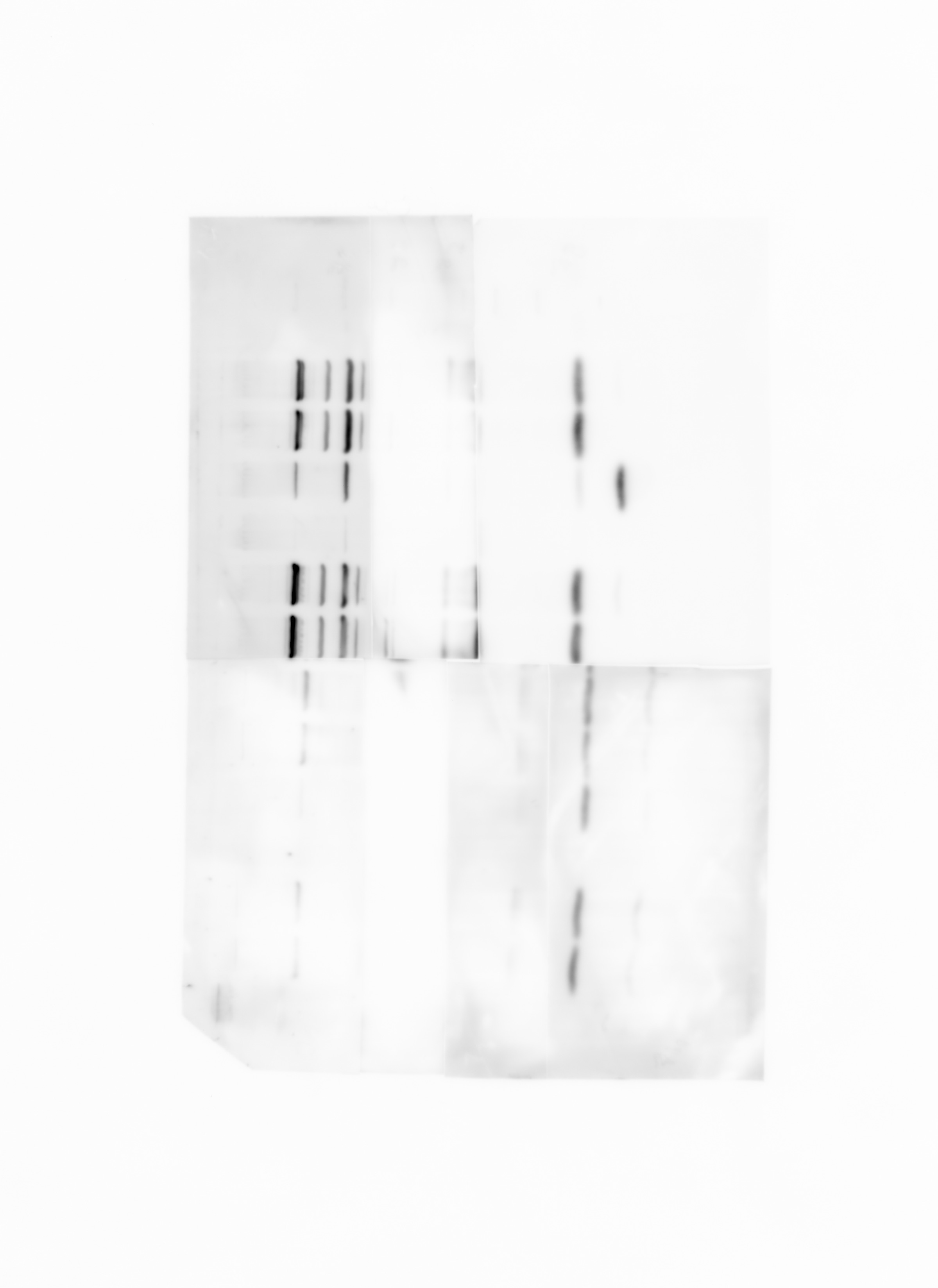

Supplement: Figure 3—source data 1. [file elife-68213-fig3-data1.zip › Figure_3_source_data/Figure_3_soure_data_5_Figure_3G/Original_data/2nd 20210623_144344-20_Ch_Chemi.jpg]

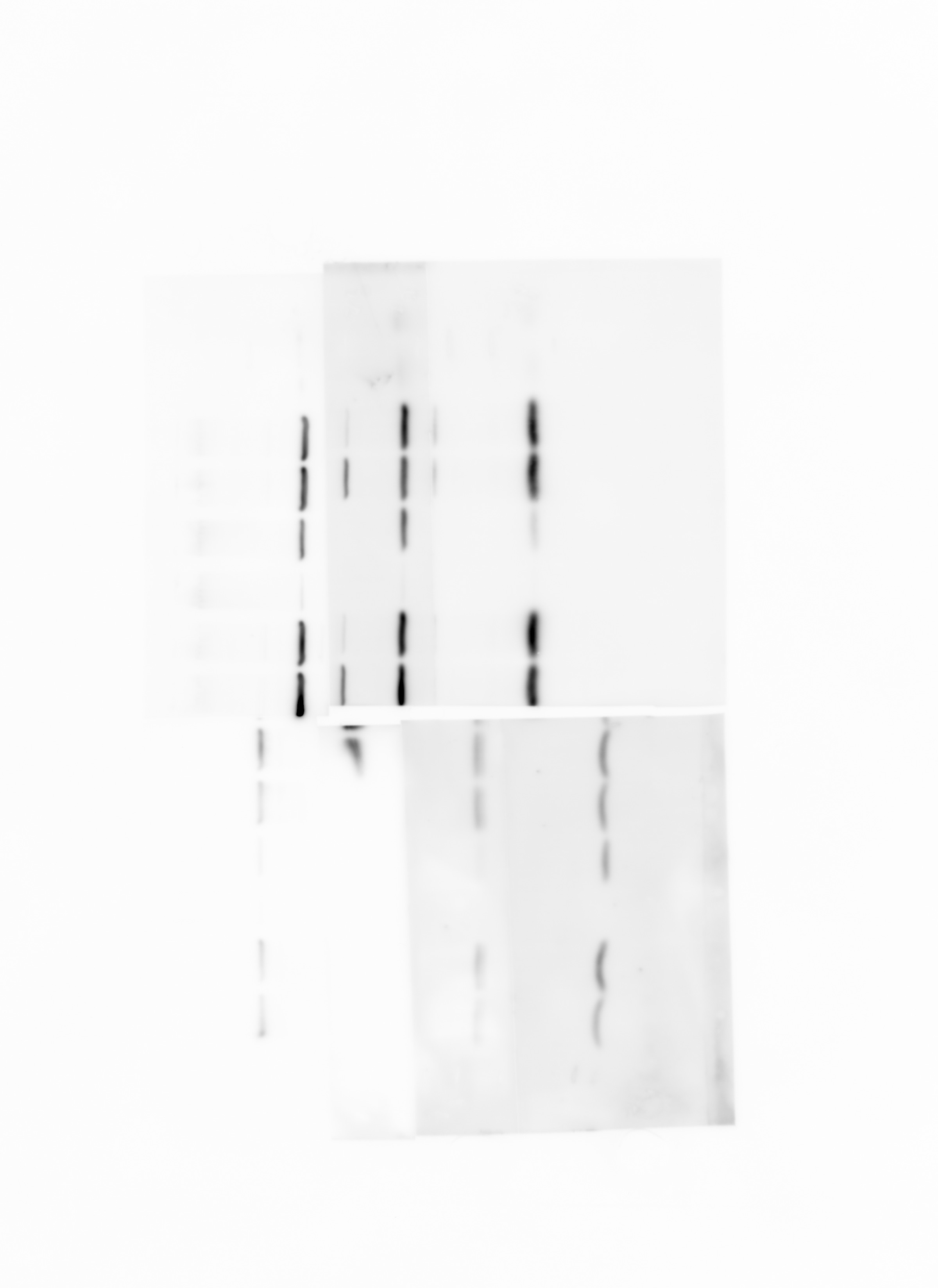

Supplement: Figure 3—source data 1. [file elife-68213-fig3-data1.zip › Figure_3_source_data/Figure_3_soure_data_5_Figure_3G/Original_data/1st expo 20210622_143750-20_Ch_Chemi.jpg]

Figure 4 source data 1 related to Figure 4C

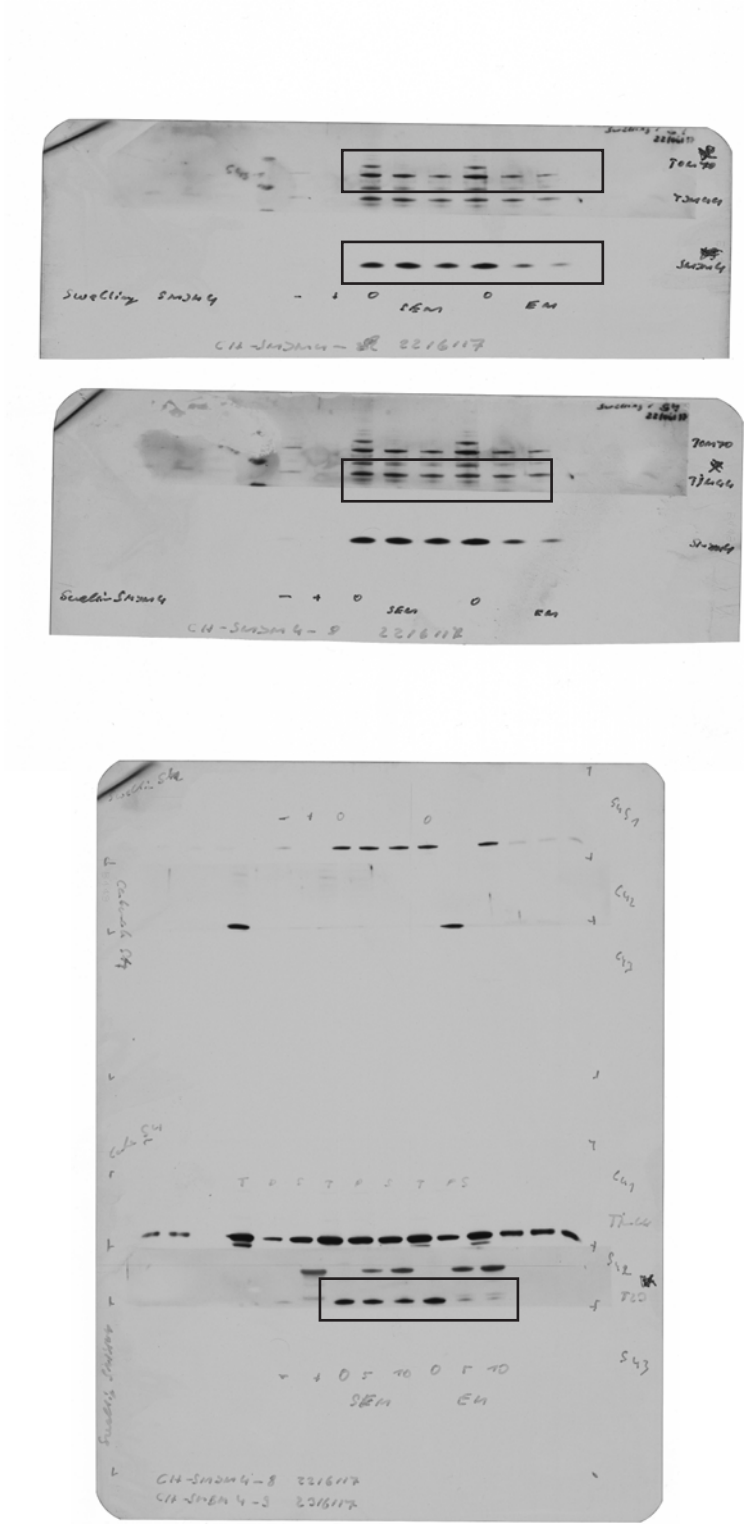

Supplement: Figure 4—source data 1. [file elife-68213-fig4-data1.zip › Figure_4_source_data/Figure_4_source_data_1_Figure_4C/Data_labelled/Figure_4_source_data_1_Figure_4C.pdf]

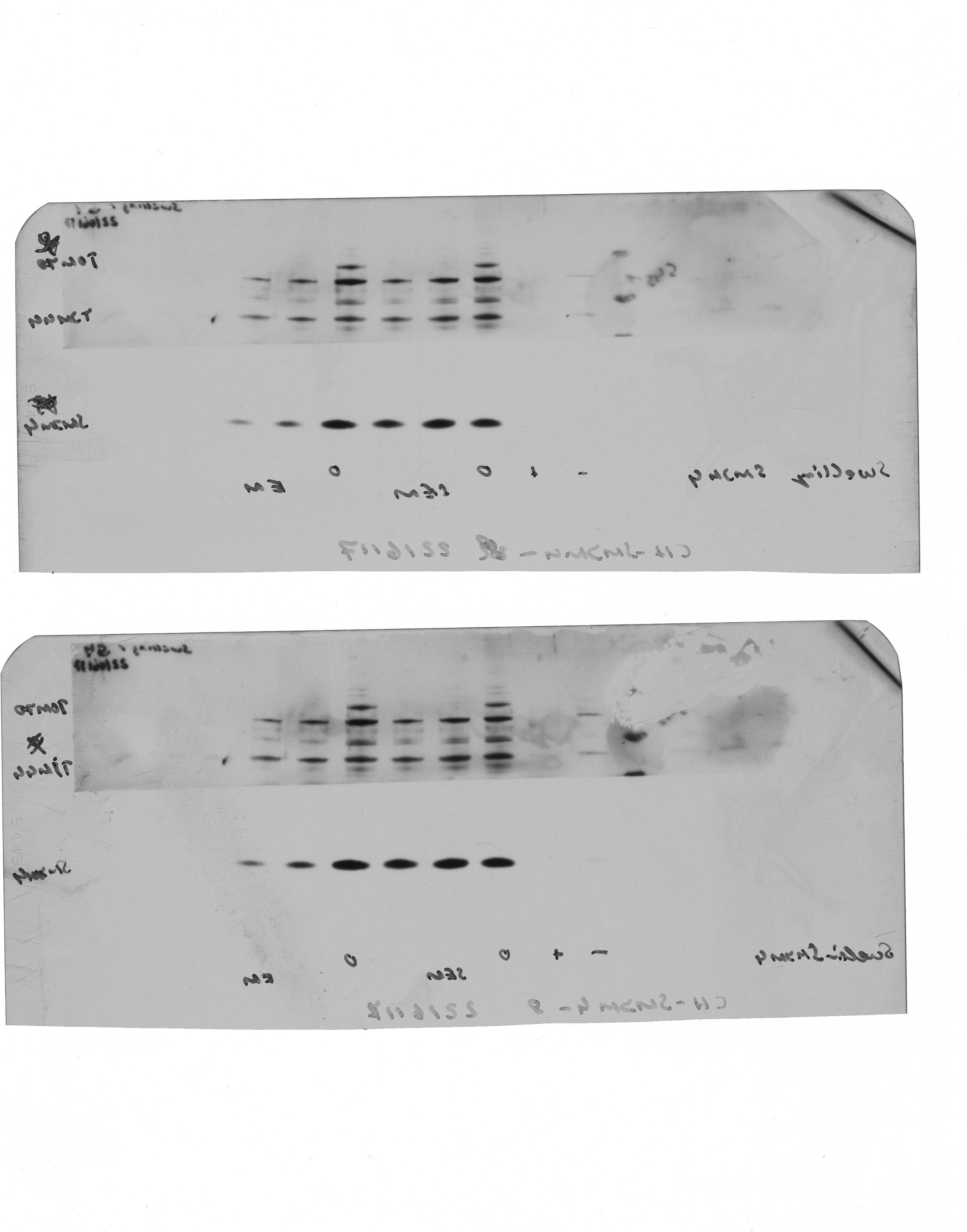

Supplement: Figure 4—source data 1. [file elife-68213-fig4-data1.zip › Figure_4_source_data/Figure_4_source_data_1_Figure_4C/Original_data/TOM70_TIMM44_SMIM4001.jpg]

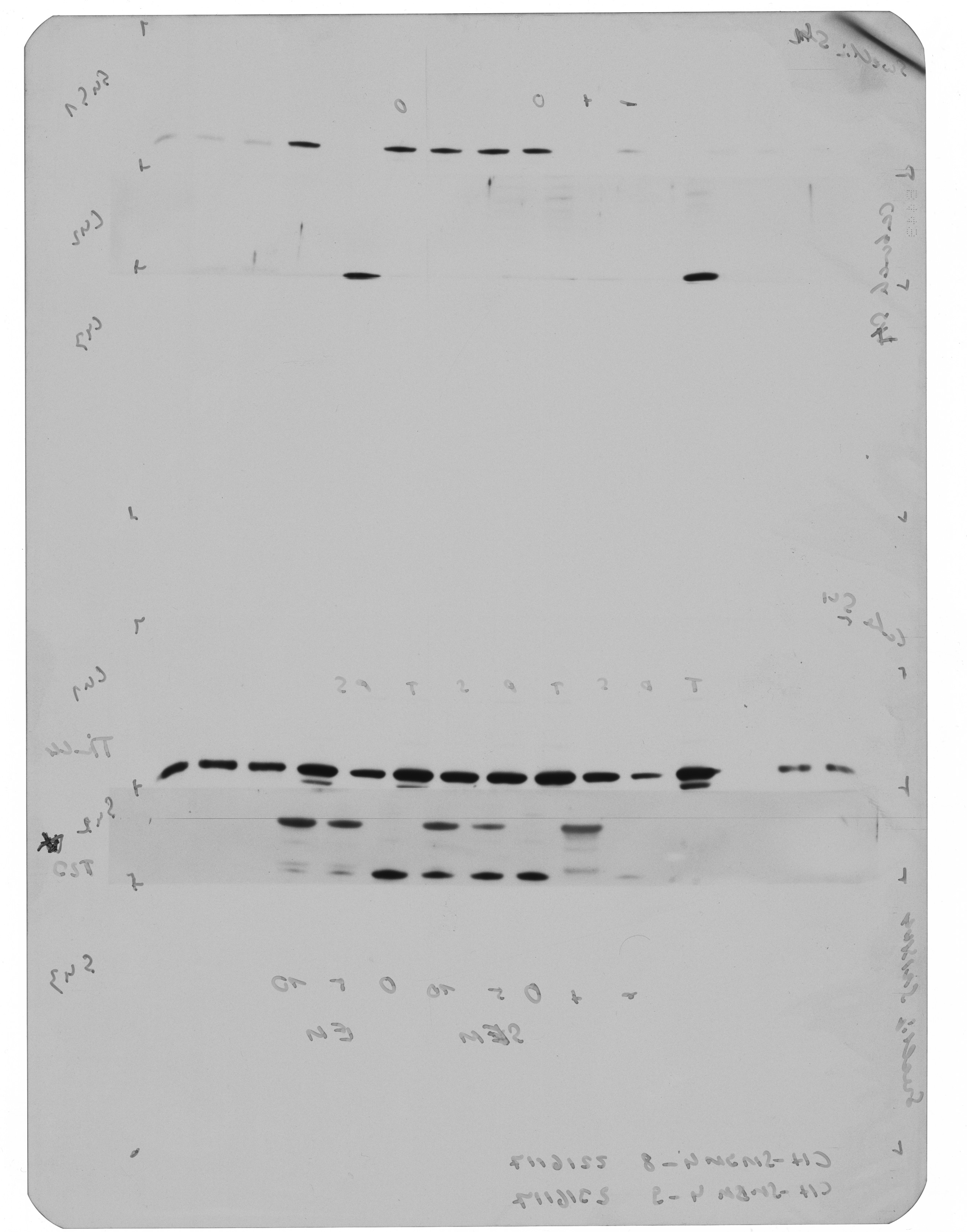

Supplement: Figure 4—source data 1. [file elife-68213-fig4-data1.zip › Figure_4_source_data/Figure_4_source_data_1_Figure_4C/Original_data/TIMM23001.jpg]

Figure 4 source data 2 related to Figure 4D

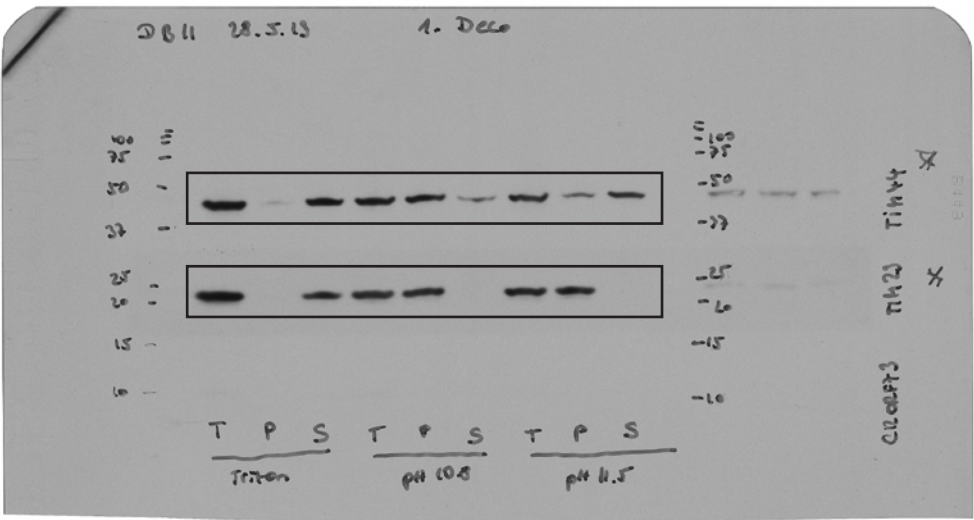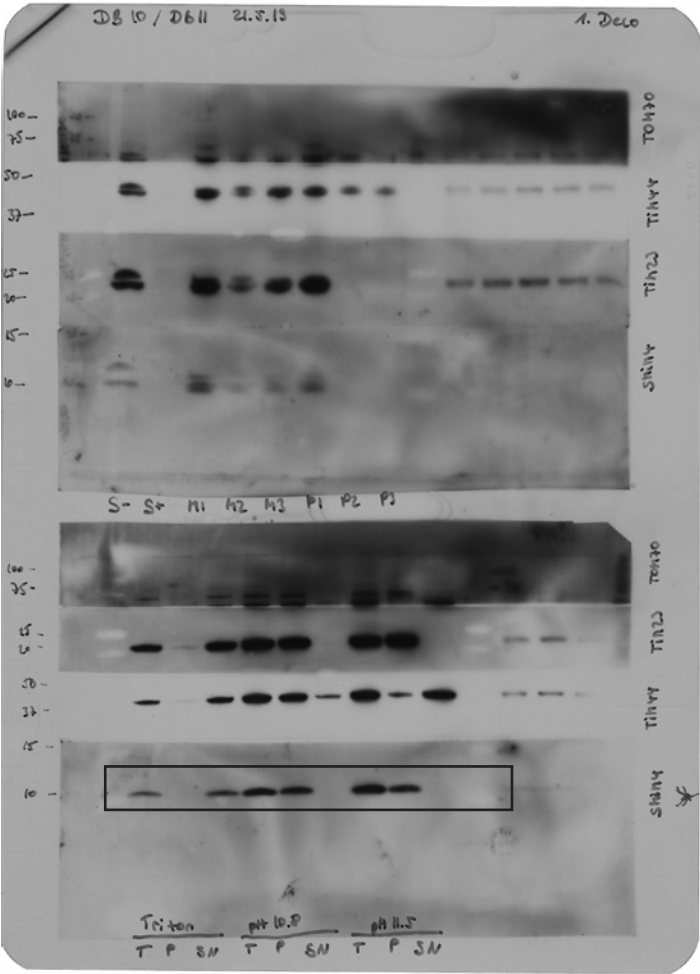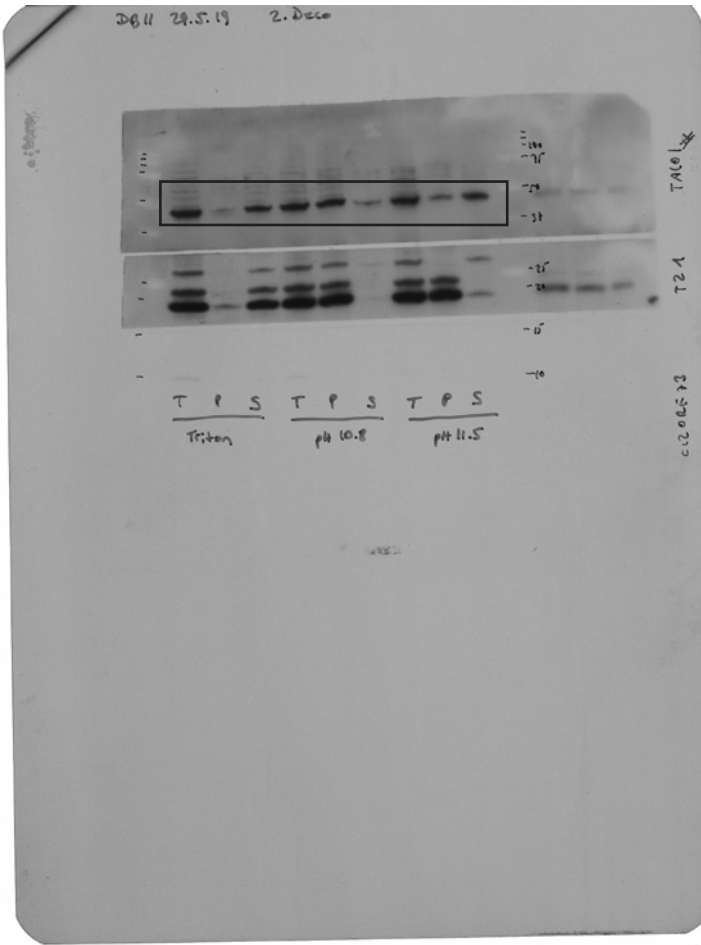

Supplement: Figure 4—source data 1. [file elife-68213-fig4-data1.zip › Figure_4_source_data/Figure_4_source_data_2_Figure_4D/Data_labelled/Figure_4_source_data_2_Figure_4D.pdf]

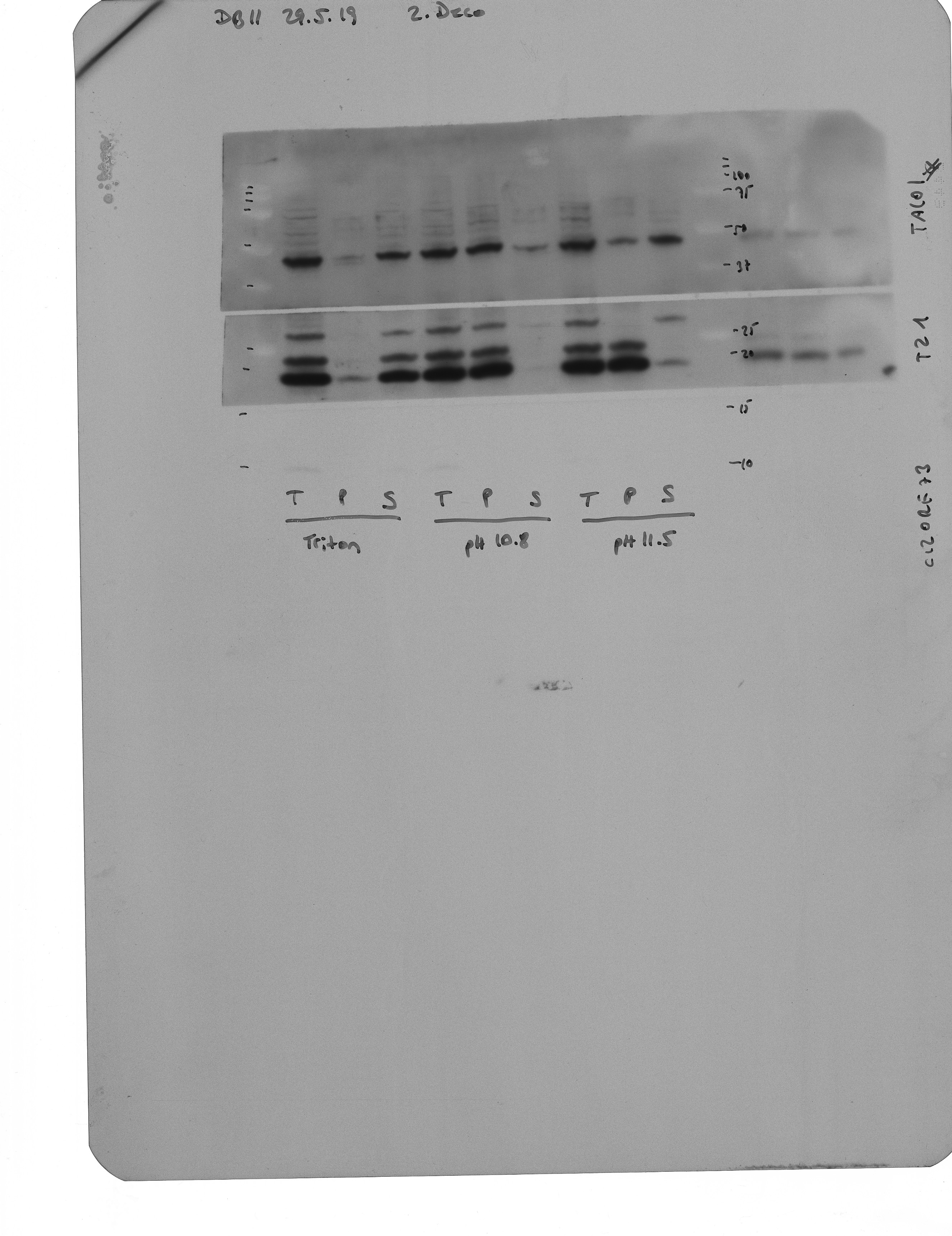

Supplement: Figure 4—source data 1. [file elife-68213-fig4-data1.zip › Figure_4_source_data/Figure_4_source_data_2_Figure_4D/Original_data/3004.jpg]

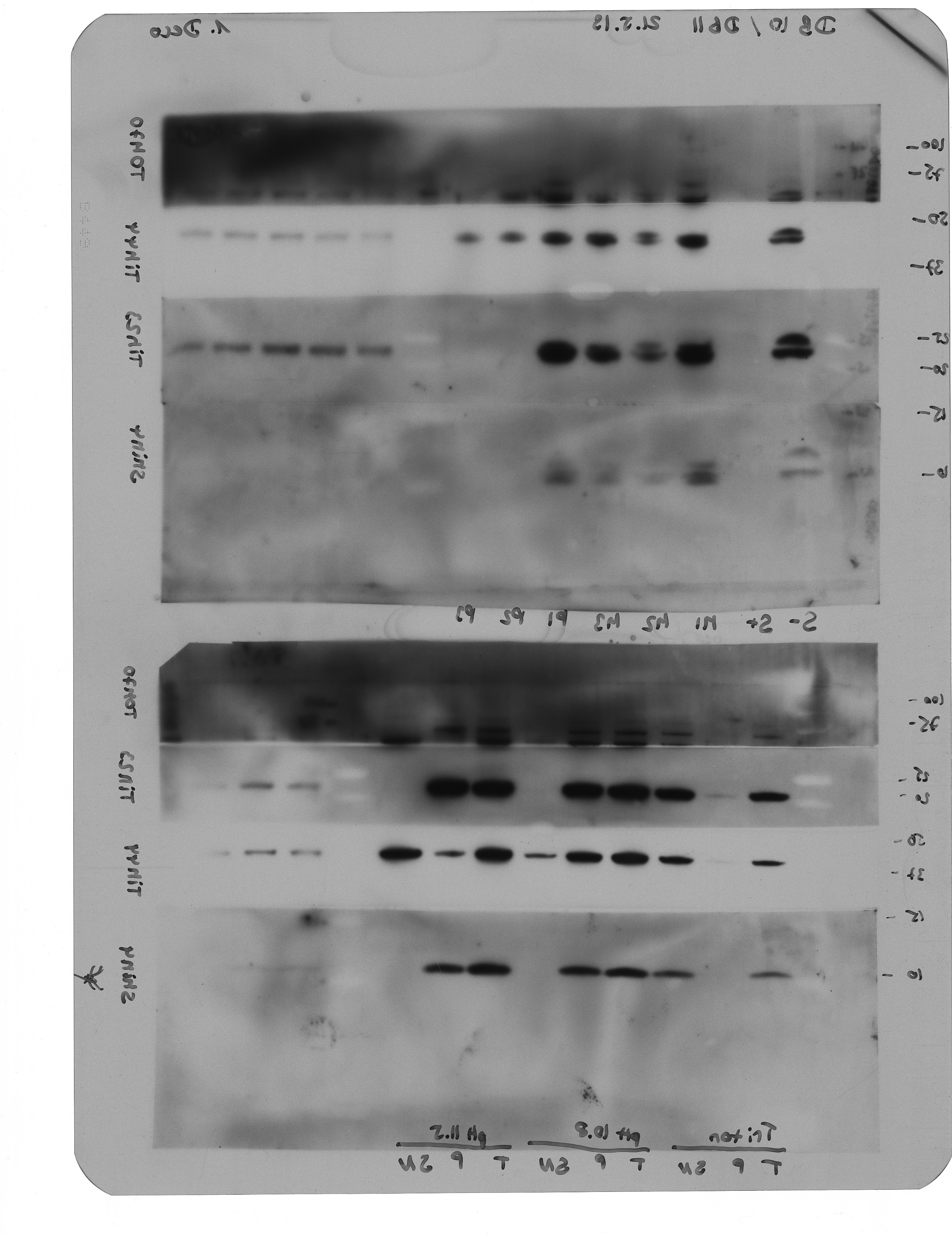

Supplement: Figure 4—source data 1. [file elife-68213-fig4-data1.zip › Figure_4_source_data/Figure_4_source_data_2_Figure_4D/Original_data/2003.jpg]

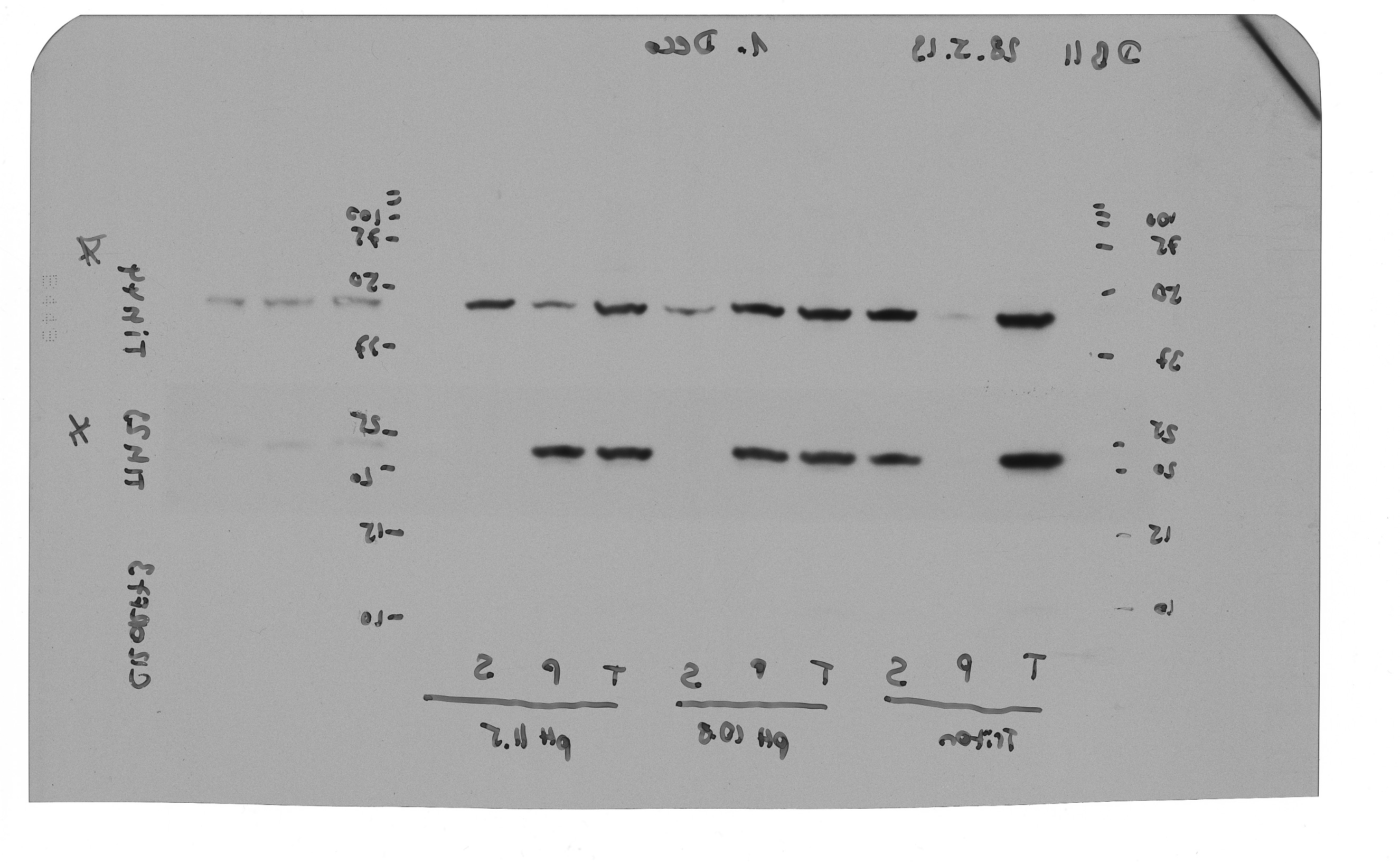

Supplement: Figure 4—source data 1. [file elife-68213-fig4-data1.zip › Figure_4_source_data/Figure_4_source_data_2_Figure_4D/Original_data/1002.jpg]

Figure 4 source data 3 related to Figure 4F

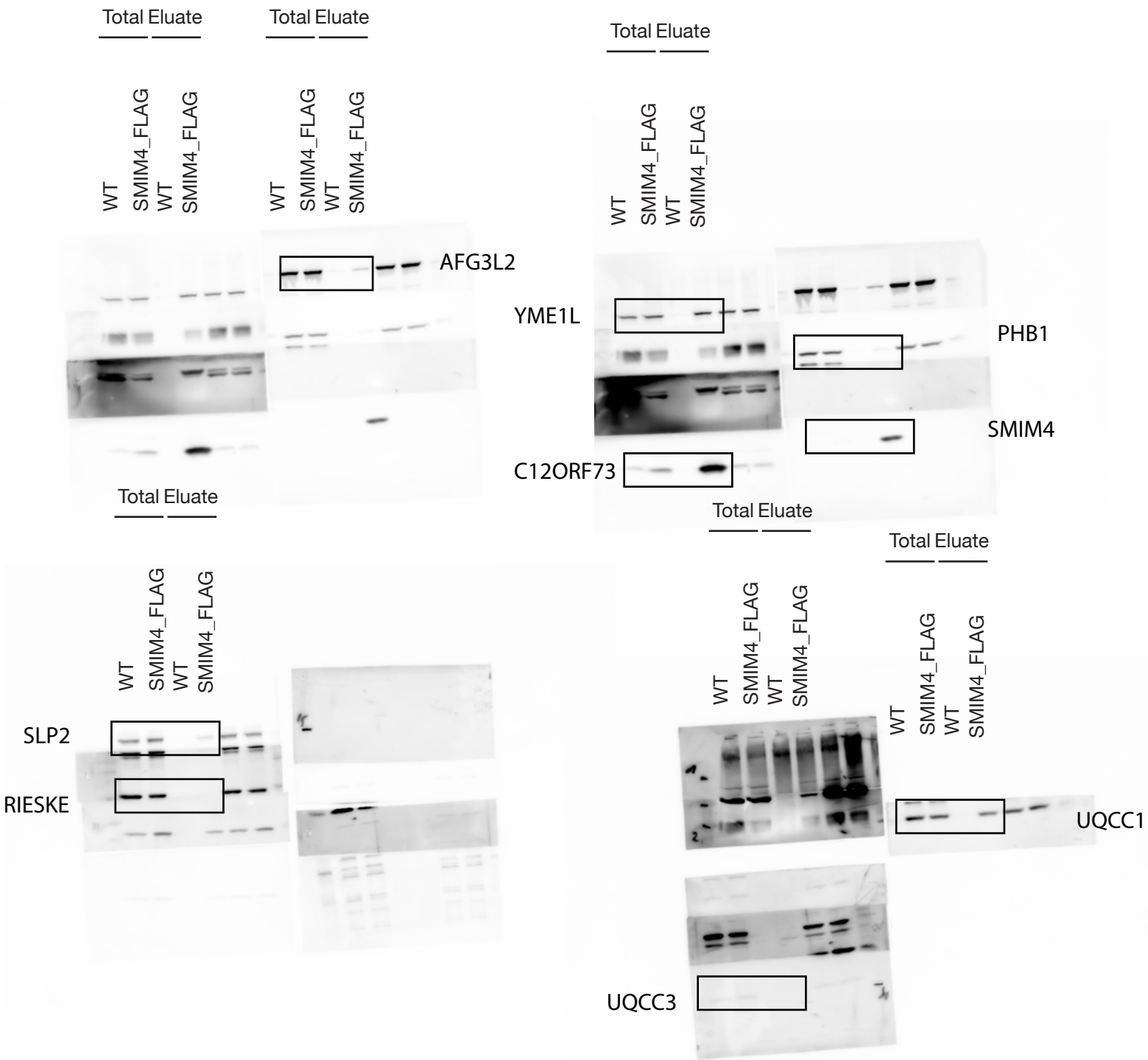

Supplement: Figure 4—source data 1. [file elife-68213-fig4-data1.zip › Figure_4_source_data/Figure_4_source_data_3_Figure_4F/Data_labelled/Figure_4_source_data_3_Figure_4F.pdf]

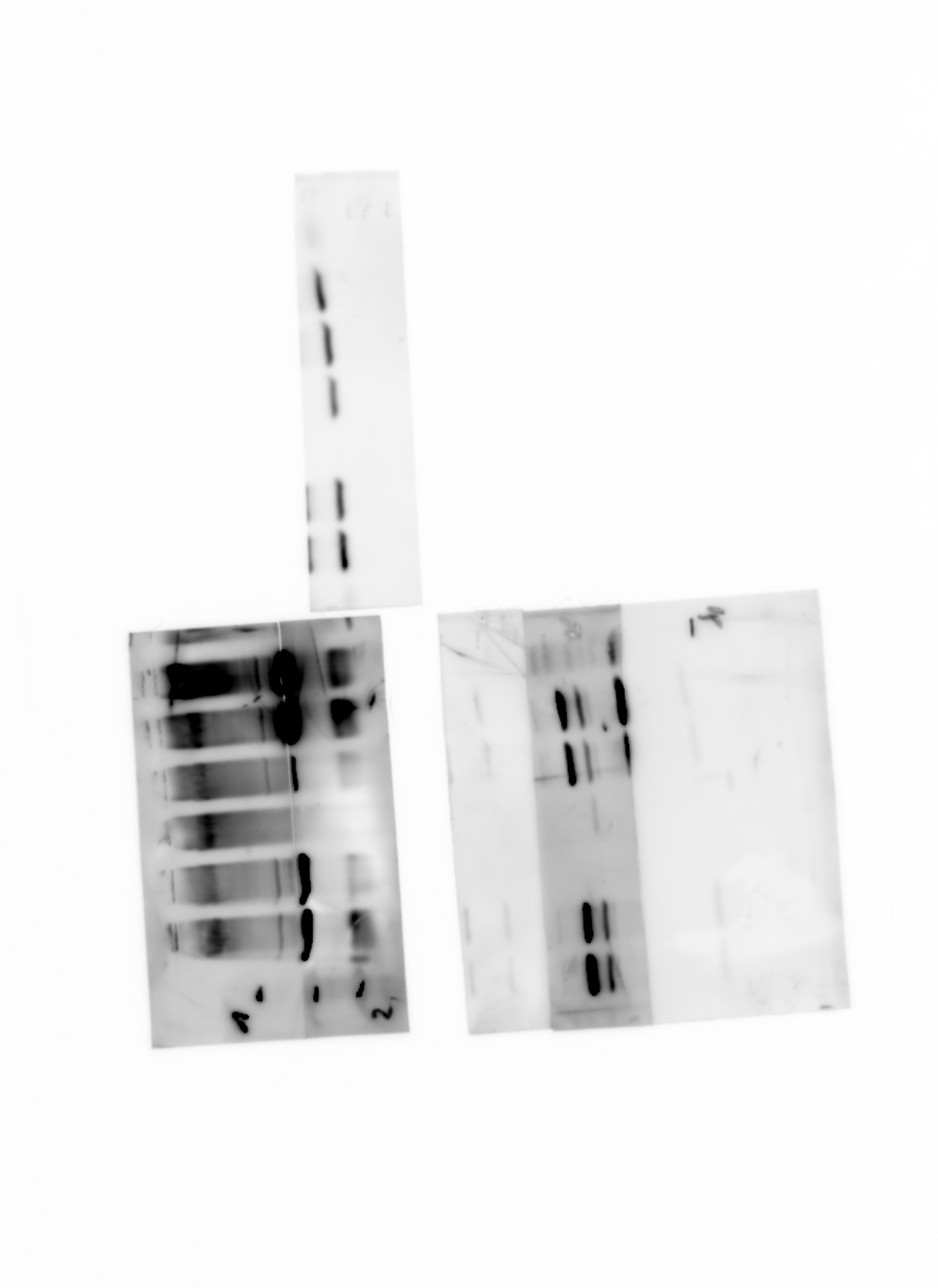

Supplement: Figure 4—source data 1. [file elife-68213-fig4-data1.zip › Figure_4_source_data/Figure_4_source_data_3_Figure_4F/Original_files/2nd expo 20201118_141821-20_Ch_Chemi.jpg]

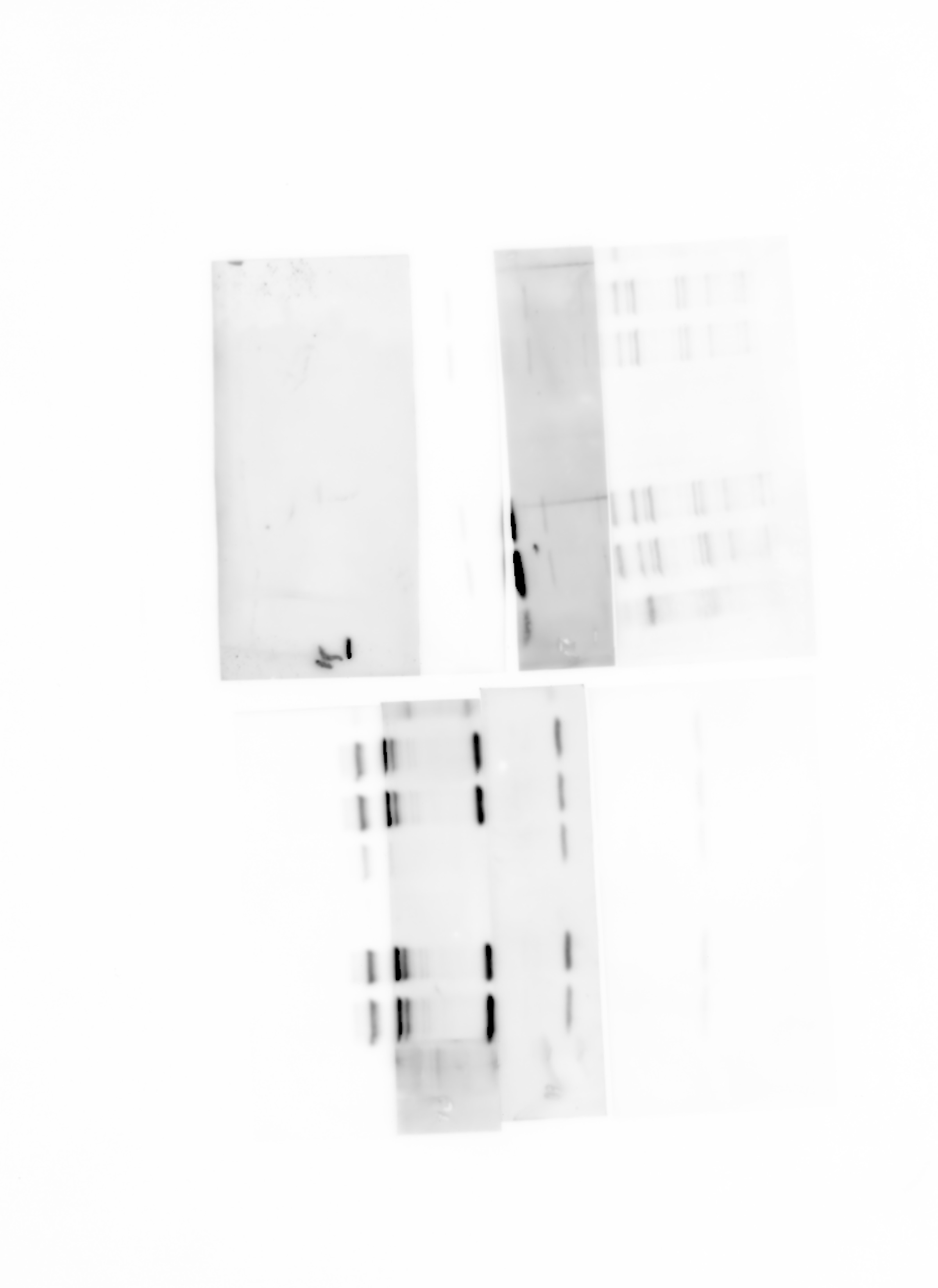

Supplement: Figure 4—source data 1. [file elife-68213-fig4-data1.zip › Figure_4_source_data/Figure_4_source_data_3_Figure_4F/Original_files/1st deko Gel 2 20201117_111001-20_Ch_Chemi.jpg]

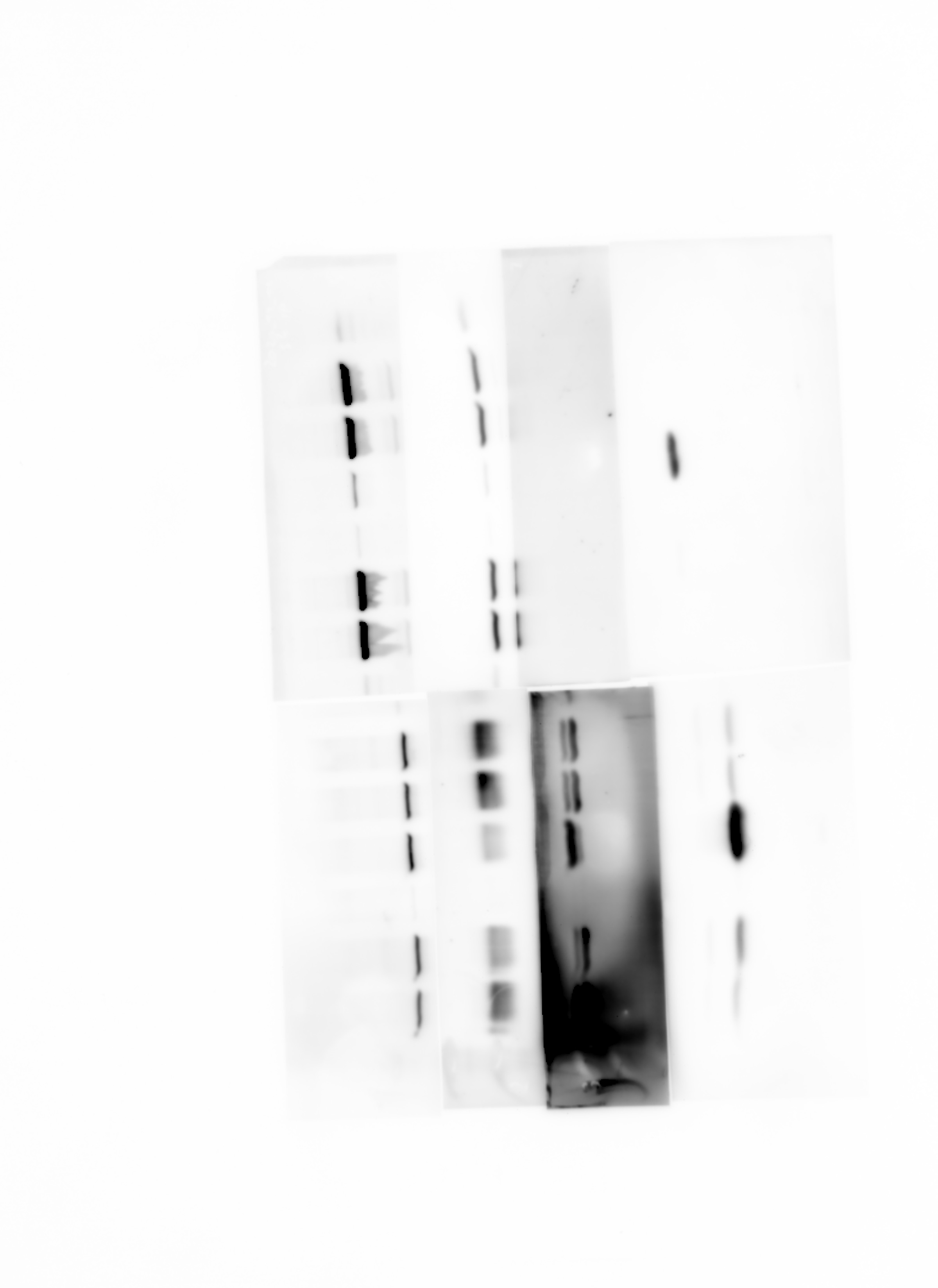

Supplement: Figure 4—source data 1. [file elife-68213-fig4-data1.zip › Figure_4_source_data/Figure_4_source_data_3_Figure_4F/Original_files/1st deko 20201117_105227-20_Ch_Chemi.jpg]

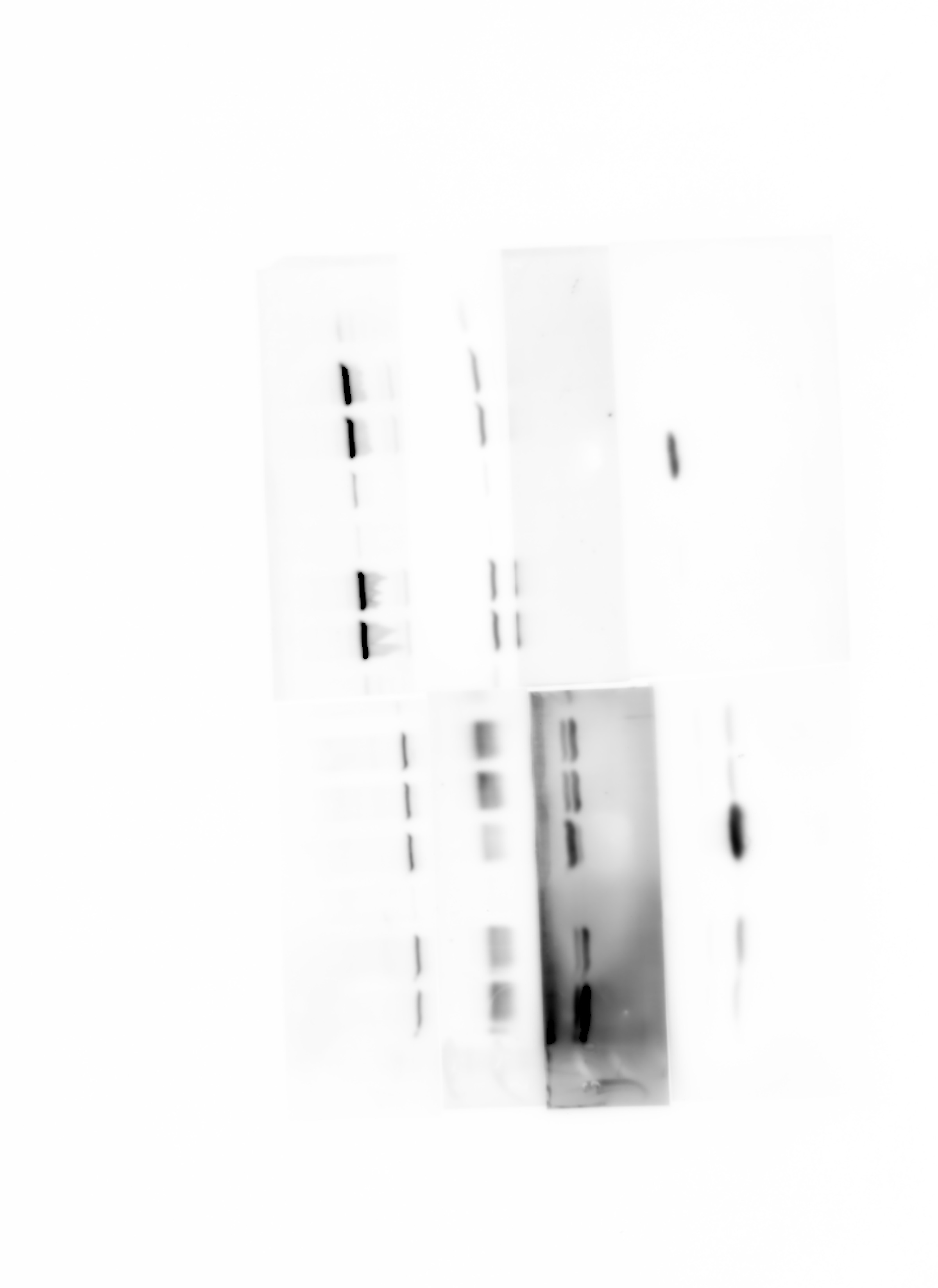

Supplement: Figure 4—source data 1. [file elife-68213-fig4-data1.zip › Figure_4_source_data/Figure_4_source_data_3_Figure_4F/Original_files/1st deko 20201117_105227-10_Ch_Chemi.jpg]

Figure 5 source data 1 related to Figure 5A

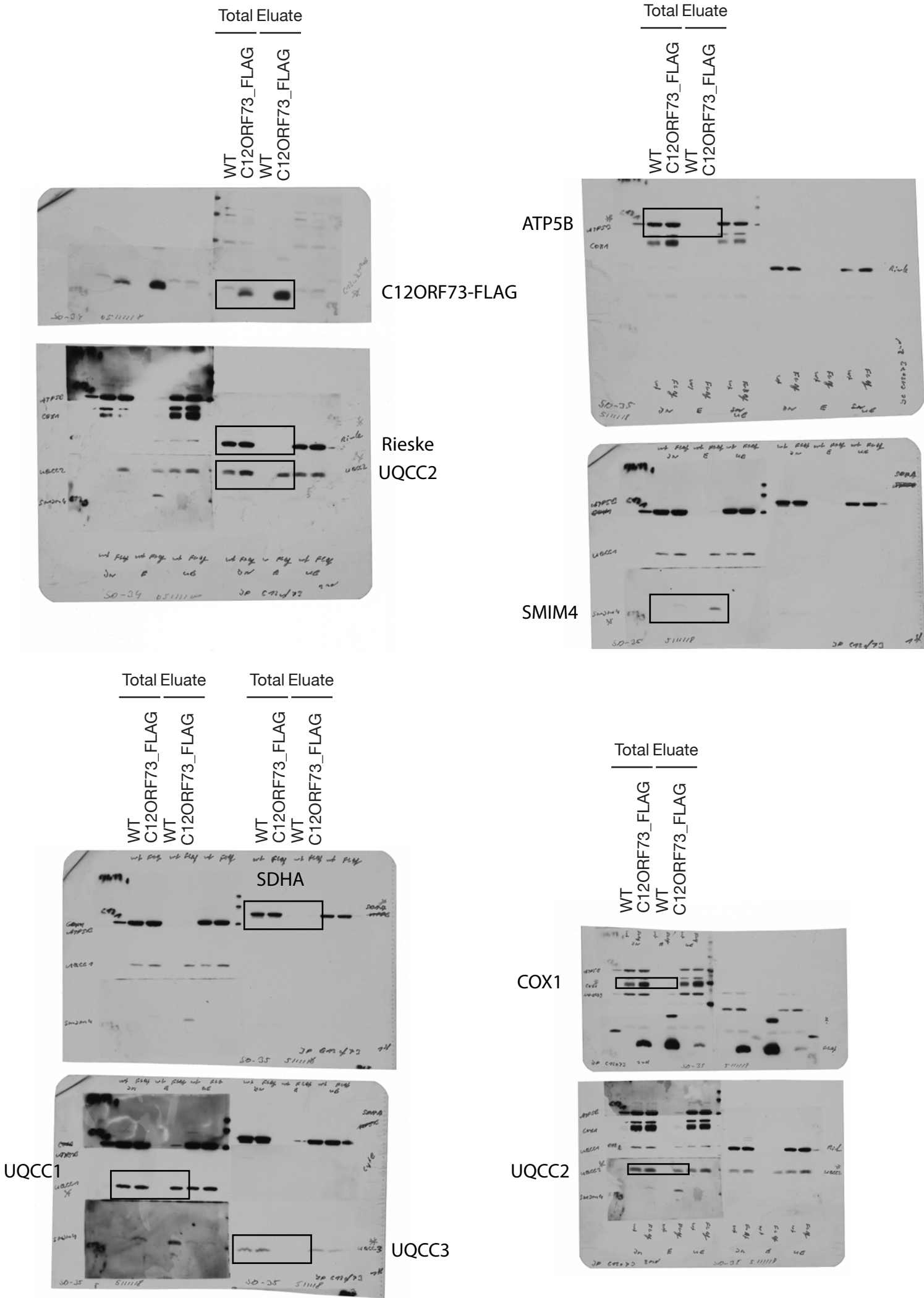

Supplement: Figure 5—source data 1. [file elife-68213-fig5-data1.zip › Figure_5_source_data/Figure_5_source_data_1_Figure_5A/Data_labelled/Figure_5_source_data_1_Figure_5A.pdf]

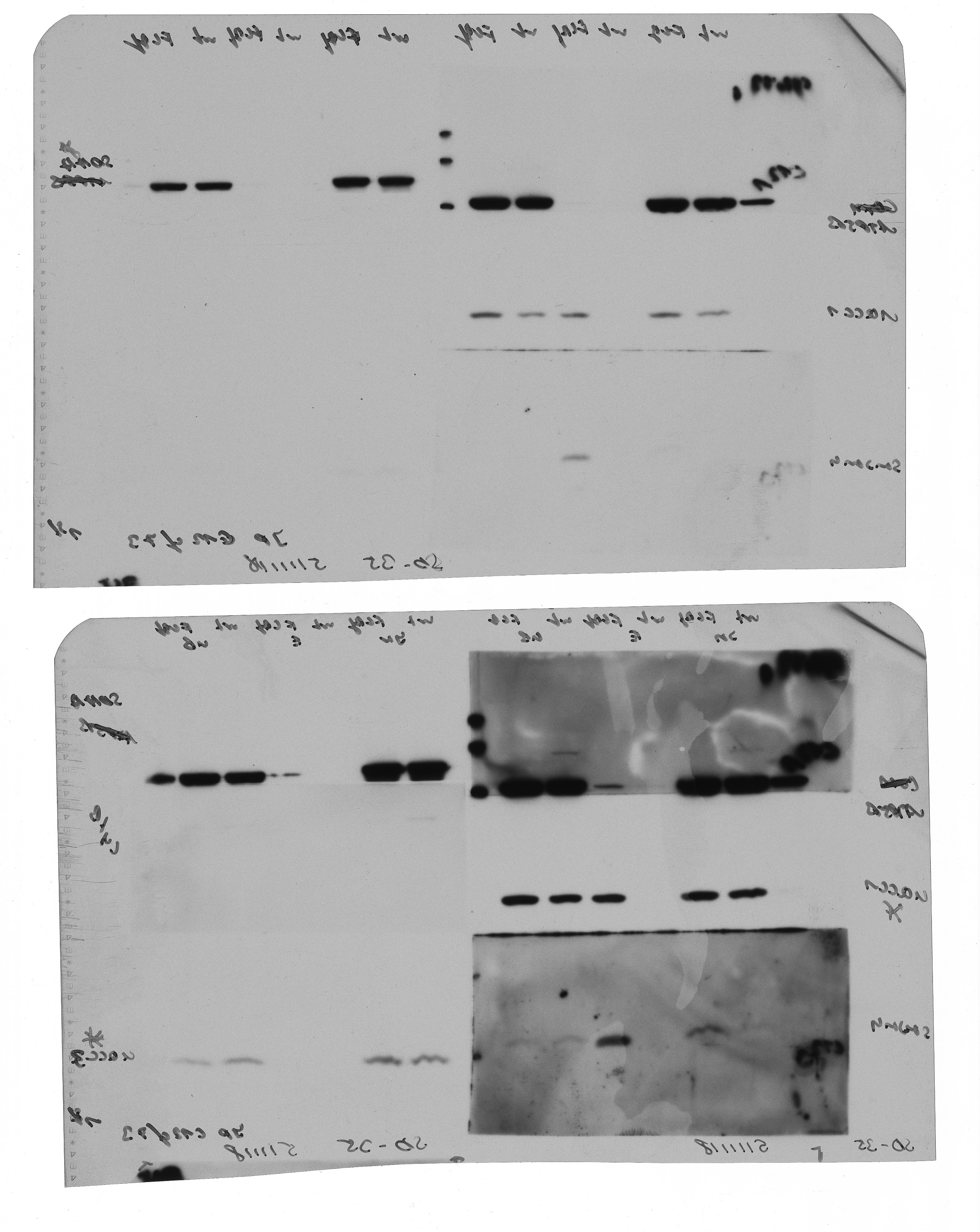

Supplement: Figure 5—source data 1. [file elife-68213-fig5-data1.zip › Figure_5_source_data/Figure_5_source_data_1_Figure_5A/Original_data/3003.jpg]

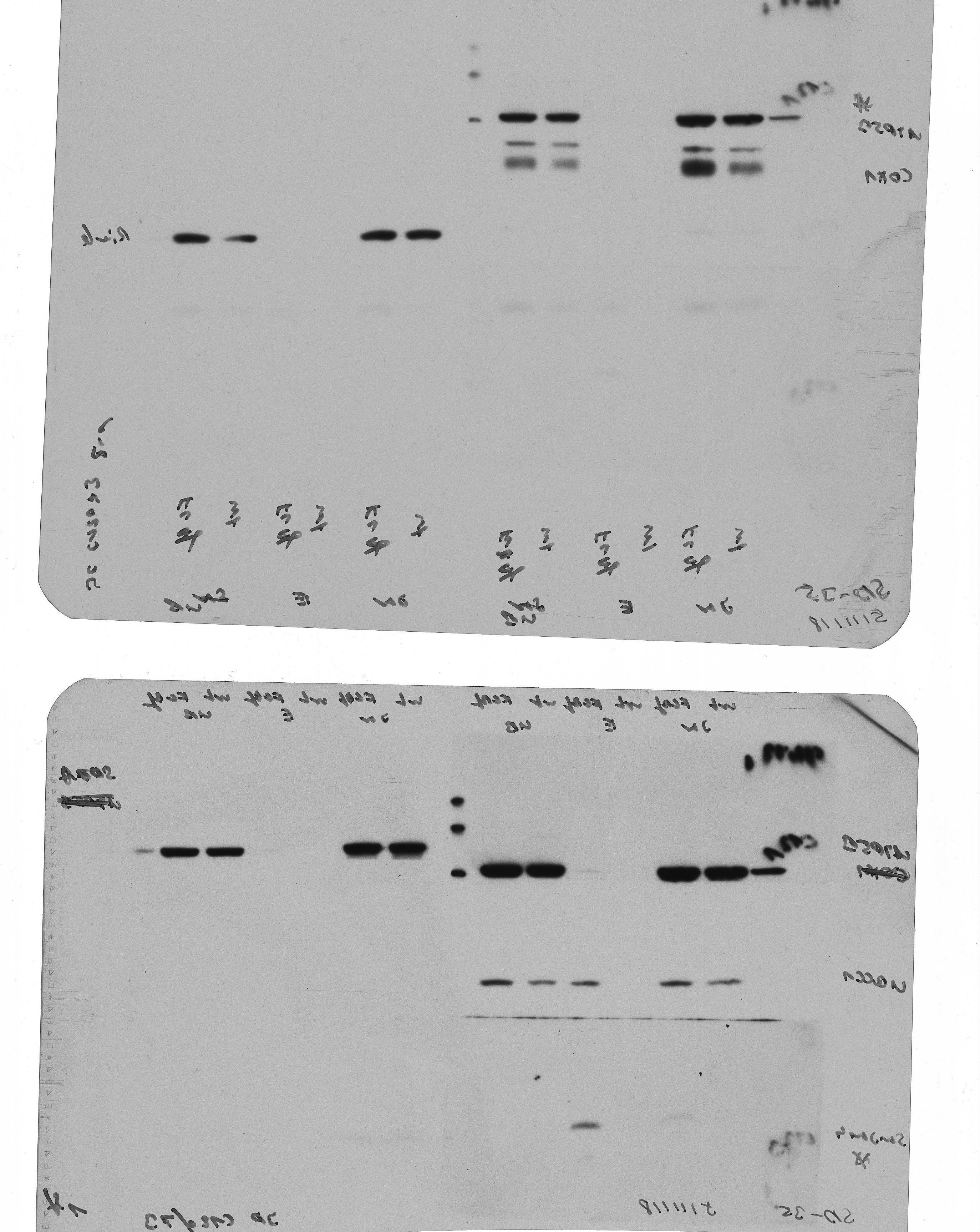

Supplement: Figure 5—source data 1. [file elife-68213-fig5-data1.zip › Figure_5_source_data/Figure_5_source_data_1_Figure_5A/Original_data/2002.jpg]

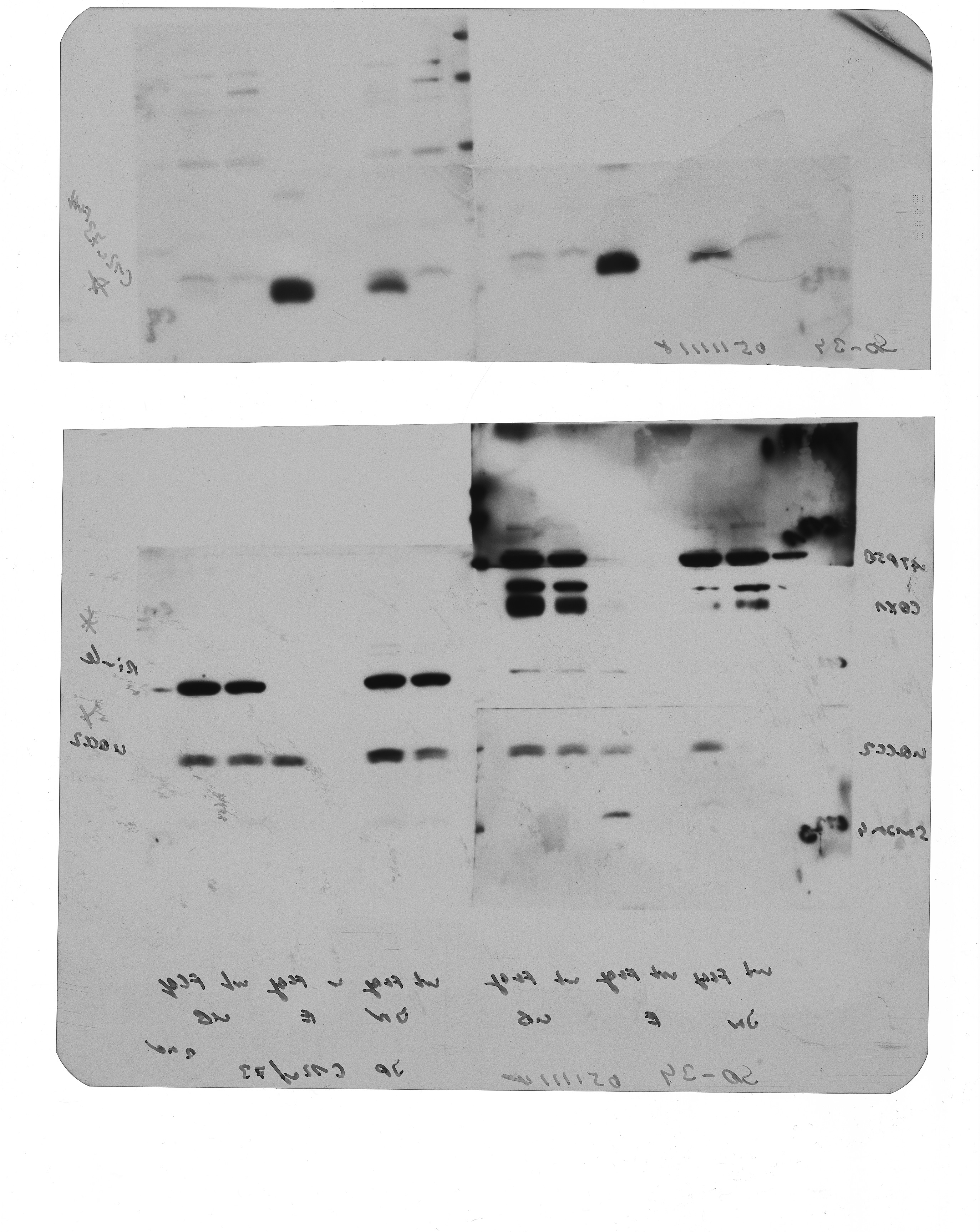

Supplement: Figure 5—source data 1. [file elife-68213-fig5-data1.zip › Figure_5_source_data/Figure_5_source_data_1_Figure_5A/Original_data/1001.jpg]

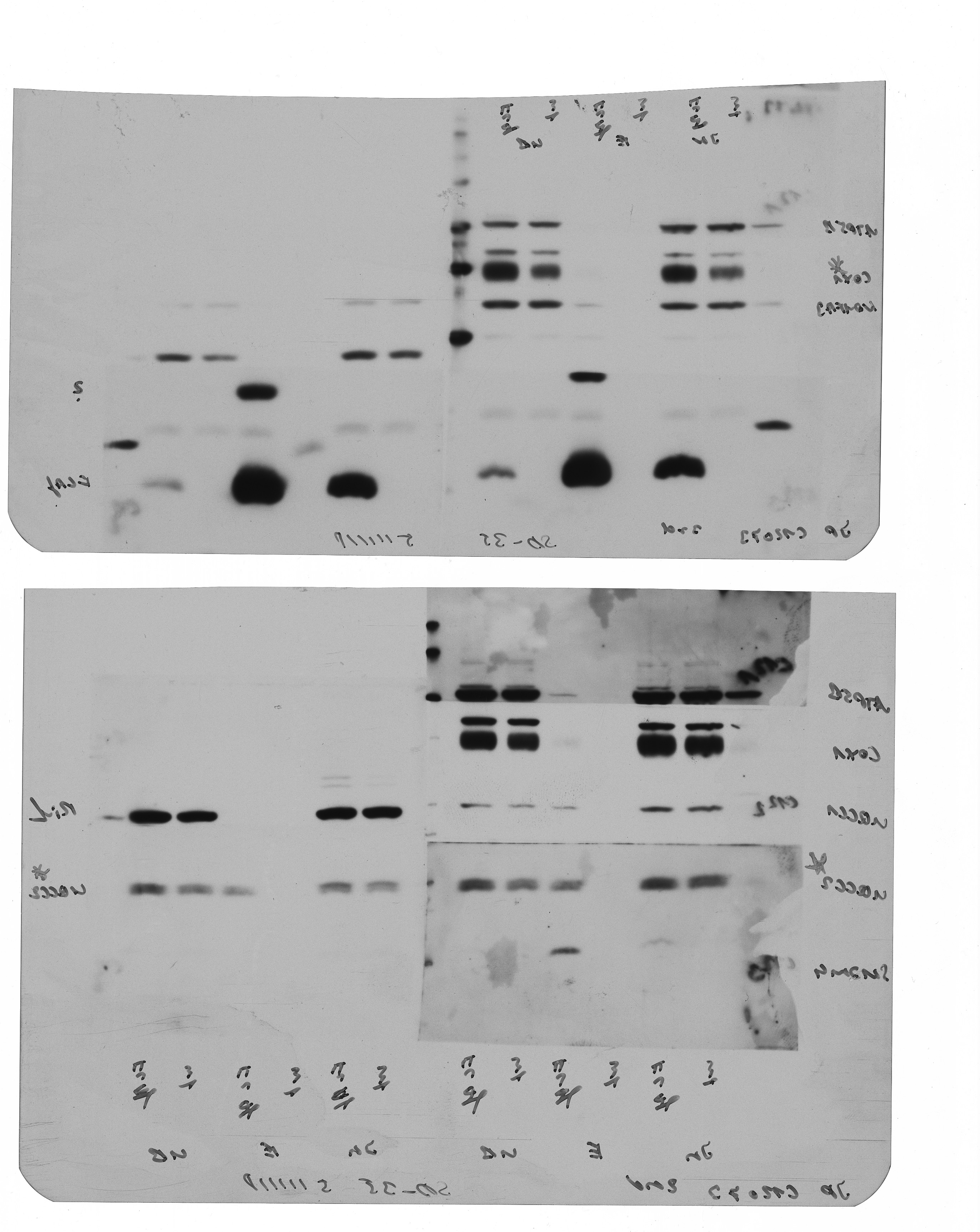

Supplement: Figure 5—source data 1. [file elife-68213-fig5-data1.zip › Figure_5_source_data/Figure_5_source_data_1_Figure_5A/Original_data/4004.jpg]

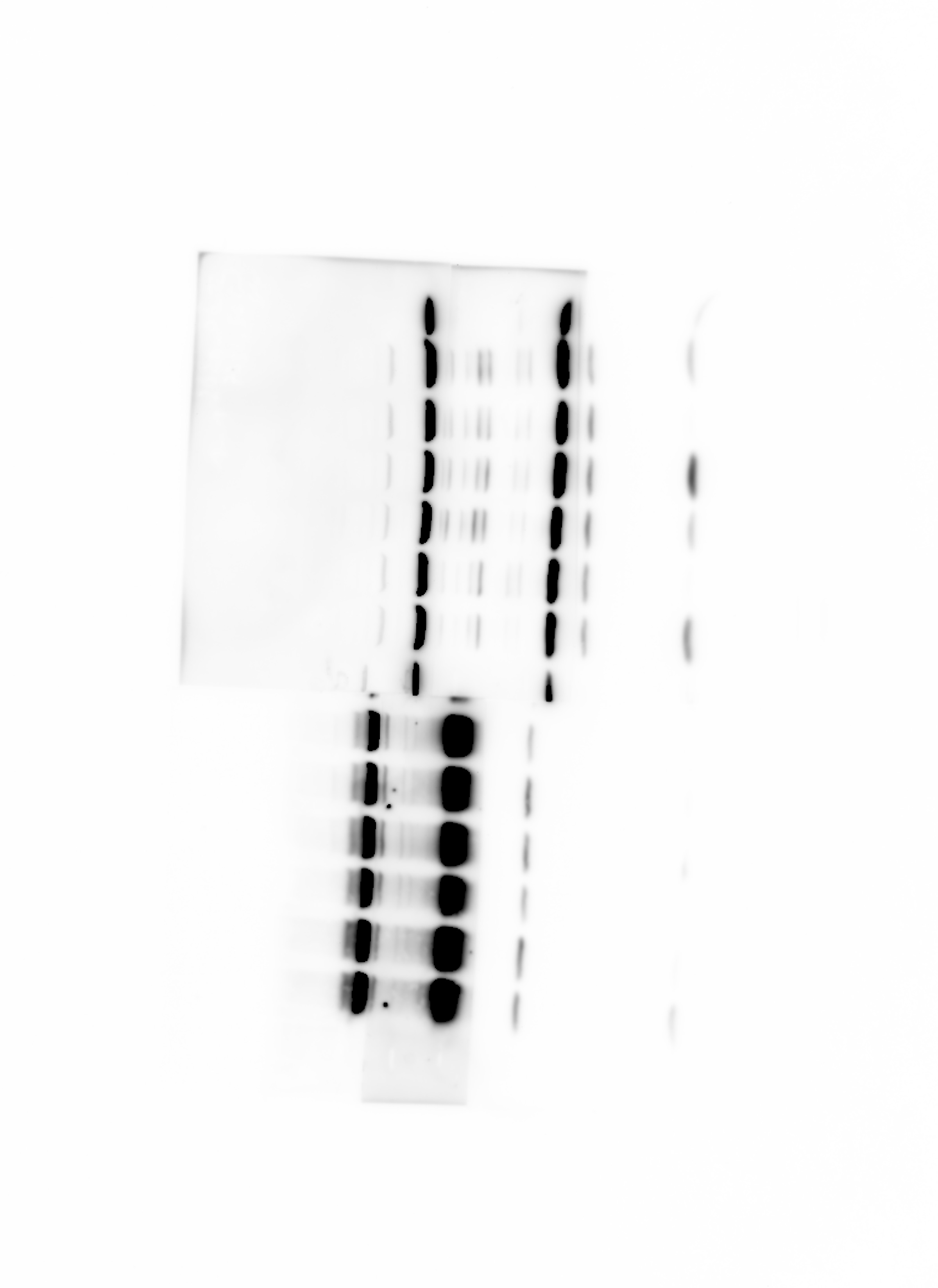

Supplement: Figure 5—source data 1. [file elife-68213-fig5-data1.zip › Figure_5_source_data/Figure_5_source_data_2_Figure_5B/Original_files/SD_354_Gel1_expo_1 20200616_111449-17_Ch_Chemi.jpg]

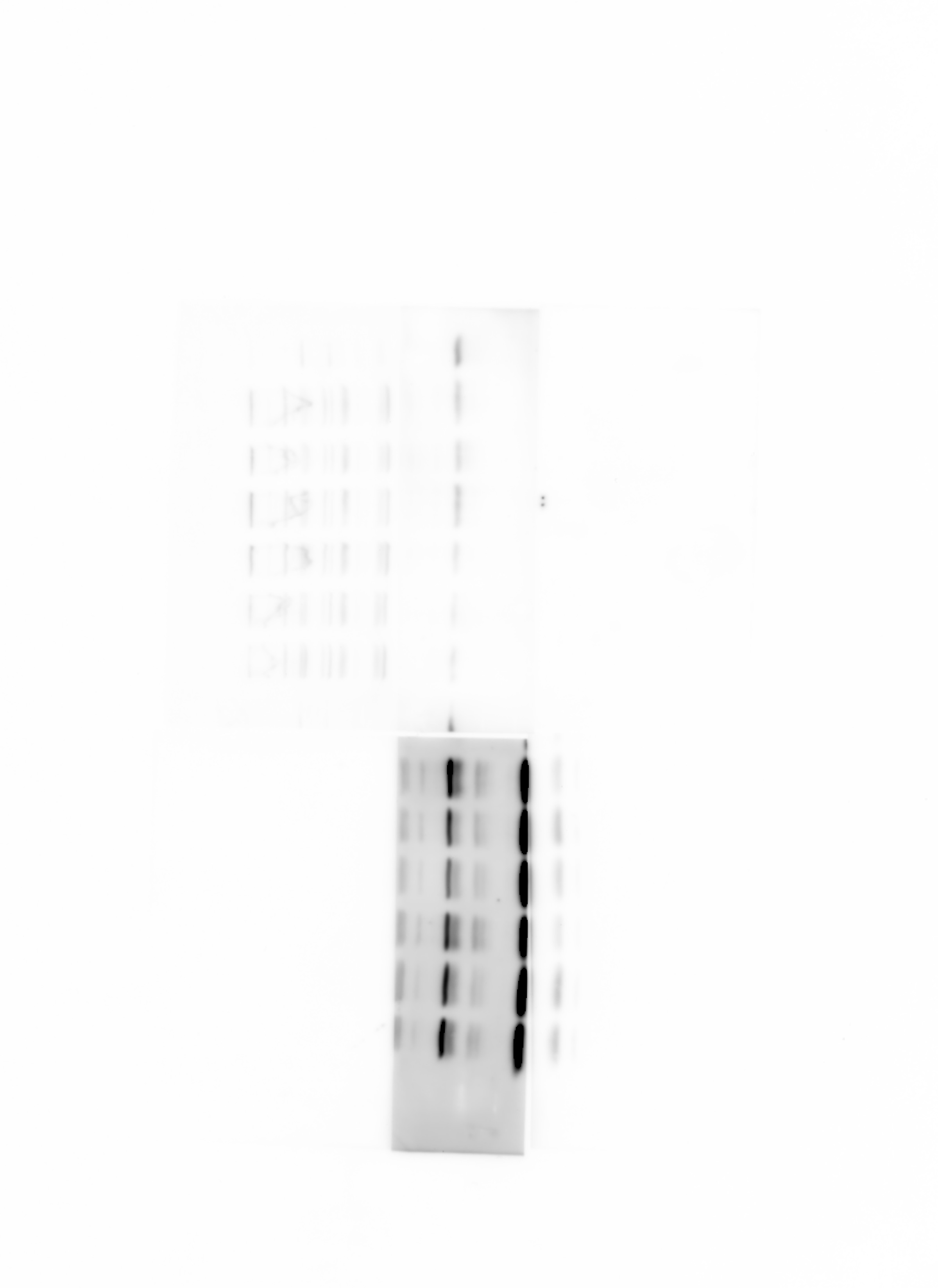

Supplement: Figure 5—source data 1. [file elife-68213-fig5-data1.zip › Figure_5_source_data/Figure_5_source_data_2_Figure_5B/Original_files/SD_354_Gel2_expo_1 20200616_112522-20_Ch_Chemi.jpg]

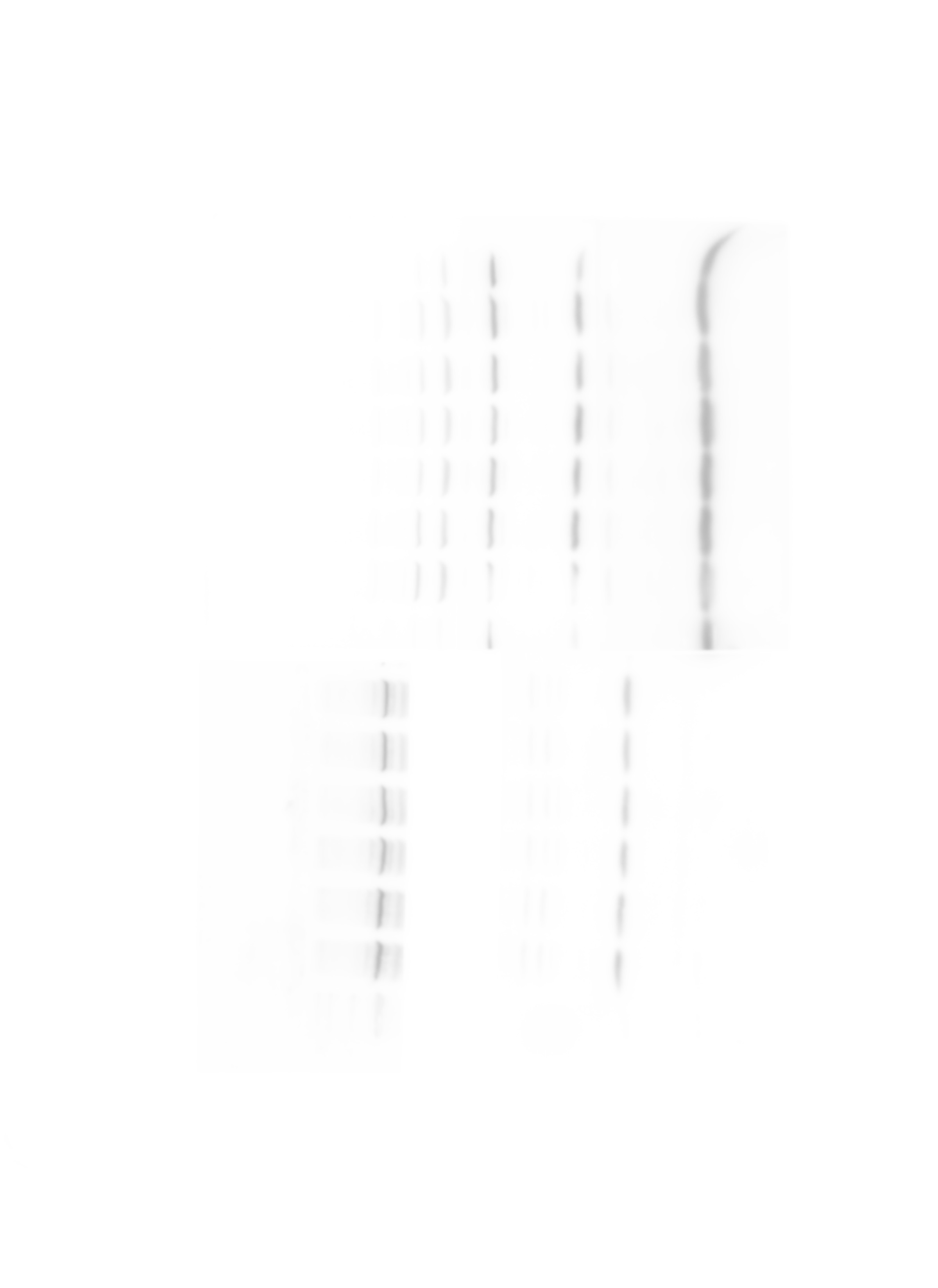

Supplement: Figure 5—source data 1. [file elife-68213-fig5-data1.zip › Figure_5_source_data/Figure_5_source_data_2_Figure_5B/Original_files/SD_355_Gel_1_expo_2 20200617_111331-01_Ch_Chemi.jpg]

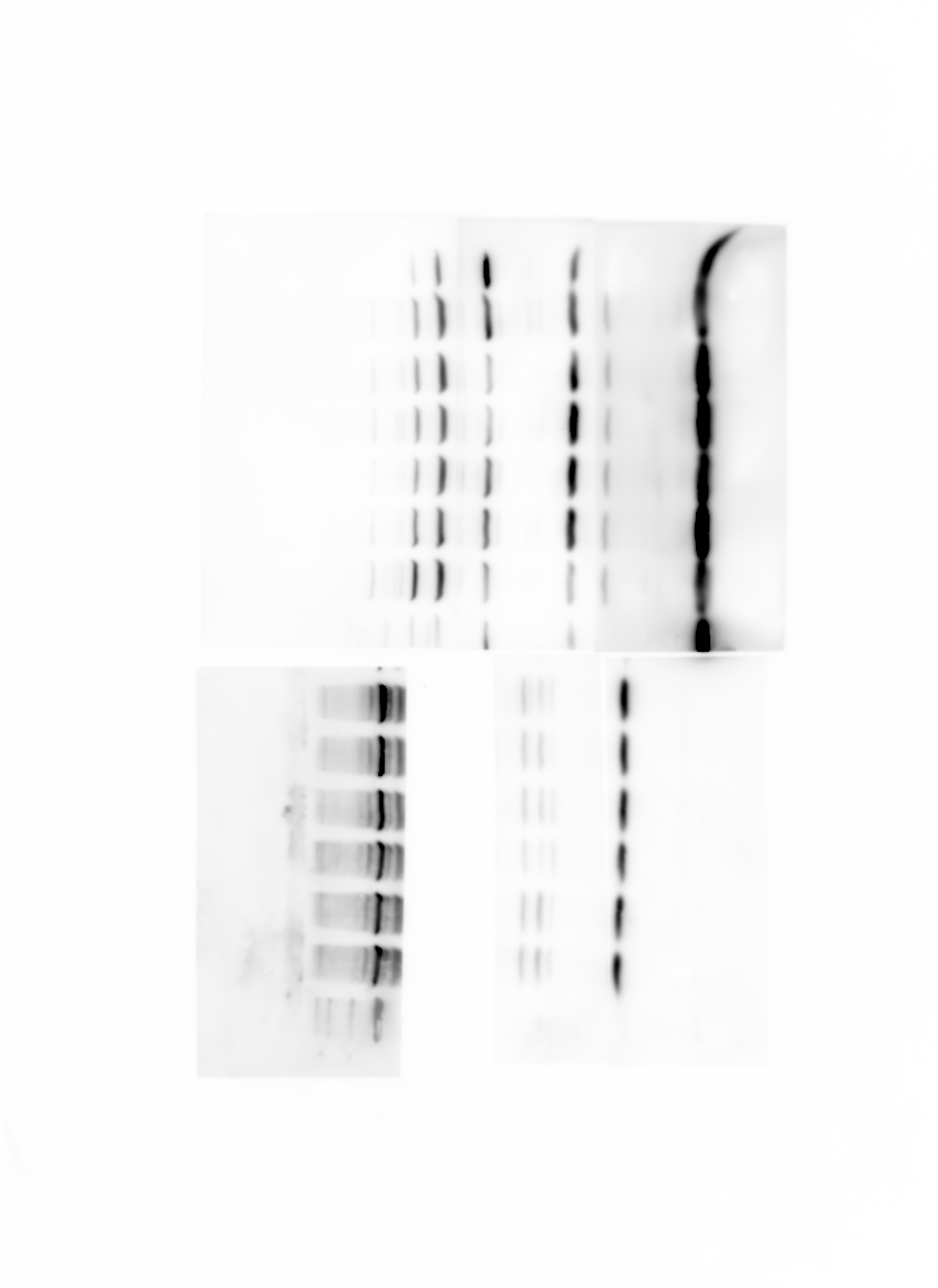

Supplement: Figure 5—source data 1. [file elife-68213-fig5-data1.zip › Figure_5_source_data/Figure_5_source_data_2_Figure_5B/Original_files/SD_355_Gel_1_expo_2_zusatz 20200617_112009-12_Ch_Chemi.jpg]

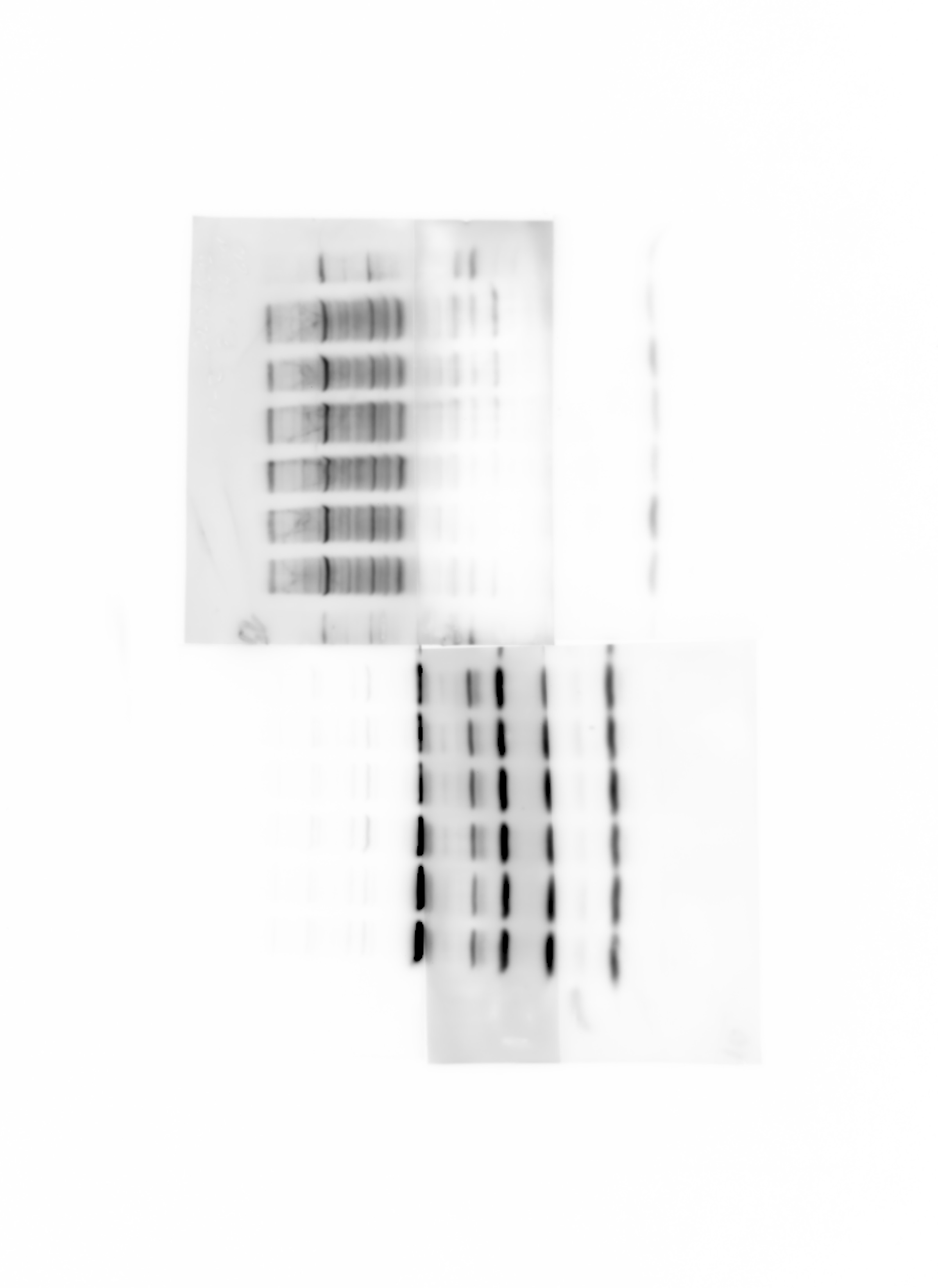

Supplement: Figure 5—source data 1. [file elife-68213-fig5-data1.zip › Figure_5_source_data/Figure_5_source_data_2_Figure_5B/Original_files/SD_355_Gel_2_expo_2 20200617_112837-09_Ch_Chemi.jpg]

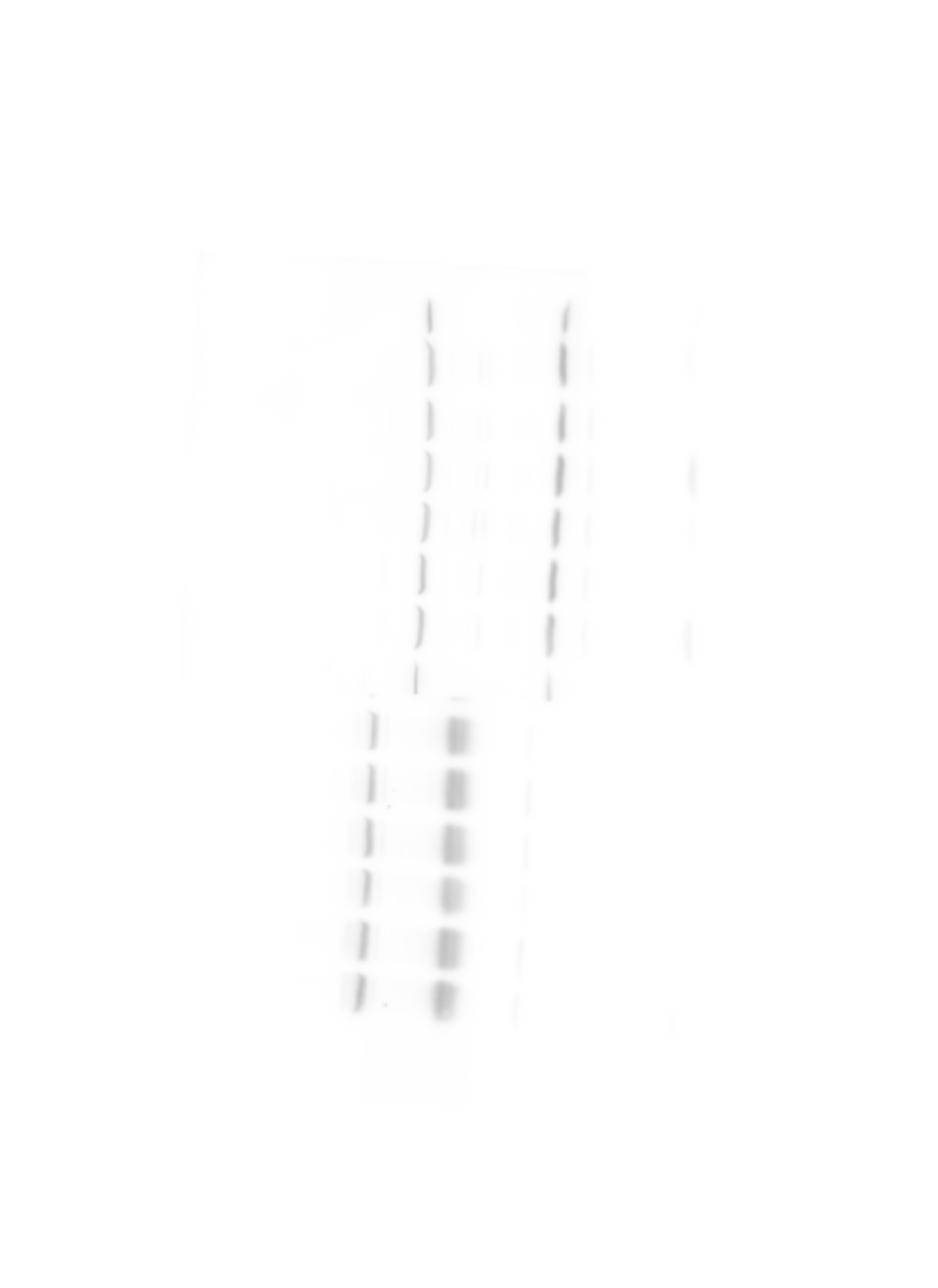

Supplement: Figure 5—source data 1. [file elife-68213-fig5-data1.zip › Figure_5_source_data/Figure_5_source_data_2_Figure_5B/Original_files/SD_354_Gel1_expo_1 20200616_111449-01_Ch_Chemi.jpg]

Figure\_5\_source\_data\_4\_Figure\_5E

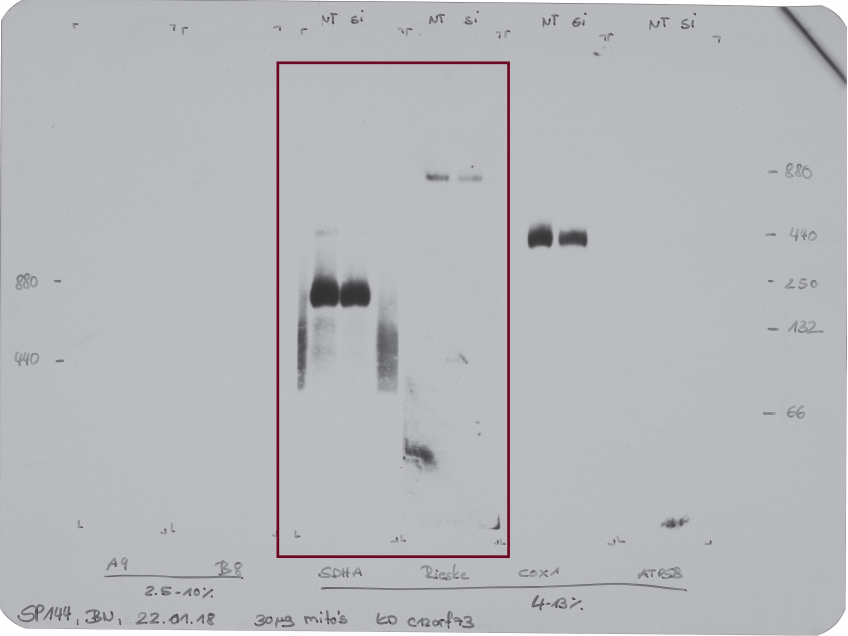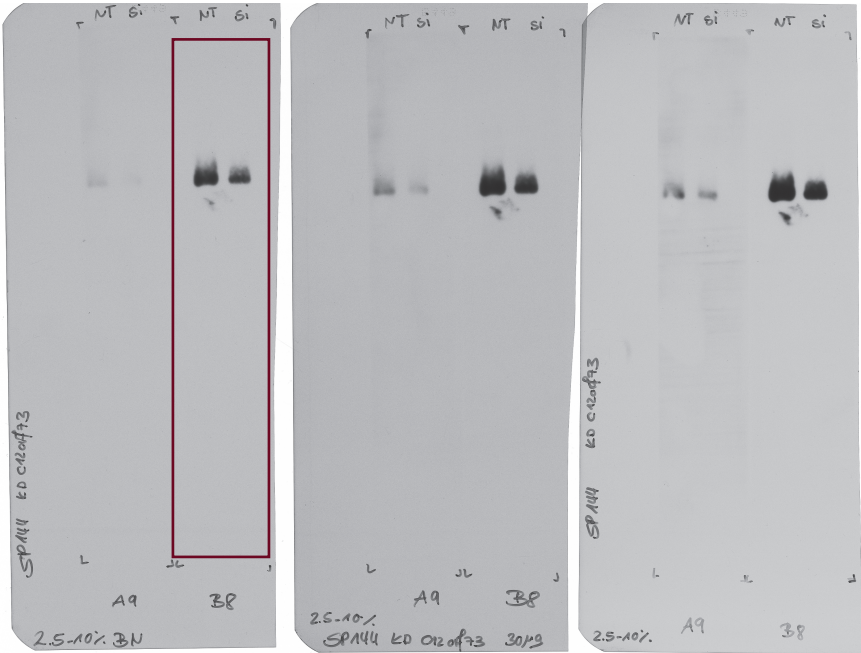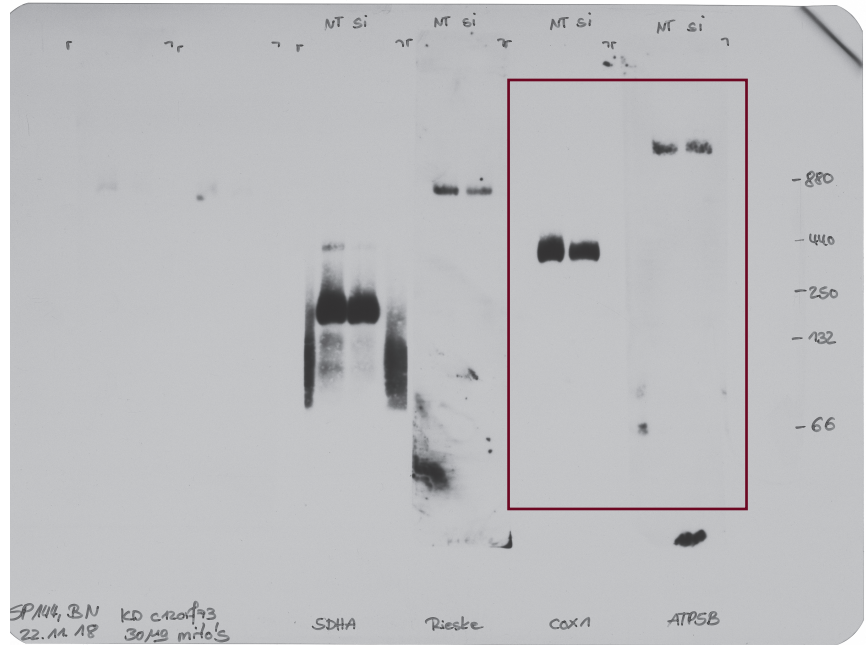

Supplement: Figure 5—source data 1. [file elife-68213-fig5-data1.zip › Figure_5_source_data/Figure_5_source_data_4_Figure_5E/Data_labelled/Figure_5_source_data_4_Figure_5E.pdf]

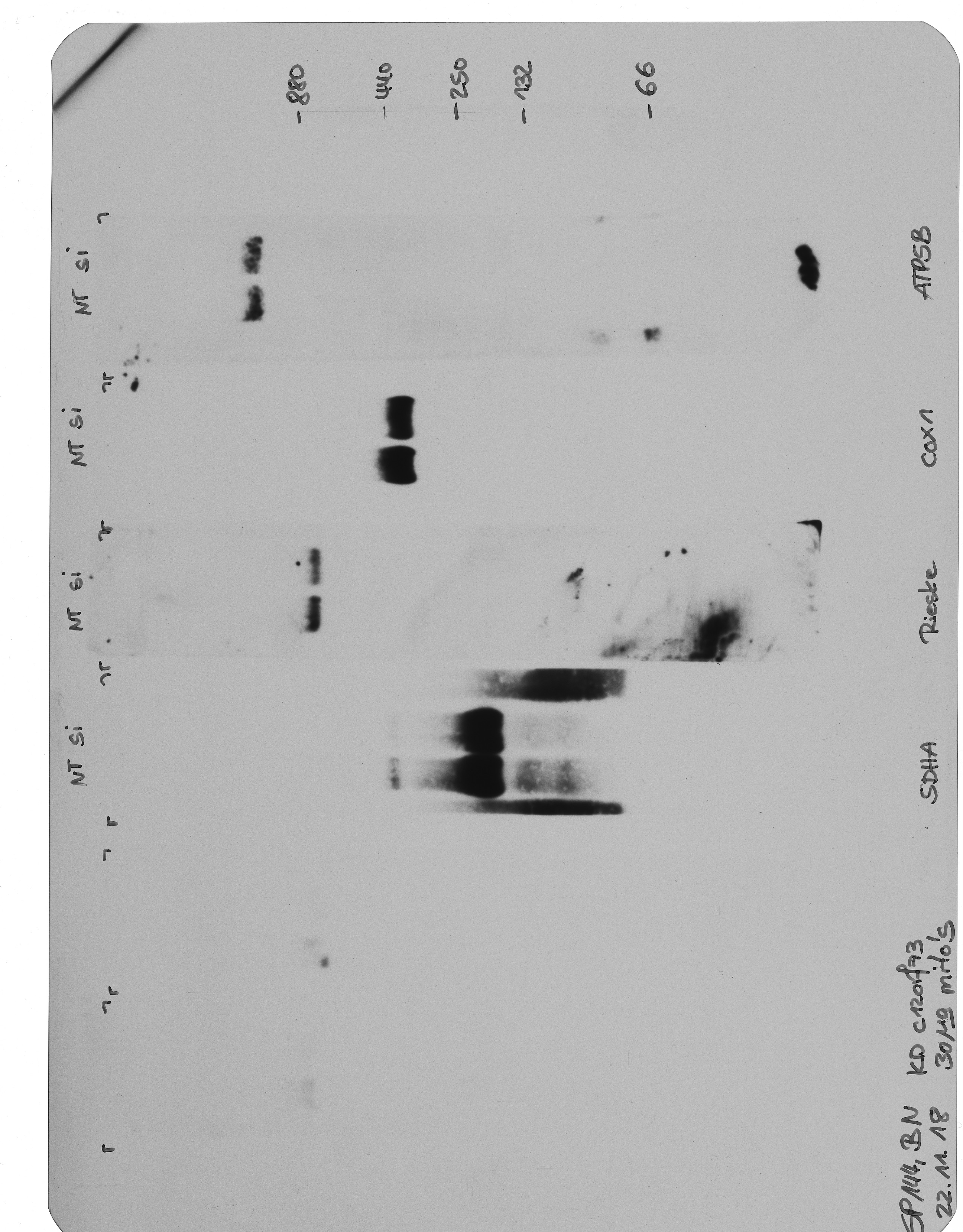

Supplement: Figure 5—source data 1. [file elife-68213-fig5-data1.zip › Figure_5_source_data/Figure_5_source_data_4_Figure_5E/Original_files/SP144_3.jpg]

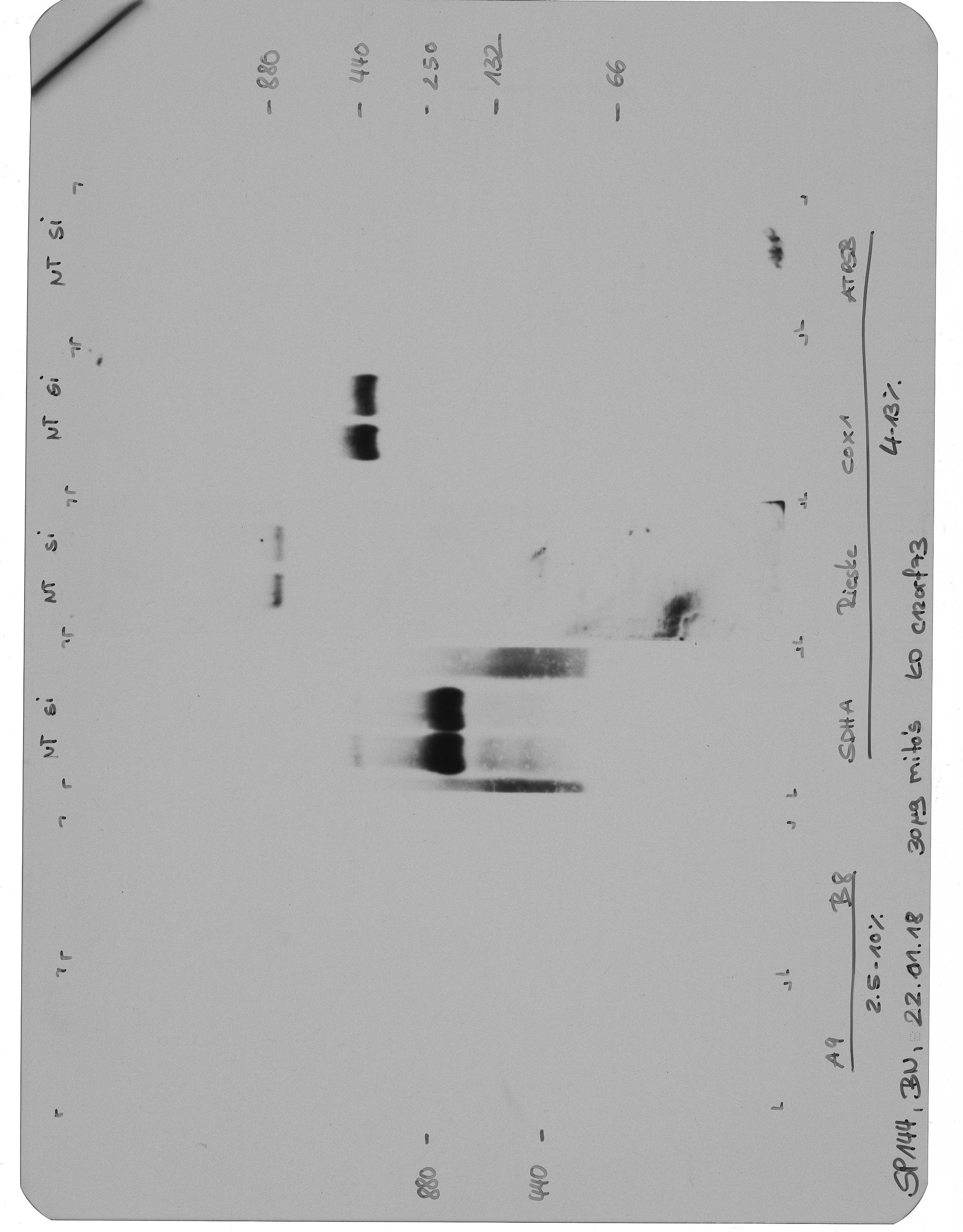

Supplement: Figure 5—source data 1. [file elife-68213-fig5-data1.zip › Figure_5_source_data/Figure_5_source_data_4_Figure_5E/Original_files/SP144_2.jpg]

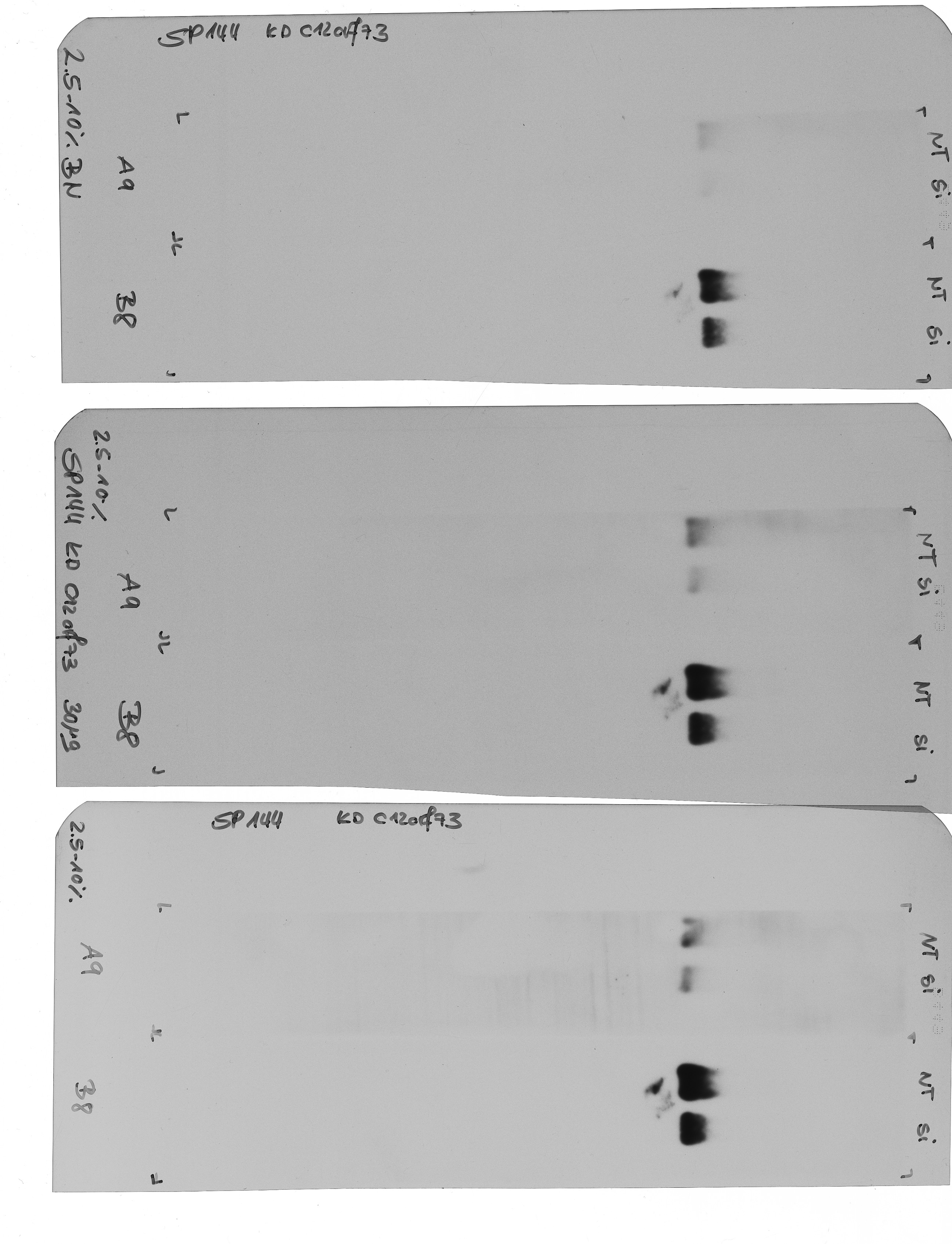

Supplement: Figure 5—source data 1. [file elife-68213-fig5-data1.zip › Figure_5_source_data/Figure_5_source_data_4_Figure_5E/Original_files/SP144_1.jpg]

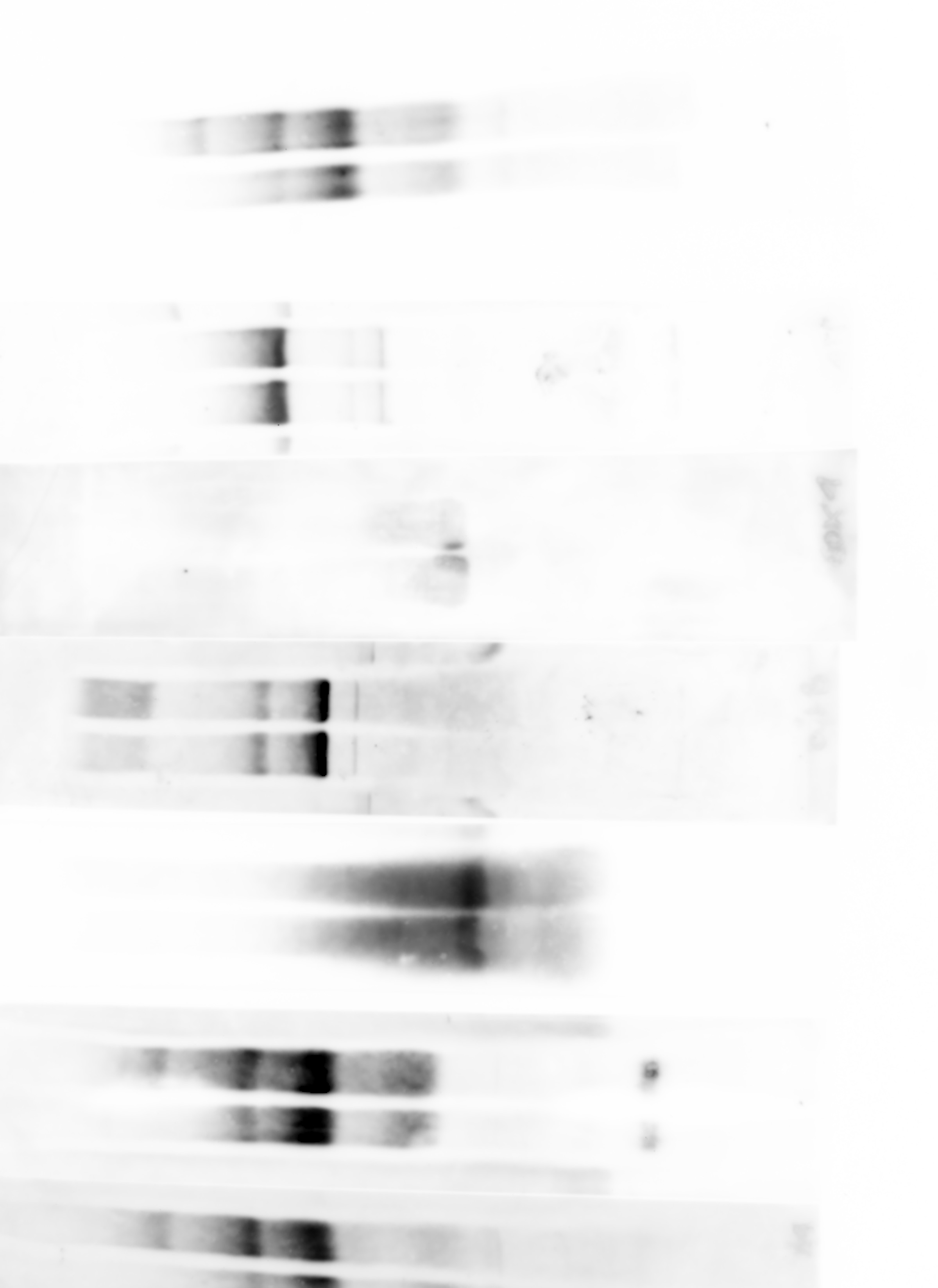

Supplement: Figure 5—source data 1. [file elife-68213-fig5-data1.zip › Figure_5_source_data/Figure_5_source_data_3_Figure_5D/Original_files/KD SMIM4, BN for revision 20210616_112812-07_Ch_Chemi.jpg]

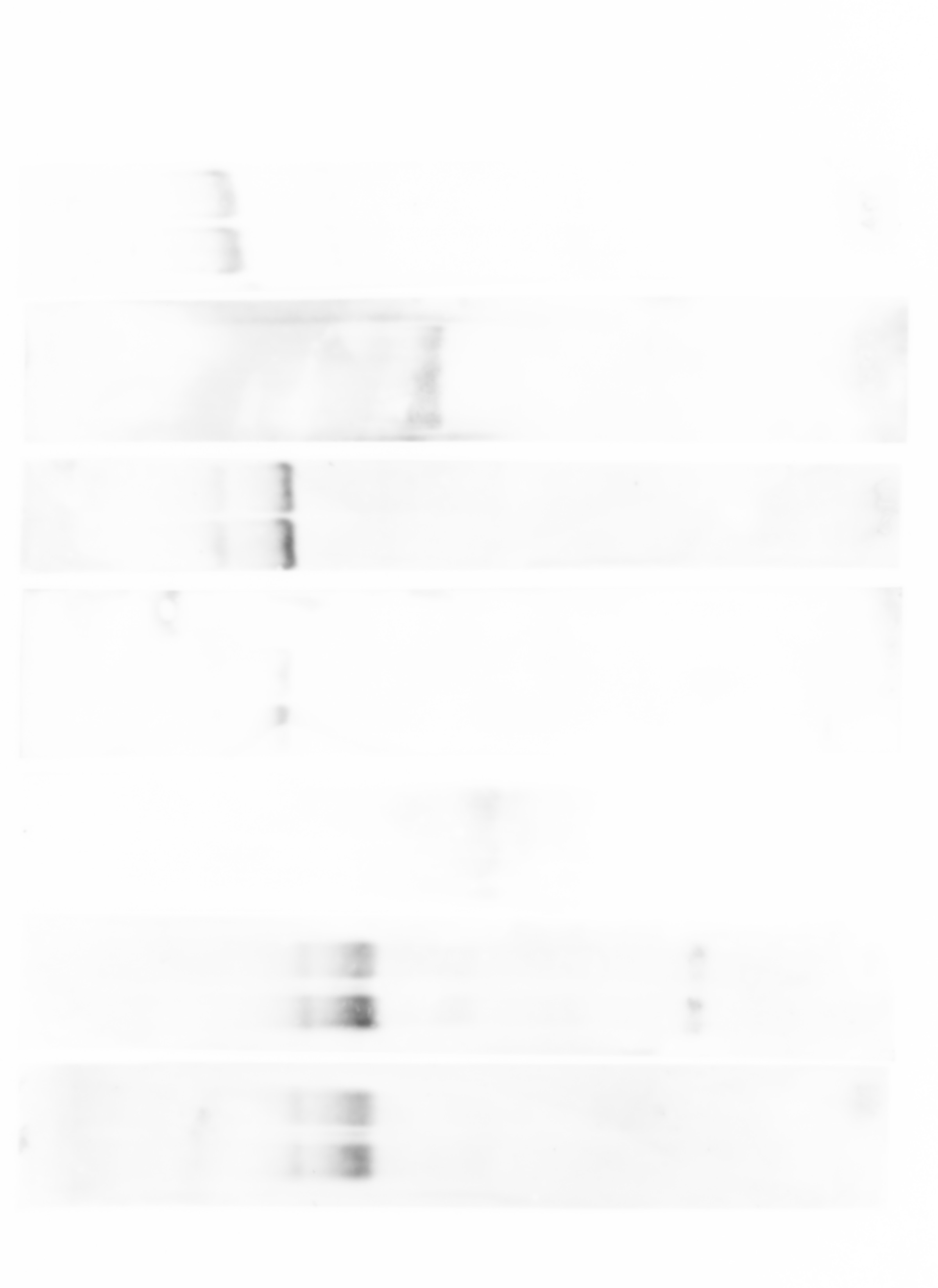

Supplement: Figure 5—source data 1. [file elife-68213-fig5-data1.zip › Figure_5_source_data/Figure_5_source_data_3_Figure_5D/Original_files/Revision KD SMIM4 BN 20210714_152815-12_Ch_Chemi.jpg]

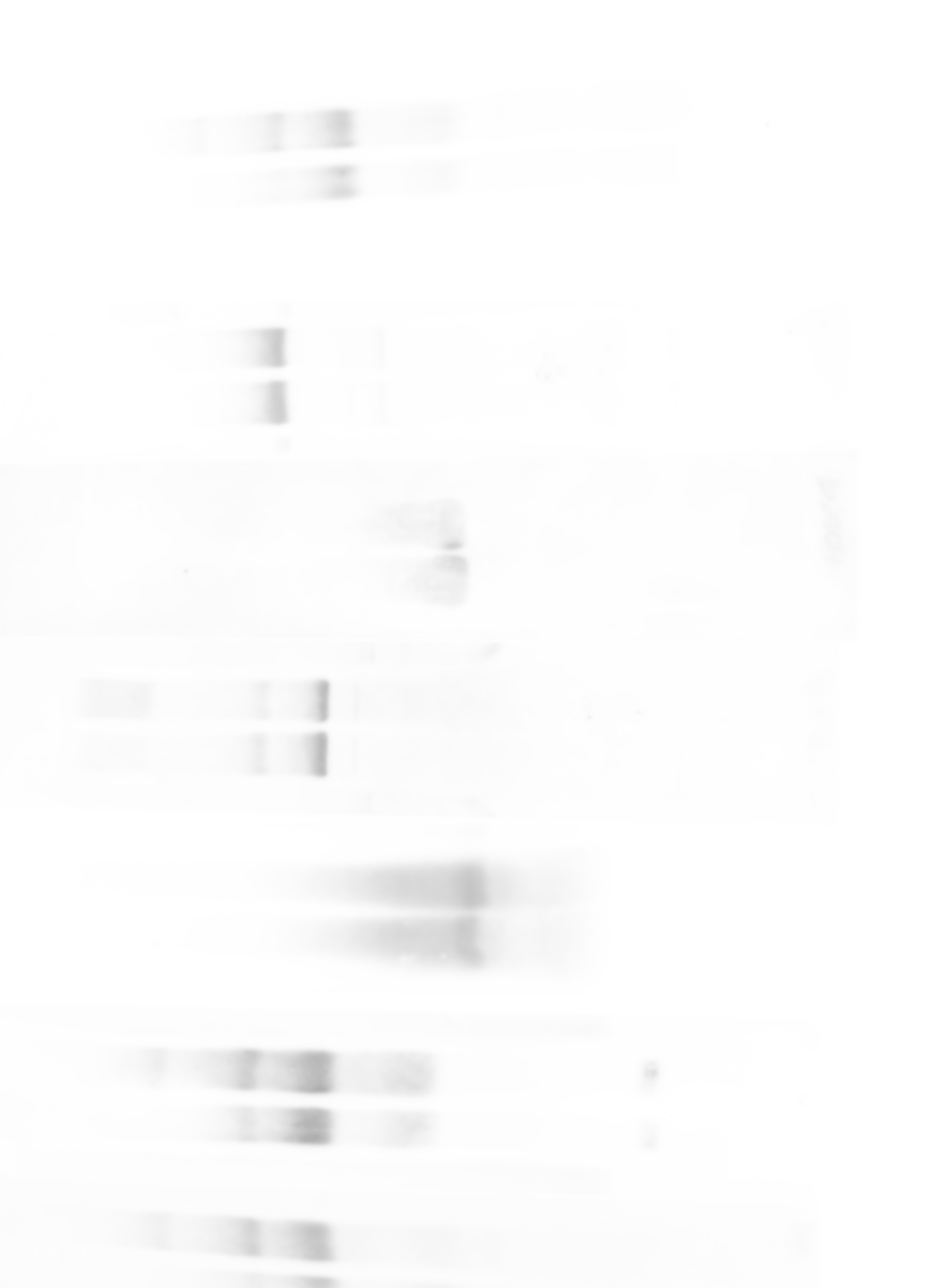

Supplement: Figure 5—source data 1. [file elife-68213-fig5-data1.zip › Figure_5_source_data/Figure_5_source_data_3_Figure_5D/Original_files/KD SMIM4, BN for revision 20210616_112812-01_Ch_Chemi.jpg]

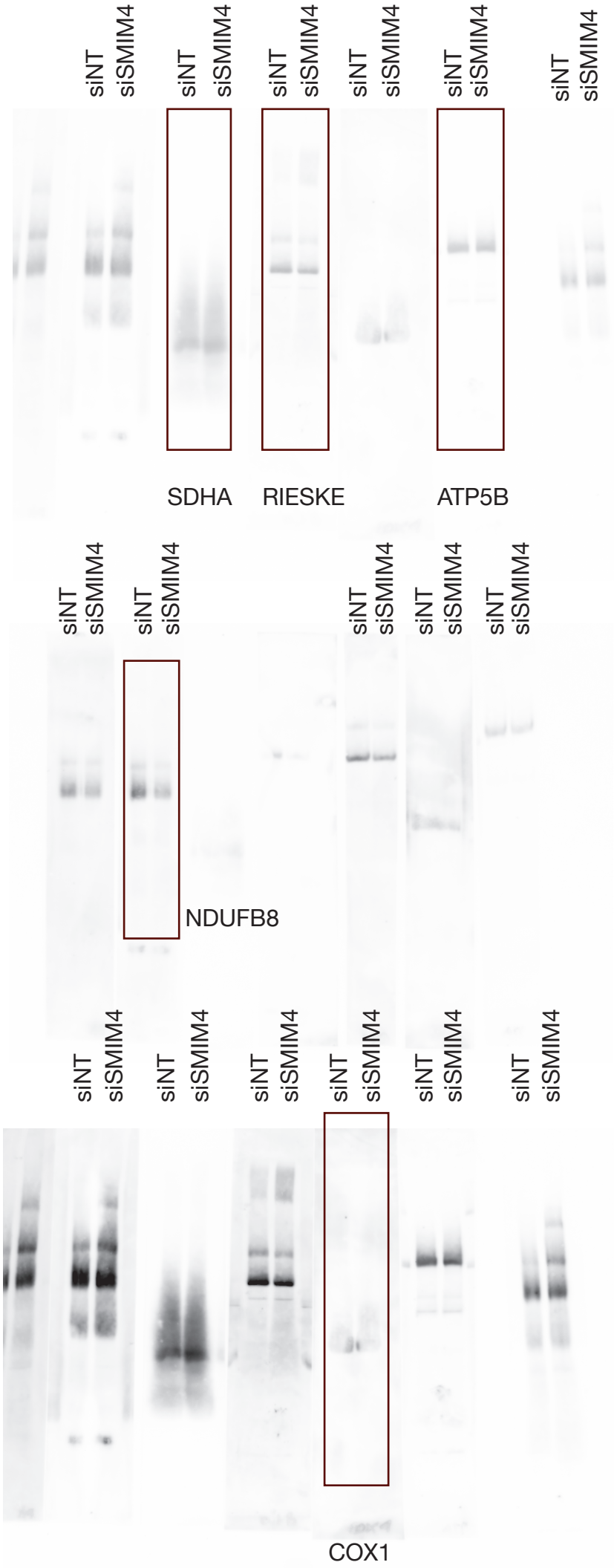

Supplement: Figure 5—source data 1. [file elife-68213-fig5-data1.zip › Figure_5_source_data/Figure_5_source_data_3_Figure_5D/Data_labelled/Figure_5_source_data_3_Figure_5D.pdf]

Figure 5 supplement 1 related to Figure 5 supplement 1B

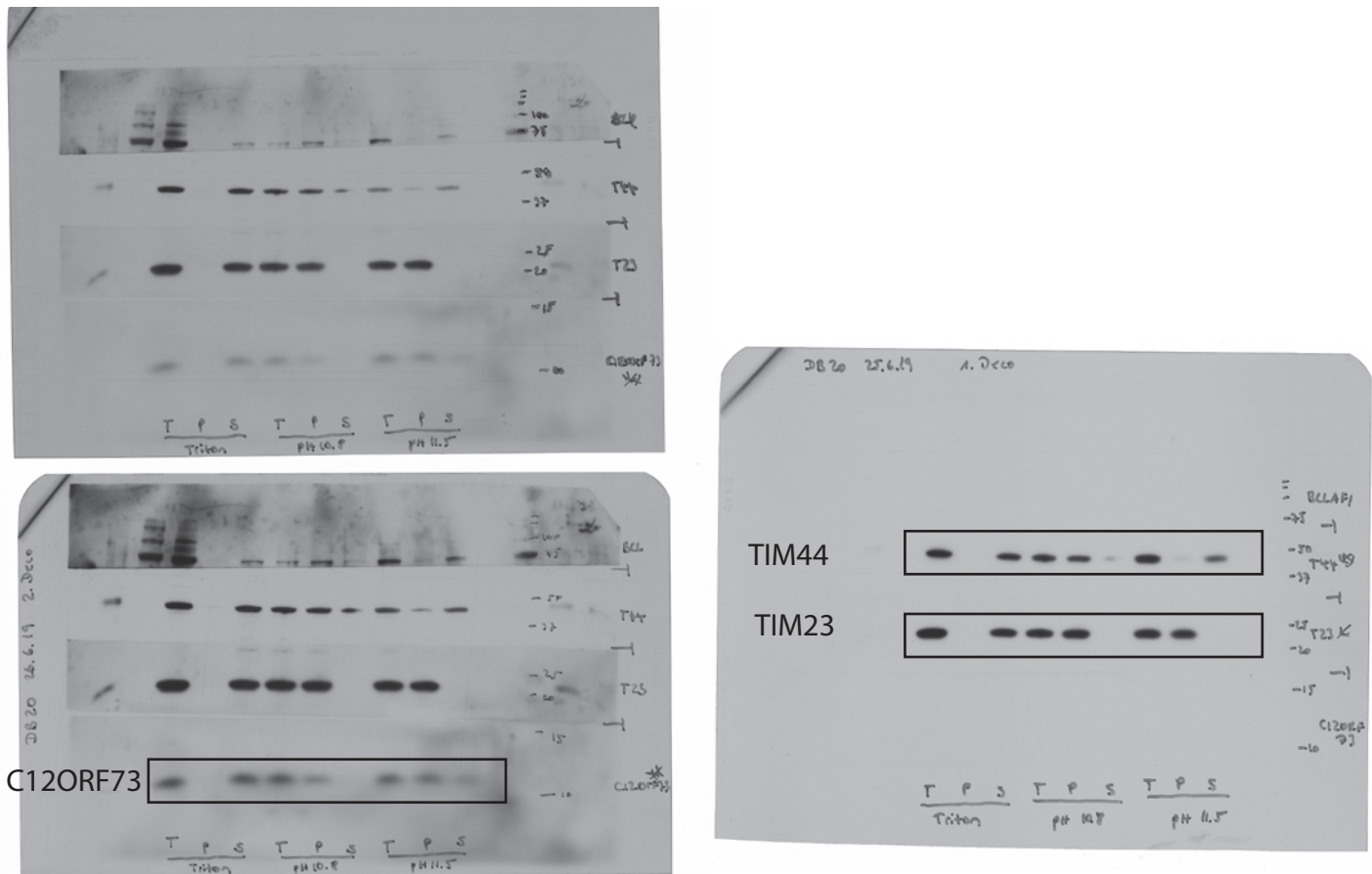

Supplement: Figure 5—figure supplement 1—source data 1. [file elife-68213-fig5-figsupp1-data1.zip › Figure_5_Supplement_1_source_data_1/Figure_5_Supplement_1_source_data_1_Figure_5_supplement_1B/Data_labelled/Figure_5_supplement_1_source_data_1_related_Figure_5_supplement_1B.pdf]

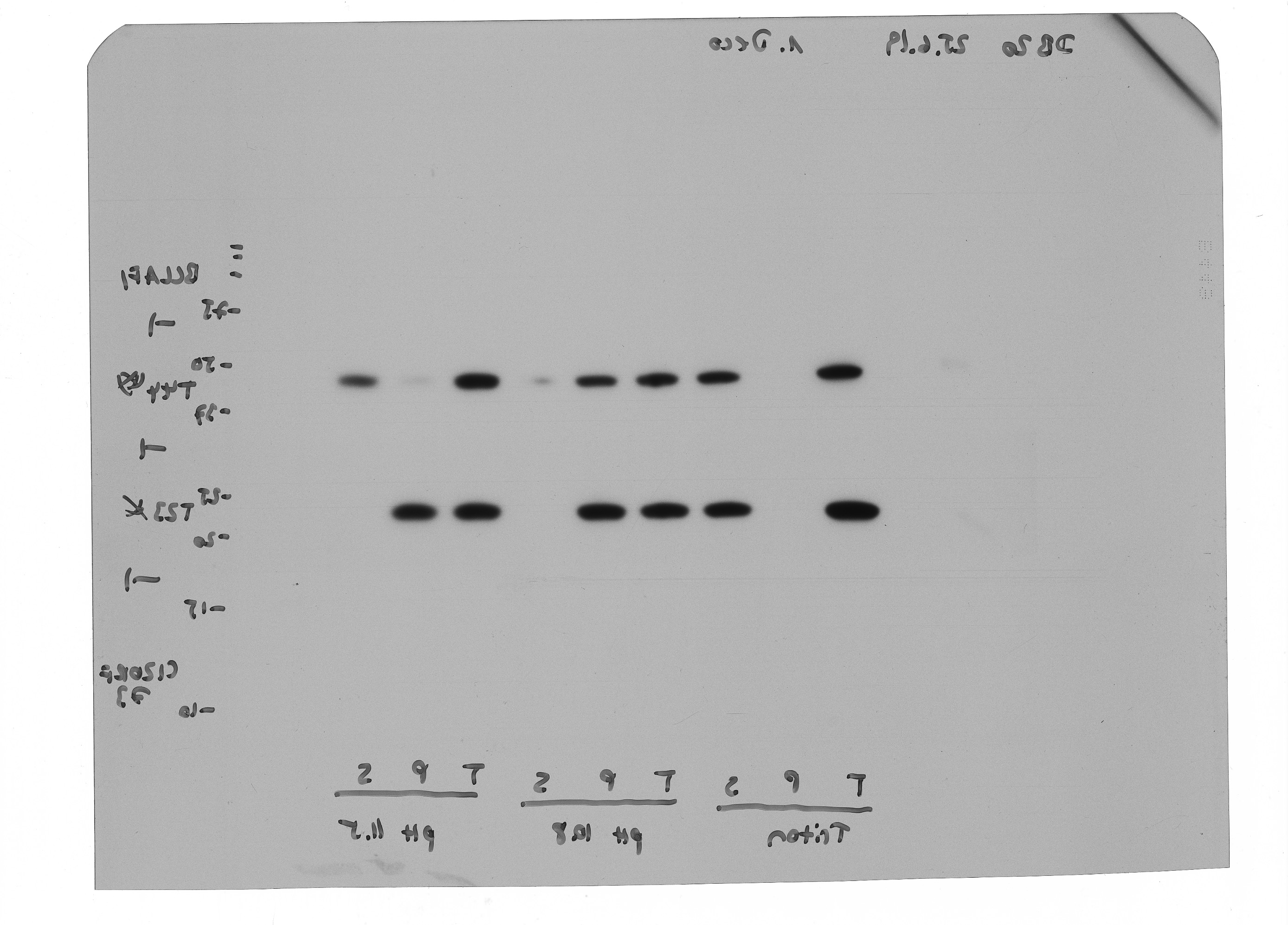

Supplement: Figure 5—figure supplement 1—source data 1. [file elife-68213-fig5-figsupp1-data1.zip › Figure_5_Supplement_1_source_data_1/Figure_5_Supplement_1_source_data_1_Figure_5_supplement_1B/Original_data/2004.jpg]

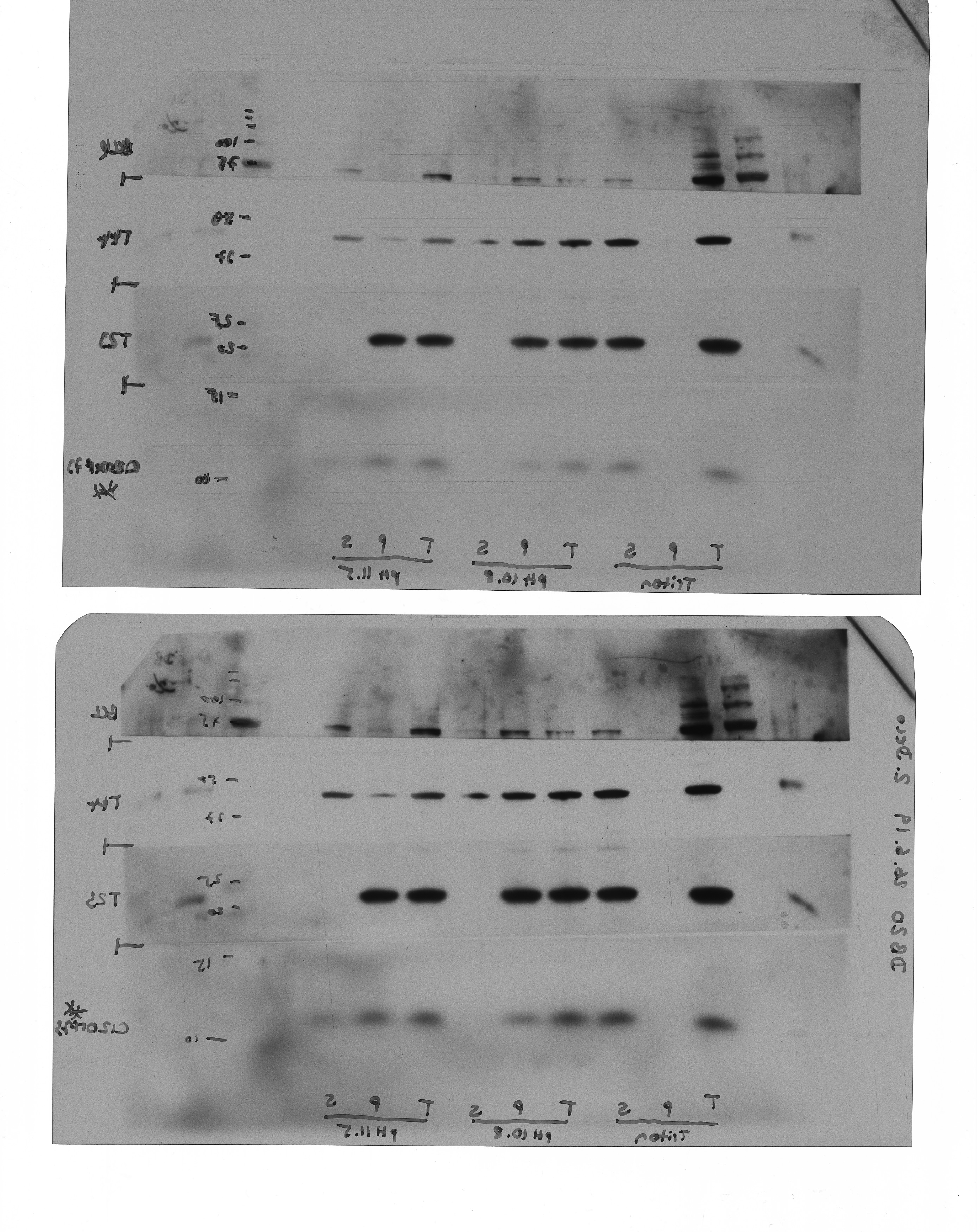

Supplement: Figure 5—figure supplement 1—source data 1. [file elife-68213-fig5-figsupp1-data1.zip › Figure_5_Supplement_1_source_data_1/Figure_5_Supplement_1_source_data_1_Figure_5_supplement_1B/Original_data/1003.jpg]

Figure 5 supplement 1 related to Figure 5 supplement 1D

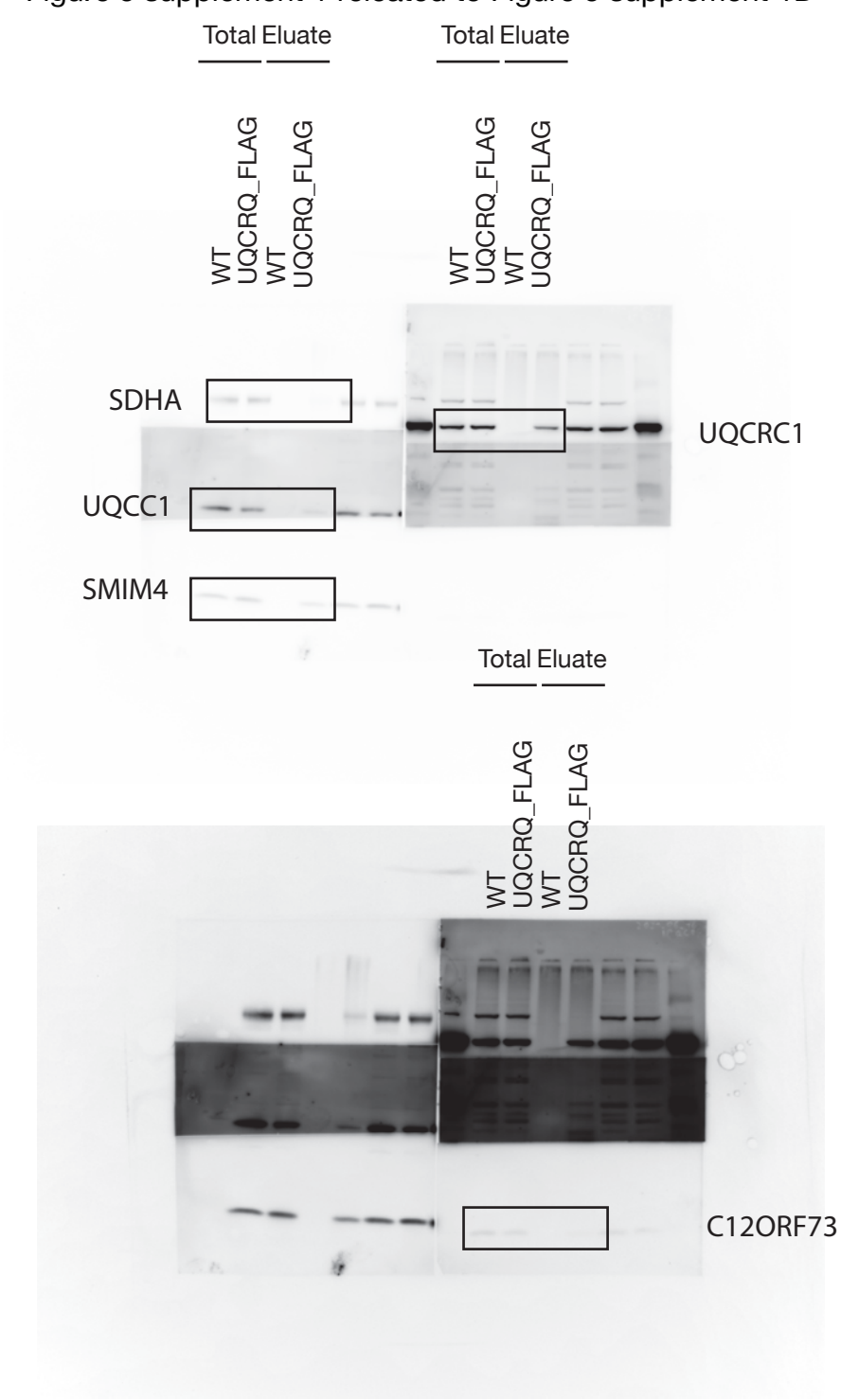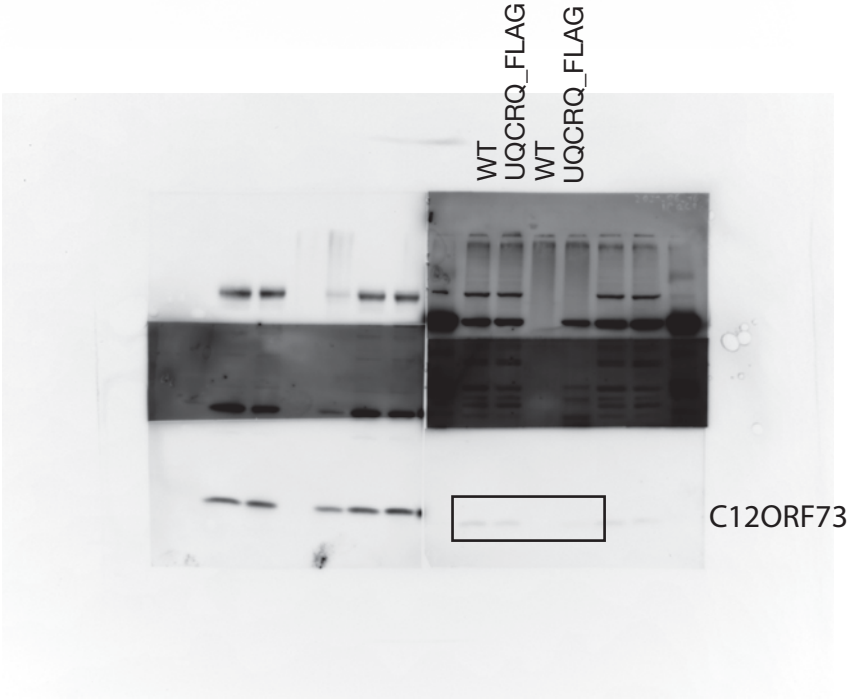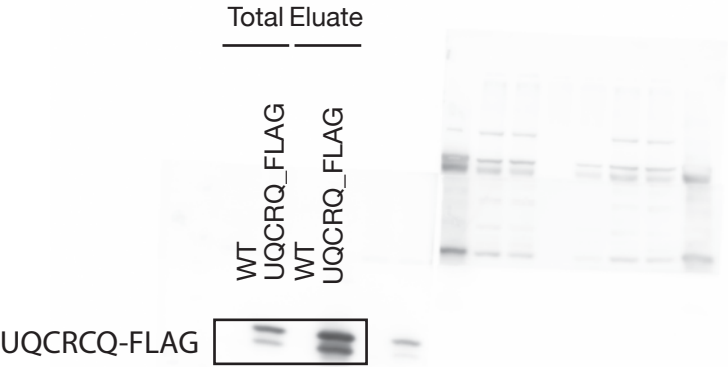

Supplement: Figure 5—figure supplement 1—source data 1. [file elife-68213-fig5-figsupp1-data1.zip › Figure_5_Supplement_1_source_data_1/Figure_5_supplement_1_source_data_3_Figure_5_supplement_1D/Data_labelled/Figure_5_supplement_1_source_data_3_related_Figure_5_supplement_1D.pdf]

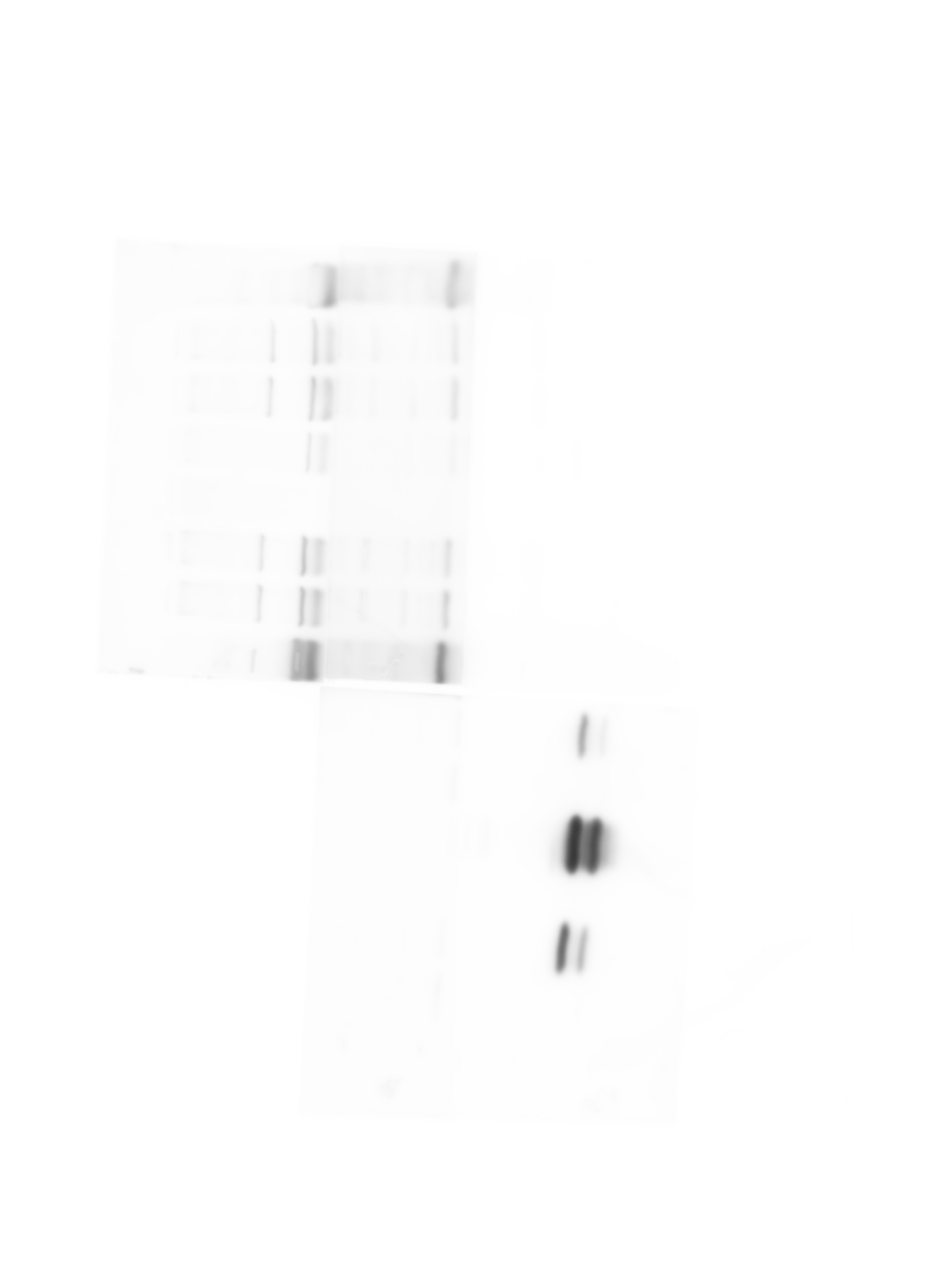

Supplement: Figure 5—figure supplement 1—source data 1. [file elife-68213-fig5-figsupp1-data1.zip › Figure_5_Supplement_1_source_data_1/Figure_5_supplement_1_source_data_3_Figure_5_supplement_1D/Original_data/2nd expo 20210618_102121-01_Ch_Chemi.jpg]

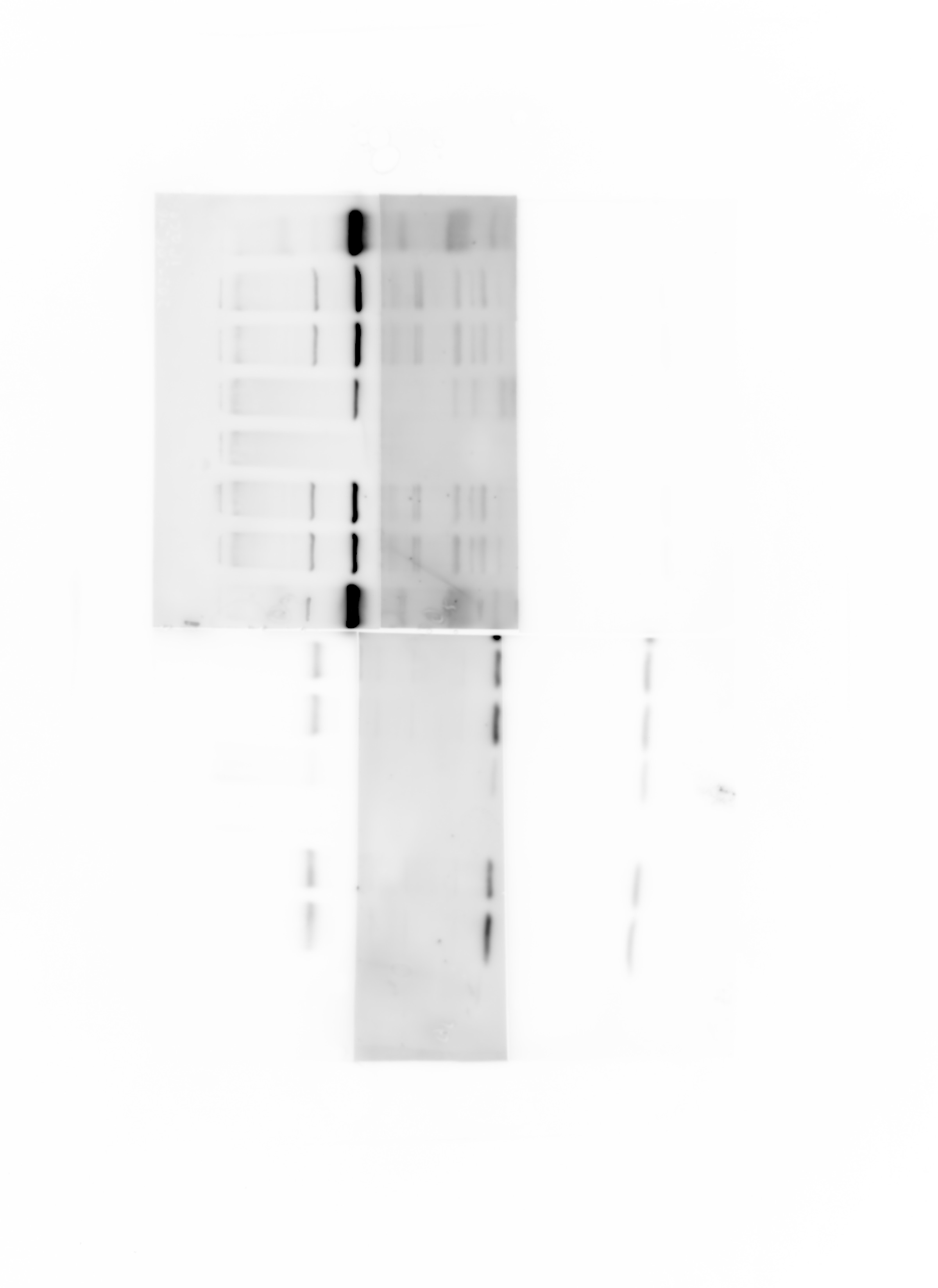

Supplement: Figure 5—figure supplement 1—source data 1. [file elife-68213-fig5-figsupp1-data1.zip › Figure_5_Supplement_1_source_data_1/Figure_5_supplement_1_source_data_3_Figure_5_supplement_1D/Original_data/1st 20210617_161720-20_Ch_Chemi.jpg]

Figure\_5\_supplement\_1\_source\_data\_2\_Figure\_5\_supplement\_1C

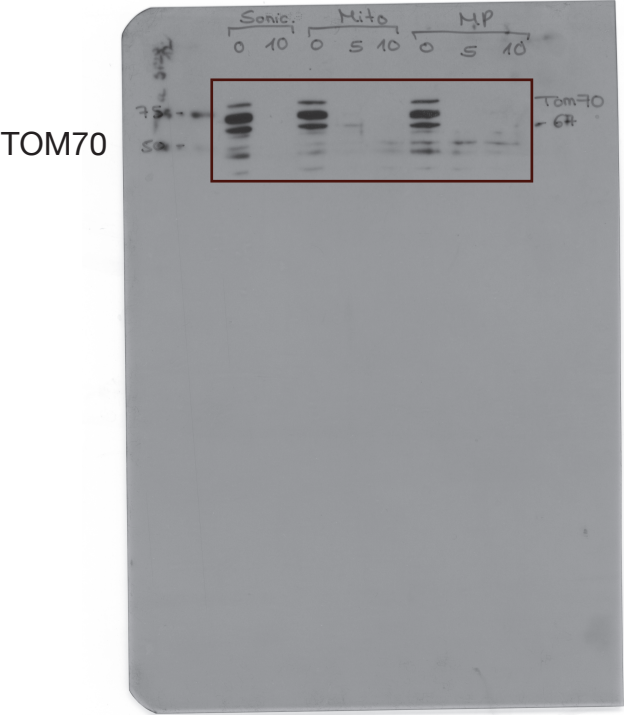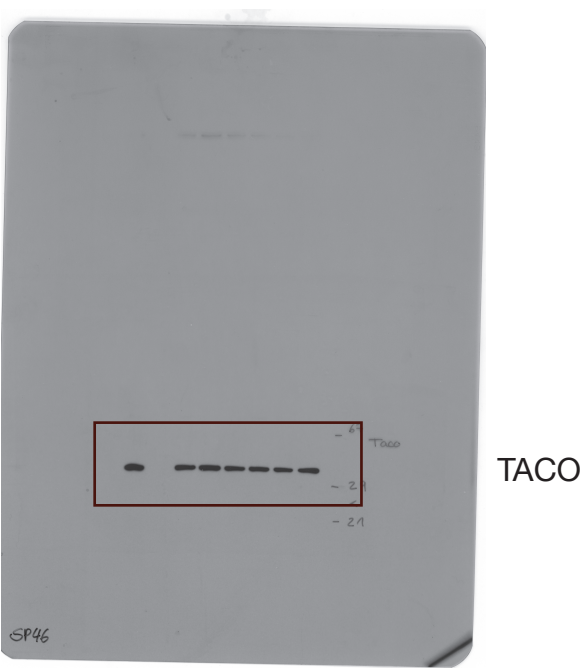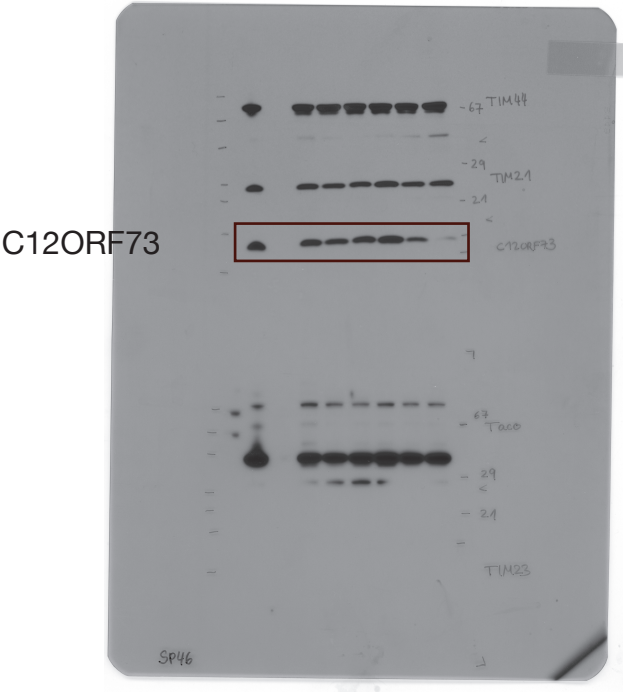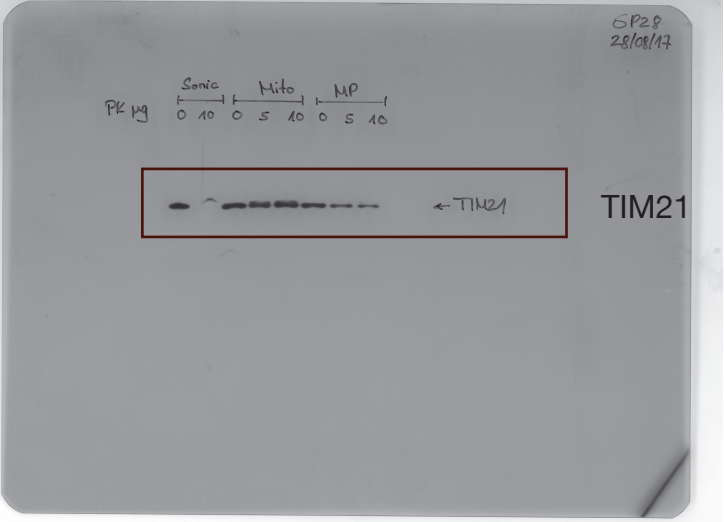

Supplement: Figure 5—figure supplement 1—source data 1. [file elife-68213-fig5-figsupp1-data1.zip › Figure_5_Supplement_1_source_data_1/Figure_5_supplement_1_source_data_2_Figure_5_supplement_1C/Data_labelled/Figure_5_supplement_1_source_data_2_Figure_5_supplement_1C.pdf]

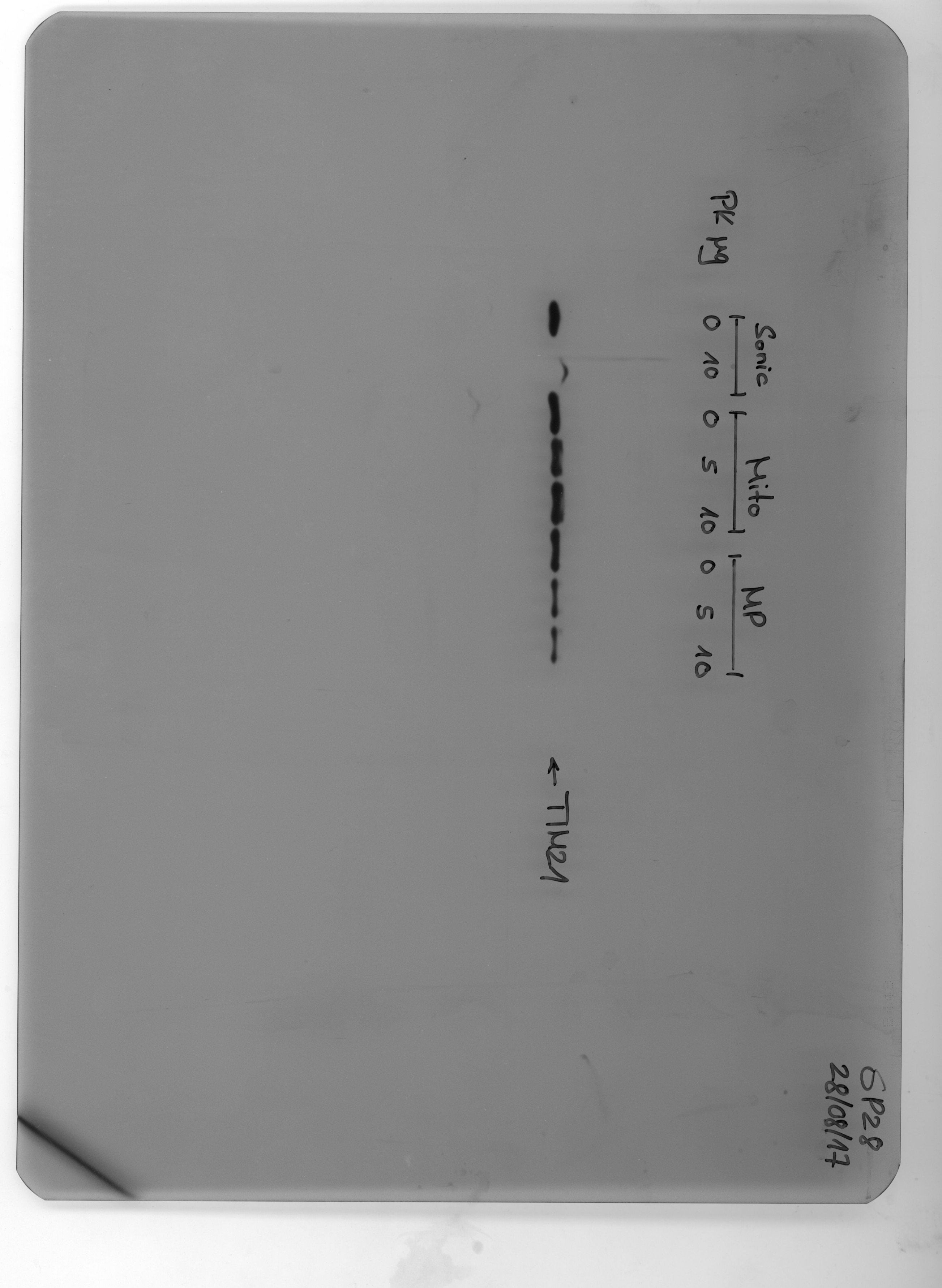

Supplement: Figure 5—figure supplement 1—source data 1. [file elife-68213-fig5-figsupp1-data1.zip › Figure_5_Supplement_1_source_data_1/Figure_5_supplement_1_source_data_2_Figure_5_supplement_1C/Original_files /SWELLING005.jpg]

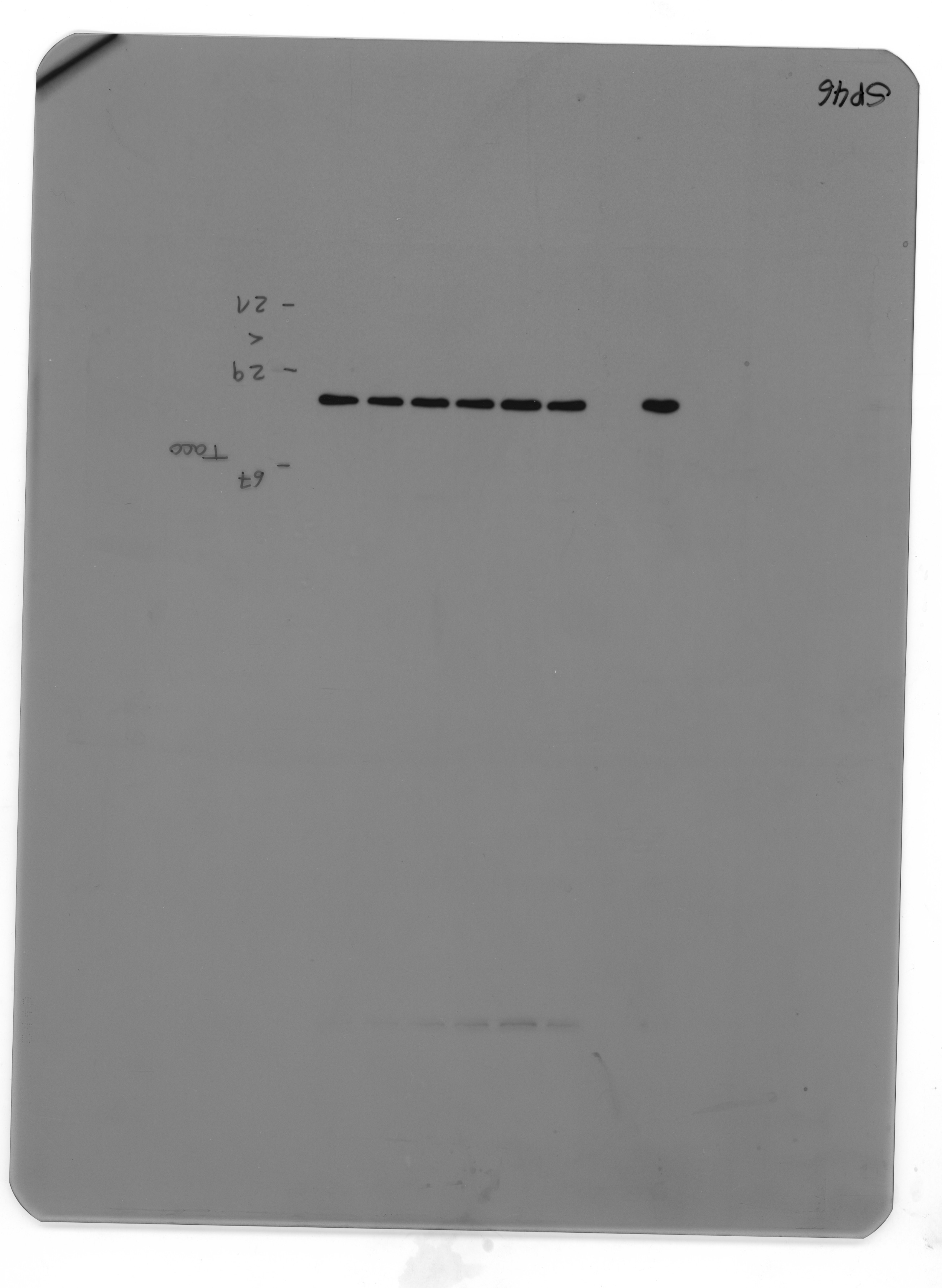

Supplement: Figure 5—figure supplement 1—source data 1. [file elife-68213-fig5-figsupp1-data1.zip › Figure_5_Supplement_1_source_data_1/Figure_5_supplement_1_source_data_2_Figure_5_supplement_1C/Original_files /SWELLING002.jpg]

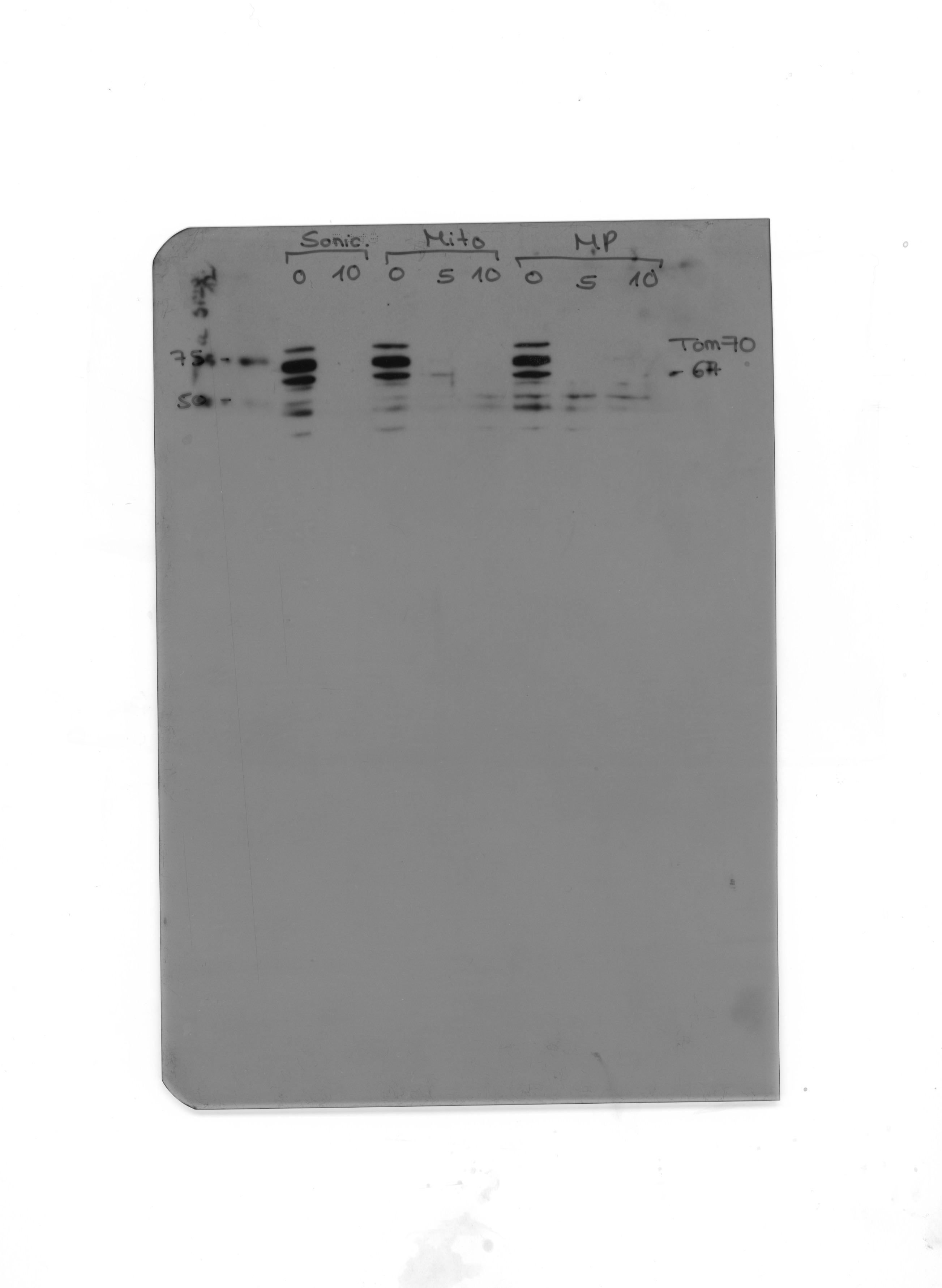

Supplement: Figure 5—figure supplement 1—source data 1. [file elife-68213-fig5-figsupp1-data1.zip › Figure_5_Supplement_1_source_data_1/Figure_5_supplement_1_source_data_2_Figure_5_supplement_1C/Original_files /SWELLING003.jpg]

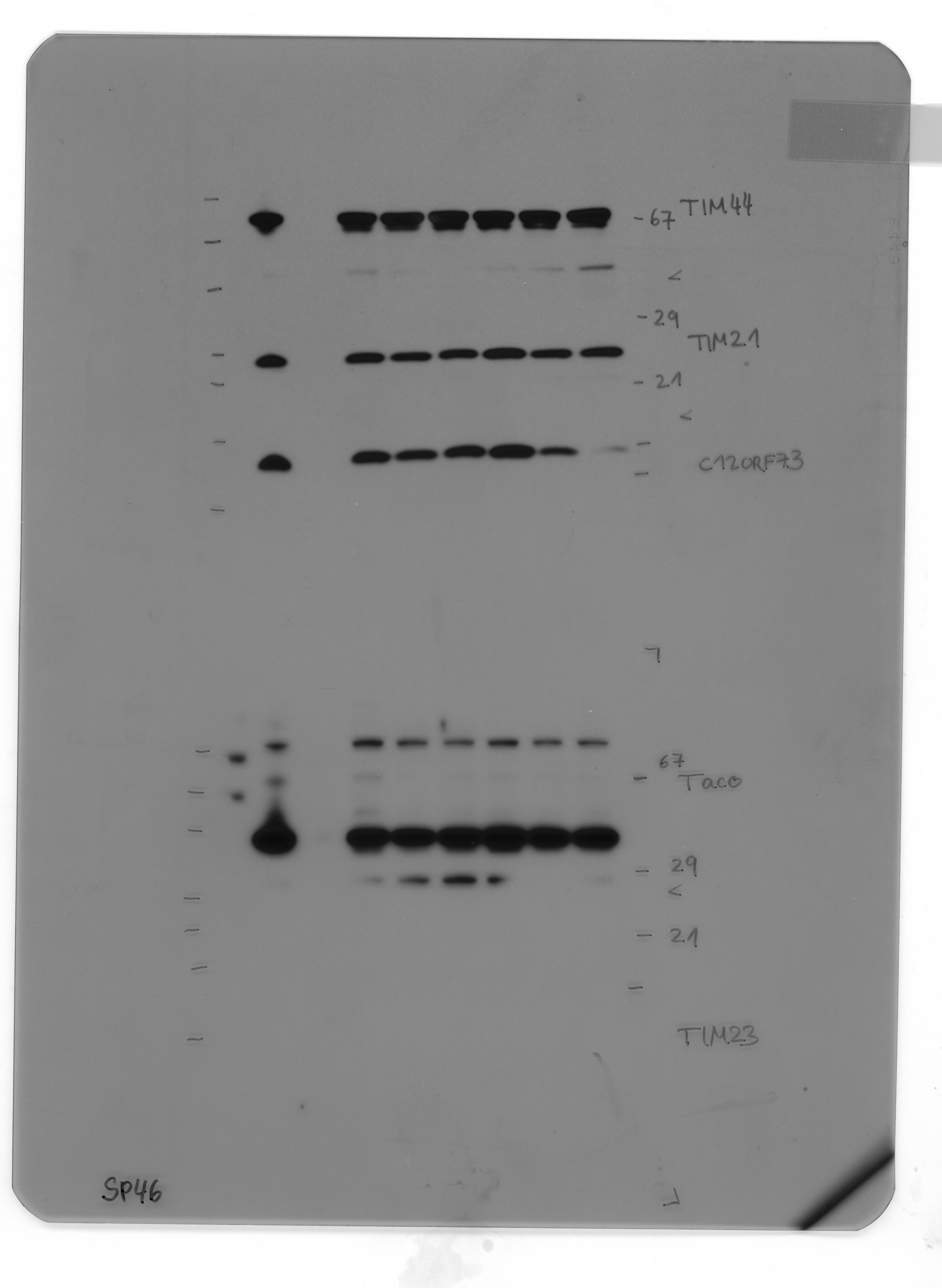

Supplement: Figure 5—figure supplement 1—source data 1. [file elife-68213-fig5-figsupp1-data1.zip › Figure_5_Supplement_1_source_data_1/Figure_5_supplement_1_source_data_2_Figure_5_supplement_1C/Original_files /SWELLING001.jpg]

Figure 5 supplement 1 related to Figure 5 supplement 1E

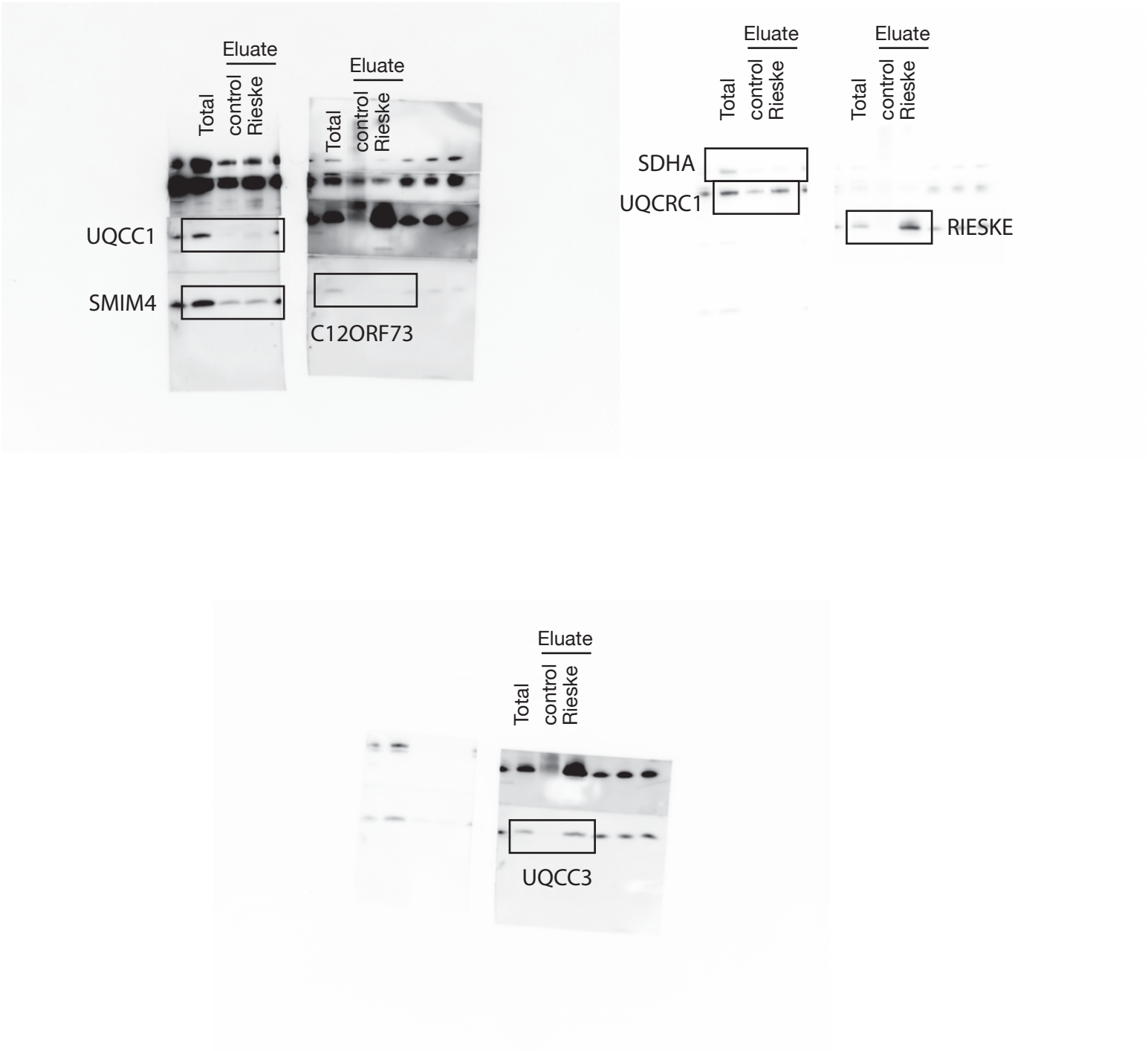

Supplement: Figure 5—figure supplement 1—source data 2. [file elife-68213-fig5-figsupp1-data2.zip › Figure_5_supplement_1_source_data_2/Figure_5_supplement_1_source_data_4_Figure_5_supplement_1E/Data_labelled/Figure_5_supplement_1_source_data_4_related_Figure_5_supplement_1E.pdf]

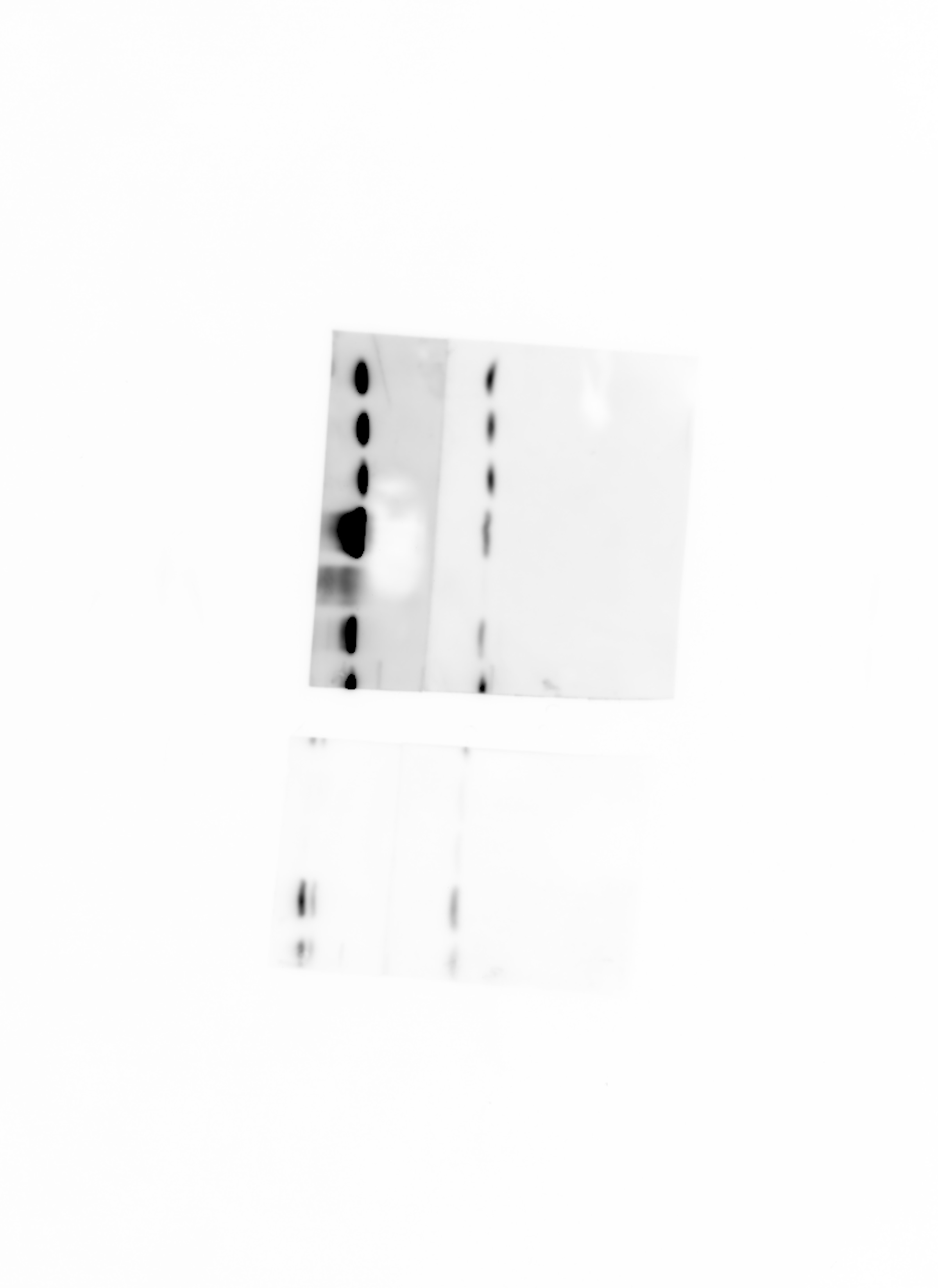

Supplement: Figure 5—figure supplement 1—source data 2. [file elife-68213-fig5-figsupp1-data2.zip › Figure_5_supplement_1_source_data_2/Figure_5_supplement_1_source_data_4_Figure_5_supplement_1E/Original_data/2nd 20210728_135227-19_Ch_Chemi.jpg]

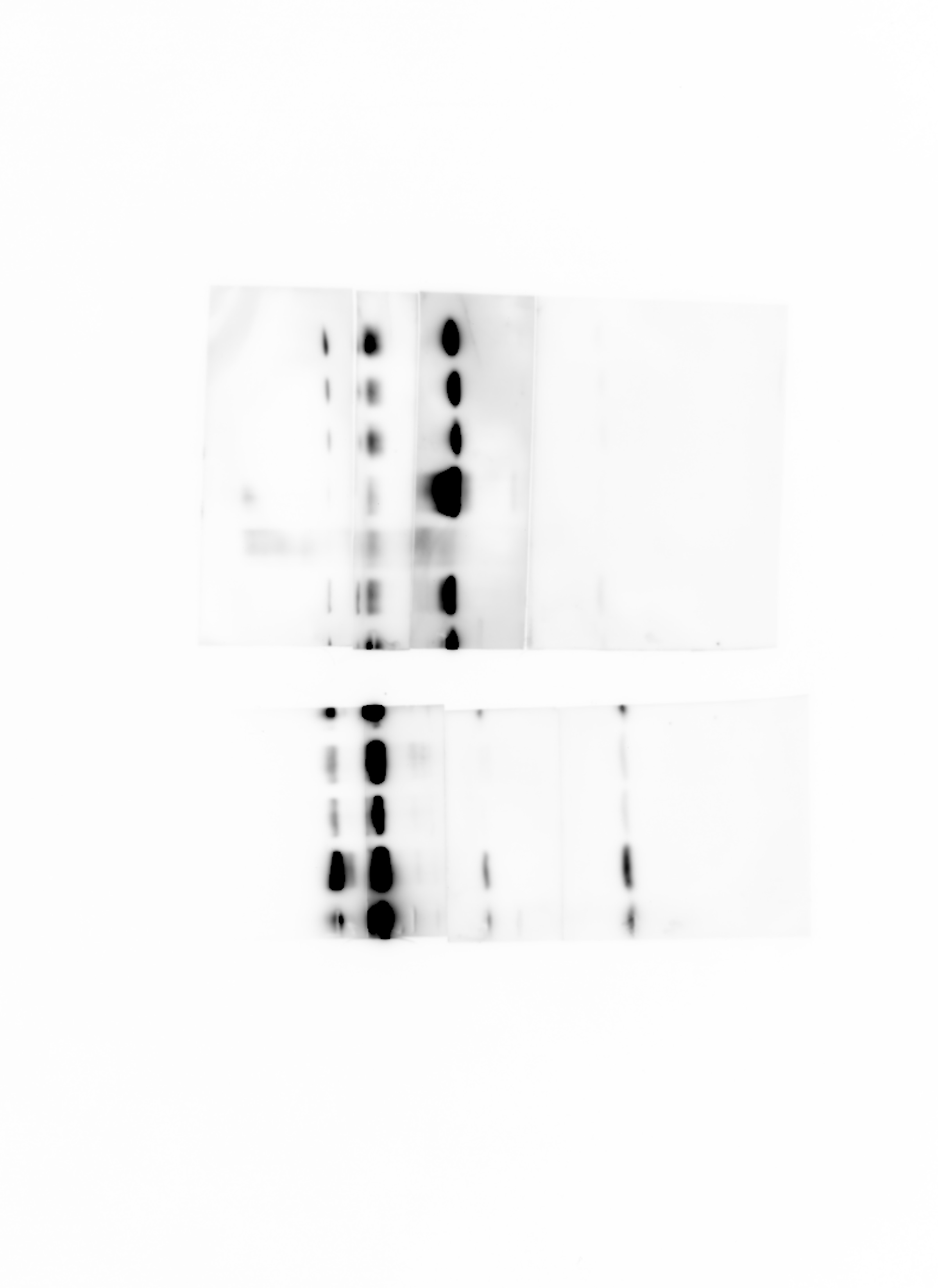

Supplement: Figure 5—figure supplement 1—source data 2. [file elife-68213-fig5-figsupp1-data2.zip › Figure_5_supplement_1_source_data_2/Figure_5_supplement_1_source_data_4_Figure_5_supplement_1E/Original_data/1st 20210727_131308-20_Ch_Chemi.jpg]
