# Supplementary material for: Defining the interactome of the human mitochondrial ribosome identifies SMIM4 and TMEM223 as respiratory chain assembly factors
Source: eLife. 2021 Dec 31;10:e68213. doi: 10.7554/eLife.68213 (PMC8719881; doi:10.7554/eLife.68213)

Figure 5 source data 2 related to Figure 5B

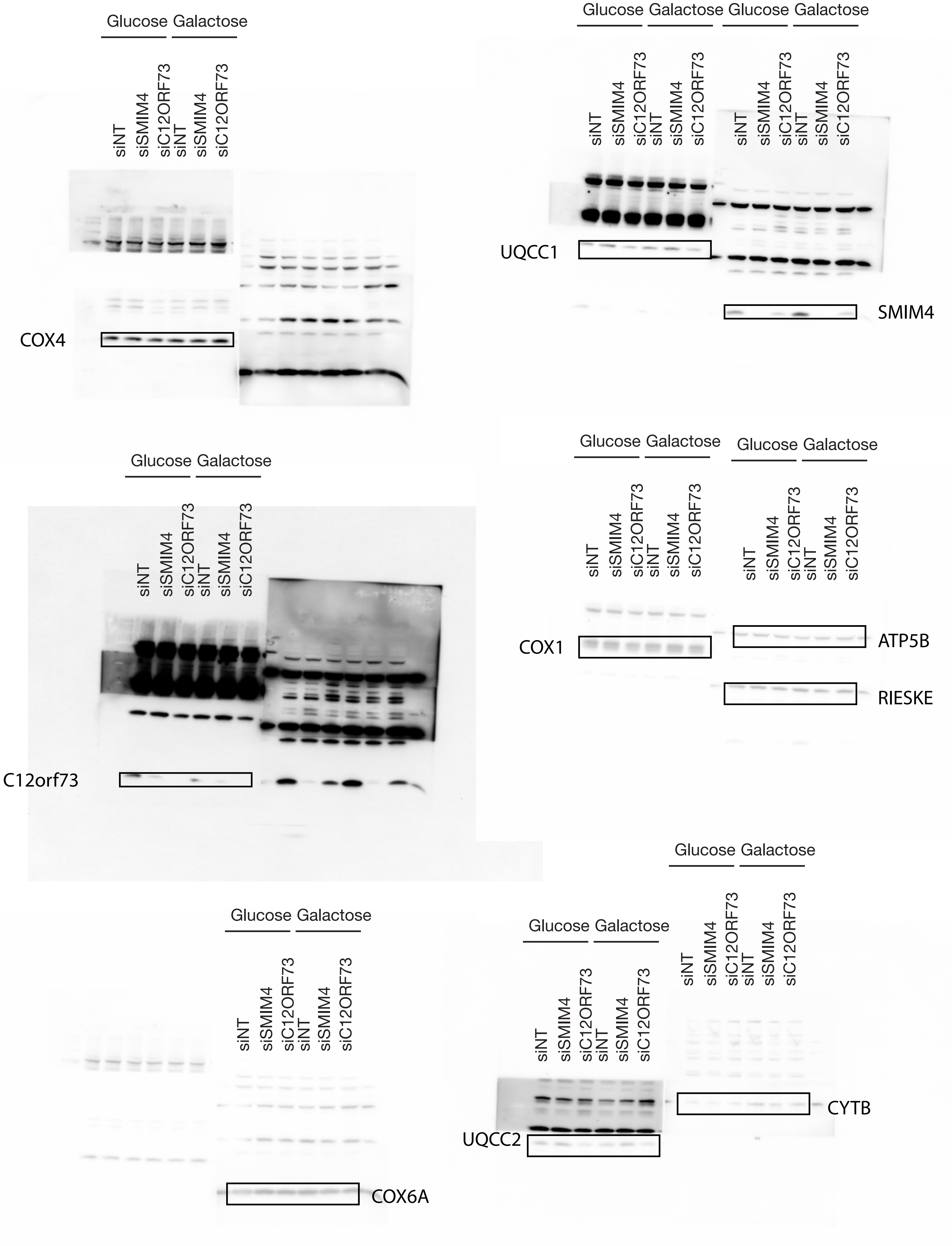

Figure 5 source data 2 related to Figure 5B

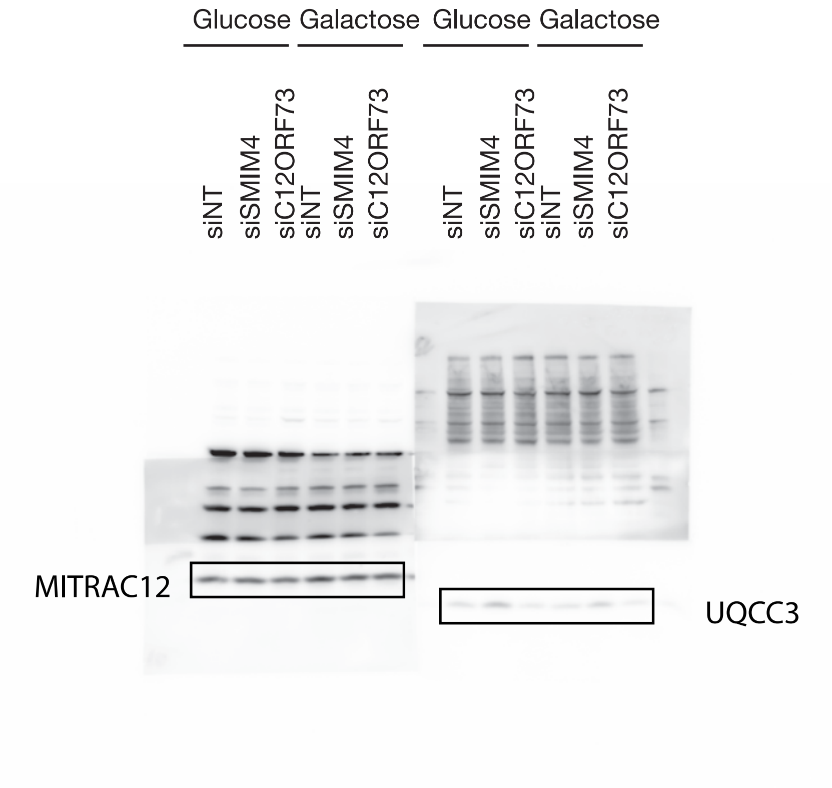

Supplement: Figure 5—source data 1. [file elife-68213-fig5-data1.zip › Figure_5_source_data/Figure_5_source_data_2_Figure_5B/Data_labelled/Figure_5_source_data_2_Figure_5B.pdf]
