# Supplementary figures and images for: Defining the interactome of the human mitochondrial ribosome identifies SMIM4 and TMEM223 as respiratory chain assembly factors (part 2 of 2)
Source: eLife. 2021 Dec 31;10:e68213. doi: 10.7554/eLife.68213 (PMC8719881; doi:10.7554/eLife.68213)

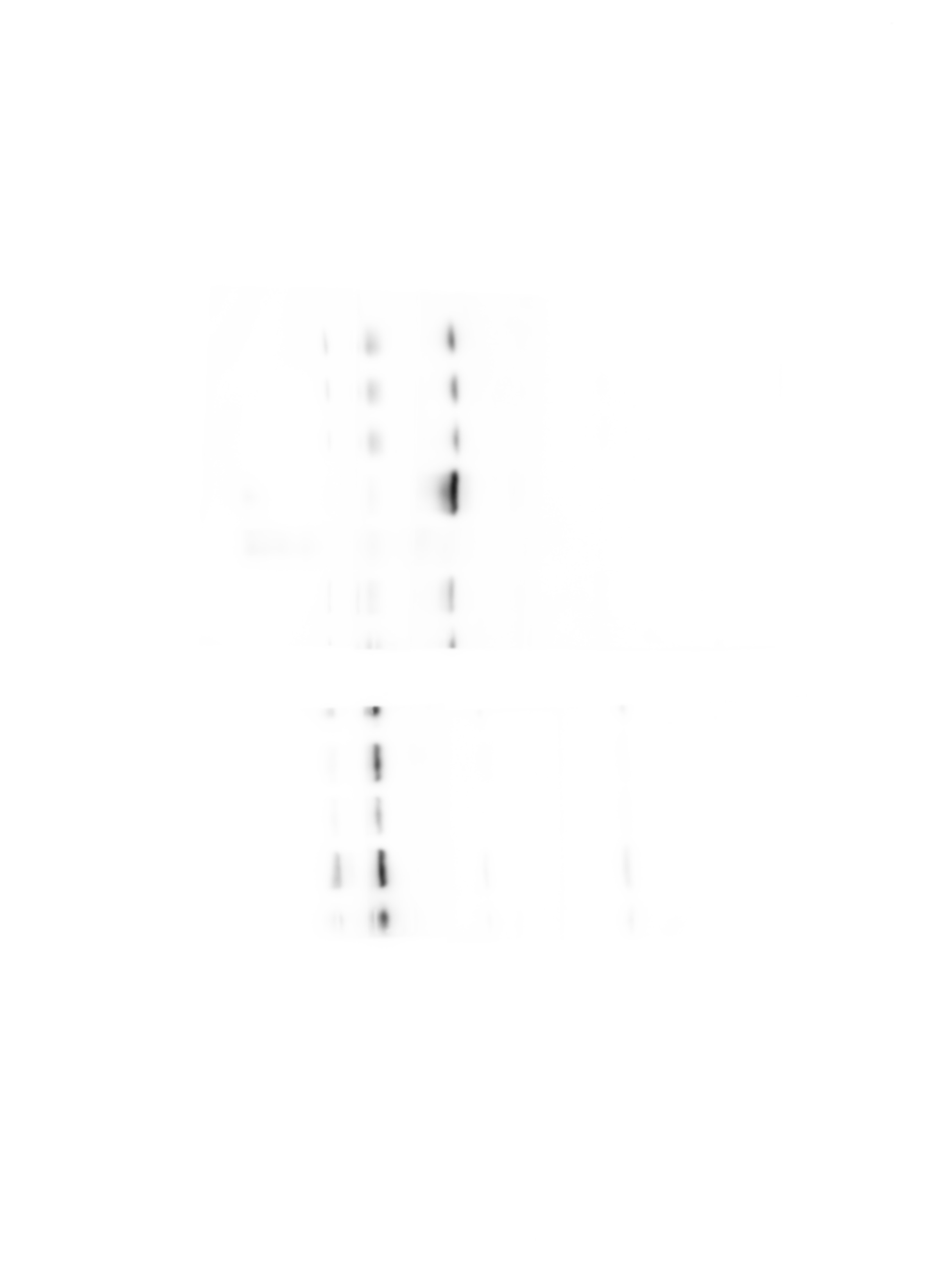

Supplement: Figure 5—figure supplement 1—source data 2. [file elife-68213-fig5-figsupp1-data2.zip › Figure_5_supplement_1_source_data_2/Figure_5_supplement_1_source_data_4_Figure_5_supplement_1E/Original_data/1st 20210727_131308-01_Ch_Chemi.jpg]

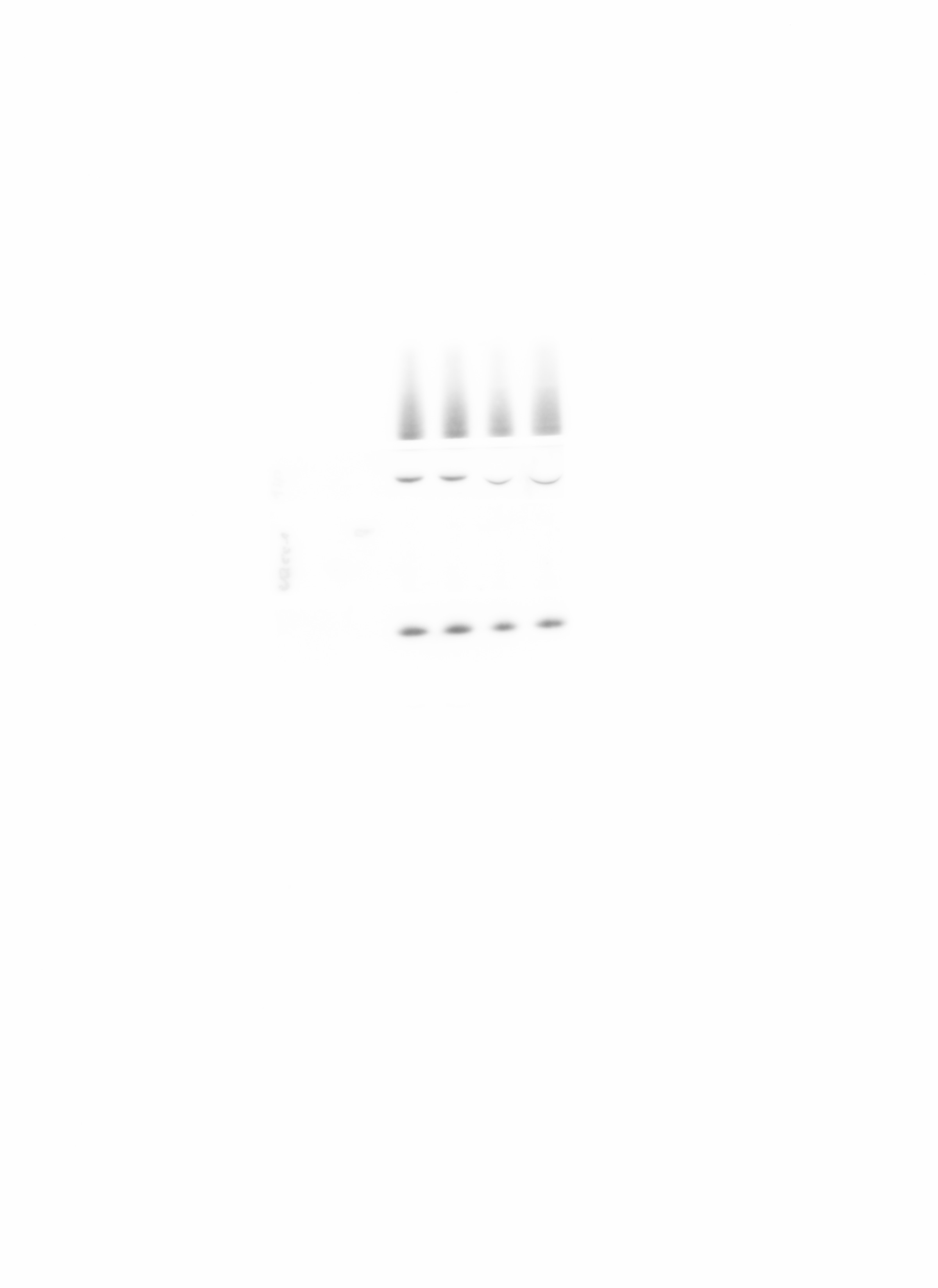

Supplement: Figure 5—figure supplement 1—source data 2. [file elife-68213-fig5-figsupp1-data2.zip › Figure_5_supplement_1_source_data_2/Figure_5_supplement_1_source_data_5_Figure_5_supplement_1F/Original_files/steady state KD SMIM4 Revision 20211020_145701-04_Ch_Chemi.jpg]

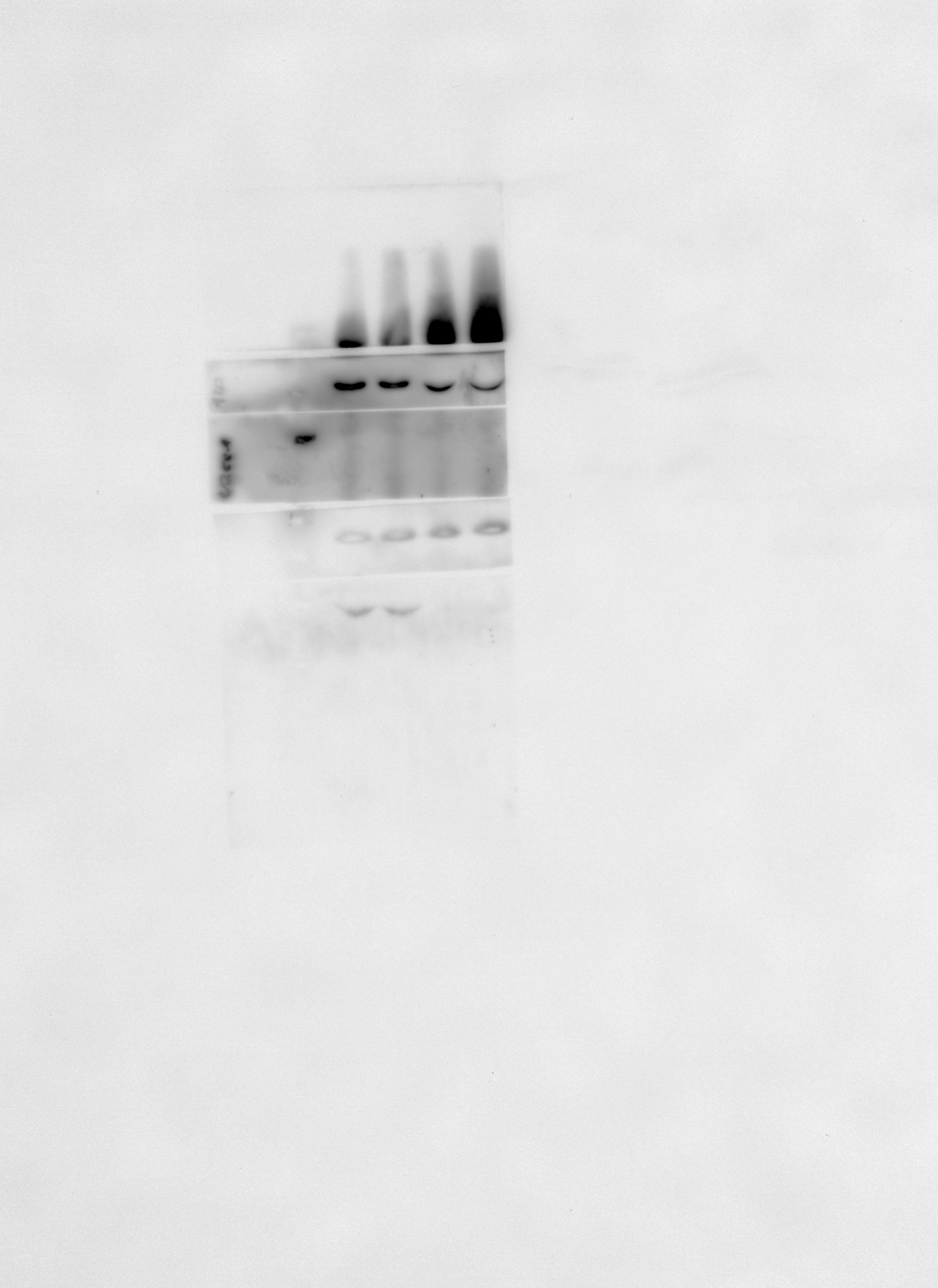

Supplement: Figure 5—figure supplement 1—source data 2. [file elife-68213-fig5-figsupp1-data2.zip › Figure_5_supplement_1_source_data_2/Figure_5_supplement_1_source_data_5_Figure_5_supplement_1F/Original_files/steady state KD SMIM4 Revision 20211020_150713-18_Ch_Chemi.jpg]

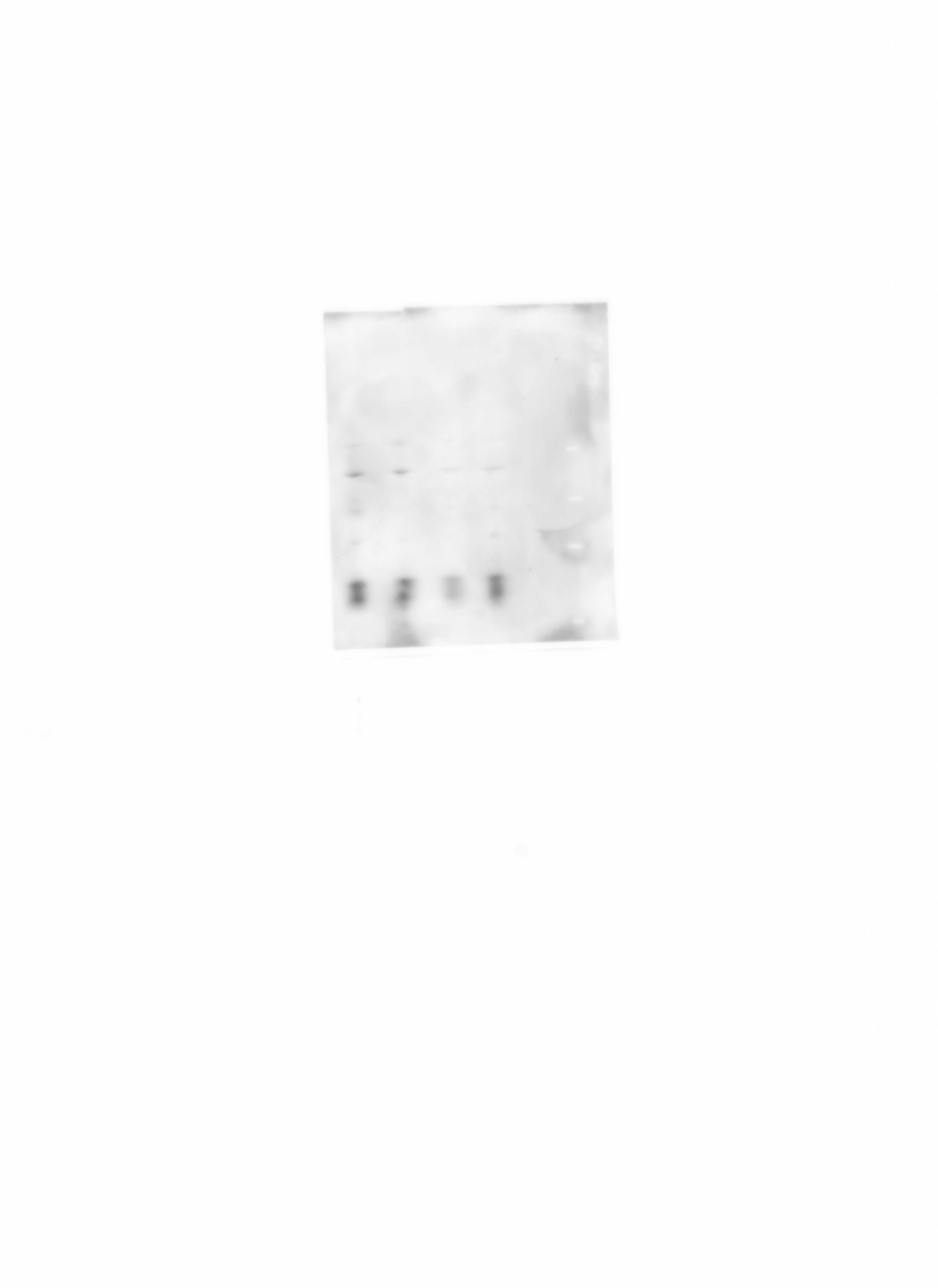

Supplement: Figure 5—figure supplement 1—source data 2. [file elife-68213-fig5-figsupp1-data2.zip › Figure_5_supplement_1_source_data_2/Figure_5_supplement_1_source_data_5_Figure_5_supplement_1F/Original_files/KD SMIM4 steady state Revision 20211021_153746-01_Ch_Chemi.jpg]

Figure\_5\_supplement\_1\_source\_data\_6\_Figure\_5\_supplement\_1G

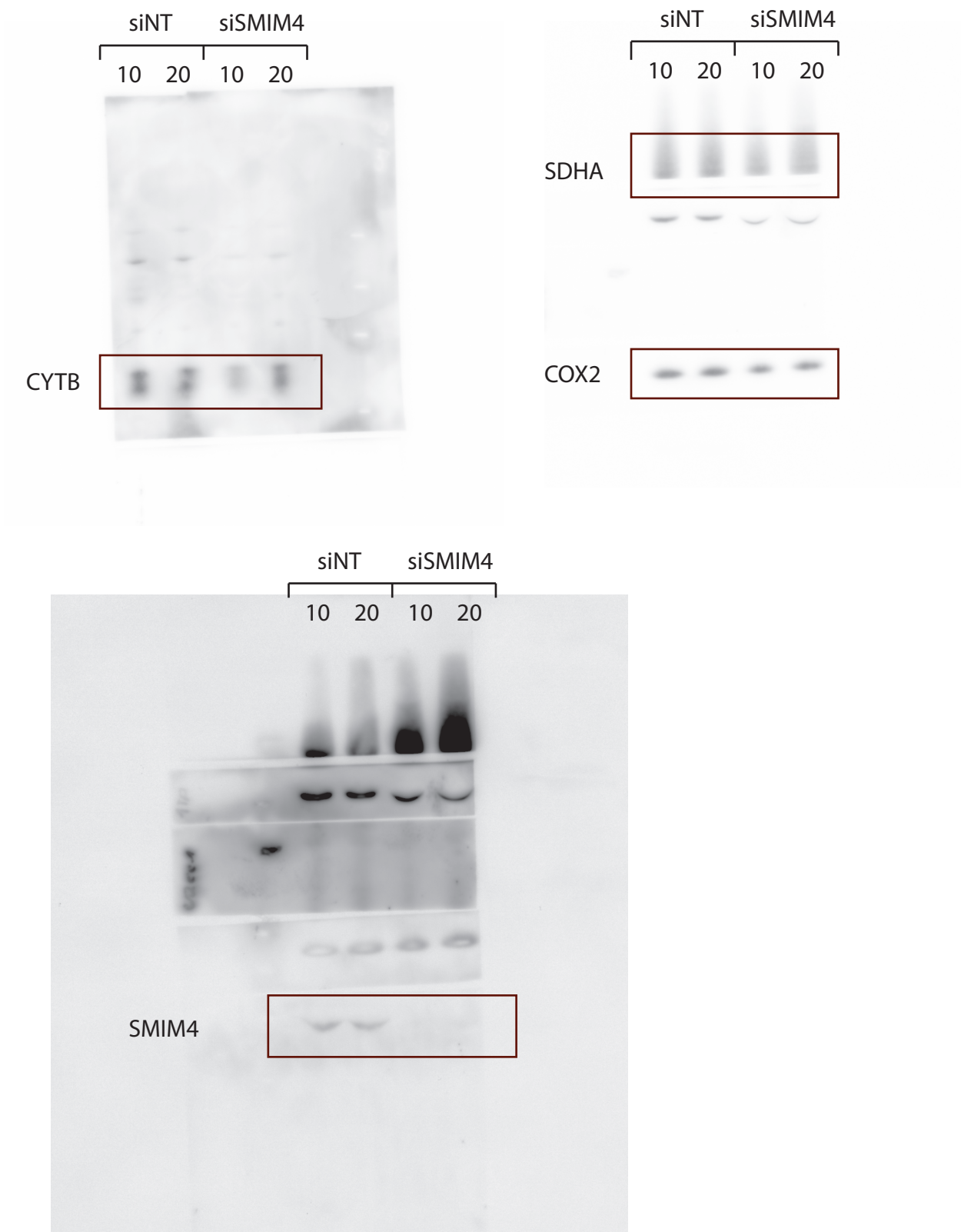

Supplement: Figure 5—figure supplement 1—source data 2. [file elife-68213-fig5-figsupp1-data2.zip › Figure_5_supplement_1_source_data_2/Figure_5_supplement_1_source_data_5_Figure_5_supplement_1F/Data_labelled/Figure_5_supplement_1_source_data_5_Figure_5_supplement_1F.pdf]

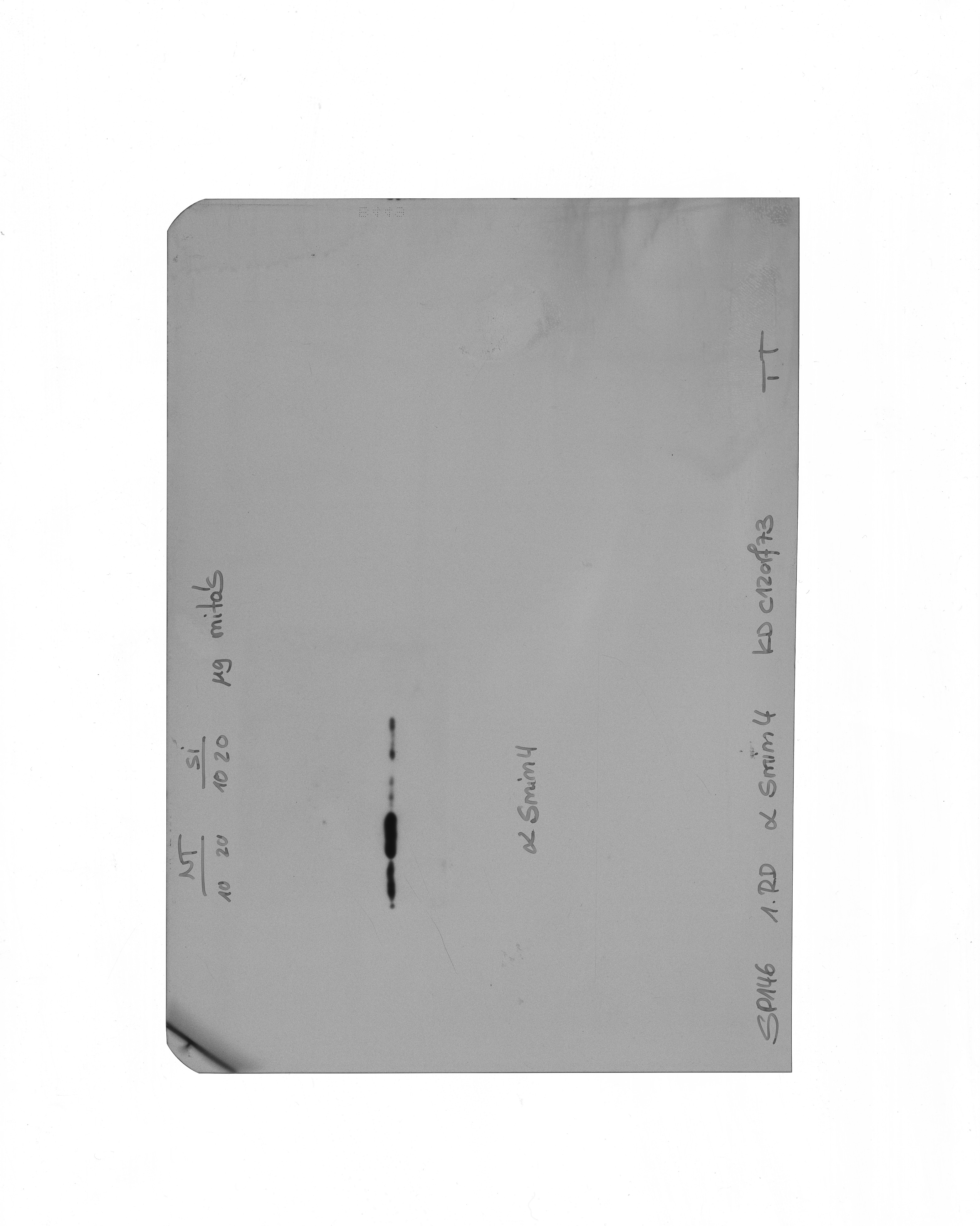

Supplement: Figure 5—figure supplement 1—source data 2. [file elife-68213-fig5-figsupp1-data2.zip › Figure_5_supplement_1_source_data_2/Figure_5_supplement_1_source_data_6_Figure_5_supplement_1G/Original_files/SP146 005.jpg]

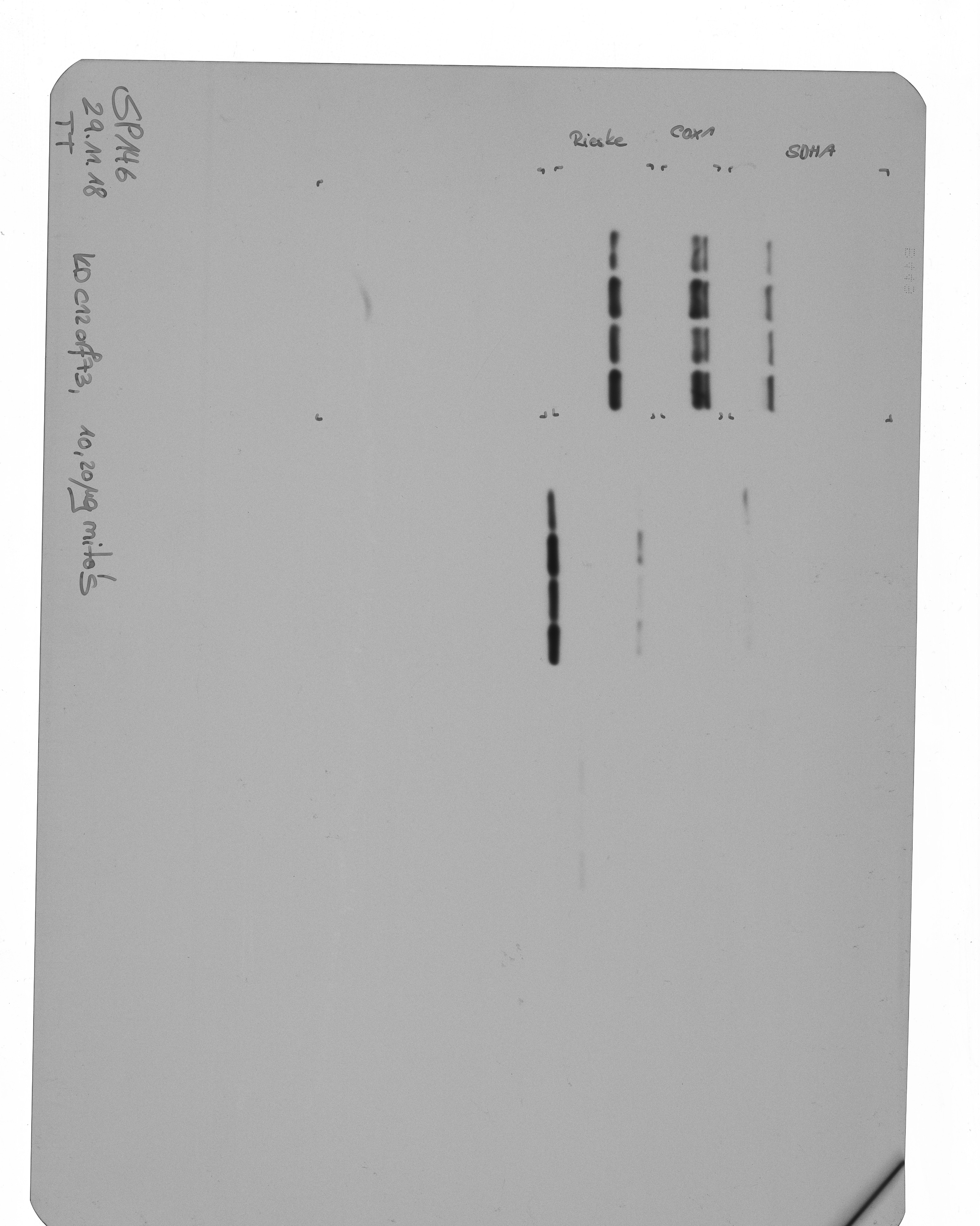

Supplement: Figure 5—figure supplement 1—source data 2. [file elife-68213-fig5-figsupp1-data2.zip › Figure_5_supplement_1_source_data_2/Figure_5_supplement_1_source_data_6_Figure_5_supplement_1G/Original_files/SP146 004.jpg]

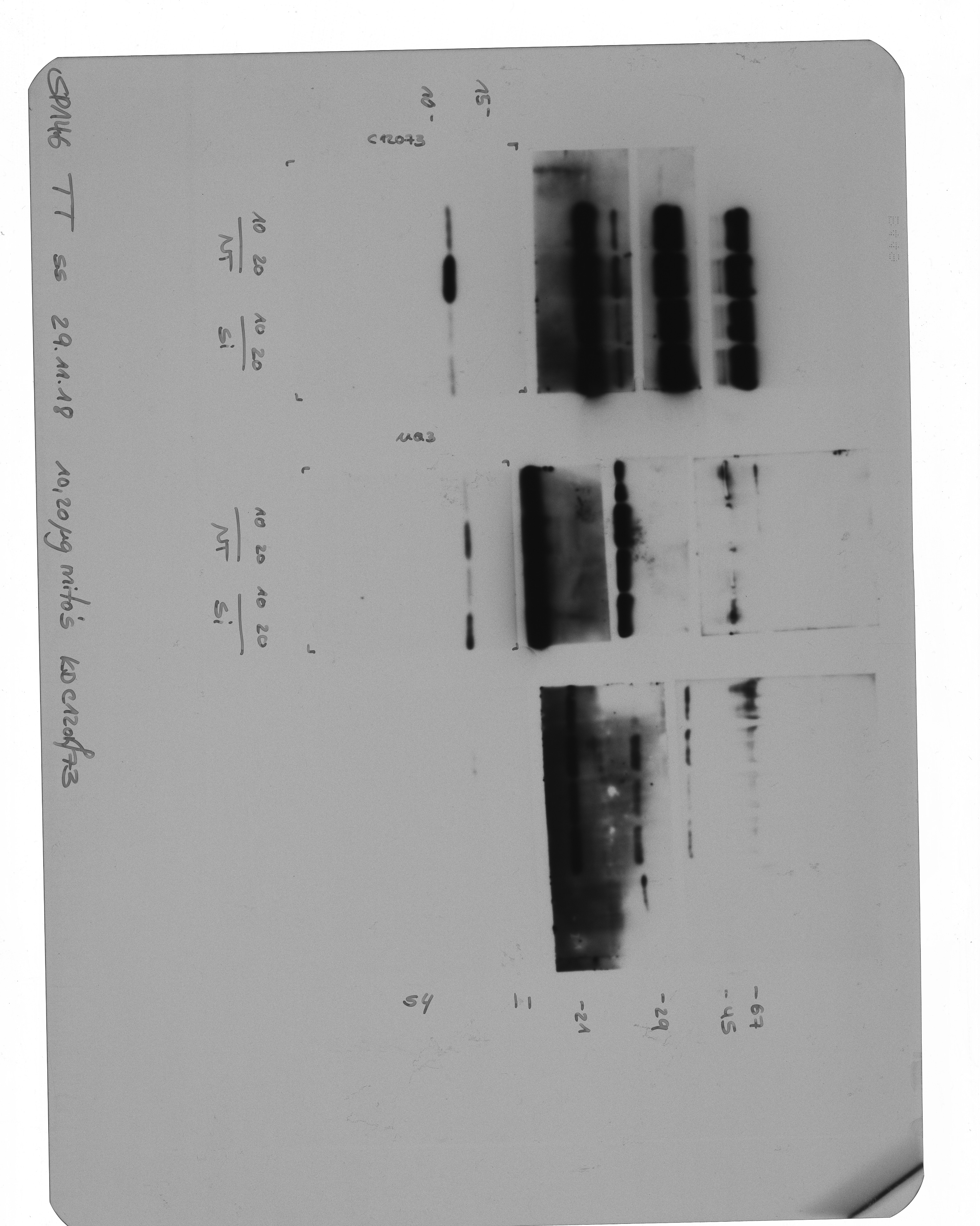

Supplement: Figure 5—figure supplement 1—source data 2. [file elife-68213-fig5-figsupp1-data2.zip › Figure_5_supplement_1_source_data_2/Figure_5_supplement_1_source_data_6_Figure_5_supplement_1G/Original_files/SP146 002.jpg]

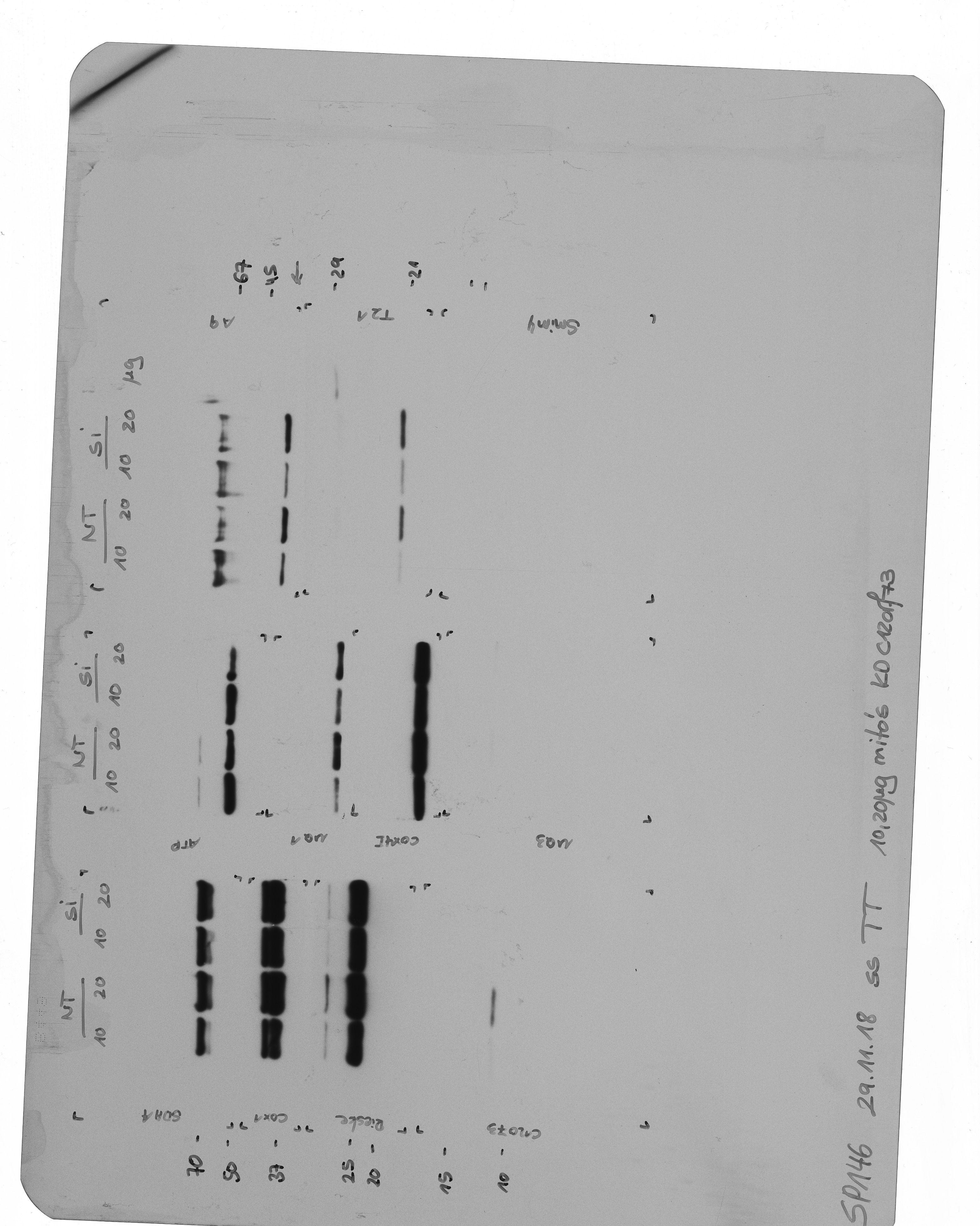

Supplement: Figure 5—figure supplement 1—source data 2. [file elife-68213-fig5-figsupp1-data2.zip › Figure_5_supplement_1_source_data_2/Figure_5_supplement_1_source_data_6_Figure_5_supplement_1G/Original_files/SP146 001.jpg]

Figure\_5\_supplement\_1\_source\_data\_6\_Figure\_5\_supplement\_1G

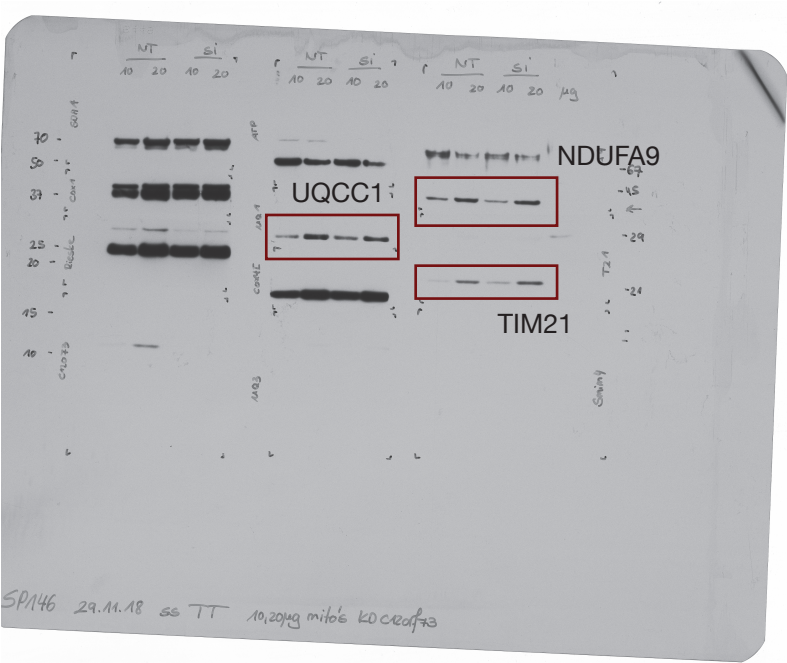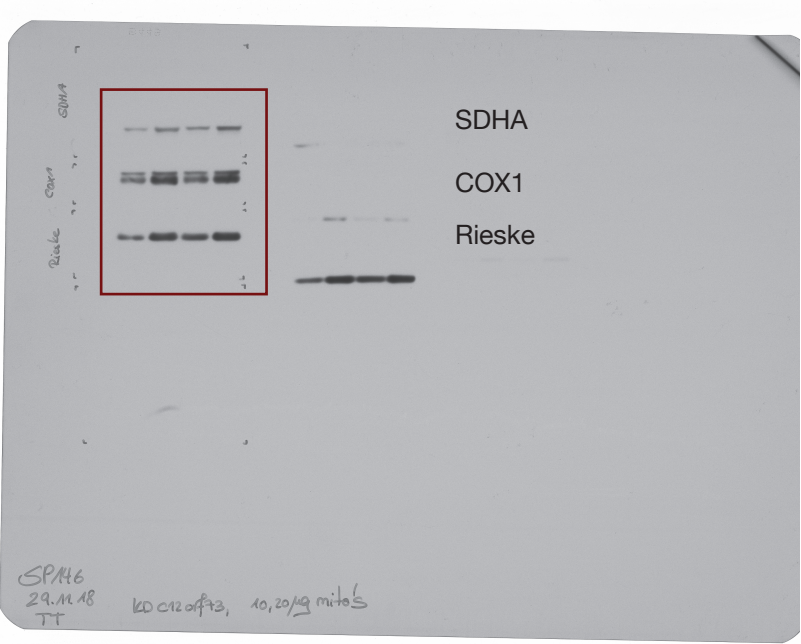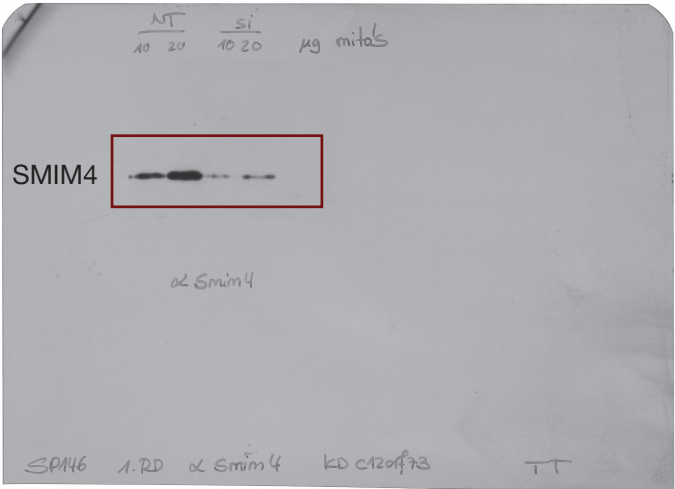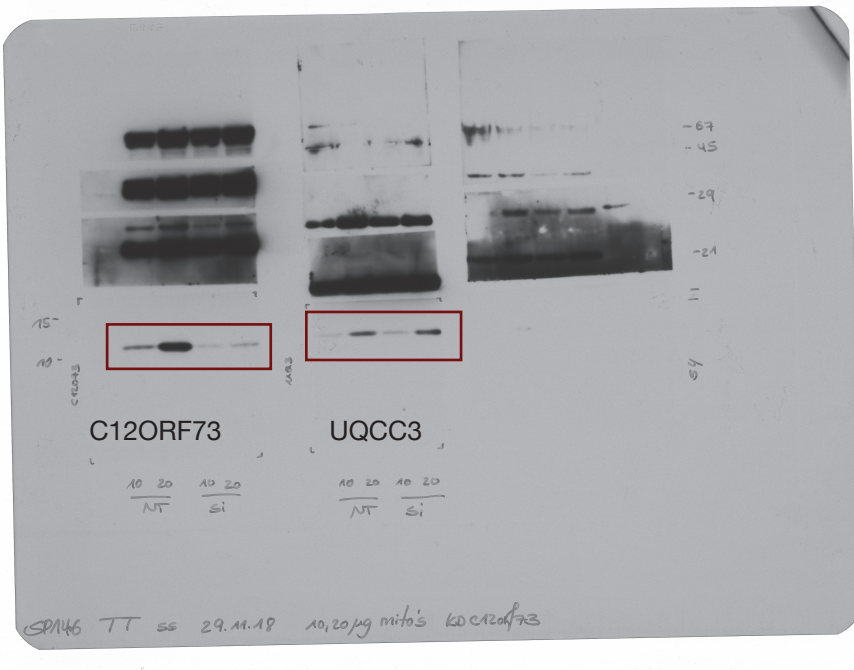

Supplement: Figure 5—figure supplement 1—source data 2. [file elife-68213-fig5-figsupp1-data2.zip › Figure_5_supplement_1_source_data_2/Figure_5_supplement_1_source_data_6_Figure_5_supplement_1G/Data_labelled /Figure_5_supplement_1_source_data_6_Figure_5_supplement_1G.pdf]

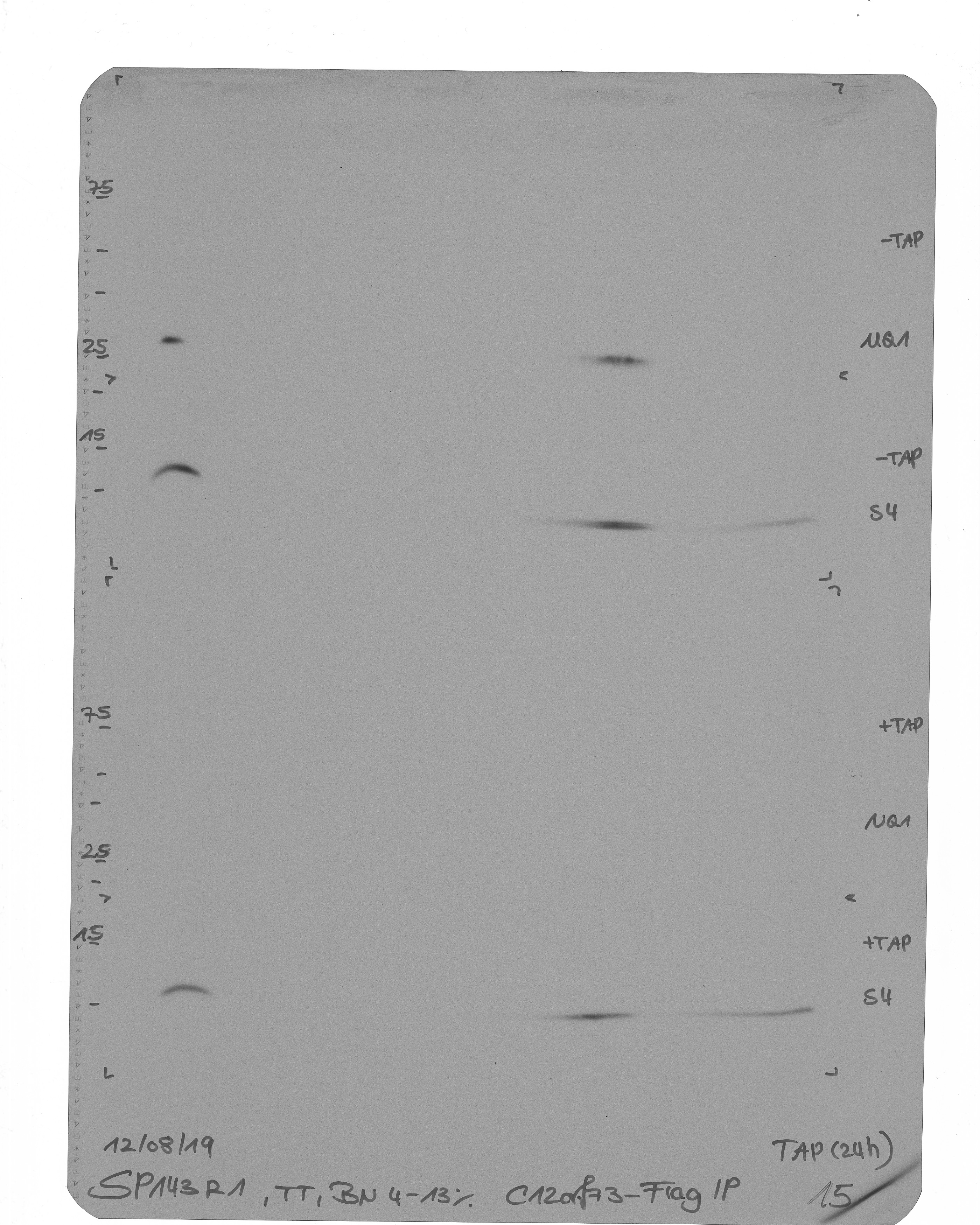

Supplement: Figure 6—source data 1. [file elife-68213-fig6-data1.zip › Figure_6_source_data/Figure_6_source_data_2_Figure_6B/Original_files/SP143R1 UQ1, SMIM4015.jpg]

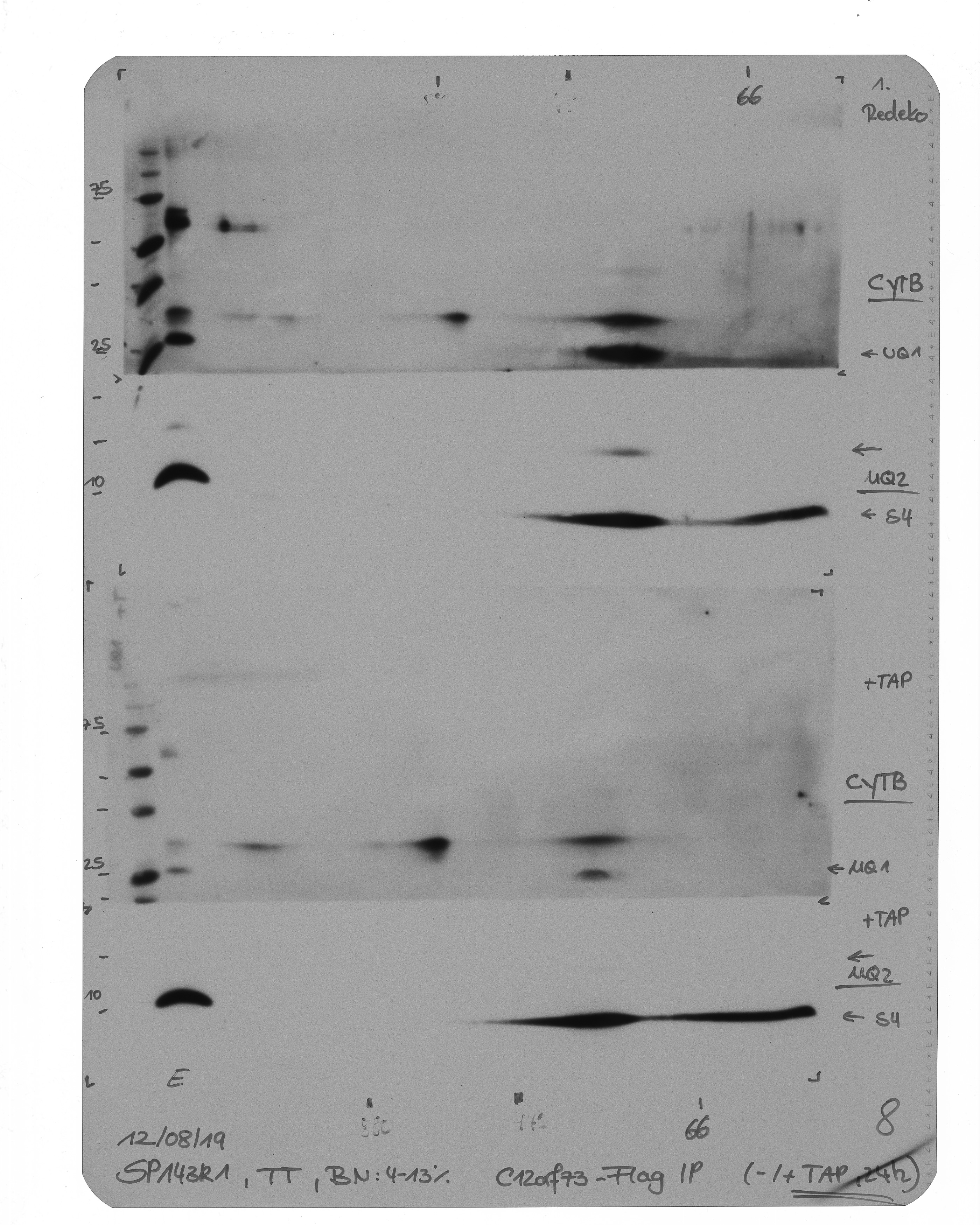

Supplement: Figure 6—source data 1. [file elife-68213-fig6-data1.zip › Figure_6_source_data/Figure_6_source_data_2_Figure_6B/Original_files/SP143R1 redeko cytb, uq2008.jpg]

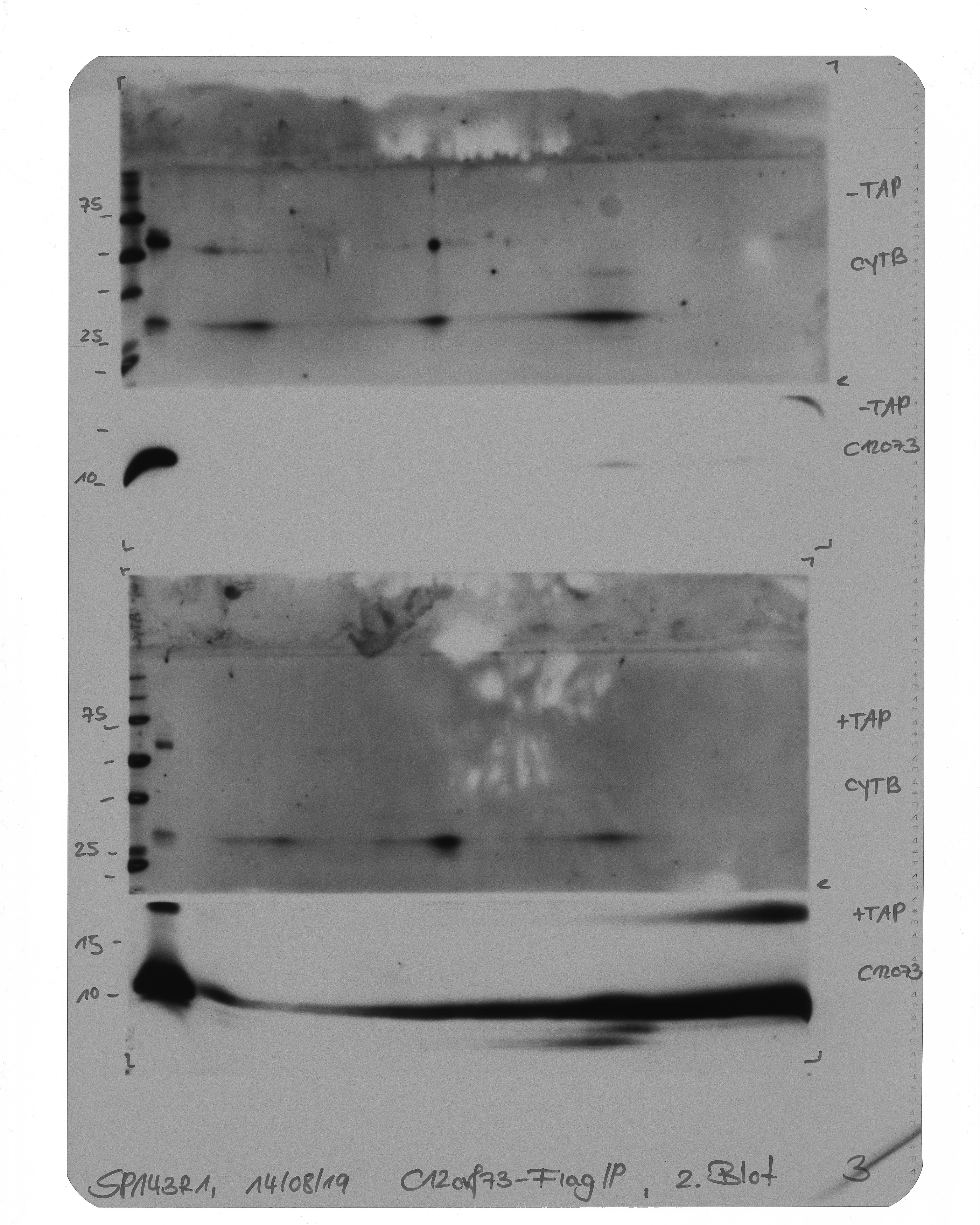

Supplement: Figure 6—source data 1. [file elife-68213-fig6-data1.zip › Figure_6_source_data/Figure_6_source_data_2_Figure_6B/Original_files/SP143R1 2.Blot003.jpg]

Figure\_6\_source\_data\_2\_Figure\_6B

UQCC1  
SMIM4

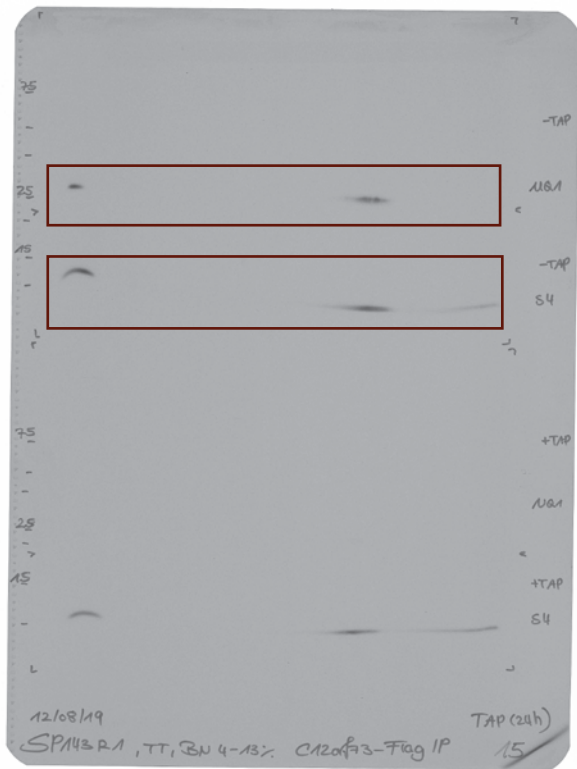

UQCC2

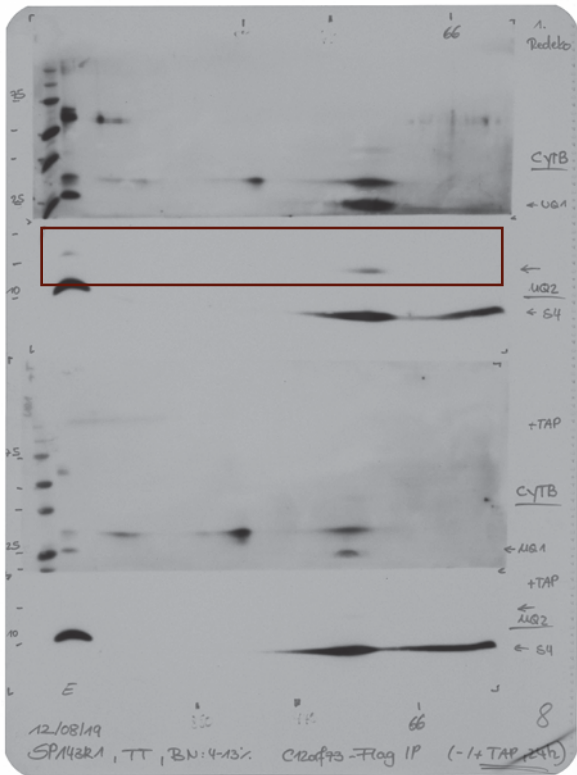

C12ORF73

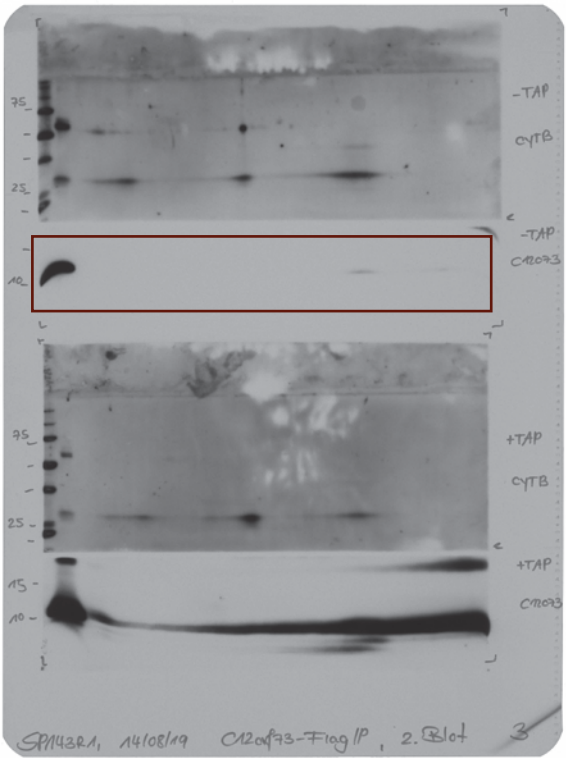

Supplement: Figure 6—source data 1. [file elife-68213-fig6-data1.zip › Figure_6_source_data/Figure_6_source_data_2_Figure_6B/Data_labelled/Figure_6_source_data_2_Figure_6B.pdf]

SDHA

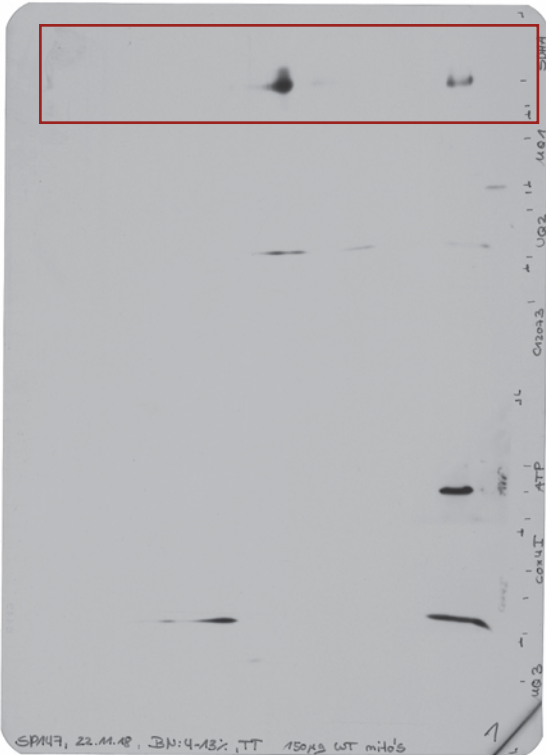

UQC2

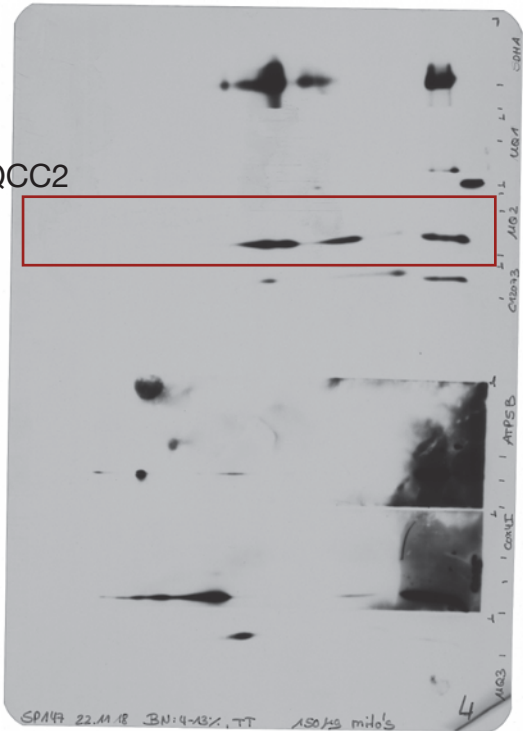

COX4I

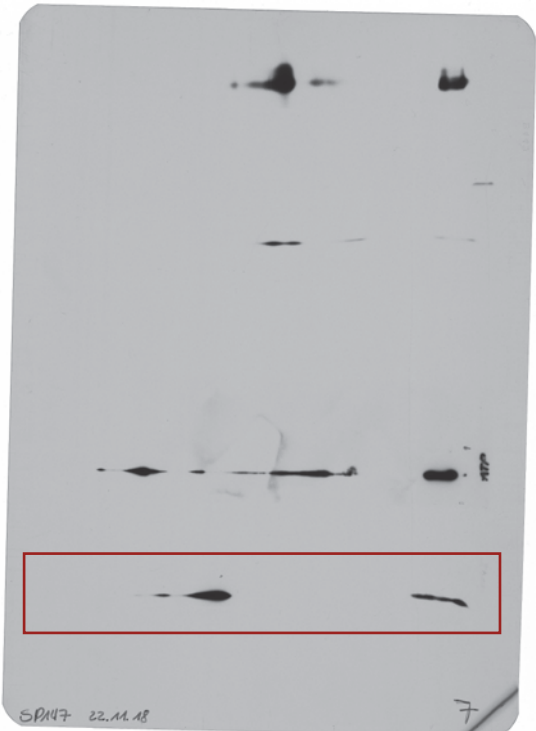

UQC3

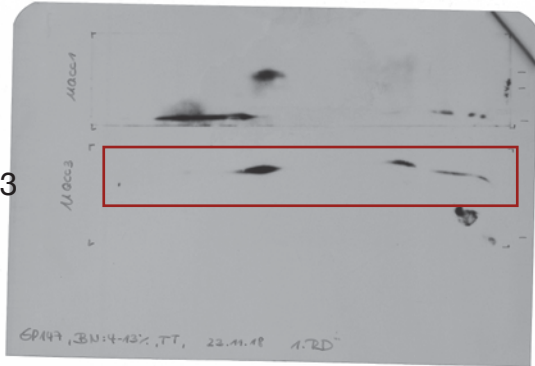

UQC1

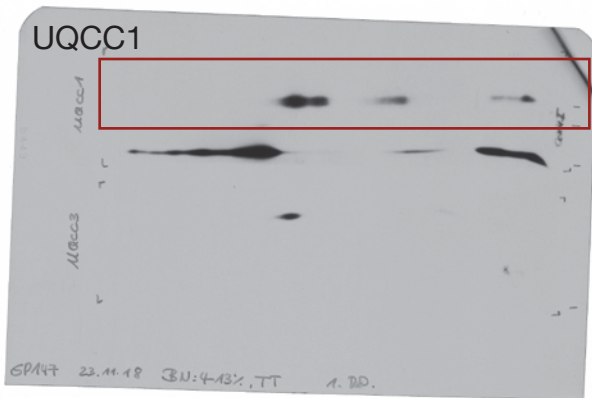

SMIM4

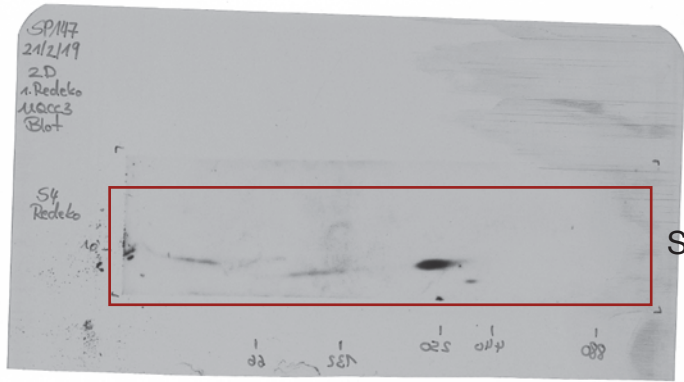

ATP5B

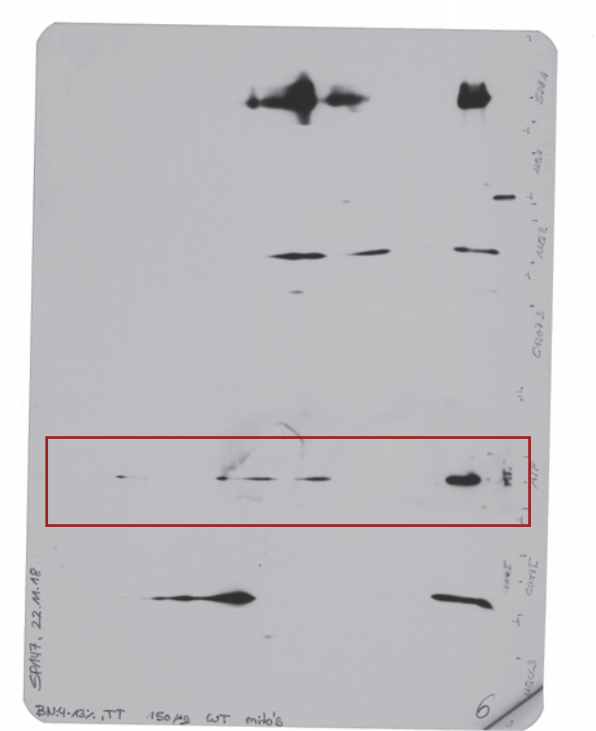

C12ORF73

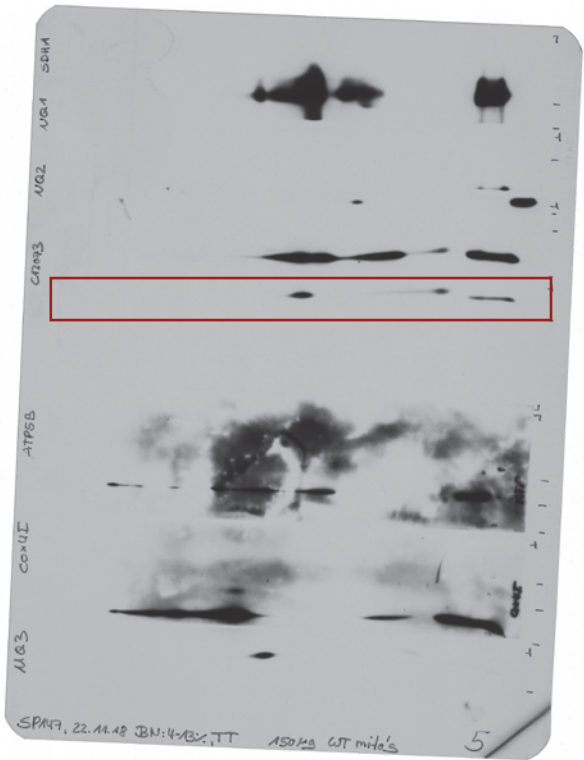

Supplement: Figure 6—source data 1. [file elife-68213-fig6-data1.zip › Figure_6_source_data/Figure _6_source_data _1_Figure_6A/Data_labelled/Figure _6_source_data _1_Figure_6A.pdf]

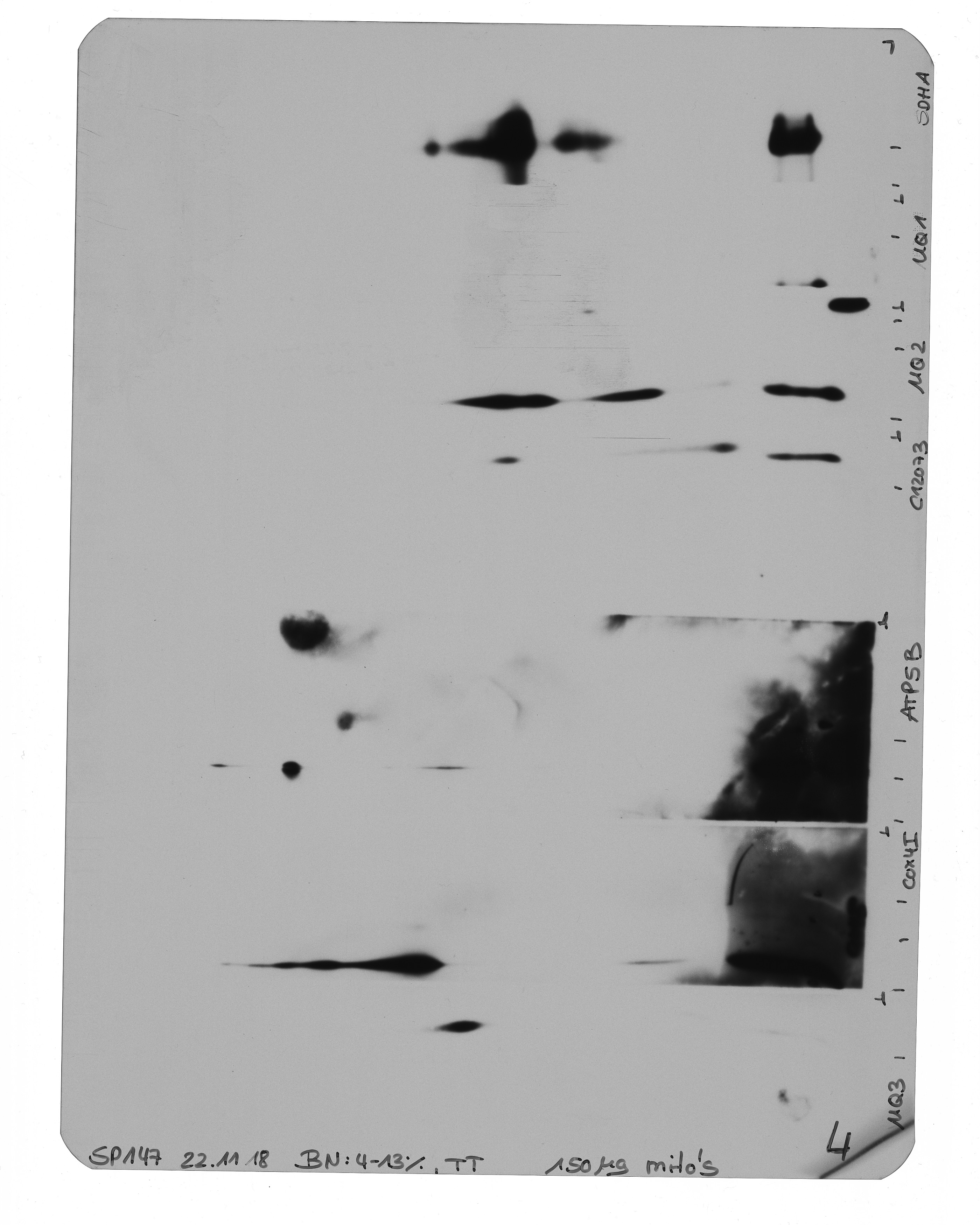

Supplement: Figure 6—source data 1. [file elife-68213-fig6-data1.zip › Figure_6_source_data/Figure _6_source_data _1_Figure_6A/Original_files/SP147_2d004.jpg]

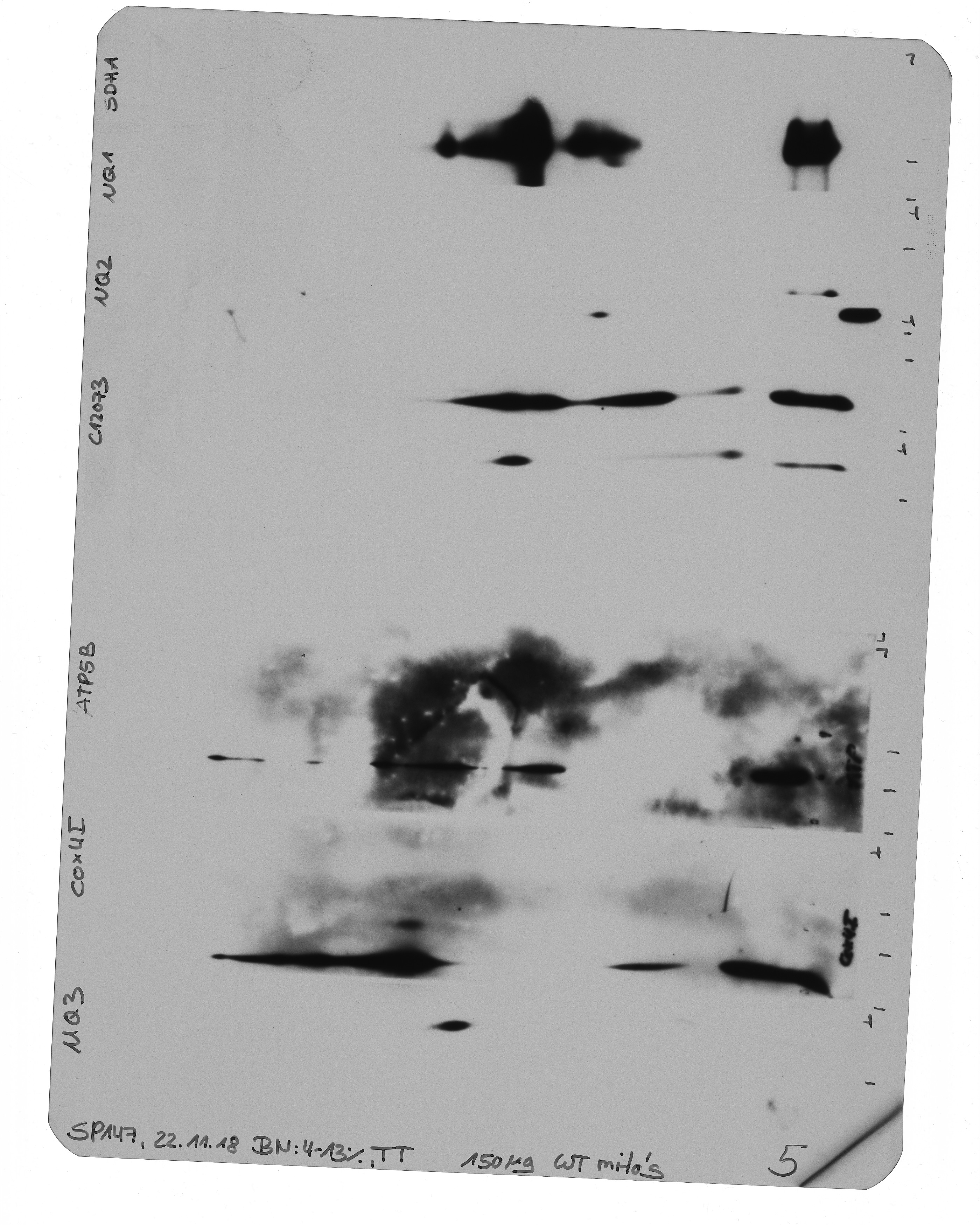

Supplement: Figure 6—source data 1. [file elife-68213-fig6-data1.zip › Figure_6_source_data/Figure _6_source_data _1_Figure_6A/Original_files/SP147_2d005.jpg]

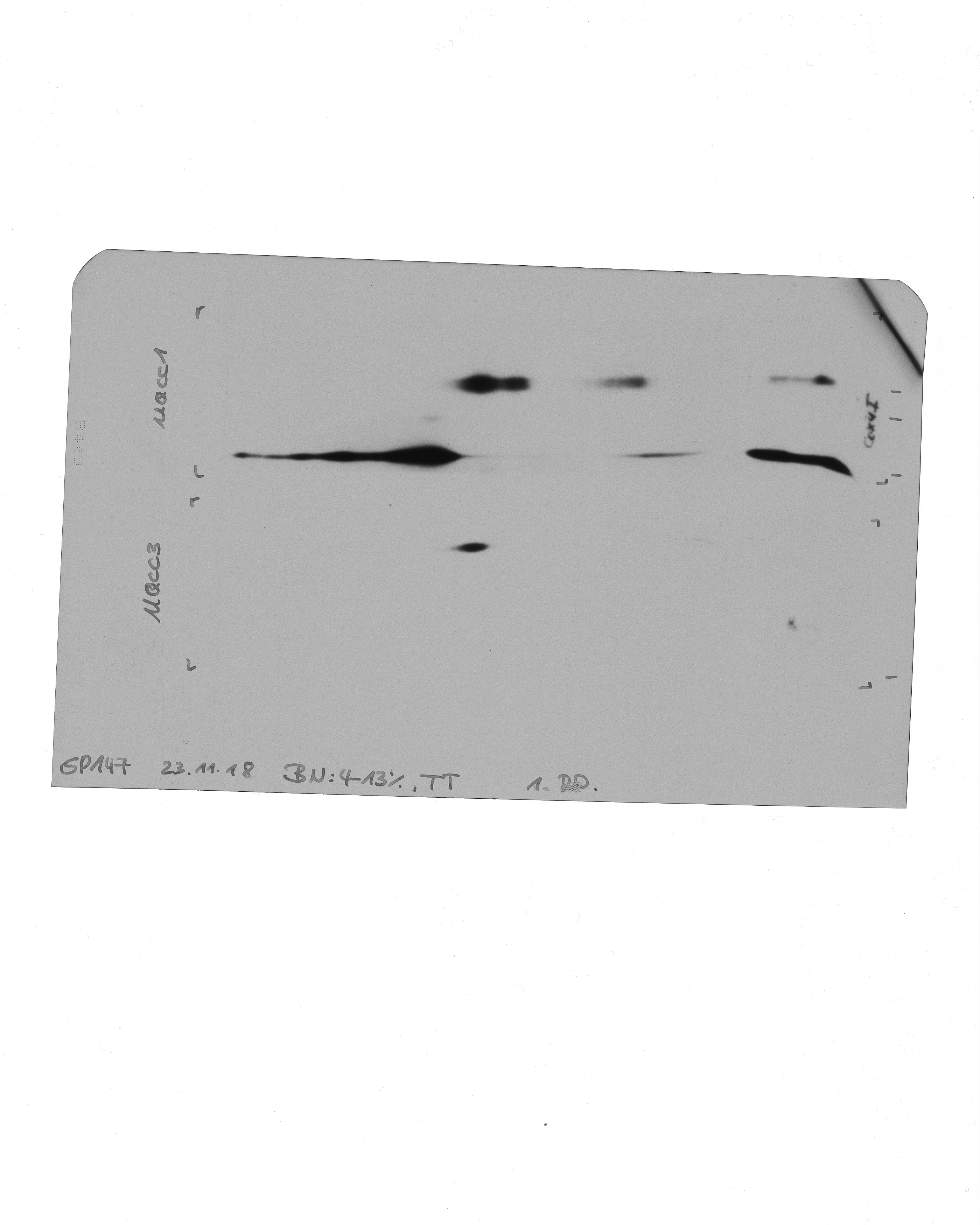

Supplement: Figure 6—source data 1. [file elife-68213-fig6-data1.zip › Figure_6_source_data/Figure _6_source_data _1_Figure_6A/Original_files/SP147_1RD002.jpg]

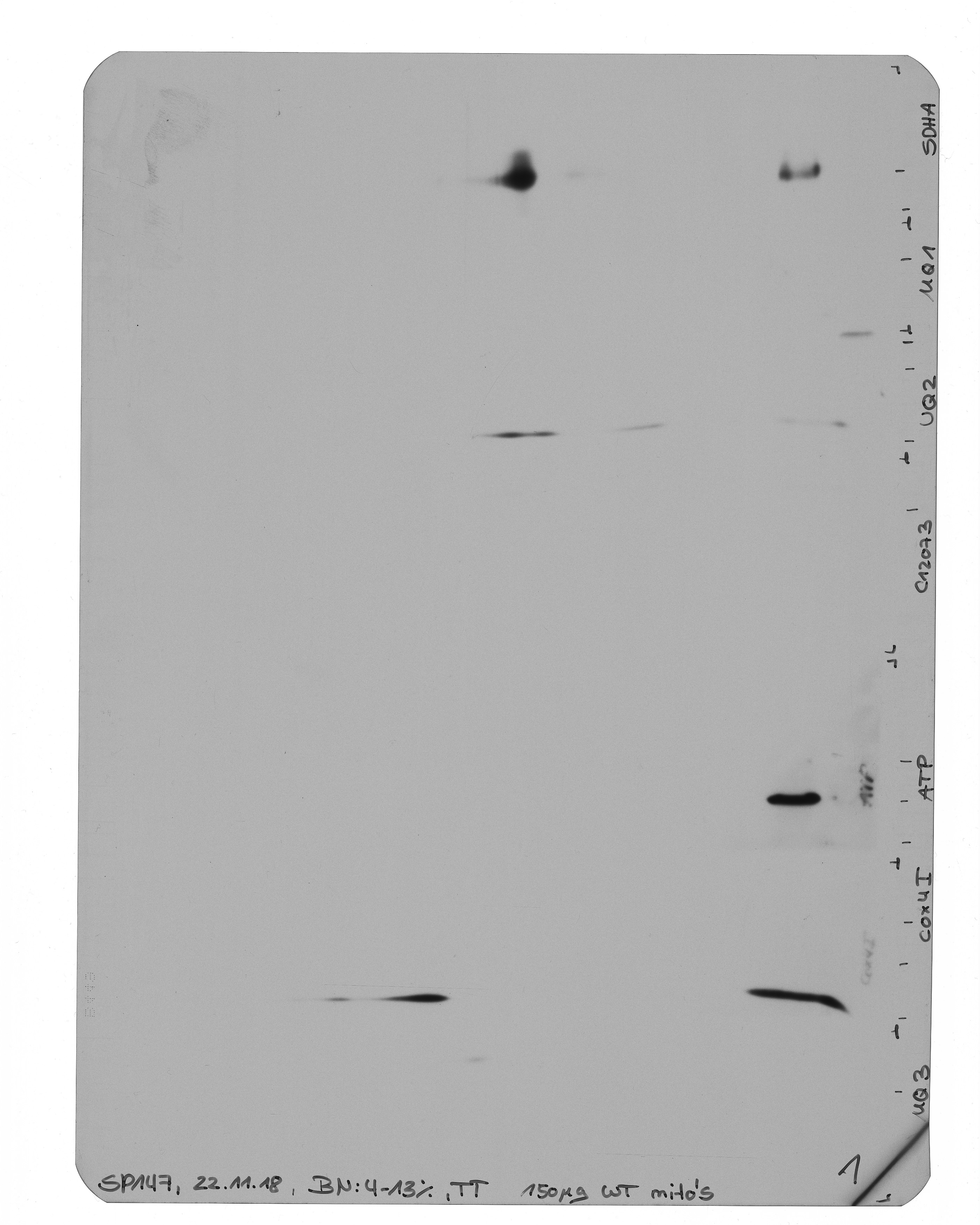

Supplement: Figure 6—source data 1. [file elife-68213-fig6-data1.zip › Figure_6_source_data/Figure _6_source_data _1_Figure_6A/Original_files/SP147_2d001.jpg]

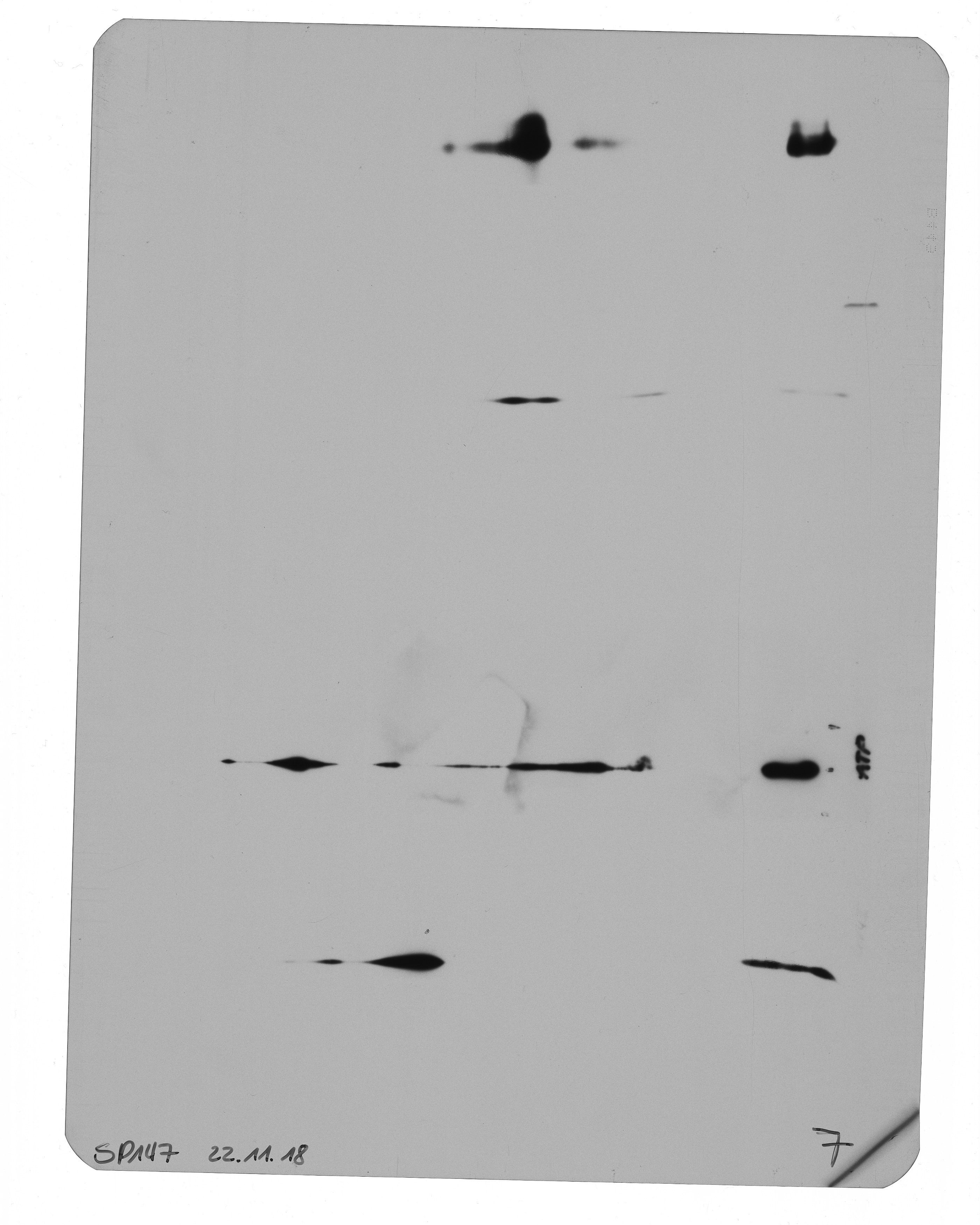

Supplement: Figure 6—source data 1. [file elife-68213-fig6-data1.zip › Figure_6_source_data/Figure _6_source_data _1_Figure_6A/Original_files/SP147_2d007.jpg]

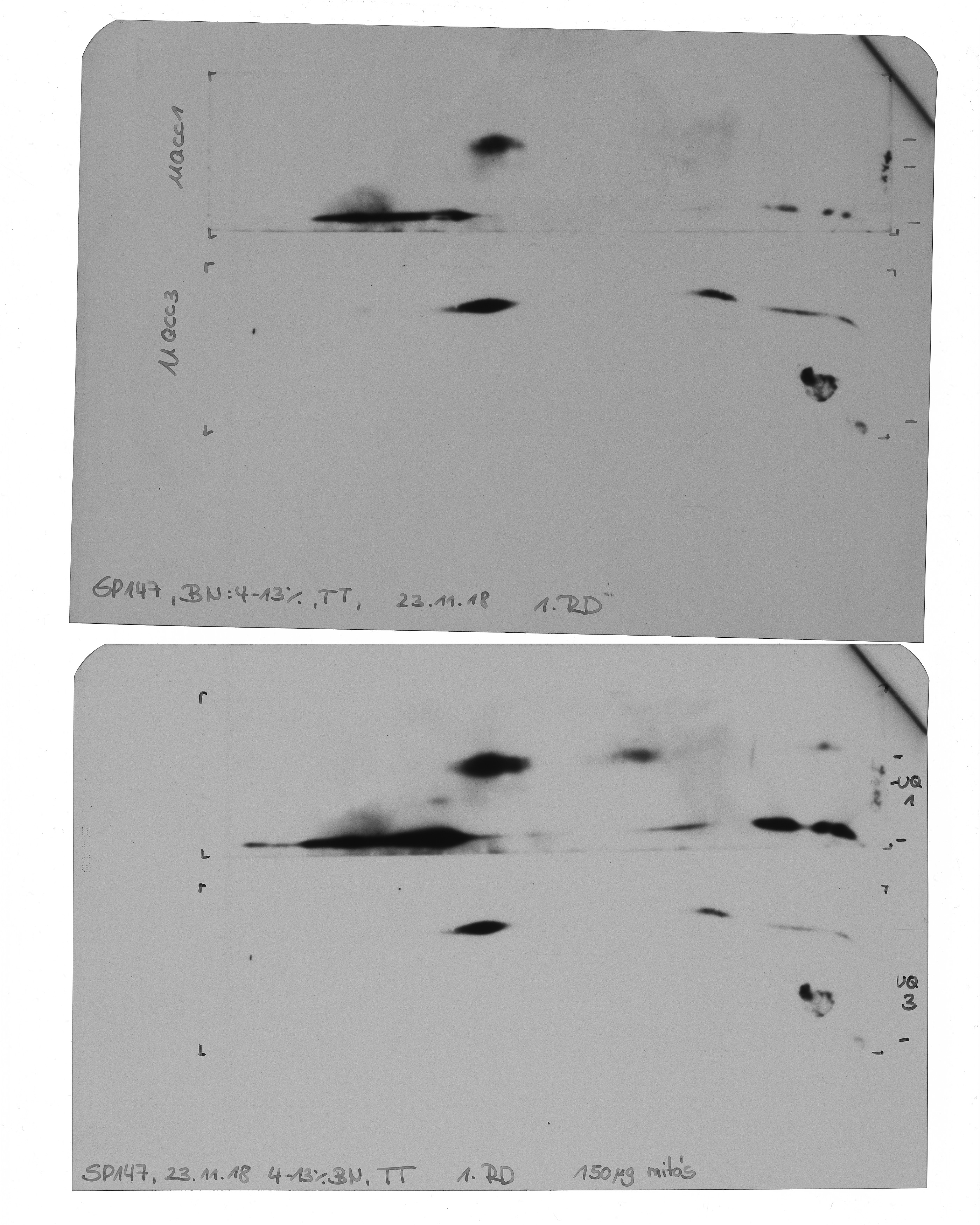

Supplement: Figure 6—source data 1. [file elife-68213-fig6-data1.zip › Figure_6_source_data/Figure _6_source_data _1_Figure_6A/Original_files/SP147_1RD001.jpg]

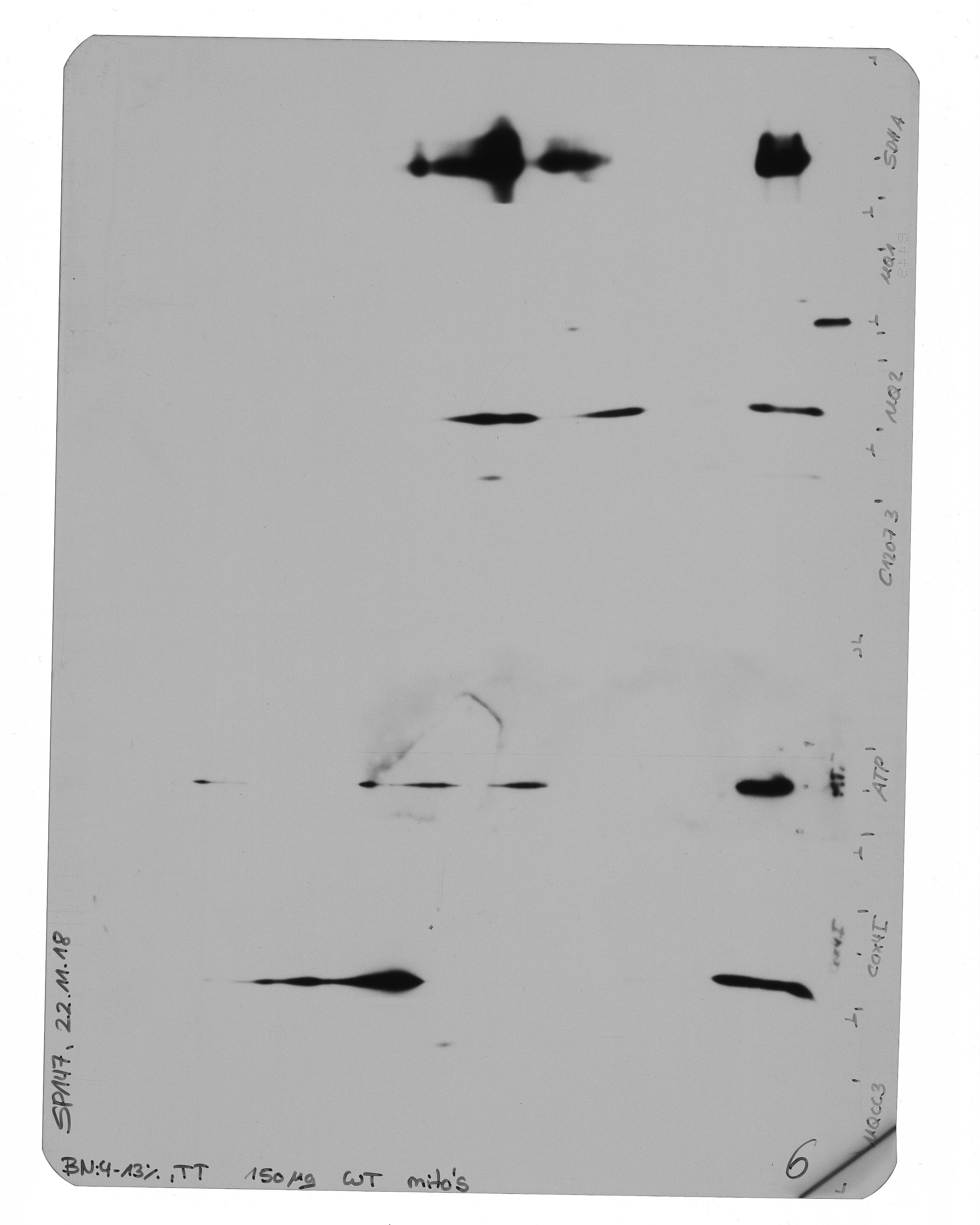

Supplement: Figure 6—source data 1. [file elife-68213-fig6-data1.zip › Figure_6_source_data/Figure _6_source_data _1_Figure_6A/Original_files/SP147_2d006.jpg]

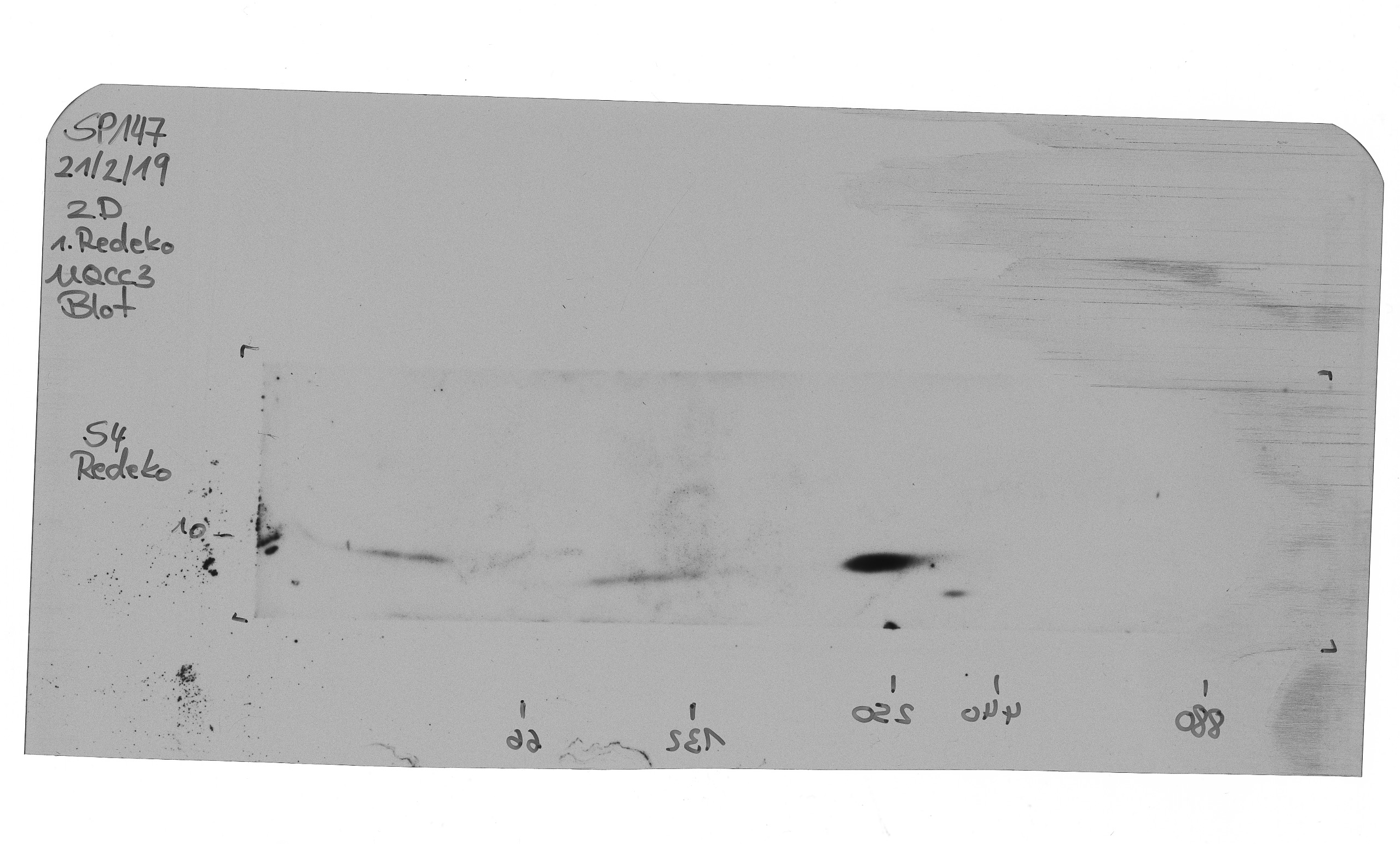

Supplement: Figure 6—source data 1. [file elife-68213-fig6-data1.zip › Figure_6_source_data/Figure _6_source_data _1_Figure_6A/Original_files/SP147 Redeko SMIM4.jpg]

Figure\_6\_source\_data\_6\_Figure\_6E

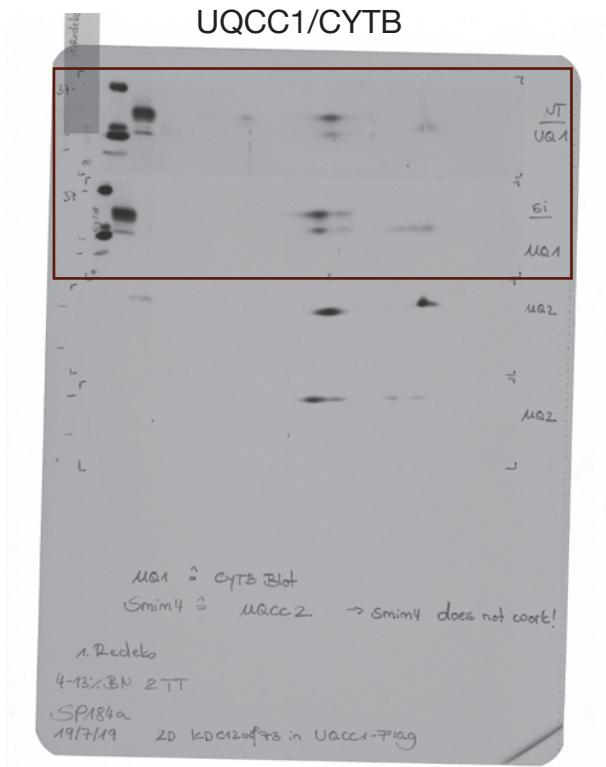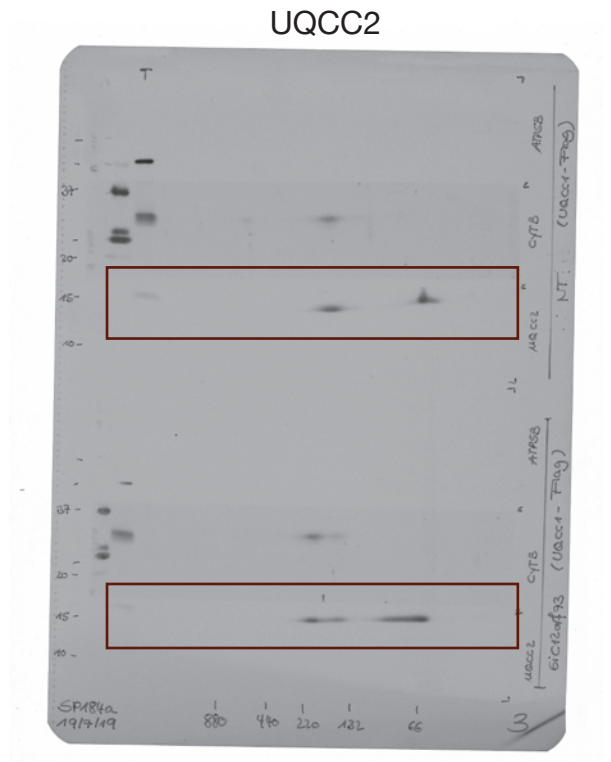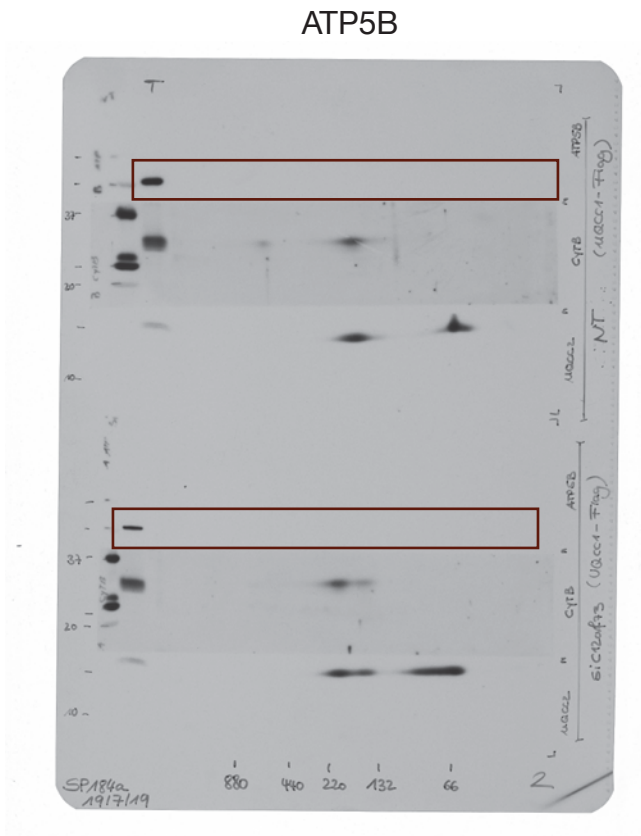

Supplement: Figure 6—source data 1. [file elife-68213-fig6-data1.zip › Figure_6_source_data/Figure_6_source_data_6_Figure_6E/Data_labelled/Figure_6_source_data_6_Figure_6E.pdf]

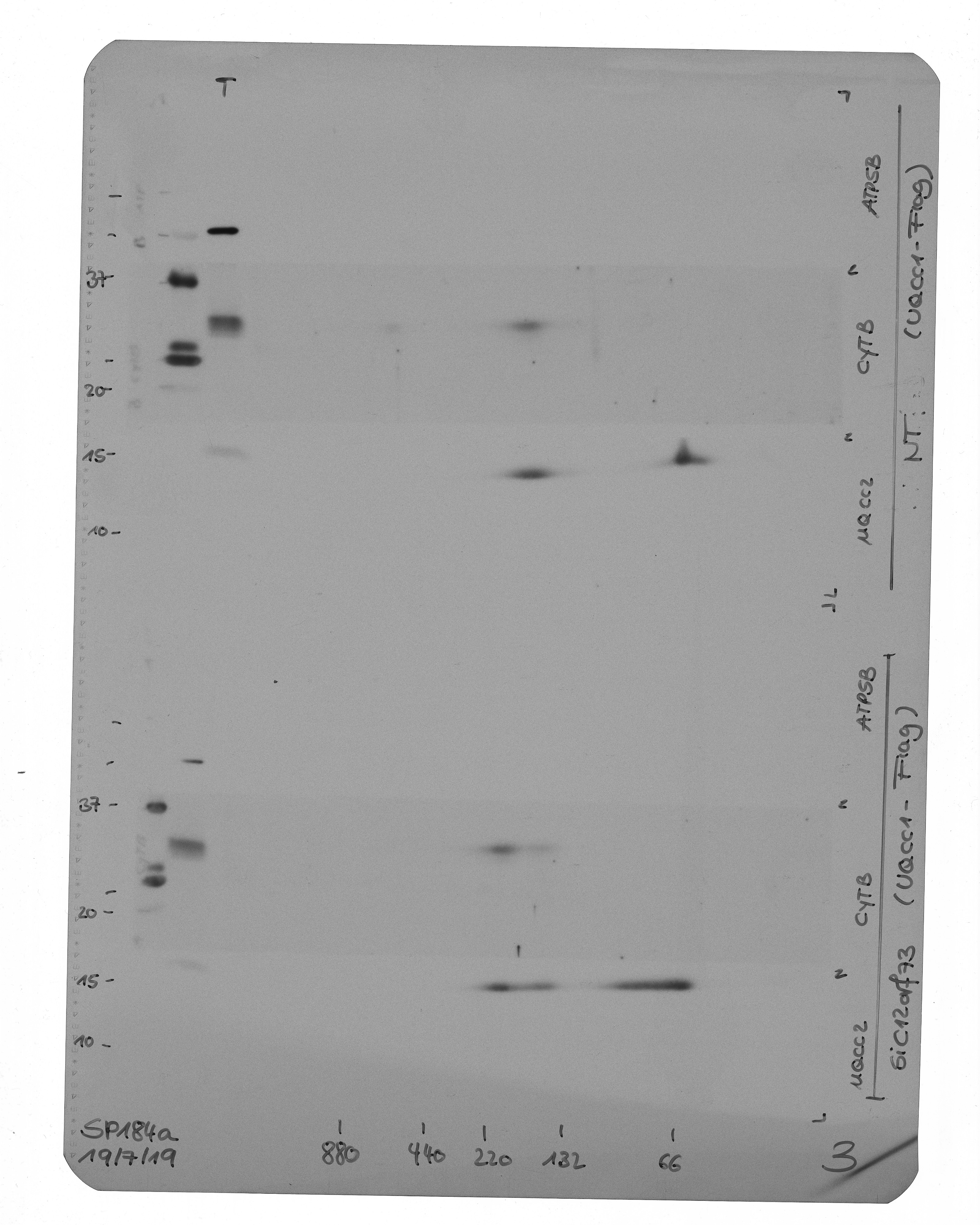

Supplement: Figure 6—source data 1. [file elife-68213-fig6-data1.zip › Figure_6_source_data/Figure_6_source_data_6_Figure_6E/Original_files/SP184a003.jpg]

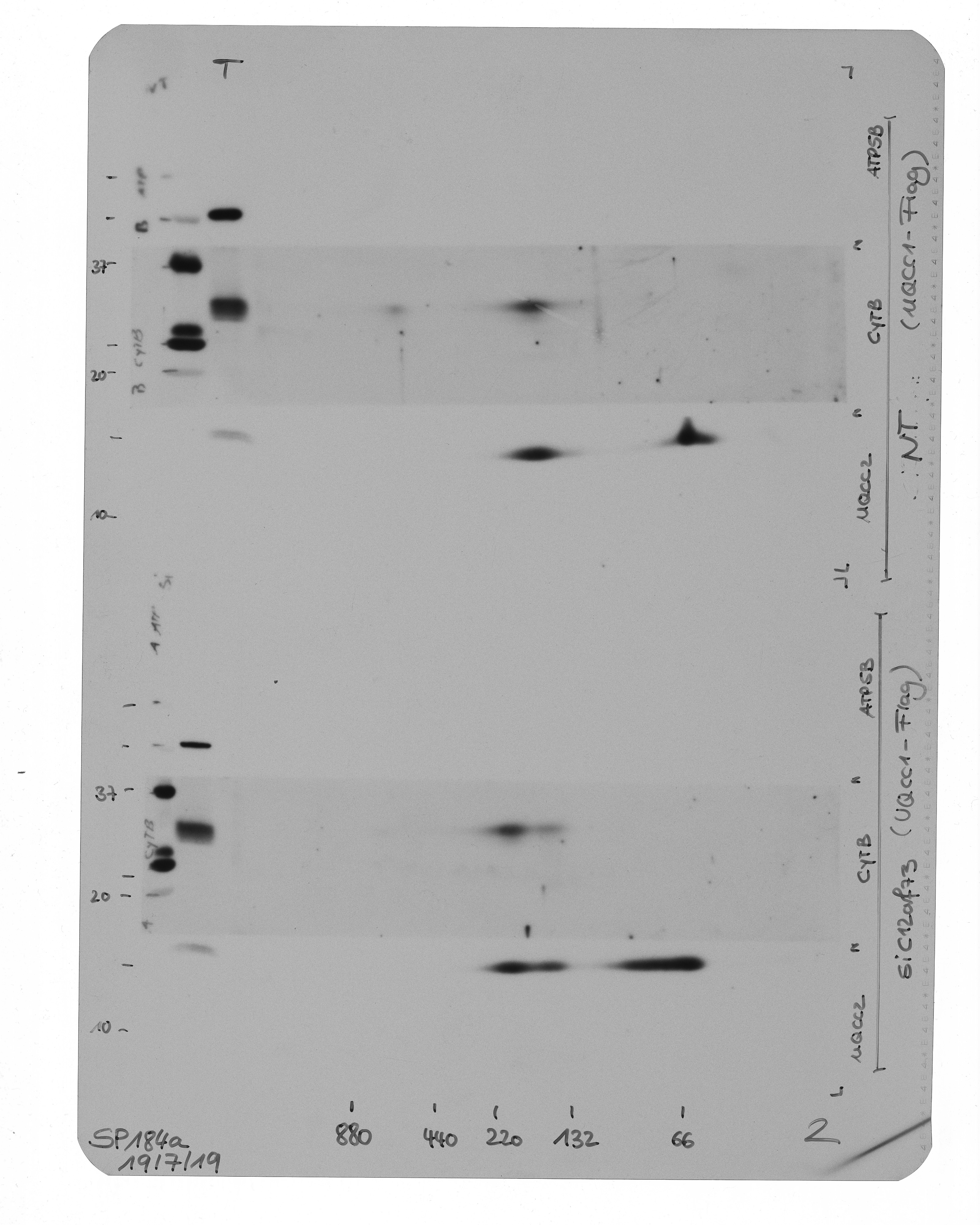

Supplement: Figure 6—source data 1. [file elife-68213-fig6-data1.zip › Figure_6_source_data/Figure_6_source_data_6_Figure_6E/Original_files/SP184a002.jpg]

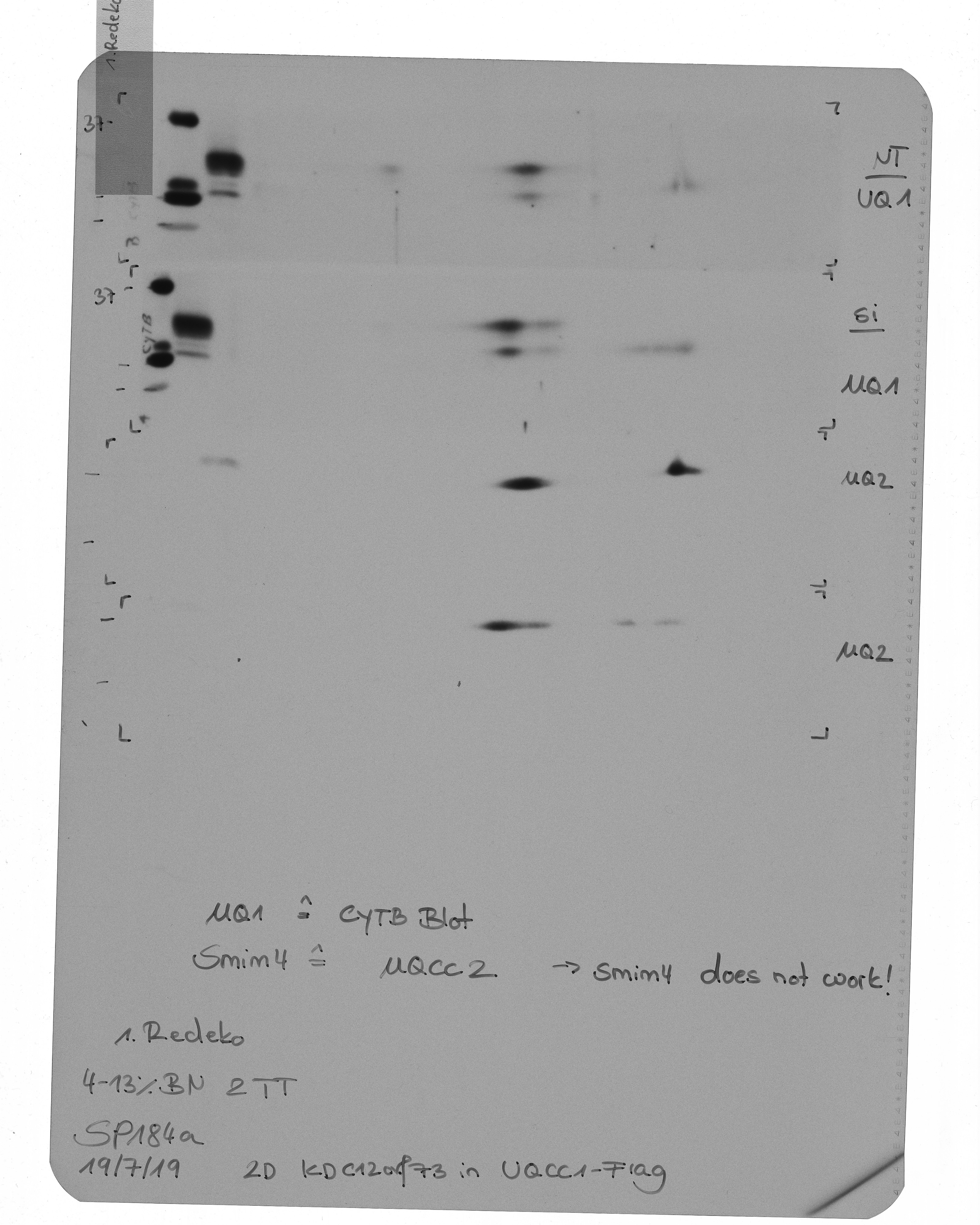

Supplement: Figure 6—source data 1. [file elife-68213-fig6-data1.zip › Figure_6_source_data/Figure_6_source_data_6_Figure_6E/Original_files/SP184a001.jpg]

Figure\_6\_source\_data\_3\_Figure\_6C

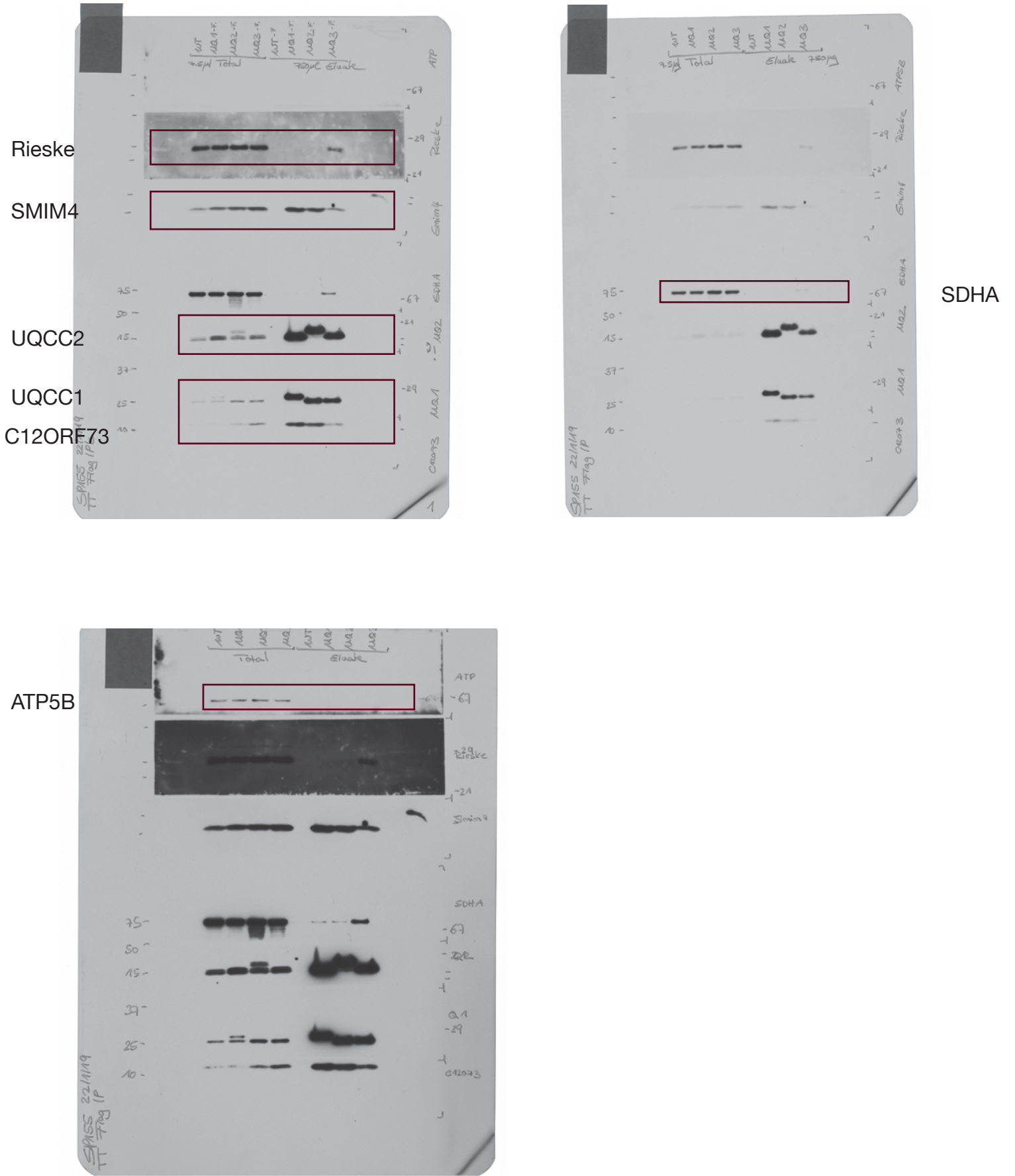

Supplement: Figure 6—source data 1. [file elife-68213-fig6-data1.zip › Figure_6_source_data/Figure_6_source_data_3_Figure_6C/Data_labelled/Figure_6_source_data_3_Figure_6C.pdf]

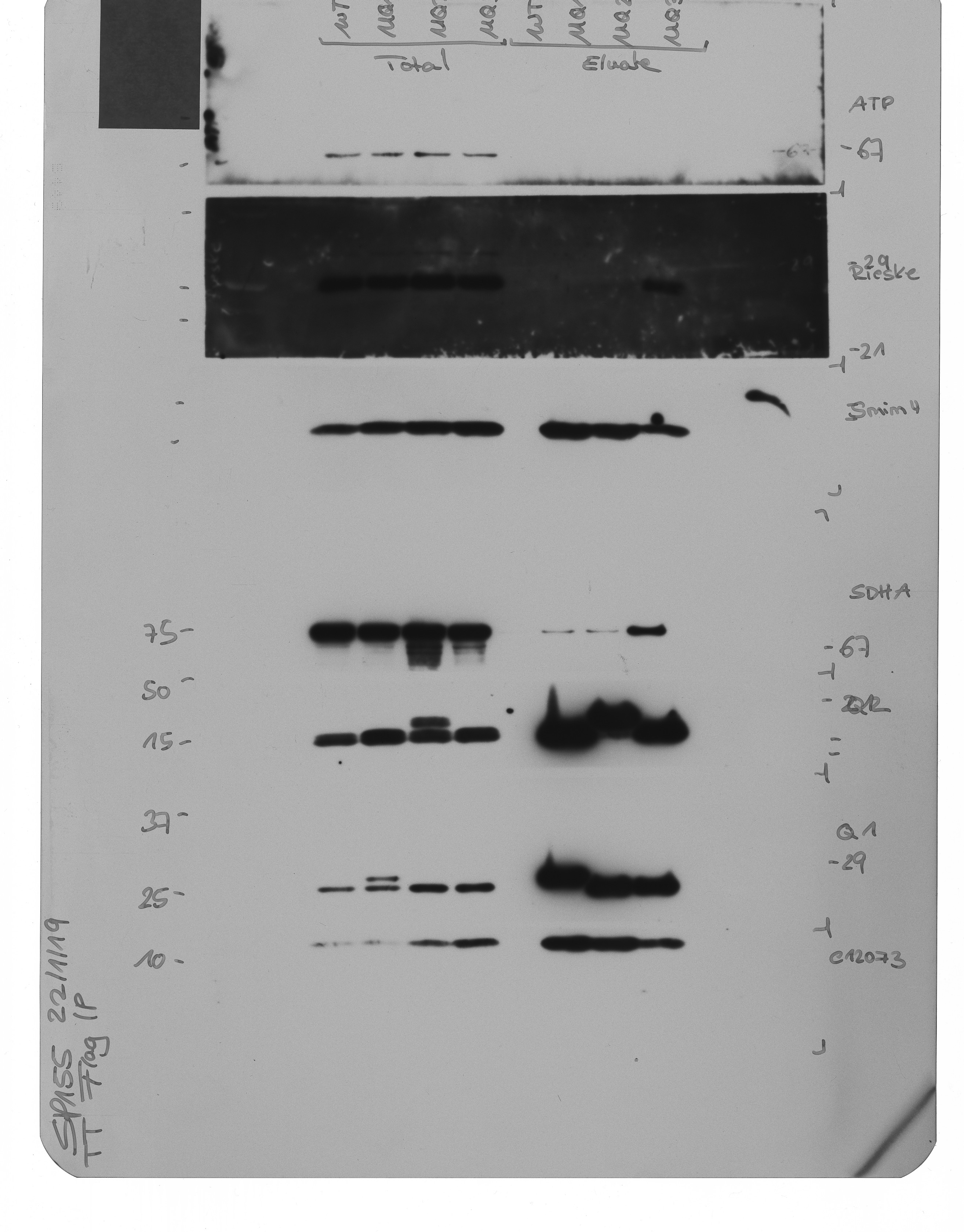

Supplement: Figure 6—source data 1. [file elife-68213-fig6-data1.zip › Figure_6_source_data/Figure_6_source_data_3_Figure_6C/Original_files/UQS FLAG IP003.jpg]

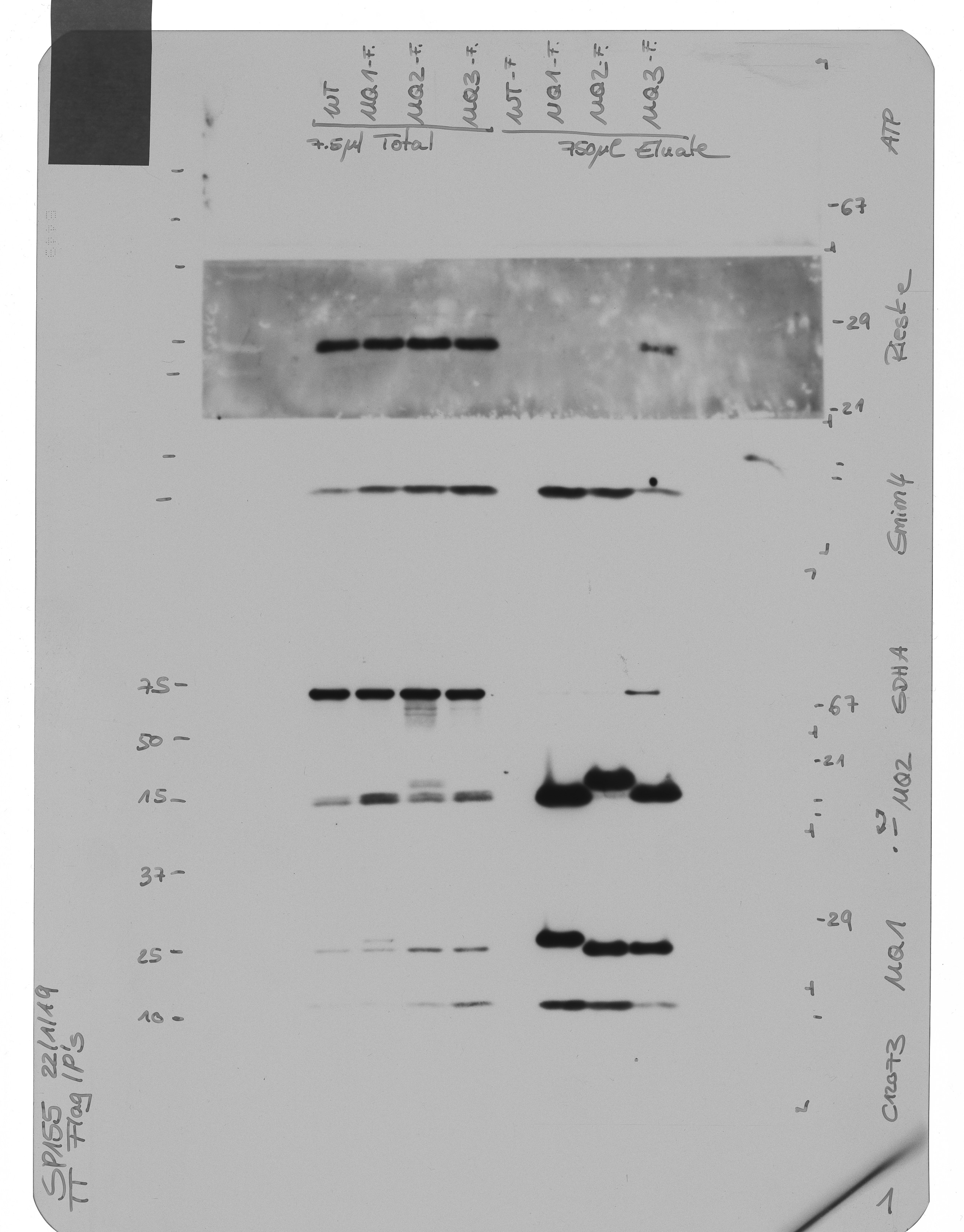

Supplement: Figure 6—source data 1. [file elife-68213-fig6-data1.zip › Figure_6_source_data/Figure_6_source_data_3_Figure_6C/Original_files/UQS FLAG IP002.jpg]

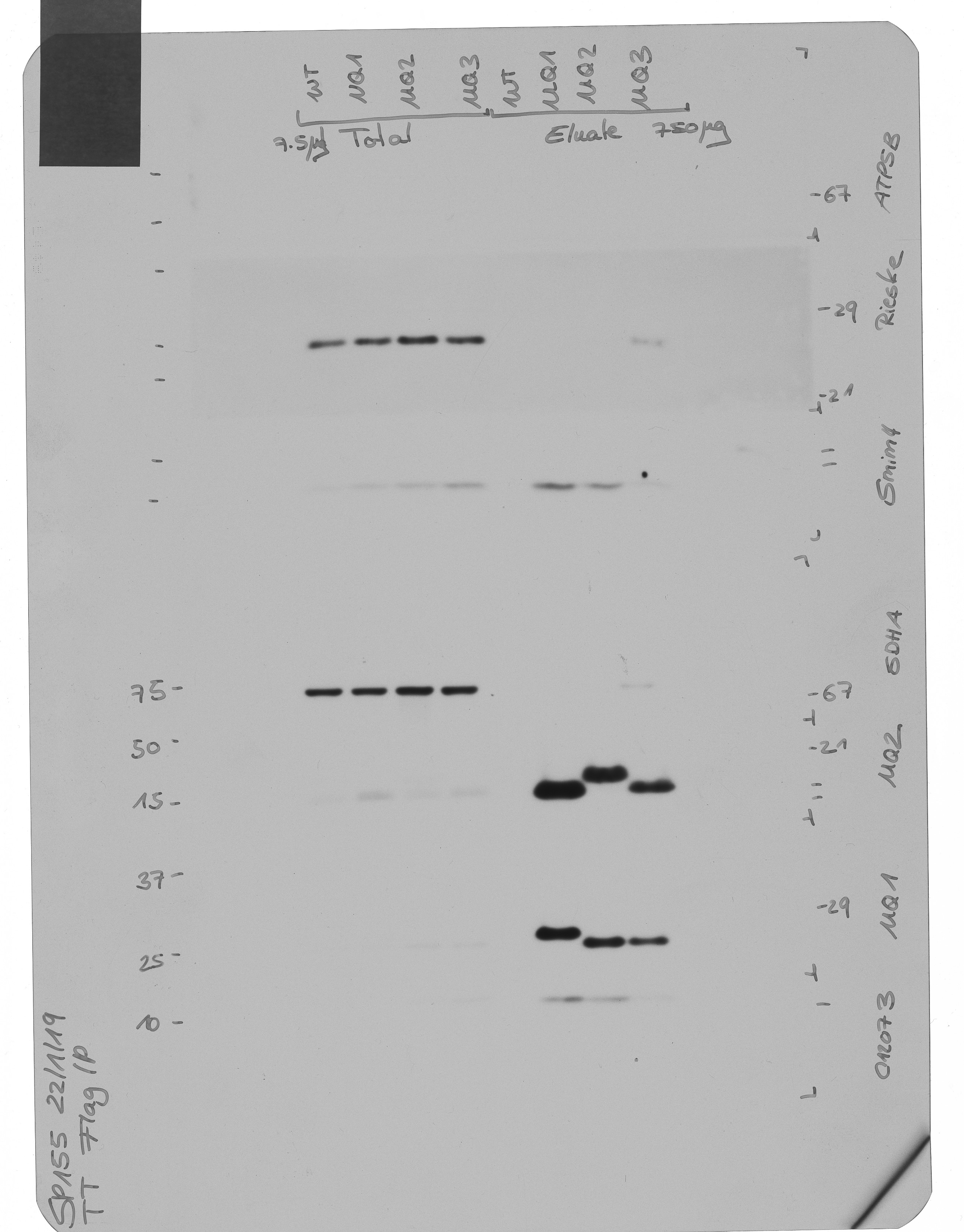

Supplement: Figure 6—source data 1. [file elife-68213-fig6-data1.zip › Figure_6_source_data/Figure_6_source_data_3_Figure_6C/Original_files/UQS FLAG IP001.jpg]

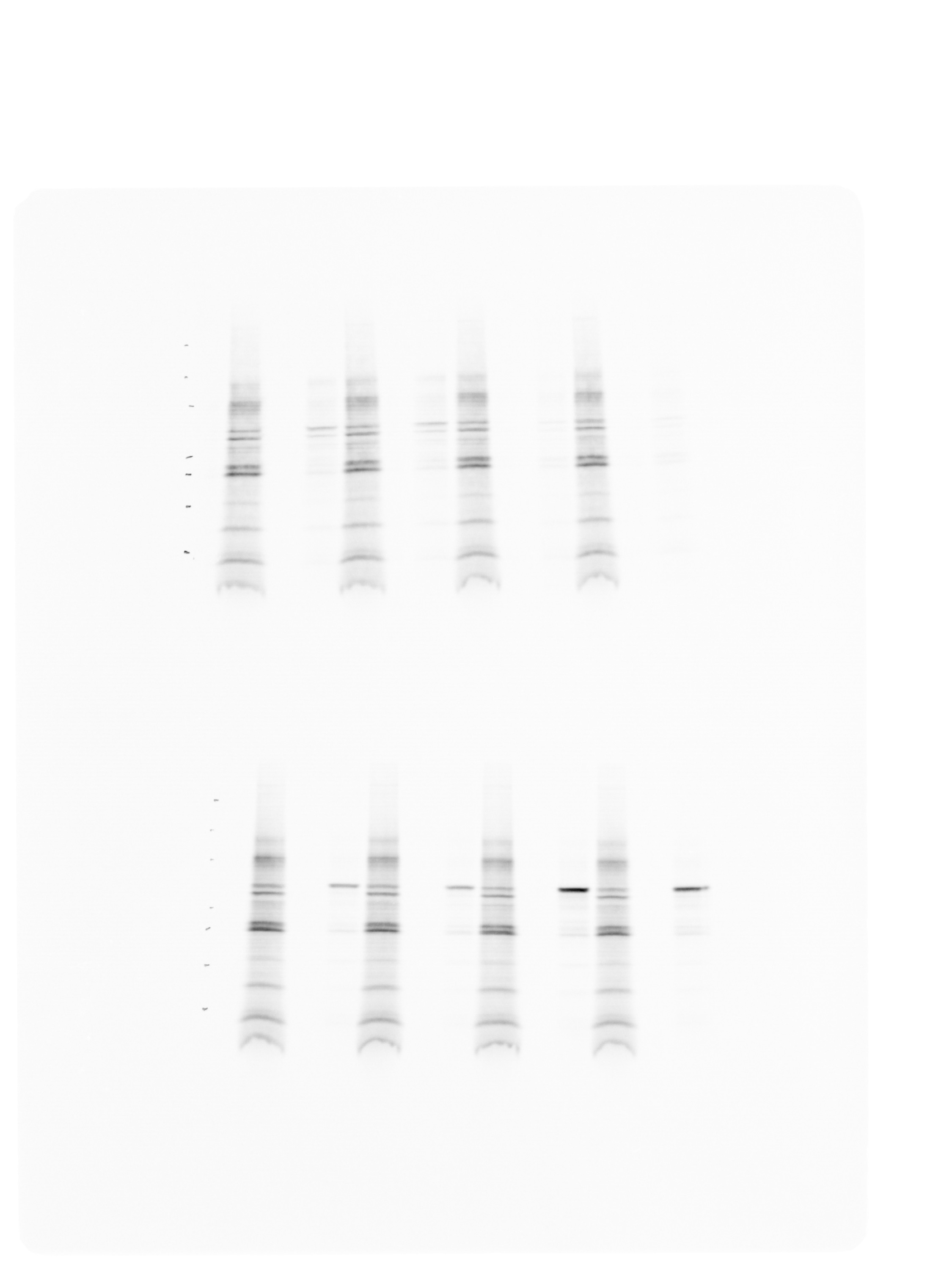

Supplement: Figure 6—source data 1. [file elife-68213-fig6-data1.zip › Figure_6_source_data/Figure_6_source_data_4_Figure_6D/Original_data/20190805-104305-[Phosphor].jpg]

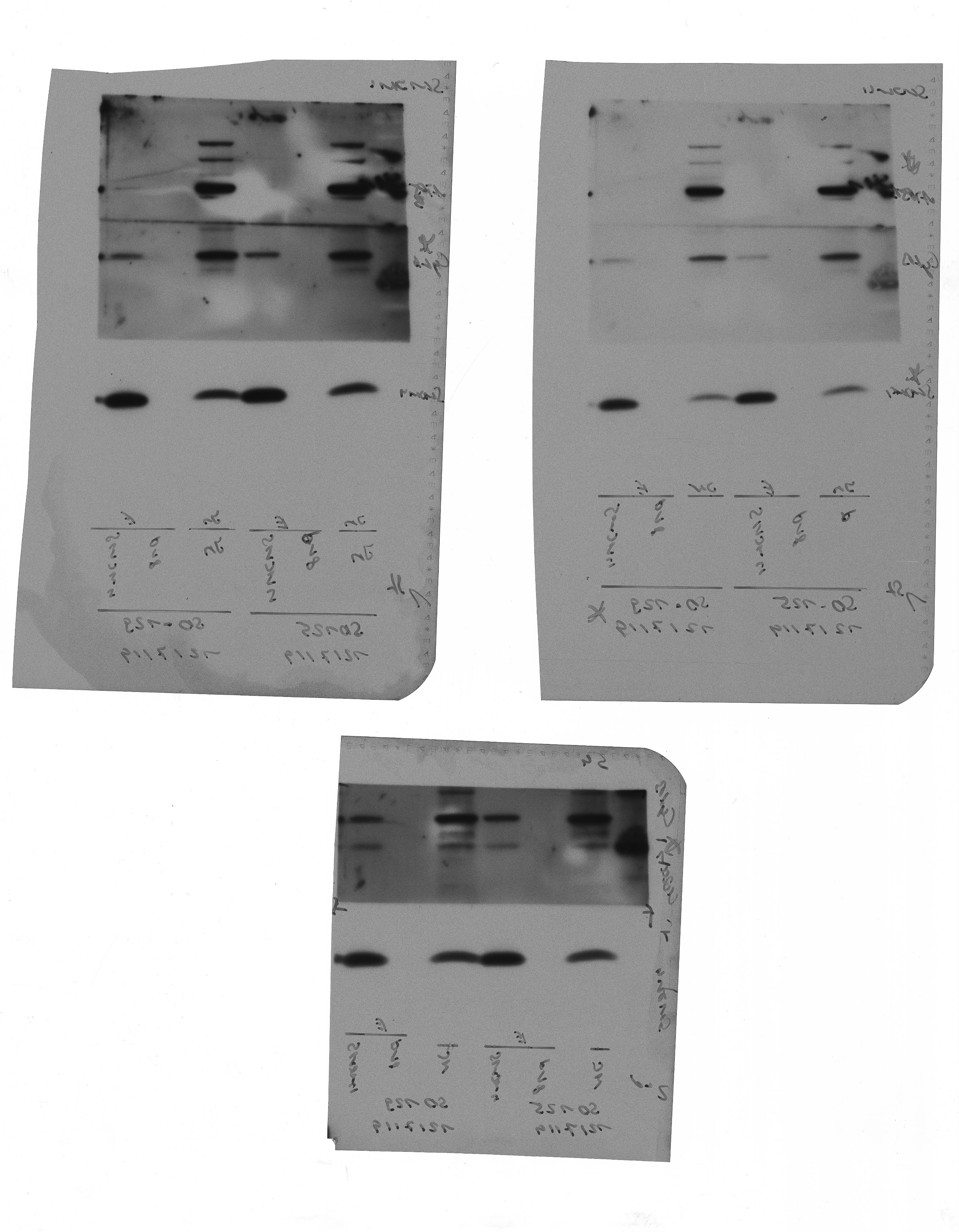

Supplement: Figure 6—source data 1. [file elife-68213-fig6-data1.zip › Figure_6_source_data/Figure_6_source_data_4_Figure_6D/Original_data/all002.jpg]

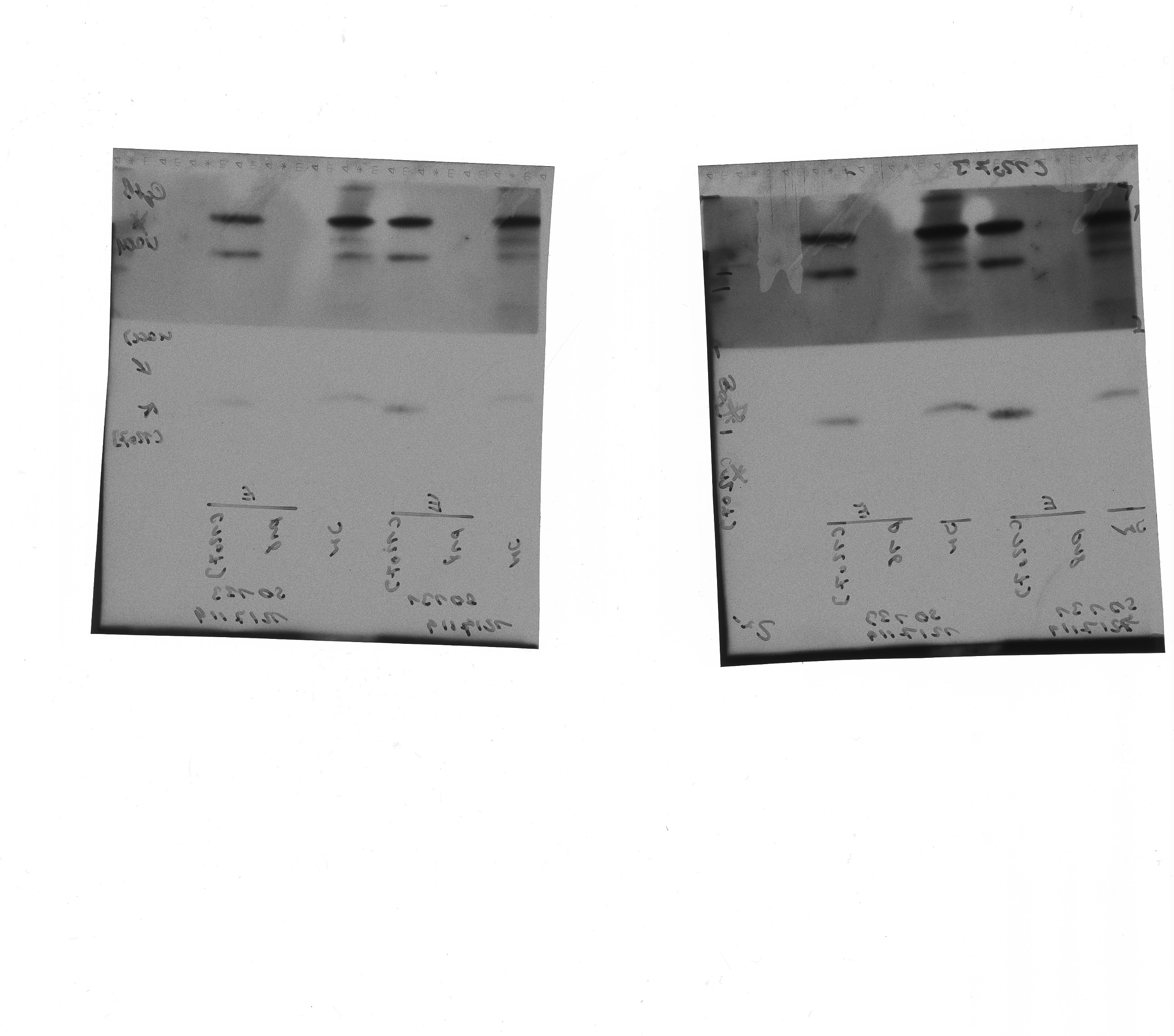

Supplement: Figure 6—source data 1. [file elife-68213-fig6-data1.zip › Figure_6_source_data/Figure_6_source_data_4_Figure_6D/Original_data/1007.jpg]

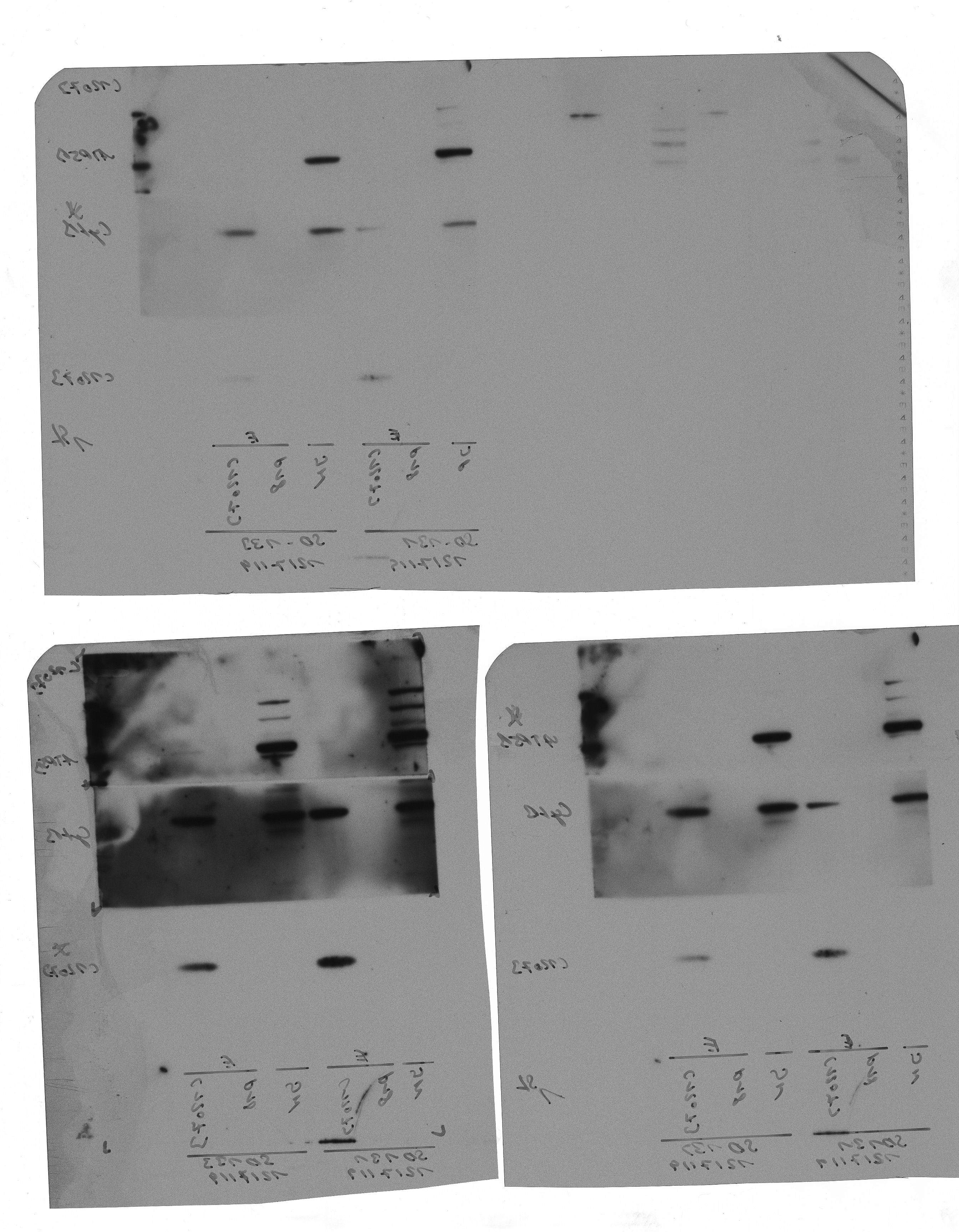

Supplement: Figure 6—source data 1. [file elife-68213-fig6-data1.zip › Figure_6_source_data/Figure_6_source_data_4_Figure_6D/Original_data/1003.jpg]
