# Supplementary material for: Defining the interactome of the human mitochondrial ribosome identifies SMIM4 and TMEM223 as respiratory chain assembly factors
Source: eLife. 2021 Dec 31;10:e68213. doi: 10.7554/eLife.68213 (PMC8719881; doi:10.7554/eLife.68213)

Figure 6 source data 4 related to Figure 6D

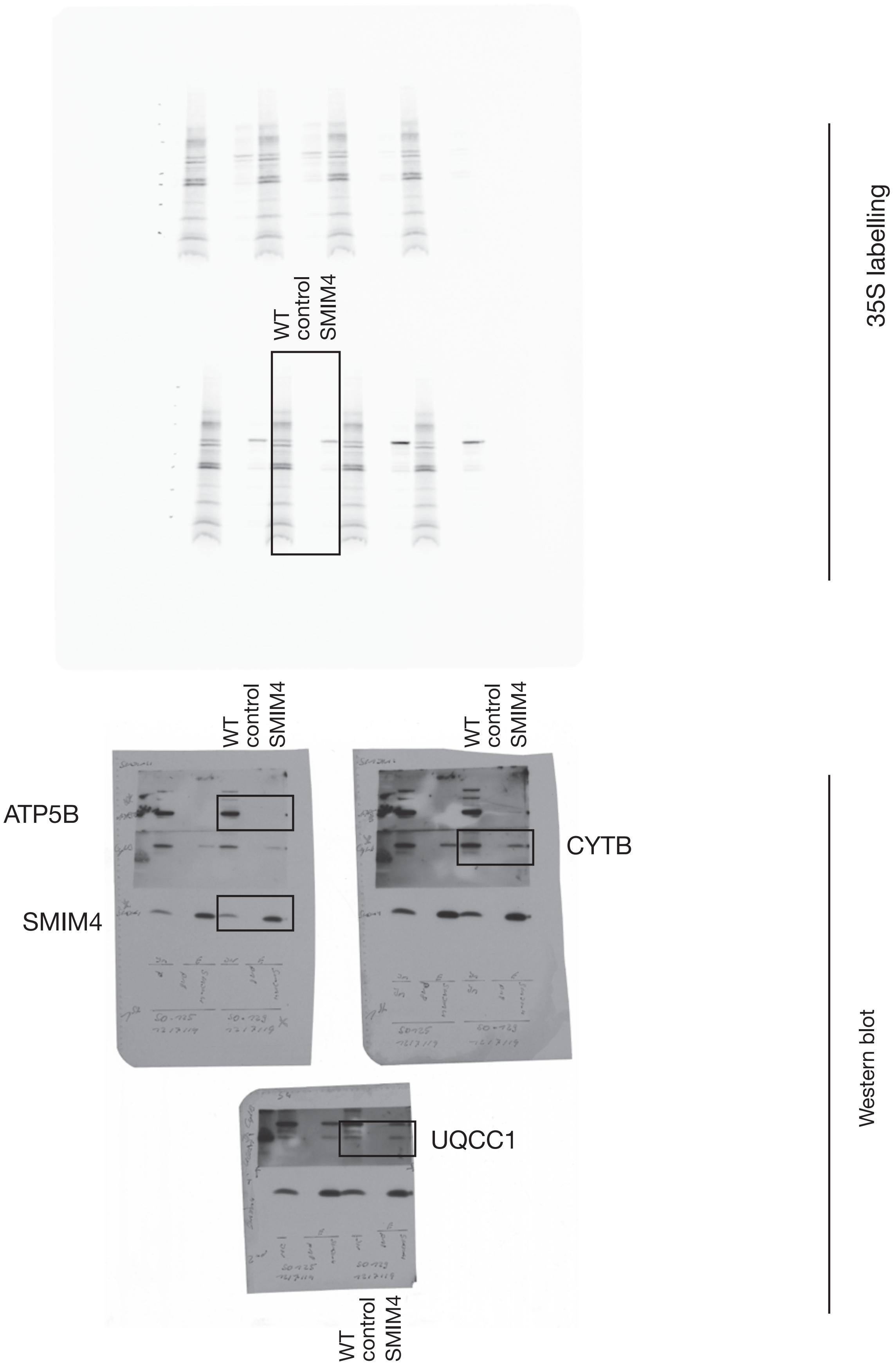

Figure 6 source data 4 related to Figure 6D

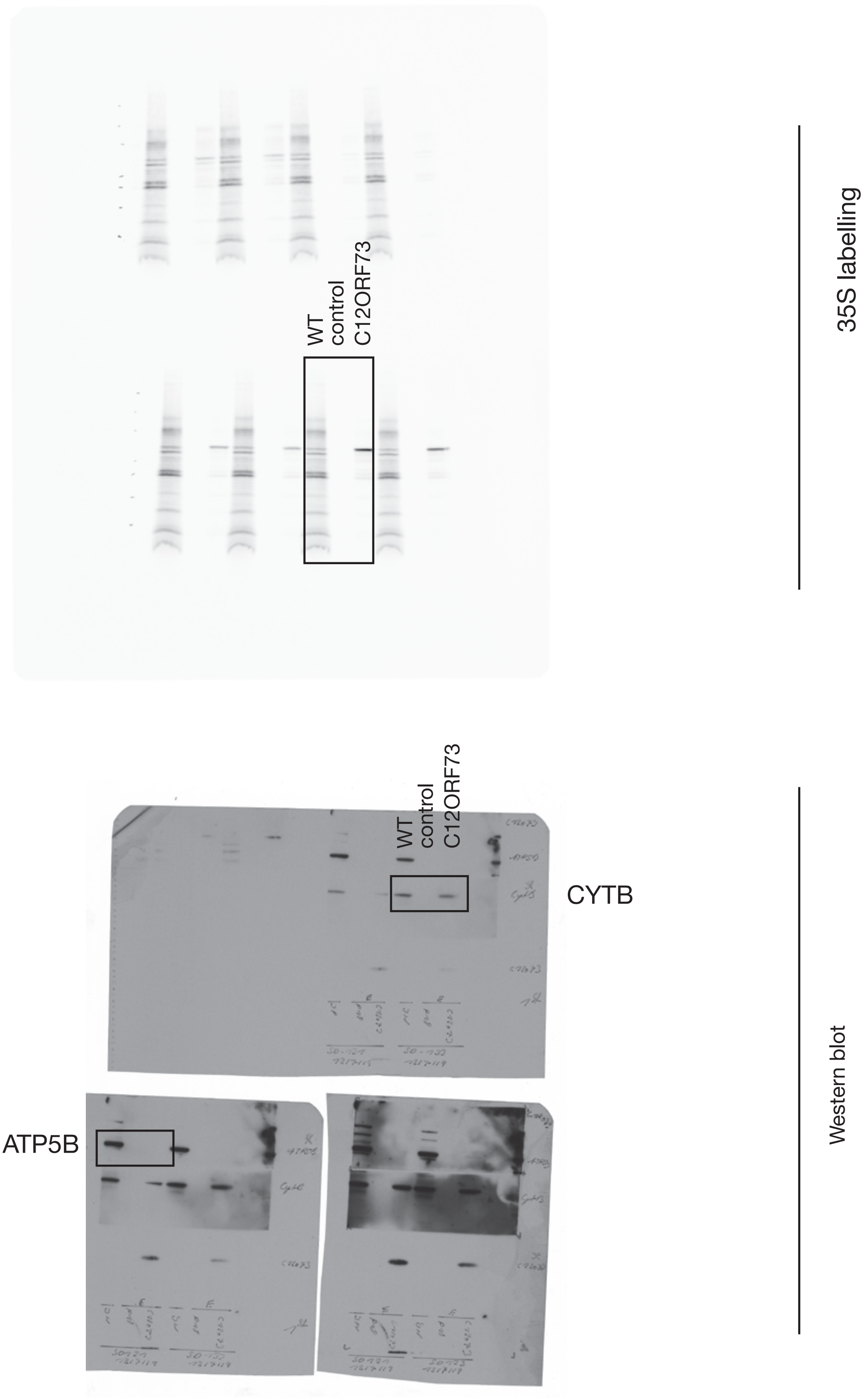

Figure 6 source data 4 related to Figure 6D

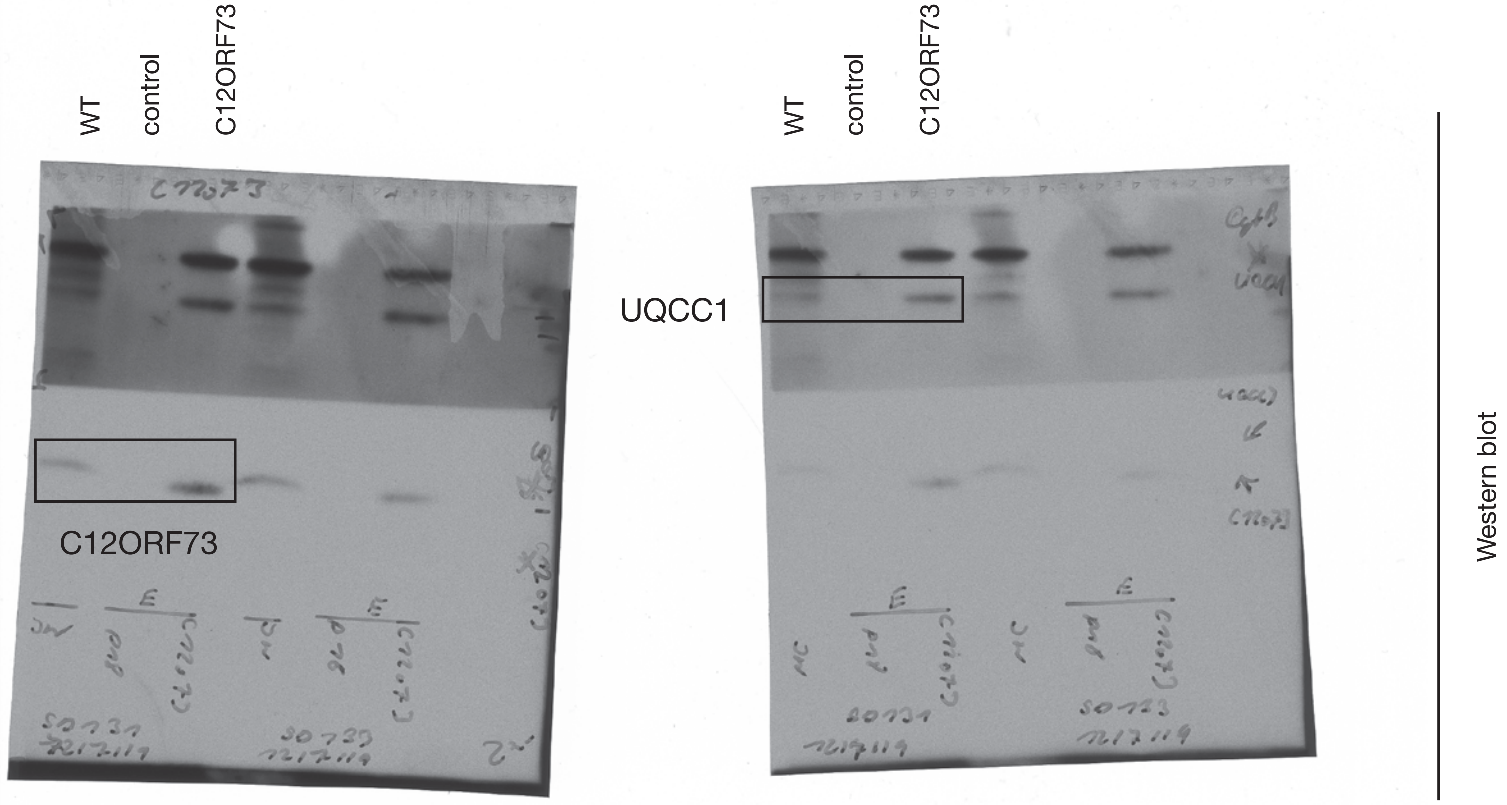

Supplement: Figure 6—source data 1. [file elife-68213-fig6-data1.zip › Figure_6_source_data/Figure_6_source_data_4_Figure_6D/Data_labelled/Figure_6_source_data_4_Figure_6D.pdf]
