# Supplementary material for: Defining the interactome of the human mitochondrial ribosome identifies SMIM4 and TMEM223 as respiratory chain assembly factors
Source: eLife. 2021 Dec 31;10:e68213. doi: 10.7554/eLife.68213 (PMC8719881; doi:10.7554/eLife.68213)
Supplement: Transparent reporting form [file elife-68213-transrepform1.pdf]

eLIFE
1st Floor
24 Hills Road
Cambridge CB2 1JP, UK
P 01223 855340
W elifesciences.org
T @elife

eLife's transparent reporting form

We encourage authors to provide detailed information *within their submission* to facilitate the interpretation and replication of experiments. Authors can upload supporting documentation to indicate the use of appropriate reporting guidelines for health-related research (see EQUATOR Network), life science research (see the BioSharing Information Resource), or the ARRIVE guidelines for reporting work involving animal research. Where applicable, authors should refer to any relevant reporting standards documents in this form.

If you have any questions, please consult our Journal Policies and/or contact us: editorial@elifesciences.org.

Sample-size estimation

- You should state whether an appropriate sample size was computed when the study was being designed
- You should state the statistical method of sample size computation and any required assumptions
- If no explicit power analysis was used, you should describe how you decided what sample (replicate) size (number) to use

Please outline where this information can be found within the submission (e.g., sections or figure legends), or explain why this information doesn't apply to your submission:

Within this study we did not use mouse models or patient material, but rather based our study on the standard tissue culture HEK293T cell line. The cell line was tested on a regular basis for mycoplasma. The culturing of the HEK293T cells is described in detail in the material and methods section. Hence, sample size estimation was not required. For each experiment we used appropriate amounts of cells or mitochondria to guaranty a reliable readout of the experiments.

Replicates

- You should report how often each experiment was performed
- You should include a definition of biological versus technical replication
- The data obtained should be provided and sufficient information should be provided to indicate the number of independent biological and/or technical replicates
- If you encountered any outliers, you should describe how these were handled
- Criteria for exclusion/inclusion of data should be clearly stated
- High-throughput sequence data should be uploaded before submission, with a private link for reviewers provided (these are available from both GEO and ArrayExpress)

Please outline where this information can be found within the submission (e.g., sections or figure legends), or explain why this information doesn't apply to your submission:

Each experiment was performed at least in triplicate as biological replicates. We define a biological replicate as by starting with the culturing of HEK293 T cells from a stock culture for each experiment individually. The stock of the HEK293T cells has been in culture for a maximum of three months. Hence, the designed experiment, such as siRNA applications, were performed with individually cultured HEK293T cells for each experiment. The obtained data are represented in the manuscript and if appropriate an statistical analysis has been performed, as it is stated within the figure legends. SEM quantification were used within the manuscript. Statistical quantifications were performed of at least three individual replicates as described in the Method section. Following good scientific practice, we included only data and figures with appropriate quality. If experiments did not have reveal a sufficient quality the experiment was repeated and not counted as replicate.

Statistical reporting

- Statistical analysis methods should be described and justified
- Raw data should be presented in figures whenever informative to do so (typically when N per group is less than 10)
- For each experiment, you should identify the statistical tests used, exact values of N, definitions of center, methods of multiple test correction, and dispersion and precision measures (e.g., mean, median, SD, SEM, confidence intervals; and, for the major substantive results, a measure of effect size (e.g., Pearson's r, Cohen's d)
- Report exact p-values wherever possible alongside the summary statistics and 95% confidence intervals. These should be reported for all key questions and not only when the p-value is less than 0.05.

Please outline where this information can be found within the submission (e.g., sections or figure legends), or explain why this information doesn't apply to your submission:

If a statistical analysis has been appropriate we calculated SEM values. This is stated within the figure legends. Statistical analysis is described in the Method section.
The statistical analysis of the mass spectrometry datasets are described in the material and method section as followed: The MS raw data were analyzed with MaxQuant/Andromeda (version 1.4.1.2 for mL62^{FLAG} and 1.5.5.1 for SMIM4^{FLAG} data; (Cox and Mann 2008; Cox et al. 2011)) and searched against the UniProt human proteome set including isoforms (release versions 08/2014 for mL62^{FLAG} and 08/2018 for SMIM4^{FLAG} data) using default settings except that the minimum requirements for protein identification and relative quantification were set to one unique peptide and one SILAC peptide pair, respectively. Arg10 and Lys8 were set as heavy labels. Carbamidomethylation of cysteine residues was considered as fixed, and N-terminal acetylation and oxidation of methionine as variable modifications. The options 'match between runs' and 'requantify' were enabled. The experiments were performed in four biological replicates including label-switch. Lists of proteins identified in the analyses of mL62^{FLAG} and SMIM4^{FLAG} complexes are provided in Supplementary Table S1 and S2, respectively and uploaded in the submission process.

(For large datasets, or papers with a very large number of statistical tests, you may upload a single table file with tests, Ns, etc., with reference to sections in the manuscript.)

Group allocation

- Indicate how samples were allocated into experimental groups (in the case of clinical studies, please specify allocation to treatment method); if randomization was used, please also state if restricted randomization was applied
- Indicate if masking was used during group allocation, data collection and/or data analysis

Please outline where this information can be found within the submission (e.g., sections or figure legends), or explain why this information doesn't apply to your submission:

Within this study we did not divide into experimental groups, since we used standard HEK293T cell for our experiments.

Additional data files ("source data")

- We encourage you to upload relevant additional data files, such as numerical data that are represented as a graph in a figure, or as a summary table

eLife Sciences Publications, Ltd is a limited liability non-profit non-stock corporation incorporated in the State of Delaware, USA, with company number 5030732, and is registered in the UK with company number FC030576 and branch number BR015634 at the address 1st Floor, 24 Hills Road, Cambridge CB2 1JP | August 2014

- Where provided, these should be in the most useful format, and they can be uploaded as “Source data” files linked to a main figure or table
- Include model definition files including the full list of parameters used
- Include code used for data analysis (e.g., R, MatLab)
- Avoid stating that data files are “available upon request”

Please indicate the figures or tables for which source data files have been provided:

We will provide the corresponding mass spectrometry data files as Excel Supplementary Table S1 and S2. These tables are generated in a logical and understandable manner, based on the enrichment of the detected protein.
